# Next generation mapping reveals novel large genomic rearrangements in prostate cancer

**DOI:** 10.18632/oncotarget.15802

**Published:** 2017-03-01

**Authors:** Weerachai Jaratlerdsiri, Eva K.F. Chan, Desiree C. Petersen, Claire Yang, Peter I. Croucher, M.S. Riana Bornman, Palak Sheth, Vanessa M. Hayes

**Affiliations:** ^1^ Laboratory for Human Comparative and Prostate Cancer Genomics, Genomics and Epigenetics Division, Garvan Institute of Medical Research, Darlinghurst, Australia; ^2^ St Vincent's Clinical School, University of New South Wales, Randwick, Australia; ^3^ Bionano Genomics Inc., San Diego, California, USA; ^4^ Bone Biology Division, Garvan Institute of Medical Research, Darlinghurst, Australia; ^5^ School of Biotechnology and Biomolecular Sciences, University of New South Wales, Randwick, Australia; ^6^ School of Health Systems and Public Health, University of Pretoria, Pretoria, South Africa; ^7^ Central Clinical School, University of Sydney, Camperdown, Australia

**Keywords:** prostate cancer, structural genomic rearrangements, next generation mapping, next generation sequencing

## Abstract

Complex genomic rearrangements are common molecular events driving prostate carcinogenesis. Clinical significance, however, has yet to be fully elucidated. Detecting the full range and subtypes of large structural variants (SVs), greater than one kilobase in length, is challenging using clinically feasible next generation sequencing (NGS) technologies. Next generation mapping (NGM) is a new technology that allows for the interrogation of megabase length DNA molecules outside the detection range of single-base resolution NGS. In this study, we sought to determine the feasibility of using the Irys (Bionano Genomics Inc.) nanochannel NGM technology to generate whole genome maps of a primary prostate tumor and matched blood from a Gleason score 7 (4 + 3), ETS-fusion negative prostate cancer patient. With an effective mapped coverage of 35X and sequence coverage of 60X, and an estimated 43% tumor purity, we identified 85 large somatic structural rearrangements and 6,172 smaller somatic variants, respectively. The vast majority of the large SVs (89%), of which 73% are insertions, were not detectable *ab initio* using high-coverage short-read NGS. However, guided manual inspection of single NGS reads and *de novo* assembled scaffolds of NGM-derived candidate regions allowed for confirmation of 94% of these large SVs, with over a third impacting genes with oncogenic potential. From this single-patient study, the first cancer study to integrate NGS and NGM data, we hypothesise that there exists a novel spectrum of large genomic rearrangements in prostate cancer, that these large genomic rearrangements are likely early events in tumorigenesis, and they have potential to enhance taxonomy.

## INTRODUCTION

Structural genomic rearrangements appear to be highly abundant and complex in the prostate cancer genome, with potential to contribute directly to oncogenic events and provide a molecular signature for subtype classification [1]. Recently, genomic rearrangements have been used to clinically subclassify primary prostate cancer [2, 3]. Accurate detection of structural variations (SVs) greater than one kilobase (Kb) in length using short-read (up to hundreds of bases) next generation sequencing (NGS) data is, however, difficult. In clinically relevant prostate cancer genome sequencing, this has been further challenged by tumor heterogeneity and frequent stromal contaminants.

Short-read NGS detection of SVs, including large deletions, insertions or duplications, inversions and translocations, is based on differences in local depth of coverage and sequence read orientation relative to a reference genome [4]. As no single informatics tool can detect the full range of SVs regarding size and subtype [5], integrated methods have been proposed [6, 7], with *de novo* assembly of tumor genomes remaining a challenge. While long-read (up to thousands of bases) sequencing methods, such as single-molecule sequencing from Pacific Biosystems (PacBio) and Oxford Nanopore, are improving SV detection [8, 9], they are still limited by relatively high costs, low throughput and relatively high error rates.

In this study we combined NGS with next generation mapping (NGM), a non-sequencing method, to capture a novel spectrum of somatic SVs that are potentially clinically relevant to prostate cancer (Supplementary Figure 1). The Bionano Genomics Inc. NGM Irys system allows for the interrogation of megabase (Mb) length DNA molecules using enzyme recognition motifs in combination with high-resolution fluorescence imaging of linearized DNA passing through nanochannels of an Irys Chip [10]. Fluorescent labels act as molecular markers allowing for the reconstruction of whole genome maps. In this first study, using NGM for the detection of somatic SVs > 1 Kb in a matched normal-tumor prostate cancer pair, we demonstrated the potential of targeted NGS interrogation for large SV validation.

## RESULTS

### Patient characterization

Patient UP2153 is a South African male of European ancestry who presented at age 63 years at the Urology Clinic of the Steve Biko Academic Hospital in Pretoria with a PSA level of 11.3 ng/mL. Histopathological analysis of prostate biopsy confirmed a diagnosis of moderately aggressive prostate cancer with Gleason score 7 (4 + 3) or Grading Group 3. The patient subsequently underwent androgen deprivation therapy and radiation therapy. There is a reported family history of prostate cancer on his maternal side, with both his sisters having a breast cancer diagnosis. Furthermore, the patient has been treated for hypertension and diabetes.

### Inherited prostate cancer risk

As patient UP2153 reported a family history of prostate cancer, we determined his inherited risk profile from 36X coverage whole genome sequencing data of whole blood, using the Illumina HiSeq X Ten platform (Supplementary Table 1). Presenting with a total of 3.6 million single nucleotide variants (SNVs) and close to 600 thousand indels (insertions and deletions < 50 bp), we examined the presence of 100 previously identified prostate cancer susceptibility alleles that were estimated to explain roughly 33% of familial risk in men of European descent [11–13]. UP2153 carried 60 of these known risk-associated alleles, of which 29 were found to be present in homozygous state (Supplementary Table 2).

As UP2153 reported a family history of breast cancer, we assessed for potentially pathogenic mutations inherited in breast cancer genes, *BRCA1* and *BRCA2*. This was motivated by the observation that, by age 65 years, men with predisposing *BRCA1* or *BRCA2* mutations are at as much as 4.5- and 8.6-fold increased risk of developing prostate cancer, respectively [14, 15], with *BRCA2* mutations further associated with poor prognosis [16]. We identified four *BRCA1* and four *BRCA2* non-synonymous mutations (Supplementary Table 3). Two *BRCA2* mutations were predicted to carry pathogenic potential, including A2951T (GCC > ACC; ClinVar ID41570) and N289H (AAT > CAT; ClinVar ID41567). However, the two mutations appear to have benign clinical significance in breast and ovarian cancer experiments [17].

### NGS-derived somatic variation and molecular subclassification

The availability of 69X coverage whole genome Illumina HiSeq data of the tumor, in addition to the blood (Supplementary Table 1) allowed for detection of somatic variants (Table 1). We identified a total of 6,123 small somatic variants, including 5,981 SNVs and 142 indels (Supplementary Table 4). To maximise for the detection of larger deletions, insertions / duplications and inversions (> 50 bp), we used five separate SV calling tools, specifically Breakdancer (read-pair) [18], Pindel (split-read) [19], CNVnator (read-depth) [20], as well as read-pair and split-read integration tools, Manta [21] and Lumpy [22], collating our findings using MetaSV [6] which required an SV to be detected by at least four reads and two NGS-based SV callers [reviewed in 23]. Due to limitations of short-read NGS data for detecting SVs in high repeat regions [24], we performed post-call filtering to remove low complexity regions, followed by manual inspection (Supplementary Table 5), identifying 45 deletions and four duplications under 1 Kb (Supplementary Table 6), and 26 deletions, seven duplications and a single inversion greater than 1 Kb in length (Supplementary Table 7).

**Table 1:** Number of NGS-derived somatic variants in UP2153

≤ 50 bp	SNVs/indels (*Strelka*)	Functional Potential^a^	Oncogenic Drivers (*TransFIC*)^b^	Oncogenic Drivers (*CanDrA*)^b^	> 50 bp < 1 Kb	SVs (*MetaSV*)	Functional Potential^c^ (*GEMINI*)	Oncogenic Potential^d^	≥ 1 Kb	SVs (MetaSV)	Functional Potential^c^ (*GEMINI*)	Oncogenic Potential^d^
SNVs	5981	23	4	4	**DEL**	45	7	1	**DEL**	26	10	6
DEL	62	0	0	0	**DUP**	4	0	0	**DUP**	7	0	0
INS	80	1	0	0	**INV**	0	0	0	**INV**	1	0	0
***Total***	**6123**	**24 (0.4%)**	**4 (0.07%)**	**4 (0.07%)**	***Total***	**49**	**7 (14%)**	**1 (2%)**	***Total***	**34**	**10 (29%)**	**6 (18%)**

*Abbreviations*: bp, base pairs; Kb, kilobases; SNVs, single nucleotide variants; DEL, deletion; INS, insertion; DUP, duplication; INV, inversion.

^a^Using functional prediction tools GEMINI, SIFT and PolyPhen2.

^b^Driver mutation prediction using TransFIC and CanDrA identified a single mutation by both methods within *ATRX* gene.

^c^Defined as ‘HIGH’ impact by the GEMINI sequence ontology.

^d^Inspection of GeneCards and PubMed identified the following genes with known oncogenic potential, namely *MAP3K5, NID1, SMAD1, ZFR, ROR2, WWC1* and *RAD51B*.

A recent analysis of 333 prostate cancer exomes from The Cancer Genome Atlas (TCGA) proposed seven major molecular prostate cancer subclassifications based on four fusion genes and three recurrent oncogenic mutations [3]. No fusion events were found involving the E26 transformation specific (ETS) family of transcription factors, specifically *ERG, ETV1, ETV4* and *FLI1*. While ETS-fusion negative tumors are reportedly more likely to present with recurrent mutations in *SPOP* (coding for Speckled-type POZ protein), *FOXA1* (coding for Forkhead box A1) or *IDH1* (coding for isocitrate dehydrogenase-1), no putative somatic mutations within these genes were detected in this patient (Supplementary Tables 4, 6 and 7). Therefore, UP2153 falls among the 26% of primary prostate cancers that are molecularly unclassified based on TCGA subclassification.

**Figure 1:**
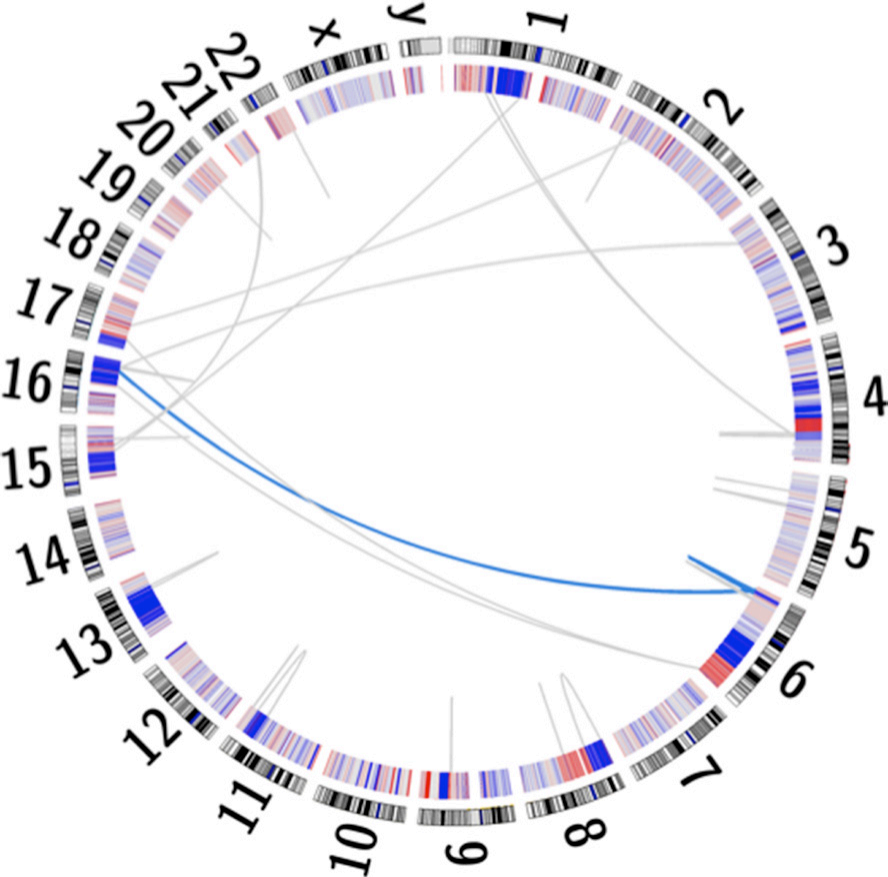
Circos plot depicting the human karyogram with coordinated chained events and SCNAs in the UP2153 tumor Somatic copy number gains (red) and losses (blue) are depicted in the inner ring, while a single coordinated chained event between chromosomes 6 and 16 (blue) and rearrangements not assigned to a chained event (gray) are depicted as lines within the plot.

Additionally, somatic copy-number alterations (SCNAs) are reportedly enriched within prostate cancer [25]. A total of 1,815 losses and 748 gains were identified within UP2153 tumor, with marked copy number losses throughout chromosomes 6, 8, 13 and 16 and gains in chromosome 8 (Figure 1 and Supplementary Figure 2). Our finding is consistent with chromosome 6 and 16 losses and chromosome 8 gains observed in the 26% of TCGA unclassified prostate cancers [3]. Notably, the chromosome 16 losses did not span the *CDH1* locus, while the chromosome 8 losses did not span the *MYC* locus.

Using the combined somatic SNV and SCNA data, we estimated tumor purity of 43.3–45.0% (Supplementary Figure 3A and 3B) and clonality at a single clone (Supplementary Figure 3B). Functional potential was predicted for all NGS-derived somatic variants (Table 1). We identified 23 SNVs (19 non-synonymous, 3 stop gained and 1 splice donor variants) and a single base insertion, consistent with those published [1, 3, 26]. Seven deletions < 1 Kb (range 224 to 684 bp) and 10 deletions > 1 Kb (range 17,313 to 1,565,842 bp) were predicted to result directly in exon loss, splice junction disruption or frameshift. Oncogenic potential (tumor driver mutational status) was predicted for four functional SNVs using two available tools. TransFIC identified oncogenic potentials in *AT2A1, NOTCH2*, *ZN462* and *ATRX* genes, and CanDrA in *CNTN6, HCRTR2, OCSTAMP* and *ATRX* genes (Supplementary Table 8), with a single gene overlap. We predicted oncogenic potential for a 333 bp deletion causing a frameshift in the apoptotic gene *MAP3K5*, previously shown to be differentially expressed in prostate cancer [27]. Six of the NGS-predicted deletions > 1 Kb result in exon loss within genes of known oncogenic potential, including a 59 Kb deletion in the prostate cancer risk associated DNA repair gene *RAD51B* [28], a 209 Kb deletion in the tyrosine kinase receptor gene *ROR2* shown to be depleted in metastatic prostate cancer [29] and a 826 Kb deletion of the androgen receptor corepressor gene *SMAD1* [30].

In addition to small and larger somatic SNVs, deletions, insertions / duplications, inversions, and SCNAs, larger chromosomal rearrangements are observed in prostate cancer, with chromoplexy, involving coordinated chains of rearrangements between chromosomes, being the most common and possibly unique phenomenon in prostate cancer [1]. While more common to EST-fusion positive (specifically *ERG*-fusion positive) tumors, ETS (*ERG*)-fusion negative tumors, particularly those shown to have deletions within *CHD1*, are more likely to display chromothriptic features, which are complex patterns of hundreds of clustered rearrangements resulting in inaccurate reassembly of a single chromosome. Using ChainFinder [1], we showed the ETS-fusion negative *CHD1*^WT^ UP2153 tumor to display a single chained event involving three gene fusion-causing SVs, specifically two inter-chromosomal translocations between chromosomes 6 and 16 and a single chromosome 6 deletion (Figure 1). Within this single event, a total of 35 genes were potentially deleted and/or rearranged (Supplementary Table 9).

### NGM-derived somatic structural variation

Both tumor and matched blood samples were genome mapped, using high molecular weight (HMW) DNA prepared for NGM with half being sheared for sample matched NGS data generation. Using the Bionano Irys platform, an average mapped depth of coverage of 35X and 68X was achieved for the tumor and blood, respectively (Supplementary Table 10). Molecules were assembled into genome maps with > 50% of genome maps (Map N50) greater than 0.52 Mb and 1.33 Mb for the blood and tumor samples respectively. SVs were independently called for the two samples relative to the *in silico* digested Hg19 reference map, and somatic SVs determined as those present only in the tumor. Unlike NGS, which is effective for detecting small variations [31], the resolution of NGM is limited to detecting variants greater than 1 Kb [32]. We found 85 somatic SVs > 1 Kb, including 59 insertions (range from 2 Kb and 100 Kb) and 26 deletions (range from 3 Kb and 75 Kb), impacting roughly 1.6 Mb of the total tumor genome (Table 2 and Supplementary Table 11).

**Table 2:** Verification of NGM-detected somatic SVs >1 Kb in UP2153 using short-read NGS data

> 1kb SVs	NGM SVs (*IrysSolve*)	Affected Genes^a^	NGS verification				
			NGS coverage^b^	MetaSV Evidence (≥ 4 reads)^c^	Manual read inspection (< 4 reads)	Evidence from NGS assembly	Total SV verified
**DEL**	26	14	26	6	20	7	26 (100%)
**INS/DUP^d^**	59	33	58	3	42	35	52 (90%)^3^
***Total***	**85**	**47 (55%)**	**84**	**9 (11%)**	**62 (74%)**	**42 (50%)**	**79 (94%)**

Abbreviations: SV, structural variation; DEL, deletion; INS, insertion; DUP, duplication; INV, inversion.

^a^ Predicted based on co-location of SV with UCSC known canonical genes.

^b^ One insertion showed no or minimal read or scaffolds coverage and was removed from the verification set.

^c^ MetaSV called somatic variants based on four or more reads and observed using two or more informatic SV callers including Manta, Breakdancer, Lumpy, CNVnator and/or Pindel for SVs > 1 kb.

^d^ All insertion variants were called duplications using NGS except one variant, which was called as a deletion.

**Figure 2:**
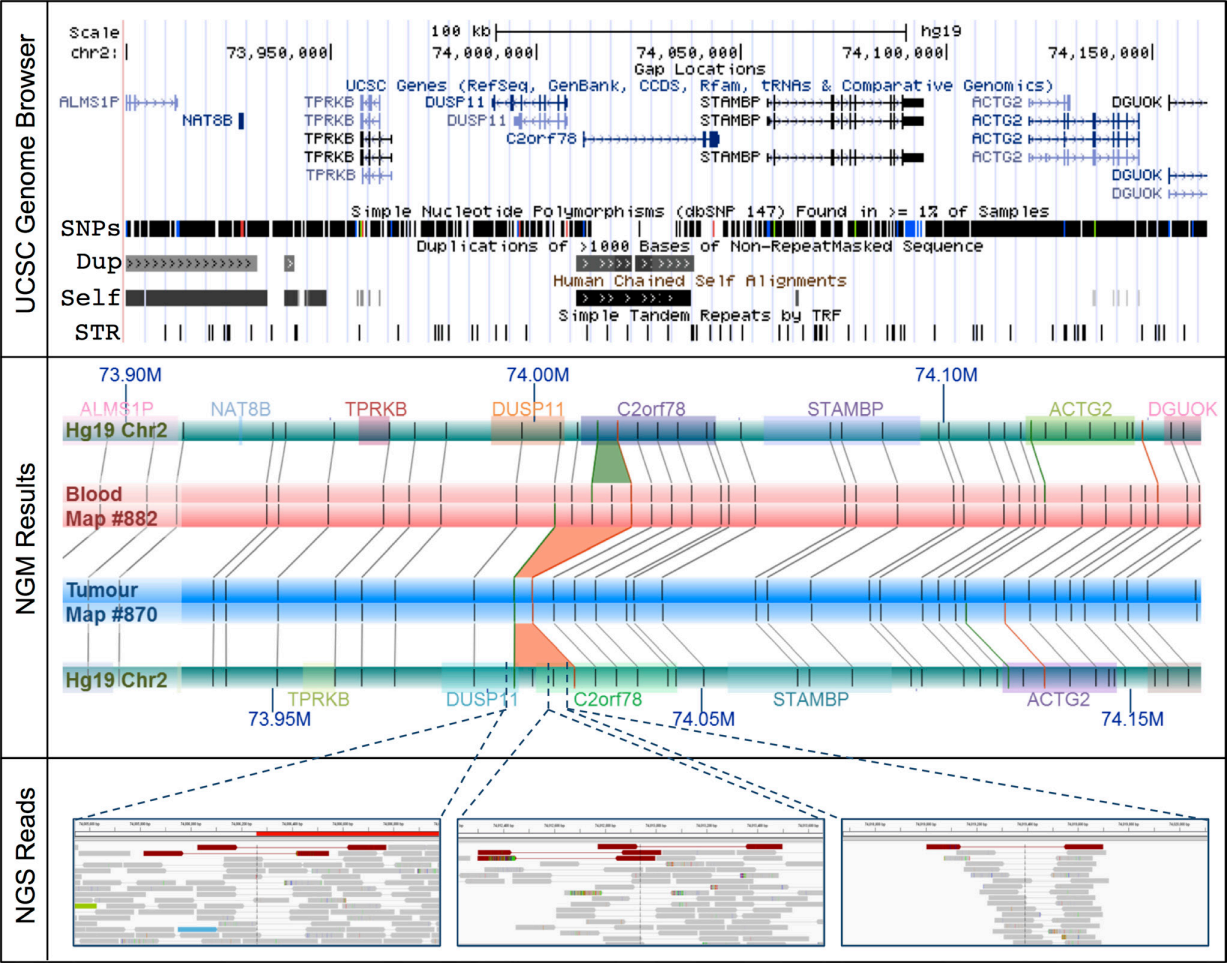
The *DUSP11-C2orf78* gene fusion event identified using NGM involves a 14.3 Kb somatic deletion at Chr2: 74.006–74.020 Mb (*Upper Panel*) The deletion is embedded within known segmental duplications and self-chains, overlapping both *DUSP11* and *C2orf78* genes. (*Middle Panel*) Rectangular tracks (horizontal bars) represent *in silico* genome map for Hg19 (green) and consensus genome maps for the tumour (blue) and matched blood (red). Hg19 genomic coordinates are indicated with dark blue font (M = Megabase). Overlaid on the Hg19 track are gene annotations, represented and distinguished by colored rectangles; gene symbols are indicated above with matching colors. Irys enzymatic labels (nick sites) are shown as vertical grey bars overlaid on the genome map tracks, and alignments of labels are shown as grey connecting lines. NGM called INS and DEL are highlighted, respectively, as green (4.8 Kb insertion in blood relative to Hg19) and orange trapezoids between aligned genome maps (9.8 Kb and 14.3 Kb deletions in tumor relative to Hg19 and matched blood, respectively). (*Bottom Panel*) In the NGS (IGV) panels, the tracks are alignments of reads, in grey. Orientation of sequencing reads are indicated by blunt ends for 5′ end and arrow end as 3′ end. Several single NGS reads with discordant alignments to Hg19 provide evidence for the deletion (red) in the tumor sample.

Over half of these large SVs (55%) are collocated with known genes, potentially disrupting 47 (Supplementary Table 11). Of these, 37 (36%) have been linked and/or shown to play a role in tumorigenesis. Significant examples include, a single 14 Kb deletion at chromosome 2: 74,006,259–74,020,290 resulting in a potential novel prostate cancer fusion *DUSP11-C2orf11* (Figure 2) and a 4 Kb insertion within the *CHL1* gene at chromosome 3: 284,661–305,427 (Supplementary Figure 4). *DUSP11* encodes for a dual specificity phosphatase (DUSP), of which the gene family has been shown to have important roles in the mitogen-activated protein (MAP) kinase pathways in prostate cancer, with changes in DUSP expression associated with prostate cancer cell survival/death [33]. The *CHL1* gene, which encodes for a neural cell adhesion molecule, has been directly implicated in prostate cancer predisposition [34] and shown to play a dual role in the tumorigenesis of major cancer types, specifically acting as a tumor suppressor gene during early growth and an oncogene during invasive and metastatic growth [35]. Neither of these tumorigenic candidates were detected using NGS.

### Verification of NGM-derived somatic structural variation using NGS data

**Figure 3:**
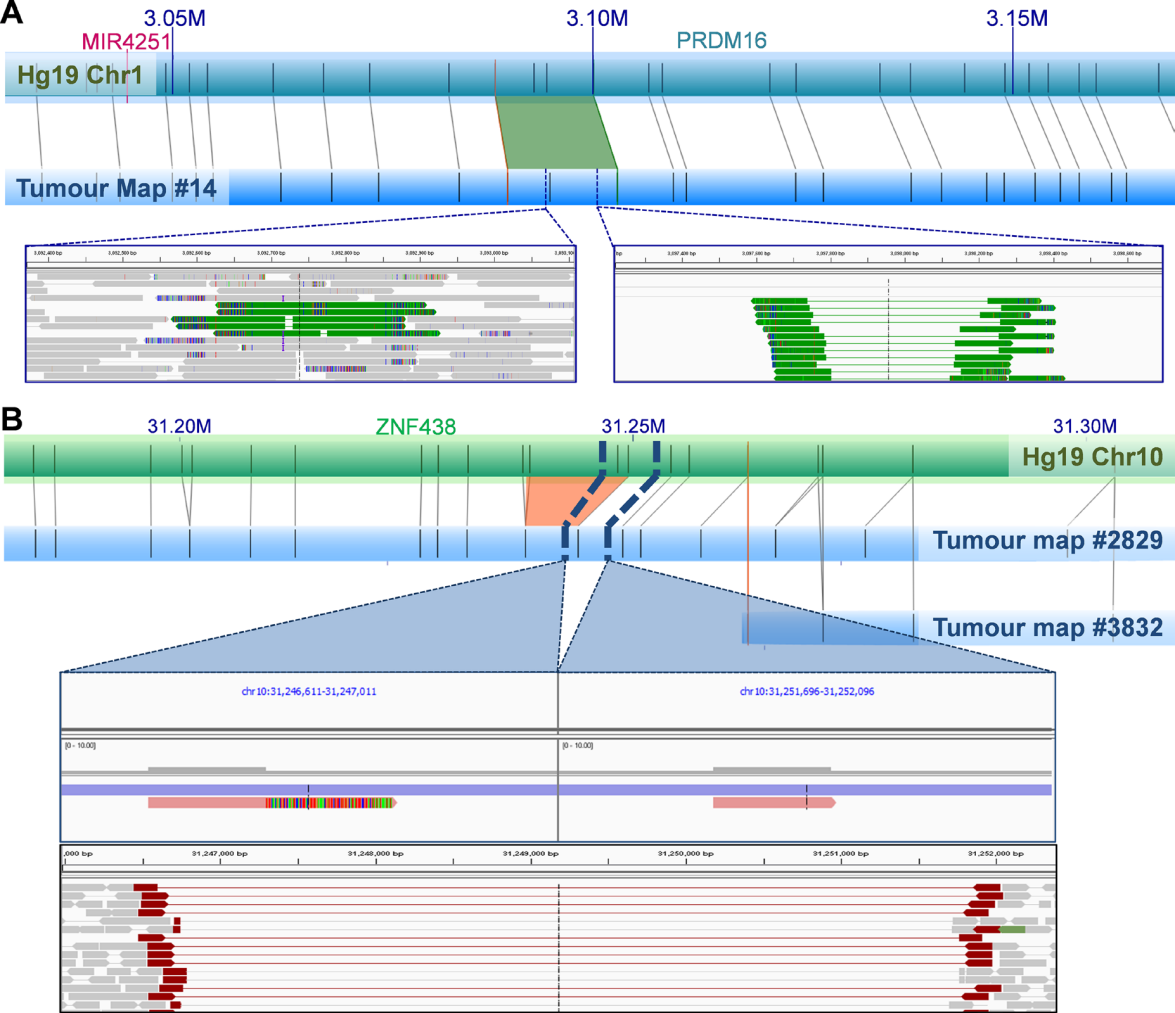
Examples of NGM-derived UP2153 somatic SVs with NGS support (**A**) A 1.4 Kb NGM-derived somatic insertion (green trapezoid) within *PRDM16* gene at Chr1: 3.088–3.100 Mb in the tumor consensus map relative to Hg19 *in silico* genome map, with alignment of enzymatic labels (nick sites) shown a grey connecting lines. NGS verification included a 223 bp and 760 bp duplication (represented by green tracks) identified by MetaSV (left inset), and from IGV manual inspection of sequencing reads that show evidence of insertion within the region (right inset). (**B**) A 4.8 Kb NGM-derived somatic deletion (orange trapezoid) within *ZNF438* gene at Chr10: 31.233–31.249 Mb in the tumor consensus map (blue horizontal bar) relative to Hg19 (green horizontal bar), was further verified by IGV manual inspection of sequencing reads (bottom panel of inset) and assembled scaffolds (top panel of inset). Note two haplotypes (scaffolds) are observed in the tumor genome assembly, corresponding to the normal (likely from stromal contamination) and mutant haplotypes (from the tumor).

A single NGM-called insertion showed no available NGS-derived read coverage. Of the 84 NGM-derived SVs with available NGS data, only a single 5 Kb deletion overlapped with a larger 3 Mb NGS-derived deletion determined using our previously described five-tool filtered pipeline. Lack of overlap and size distribution difference was not surprising as 81 (96.4%) of the 84 NGM-derived SVs were found in low complexity regions, subjected to NGS call filtering during low confidence removal. To enhance verification, we applied a relaxed NGS tumour only approach using the previously described five-tool MetaSV analysis (Table 2), identifying nine of the NGM called SVs (11%) from 872 deletions, 238 duplications, and 140 inversions, of which three deletions (involving genes *THSD4, ZNF438* and *TBCK*) and one insertion (involving gene *PRMD16*, Figure 3A) impacted known genes.

Due to low tumor purity (< 50%), it is plausible that NGS signals of some SVs would be too weak to be identified. To better understand the occurrence of the remaining 20 deletions (77%) and 55 insertions (95%) in the NGS data, we visually inspected these regions. In the vast majority of cases, evidence of the candidate SVs were in fact present albeit in only a few sequencing reads. This is predominantly due to the SVs being present in “low complexity” regions of the genome, such as regions with high repeat contents and non-unique sequences. The *DUSP11-C2orf78* SV is a good example (Figure 2), wherein the 9.8 Kb deletion is located within a segmental duplication and known self-chain (non-unique genomic sequence), causing high dropout of many sequencing reads within the region as a result of low mapping scores. Targeted inspection showed multiple discordant read-pairs of small deletions. Low frequencies of discordant reads were observed for 20 (77%) of the deletions and 42 (72%) of insertions (Table 2). Appreciating short sequence reads is the biggest limitation to SV detection using NGS, we additionally performed *de novo* NGS assembly of the tumor and normal samples, generating 14,622 and 12,362 scaffolds larger than the N50 length (the length at which > 50% of the scaffolds exceed) of 56,903 and 67,039 bp respectively (Supplementary Table 12). Manual scaffold interrogation of NGM-derived SV regions verified half the detected SVs (see example in Figure 3B), increasing the total number of verified deletions to 100% and insertions to 90%.

## DISCUSSION

Advances in NGS, has dramatically facilitated our ability to define genetic alterations acquired during primary prostate cancer tumorigenesis. In particular, a new molecular subclassification has emerged, providing for the first time potential to define clinical courses for this highly heterogeneous disease. In an analysis of 333 individual prostate cancer exomes, TCGA identified seven subtypes defined either by gene fusions involving the ETS family (59% of cases), specifically *ERG*, *ETV1*, *ETV4*, or *FLI1*, or recurrent mutations (15% of cases) in *SPOP*, *FOXA1*, or *IDH1* [3]. Negative for all seven subclassifications, we speculate the prostate tumor in UP2153, which harbour the expected extent of SCNAs and chained chromosomal rearrangements, is driven by an as yet undefined molecular alteration. The aim of this study was to characterize a near-to-full spectrum of molecular alterations acquired within a single intermediate stage (Gleason score 7), most commonly presenting, primary prostate tumor. To achieve our goal, we adopted a new, yet complimentary technology to NGS, providing an alternative non-sequencing approach focused on the detection of large genomic rearrangements. We generated not only high-coverage whole genome sequencing (WGS), but also the first complete NGS-matched whole genome mapped (WGM) prostate tumor-normal genome pair.

Based on the presence of 60 prostate cancer specific risk alleles, our patient presents with a classical somatic mutational burden for an unclassified intermediate stage primary prostate tumor as determined from publically available whole exome/genome sequencing efforts [1, 3, 26]. Specifically, we observed 1.057 somatic SNVs/indels per Mb, including 19 non-synonymous, three termination gain and one splice-donor variants. While none of the impacted genes have previously been identified as recurrently mutated, we predict for the possibility of novel prostate tumorigenic potential using oncogenic driver mutation identification tools. A single potential prostate cancer driver mutation, possibly a novel molecular subclassifier, was identified using two independent computational methods. The *ATRX* L2237P mutation (chromosome X: 76,778,869 A>G) observed on the single male-derived X chromosome, lies within the gene encoding for the alpha thalassemia/mental retardation syndrome X-linked protein implicated in chromatin remodeling. Classified as a tumor suppressor gene, *ATRX* is frequently mutated in neuronal-related cancers, such as neuroendocrine cancers [36, 37], gliomas [38, 39], and neuroblastoma [40–42], and more recently also in an aggressive form of smooth muscle tumors [43, 44]. Other potentially clinically relevant genes with single base somatic mutations include *NOTCH2*, recently proposed as an oncogene in bladder cancer [45], with the Notch family pathway implicated in prostate tumorigenesis [46], and *CNTN6* within the 3p26 prostate cancer susceptibility locus [47]. A somatic mutation resulting in the loss of 333 bp within the coding region of *MAP3K5*, a gene previously shown to be differentially expressed in prostate cancer [27], was also observed. Other gene members of the MAP (mitogen-activated protein) kinase pathway, including *MAP3K1* and *MAP3K7*, have been shown to have recurrent deletions in prostate cancer. The co-deletion of the *MAP3K7* and *CHD1* loci has been associated with aggressive ETS-fusion negative tumors [48, 49]. Notably, the overall percentage of oncogenic potential of identified somatic variants increased with variant size, with six NGS-derived SVs over 1 Kb in size showing oncogenic potential. None of these variants, however, were observed using NGM.

While NGS is ideal for capturing small genomic variants, NGM allows for capturing of kilobase to megabase SVs, a class of variants increasingly recognised as a key player in tumorigenesis. A major advantage of NGM is the ability to observe long intact DNA molecules that can be assembled into megabase length consensus genome maps, and hence the identification of large somatic SVs. Using this novel platform we identified 85 large somatic events (range from 2 Kb to 101 Kb), including twice as many insertions as deletions. Over half the SVs co-locate with a known gene or genes, with a single deletion predicted to result in a novel fusion event *DUSP11-C2orf11* (Figure 2). The tumorigenic potential of this fusion event may be speculated via DUSP significance in MAP kinase pathways in prostate cancer [33]. Direct verification of these large SVs using short-read NGS is problematic and biased towards deletions, with only one (0.1%) and nine somatic events (11%) verified using our multi-tiered NGS-based SV calling pipeline, for filtered and unfiltered NGS data respectively. Detection is further complicated by excessive stromal contamination resulting in tumor purity less than 50%. Although NGM does not provide specific sequence information, knowledge of approximate genomic coordinates of the candidate SVs allow for manual inspection of NGS data at the read or assembly level, the latter facilitated by *de novo* assembly of the NGS data. Using this approach, we verified all 26 large deletions and over 90% (47/52) of the large insertions. Our data is comparable with recent reports validating Bionano Irys NGM-called SVs with SVs called using Illumina short read NGS [50], PacBio long read NGS [51] or PacBio sequenced SV-targeted BAC contigs [52], or a combination of short read NGS SV caller analyses and presence within the Database of Genomic Variants (DGV) [53]. From these studies, we expect at most a 15% false positive rate. Based on this assumption, all somatic SVs were manually inspected and SVs only included with strong evidence from both NGM and NGS.

**Figure 4:**
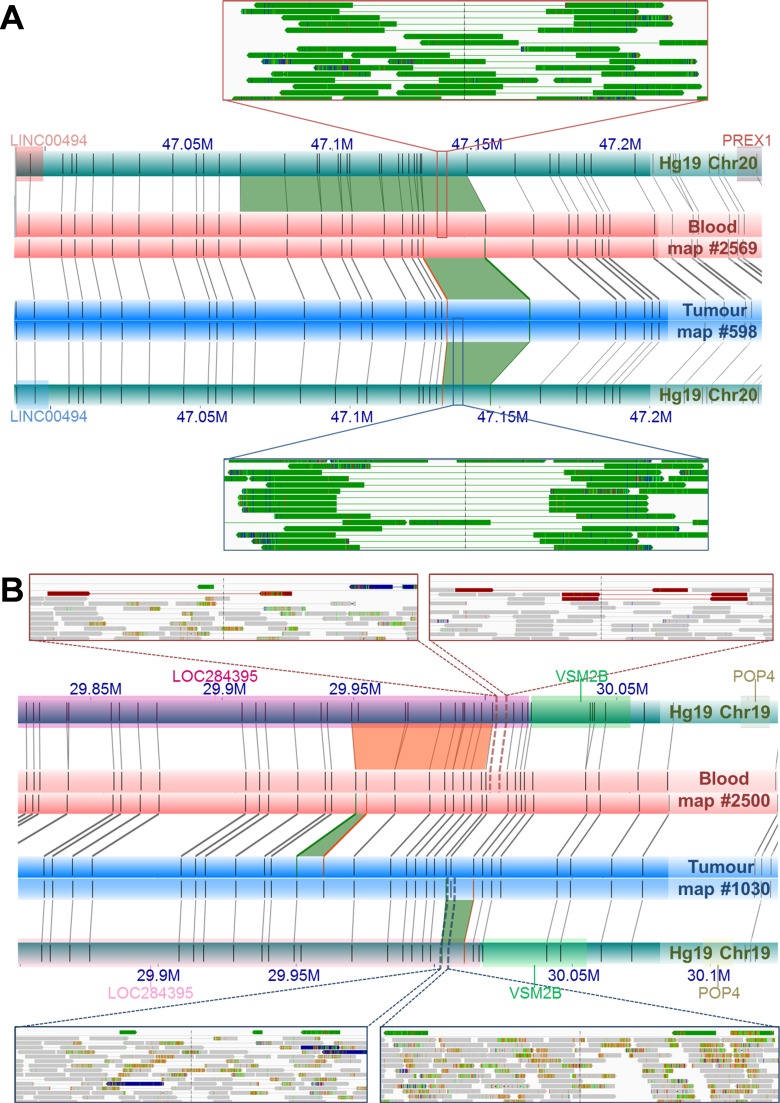
Examples of NGM-derived somatic SVs found in UP2153 with confounding calls by direct tumor-blood comparisons, compared to SV calls relative to Hg19 (**A**) Comparing the tumor (blue horizontal bar) and blood genome maps (red horizontal bar) directly, identified a 7.3 Kb somatic insertion (green trapezoid) at Chr20: 47.131–47.147 Mb. Relative to Hg19 reference the (aqua horizontal bars), the NGM IrysSolve Pipeline identified a 11.5 Kb insertion (green trapezoid) at Chr20: 47.130–47.147 Mb in the tumor and a 6.6 Kb insertion (green trapezoid) at Chr20: 47.068–47.147 Mb in the blood. Several sequencing reads provided NGS support for these insertions (green tracks) in both the blood (top inset) and tumor (bottom inset). (**B**) Direct comparison between the tumor (blue horizontal bar) and blood genome maps (red horizontal bar) identified a 6.3 Kb somatic insertion (green trapezoid) at Chr19: 2 9.949–29.952 Mb, within *LOC284395* (pink rectanglular gene annotation). Relative to the *in silico* reference genome map Hg19, the NGM IrysSolve Pipeline identified a 2 Kb insertion (green trapezoid) at Chr19: 30.003–30.011 Mb in the tumor and a 4 Kb deletion (orange trapezoid) at Chr19: 29.949–300.003 Mb in the blood, each supported by several sequencing reads corresponding to deletions (red tracks, top insets) and duplications (green tracks, bottom insets), with aligned reads in grey.

The dominant NGS-based SV calling approaches are reliant on initial alignments of individual sequencing reads to a reference genome. Although *de novo* assembly of short read NGS data is possible, as applied in this study, it is well established that this data is far from complete, generating large gaps largely as a result of repetitive regions and segmental duplications, which in tumor genomes, is further impacted by mutated regions and heterogeneity in sequence coverage [reviewed in 54]. As a non-sequencing technology, NGM overcomes these limitations by spanning these gapped regions, and in turn allowing for somatic variants to be called directly by comparing matched tumor/normal genome maps in the absence of a reference genome. The advantage of a reference-free approach is important for somatic variants that collocate with germline or natural variants. The identification of the novel *DUSP11-C2orf11* gene fusion on chromosome 2 is an excellent example (Figure 2). While observing a 9.8 Kb deletion in the UP2153 tumor relative to Hg19, we also found a 4.8 Kb insertion in the blood sample relative to Hg19. Through a direct comparison of tumor and blood genome maps, this somatic SV appears to be a 14.3 Kb deletion, with repeated label patterns that might correspond to a recent segmental duplication identified in the human genome [55]. Two additional examples highlighting the advantage of using tumor-matched blood as the mapped reference are shown in Figure 4. At chromosome 20 between approximately 47.130 Mb and 47.146 Mb (Figure 4A), 11.5 Kb and 6.5 Kb insertions were found in the tumor and blood samples, respectively, when using Hg19 as the reference. Due to their collocation, a somatic SV would likely not have been called, and the concurrent insertion would very likely have been classified as a germline mutation persisting in the tumor. However, when directly comparing tumor and blood, a 7.3 Kb somatic insertion was identified. Another example is the collocation of a 2 Kb insertion in the tumor and a 4 Kb deletion in the blood sample relative the Hg19, at chromosome 19 between 29.949 Mb and 30.002 Mb, which we see evidence of in NGS data (Figure 4B). When compared directly, a 6.2 Kb insertion was found at chromosome 19: 29,949,207–29,969,680.

Of the 46 genes spanning NGM-derived somatic SVs, excluding the previously discussed fusion event, 15 have been associated with a variety of cancers including; *ACTR3B, CELF2, CHL1, TSPYL2, LINGO1, MAGEA4, OLFM1, PDGFRA, PRDM16, PRKCA, RPS6KA6, SLC13A2, TBCK, UCK2*, and *ZMAT4*. What is notable is the potential of two oncogenic driver events within the known 3p26 prostate cancer susceptibility locus [47]. Specifically, UP2153 carries not only a NGS-derived somatic non-synonymous (T103I) mutation in *CNTN6* (chromosome 3: 1,269,627 C > T), but also a NGM-derived 4 Kb somatic insertion within the tumor suppressor gene *CHL1* (chromosome 3: 284,661–305,427, Supplementary Figure 4). Both *CNTN6* and *CHL1* code for cell-adhesion proteins implicated in tumorigenesis. Another notable large somatic mutation is a 12.5 Kb insertion within *PDGFRA*, which contributes to a known gene fusion in eosinophilic leukemia [56] and has been expressed in small cell neuroendocrine carcinoma of the prostate [57].

In conclusion, we have generated a near to complete genome profile for a single patient with intermediate grade prostate cancer and an as-yet unexplained molecular taxonomy. This study is the first to generate a complete mapped prostate cancer genome to complement deep whole genome sequencing. Using nanochannel-based genome mapping technology, we have detected a novel spectrum of large genomic rearrangements, with over double the number of insertion to deletion events (59:26). Although only a small fraction were also detected using a standard NGS-based SV detection approach, we found anecdotal evidence in the NGS data for 94% of the large SVs, of which over a third directly impacts a gene or genes with known oncogenic potential, including a novel fusion event. Thus, we have provided the first evidence that the new NGM technology has the potential to uncover a broader spectrum of potentially oncogenic prostate cancer genomic driver events that has been under-detected using NGS alone, with significant potential for further prostate cancer subclassification.

## MATERIALS AND METHODS

### Ethics

At time of diagnosis, patient UP2153 was consented for genomic research under project approval number 43/2010 of the University of Pretoria Faculty of Health Sciences Research Ethics Committee (with US Federal wide assurance FWA00002567 and IRB00002235 IORG0001762). A blood sample and prostate biopsy core were snap frozen at time of diagnosis. Samples were shipped under the Republic of South Africa Department of Health Export Permit, in accordance with the National Health Act 2003 (J1/2/4/2 No 1/12), to the Garvan Institute of Medical Research in Australia. Samples were processed, genome sequenced and genome mapped in accordance with St Vincent's Hospital (SVH) Human Research Ethics Committee (HREC) site-specific approval (SVH 15/227).

### High-molecular weight DNA extractions

High-molecular weight (HMW) DNA was extracted using the IrysPrep™ Plug Lysis Long DNA Isolation Protocol (Bionano Genomics Inc.) from frozen whole blood and prostate tissue. The blood was thawed at room temperature, lyzed to remove red blood cells and recover white blood cells. Approximately 9 mg of OCT embedded tissue was thawed and homogenized with a dounce tissue grinder leaving a cell pellet. For both samples, HMW DNA was extracted by embedding cells in agarose plugs and using components from the Bio-Rad Plug Lysis Kit in a modified plug lysis method. The latter minimises physical shearing during lysis and overnight Proteinase K digestion allows for optimal recovery of megabase DNA [58].

After multiple stabilization and TE washes, followed by melting of agarose plugs and treatment with GELase enzyme (Epicenter), HMW DNA was released and further purified by drop dialysis. A mixing step was performed to ensure that DNA homogeneity was achieved overnight prior to quantification using the Qubit^®^ BR (Broad Range) dsDNA Assay Kit (Thermo Fisher Scientific). HMW DNA was then further prepared for whole genome sequencing and whole genome mapping.

### Next generation sequencing

HMW DNA for the blood and tumor tissue was independently sheared and used as starting materials for the Nextera TruSeq Library Preparation with PCR amplification. Each sample underwent 2 × 150 cycle sequencing on an Illumina HiSeq X Ten instrument generating a mean coverage of 36.5X for the blood and 69.06X for the tumor, with a mapped read rate of 98.01% and 98.33%, respectively (Supplementary Table 1).

Sequenced reads were adapter-trimmed using Illumina's Bcl2fastq2 Conversion software (http://www.illumina.com/) and filtered using cutadapt v1.9 [59] to remove bases < Q15, reads < 70 bp and missing paired-reads. Filtered reads were aligned to the human reference genome Hg19 (http://hgdownload.soe.ucsc.edu/goldenPath/hg19) using bwa-mem v0.7.12 [60]. The GATK Pipeline v3.5 [61, 62] was used for identifying duplicate reads, performing local re-alignment at indel intervals, base quality score recalibration and co-realignment of the tumor-blood pair. Mapping statistics were calculated using QualiMap v2.1.3 [63].

### Inherited risk predictions

Read alignment of the blood sample was assessed for germline variation using FreeBayes v1.0.2 [64] and VarScan v2.3.9 [65] to generate a consensus set of SNVs and indels. Germline variants of UP2153 were examined for the presence of 100 known risk alleles for prostate cancer [11] and annotated using GEMINI v0.18.3 [66] for their genes, functional features and loss of functions. All the risk and deleterious variants observed in UP2153 were manually inspected using Integrative Genomics Viewer (IGV) [67].

### Detecting somatic SNVs and indels using NGS

Co-realigned alignment of the tumor and blood data were examined for small somatic variants (SNVs and indels) using Strelka v1.0.11 [68] and variants were reported if they showed *i*) homozygous references and non-reference alleles in the blood and *ii*) at least 20 reads observed in both the blood and matched tumor samples across variant positions. Functional impact of somatic variants was annotated using GEMINI v0.18.3 [66]. Missense mutations were further annotated using either the TransFIC (http://bg.upf.edu/transfic/home) or CanDrA software with prostate cancer-specific databases [69].

### Detecting SCNAs using NGS

Binned copy number and segmentation data of the study tumor, compared to the matched blood, were computed using the copy number calling pipeline of alignment data in the CNVkit package [70]. Cutoffs of log_2_ copy number ratios between −0.25 and +0.2 were set to assign genome losses and gains, respectively. Estimation of stromal cell contamination and ploidy was calculated using the Sequenza software, where the patient's copy number profiles and frequency of germline heterozygous SNVs were used for the estimation [71]. To estimate the number of tumor subclones, the segmentation data of the tumor and germline allele frequency were computed using the THetA2 program [72]. The estimate of tumor purity corrected by the clone number was also calculated using this program, for a comparison with the Sequenza.

### Detecting somatic chained rearrangements and deletions using NGS

Chromoplexy rearrangements, where inter-chromosomal breakpoints have been chained with other SVs, were examined using ChainFinder version 1.0.1 [1]. Following the program's instruction, two NGS data generated in this study, segmented copy number and somatic SV data were combined to estimate intra-chromosomal and inter-chromosomal fusions between SVs in a chain. To generate the dataset of high confidence somatic SVs, tumor and blood alignment data were called using MetaSV version 0.5.3 [6] that employed overlapped results of somatic calls from split-read, read-pair and read-depth approaches of the five NGS callers mentioned above. Only SVs > 50 bp detected by at least two tools were reported and were further filtered out if both breakpoints were within low complexity regions. The remaining was visually inspected using the IGV software and removed any suspect SVs, including *i*) those supported by one or more reads in the matched blood; and *ii*) those with supporting reads ambiguously aligned with Hg19 using BLAT search [73]. SV breakpoints were corrected based on their 95% confidence intervals calculated using a general probabilistic framework in Lumpy [22].

### Next generation mapping

Using the IrysPrep™ NLRS assay (Bionano Genomics Inc.), 300 ng/μL of HMW blood DNA and HMW tumor DNA underwent single-strand nicking with 7U and 10U of Nt.BspQ1 nickase (New England BioLabs), respectively. This was followed by labelling with a fluorophore-labelled nucleotide and the repair of labelled nicks to restore strand integrity. Fluorescently labelled DNA was stained with YOYO-1 to ensure motif location against a visual backbone using the Irys instrument control software (Bionano Genomics Inc.).

Raw molecules were filtered based on molecule length > 150 Kb and a signal-to-noise ratio (SNR) between label and background fluorescence > 2.75 for statically calculated SNR or > 3.00 for dynamically calculated SNR. Filtered molecules were normalised for stretch differences between different runs and scans of the same sample.

*De novo* assemblies of single molecules into consensus genome maps and SV detection relative to Hg19 were performed, independently for the tumor and blood samples, using Bionano Irys Pipeline version 4125 (released 19 Sept 2015), described in the Bionano Genomics Inc. website as well as Pendleton et al [9]. All SV events spanning known N-filled gaps in Hg19 were excluded.

### Detecting somatic SVs > 1 Kb using NGM

Somatic SVs were defined as those present in the tumor sample with zero base overlap with any SVs found in the blood sample. SV events were annotated as affecting genes, if the estimated breakpoints spanned any of 30,504 known genes in the UCSC Known Canonical Genes table. A gene fusion was predicted from a deletion event if the two breakpoints observed were within different annotated genes.

### Verification of NGM-derived SVs using NGS

Somatic SVs identified by NGM were verified using the following NGS evidence: *i*) overlaps with SVs > 1 Kb identified using MetaSV; *ii*) those with at least one NGS-supporting read(s) present within the NGM candidate region as manually inspected using IGV [67], and *iii*) those observed in *de novo* genome assembly of the tumor. MetaSV analyses, described in ‘detecting somatic chained rearrangements and deletions using NGS’, were performed jointly and separately for the tumor and blood samples, and only SVs > 50 bp detected by at least two tools per sample were selected for a direct comparison with each of the tumor and blood NGM datasets, respectively. SV overlaps between MetaSV and NGM calls were performed using BEDTools [74], with at least 1-bp overlaps and identical SV types.

*De novo* genome assemblies of the tumor (a single and two sequencing lanes) and matched blood (a single lane) were independently performed using ABySS v1.9.0 [75] (results in Supplementary Table 12). Default paired-end sequencing parameters for human genome assemblies (abyss-pe) were used with a stringent k-mer of 96 (k = 96) and five pairs minimally required for construction of a contig (*n* = 5). Scaffolds larger than 500 bp were retained using cutadapt v1.9 and subsequently aligned to Hg19 using bwa-mem v0.7.12 with a secondary alignment option enabled. Genomic regions containing NGM-derived SVs were manually inspected using the IGV and verified if deleted or inserted sequences were observed within the alignment between NGS scaffold and reference genome [76].

## SUPPLEMENTARY MATERIALS FIGURES AND TABLES












