# Supplementary material for: Next generation mapping reveals novel large genomic rearrangements in prostate cancer
Source: Oncotarget. 2017 Mar 1;8(14):23588–602. doi: 10.18632/oncotarget.15802 (PMC5410329; doi:10.18632/oncotarget.15802)
Supplement: Supplementary file 1 [file oncotarget-08-23588-s001.pdf]

Next generation mapping reveals novel large genomic rearrangements in prostate cancer

Supplementary Materials

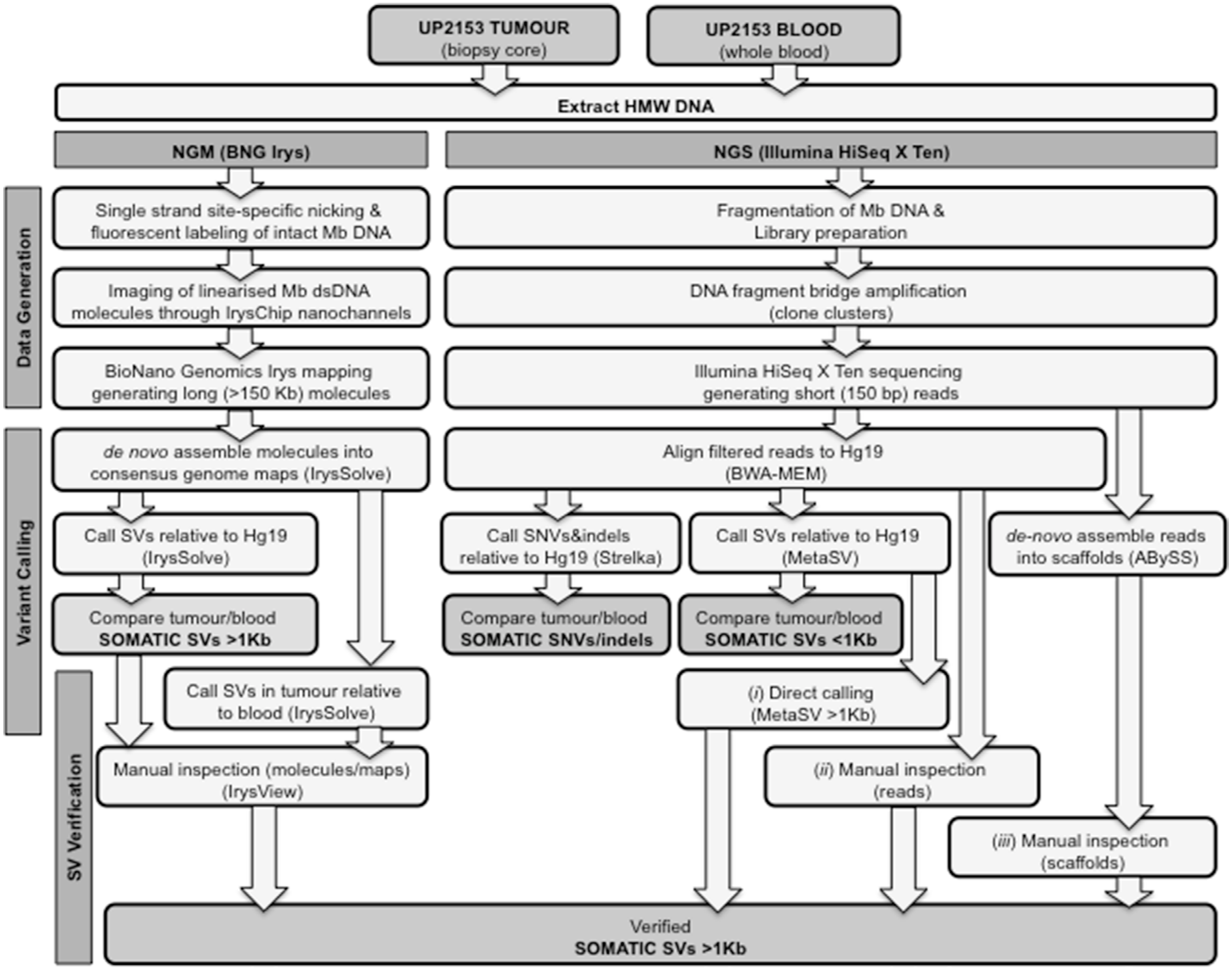

Supplementary Figure 1: UP2153 analysis workflow. Schematic representation of the workflow used for the identification of somatic variations in a matched tumor-normal pair, using next-generation mapping (NGM) and sequencing (NGS). HMW DNA from the prostate tissue and matched whole blood samples were prepared once and used as starting material for both technologies. SVs larger than 1 Kb identified using NGM were verified via several NGS approaches: *i*) unguided somatic SV identification using a collection of five SV-calling tools, *ii*) visual inspection of sequencing reads, and *iii*) manual inspection of *de novo* assembled scaffolds.

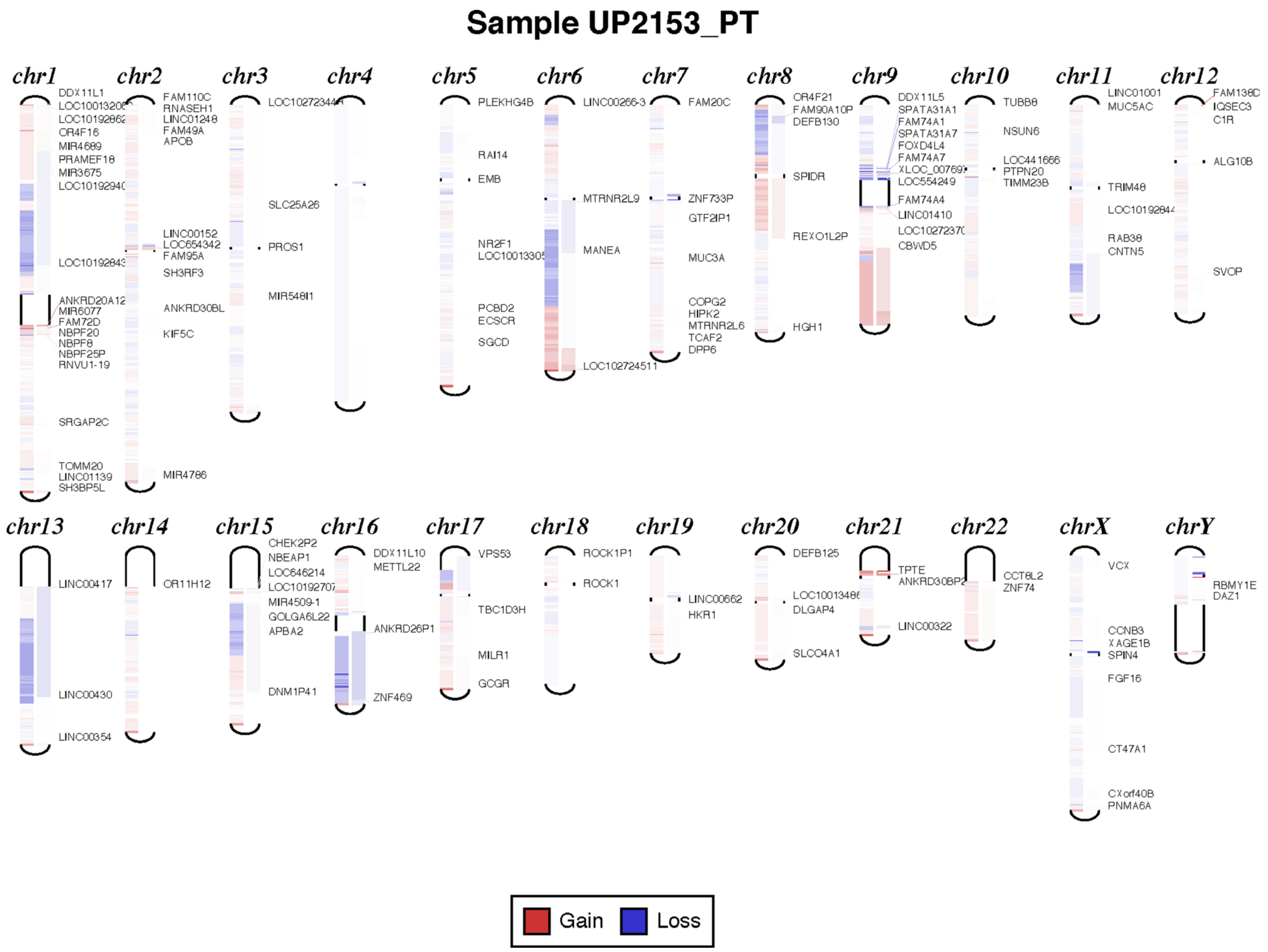

Supplementary Figure 2: Copy number gains (red) and losses (blue) in the prostate tumor of UP2153 represented per chromosome. The diagram shows somatic copy number alterations based on raw copy number (right column) and segmentation data (left column). Color gradients indicate $\log_2$ changes in the ratio of copy numbers between tumor and blood. Genes affected by copy number alterations are labelled using the $\log_2$ ratio cutoff at 0.5.

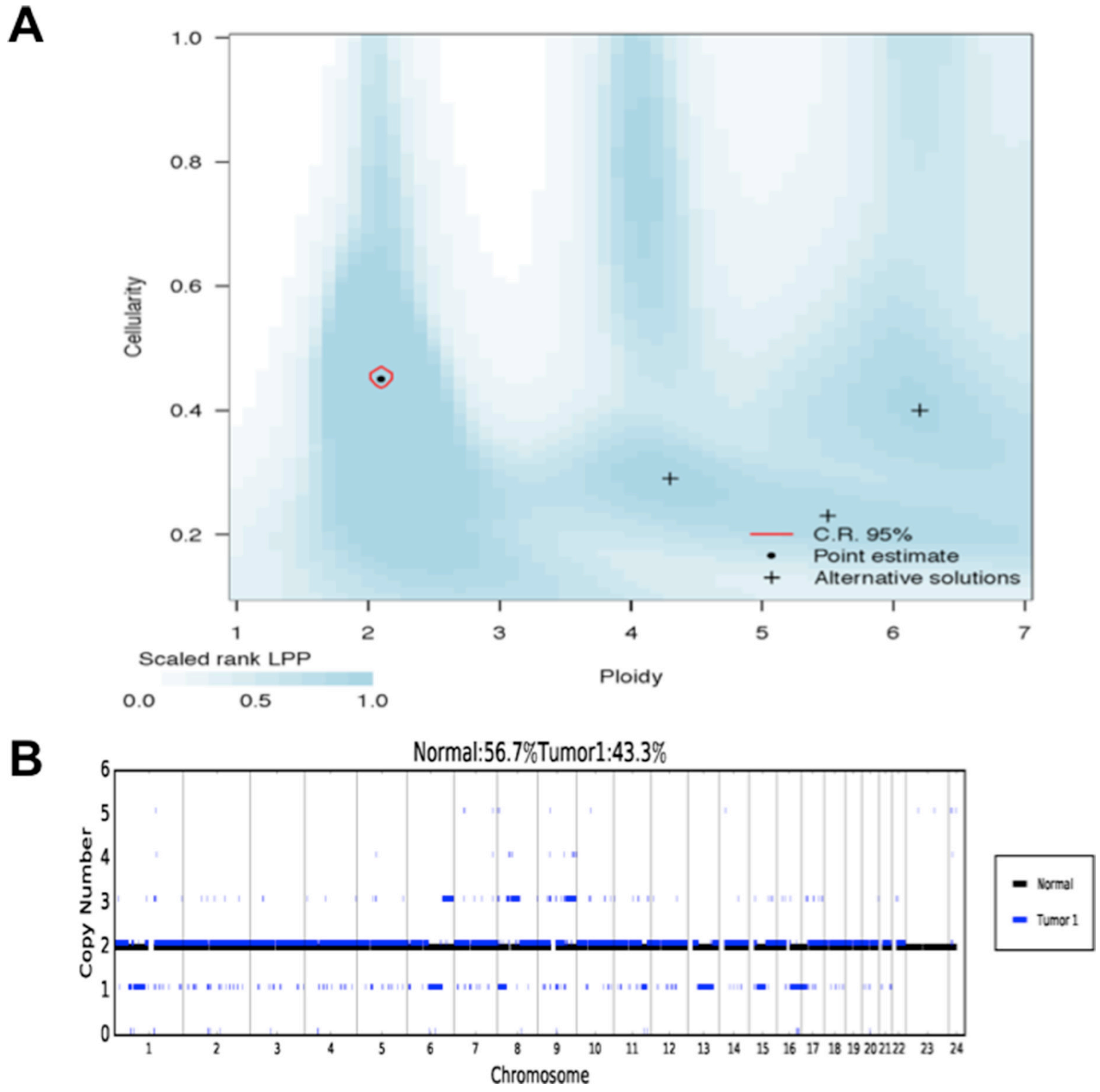

Supplementary Figure 3: Estimates of (**A**) tumor purity and ploidy and (**B**) number of subclones within the UP2153 tumor. Purity and ploidy levels were estimated to be 45% and 2.1 respectively using Sequenza, while only a single subclone (Tumor 1) with 43.3% purity was found in the UP2153 tumor using THetA2.

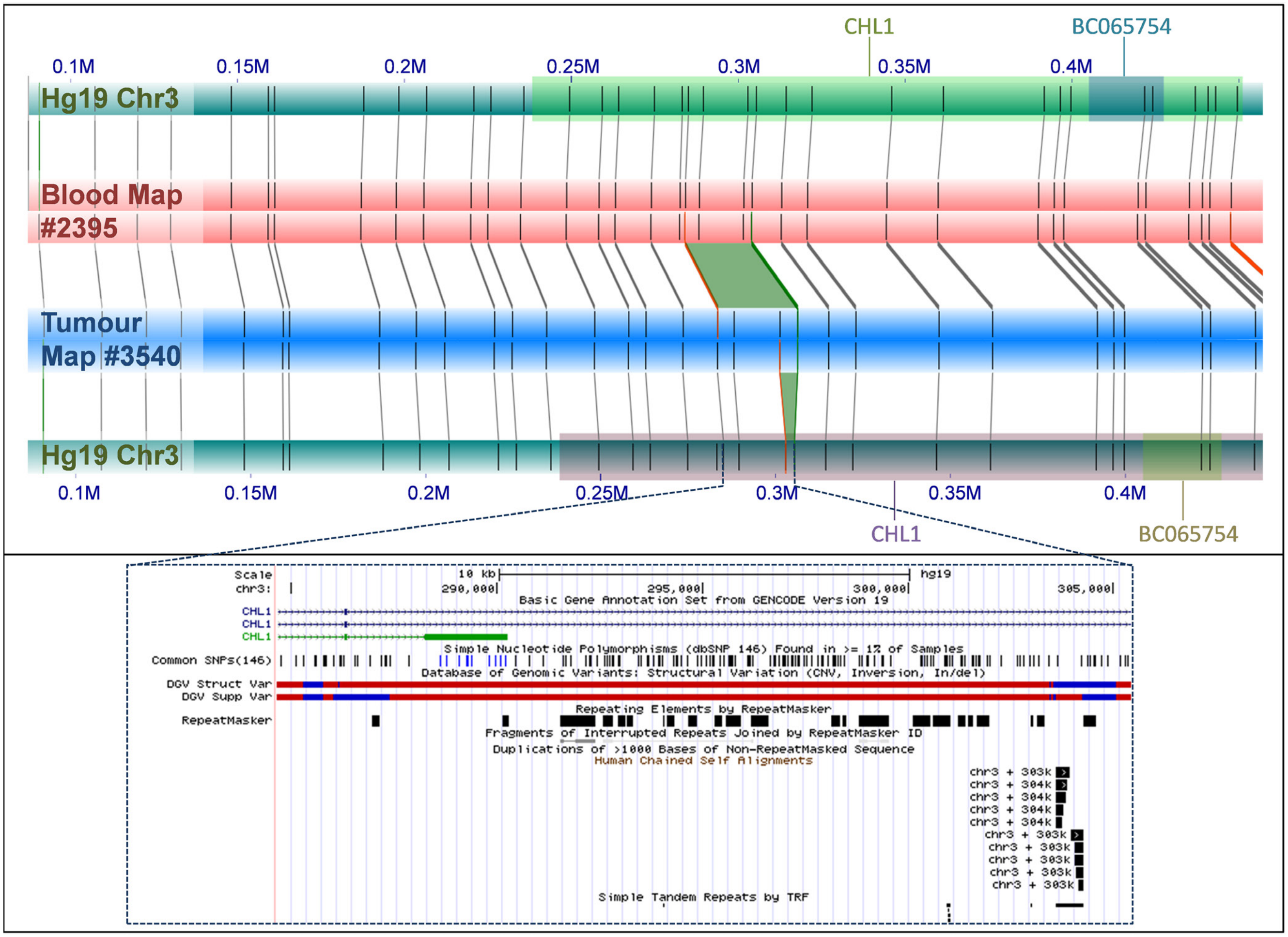

Supplementary Figure 4: A 4 Kb somatic insertion within the *CHL1* gene at chromosome 3: 284.6 - 305.4 Kb identified in the prostate tumor of UP2153 using NGM. While the tumor genome map (blue horizontal bars) showed a 2.5 kb insertion (green trapezoid) at Chr3: 302.9–305.4 Kb (coordinates are indicated with dark blue font) relative to the *in silico* genome reference map Hg19 (aqua horizontal bar), direct comparison of the tumor to matched blood genome maps (red horizontal bars) showed a larger 4 Kb somatic insertion (green trapezoid) within *CHL1* gene region (green rectangle with gene symbol overlaid on the reference genome map). Irys enzymatic labels (nick sites) are shown as vertical grey bars overlaid on the genome map tracks, with alignment of labels shown as grey connecting lines. The region is defined by a tandem repeat interval as observed within the UCSC Genome Browser (inset).

Supplementary Table 1: Illumina HiSeq X Ten data summary for patient UP2153 blood and tumour

	UP2153 Blood	UP2153 Tumour
**Mapping characteristics**		
hg19 reference size in bp	3,095,677,412	3,095,677,412
Number of reads	796,499,489	1,507,407,917
Mapped reads	780,670,779 (98.01%)	1,482,262,140 (98.33%)
Unmapped reads	15,828,710 (1.99%)	25,145,777 (1.67%)
Mean read length in bp (range)	147.6 (30–150)	147.06 (30–150)
Duplicated reads	125,017,618 (15.7%)	340,191,782 (22.57%)
Mean Mapping Quality	54.2	54.34
**ACGT Content**		
Number of A bases	33,277,989,258 (29.47%)	62,422,436,659 (29.21%)
Number of C bases	23,099,793,904 (20.45%)	44,262,224,381 (20.71%)
Number of T bases	33,386,200,946 (29.56%)	62,617,474,753 (29.3%)
Number of G bases	23,175,009,100 (20.52%)	44,406,428,338 (20.78%)
Number of N bases	0 (0%)	0 (0%)
GC Percentage	40.97%	41.49%
**Coverage**		
Mean depth of coverage	36.5X	69.06X
Coverage-per-chromosome range	18.30X (chrY)–44.389(chr10)	30.61X (chrY)–83.08 (chr10)

Note, Illumina reads were filtered based on their length, quality scores, ambiguous bases and contaminant sequences. Sequencing reads that were aligned to the human reference genome hg19.

Supplementary Table 2: UP2153 germline alleles at 100 SNVs linked to prostate cancer risk.
See Supplementary _Table_2

Supplementary Table 3: UP2153 germline SNVs/indels identified within *BRCA1* and *BRCA2* genes.
See Supplementary _Table_3

Supplementary Table 4: NGS-derived small somatic variants (SNVs and indels < 50 bp) detected in UP2153. See Supplementary _Table_4

Supplementary Table 5: NGS-derived somatic SVs (> 50 bp) in UP2153 using five tools and post-call filtering

	NGS SV detection tools					NGS SV post-call Filtering[1]			
	**Manta**	**BreakDancer**	**Lumpy**	**CNVnator**	**Pindel**	**MetaSV (≥ 2 tools)**	**LCR exclusion**	**IGV SVs < 1 Kb**	**IGV SVs > 1 Kb**
**DEL**	35	1466	688	1529	6681	218	208	45	26
**DUP**	12	0	112	717	1118	39	35	4	7
**INS**	0	0	0	0	1574	0	0	0	0
**INV**	27	429	24	0	93	8	8	0	1
Total	74	1895	824	2246	9466	265	251	49	34

Abbreviations: bp, base pairs; Kb, kilobases; SNVs, single nucleotide variants; DEL, deletion; INS, insertion; DUP, duplication; INV, inversion;
LCR, low complexity regions; IGV, Integrative Genomics Viewer (manual inspection)
[1]After filtering we identify 83 somatic SVs between 75 bp and 3 Mb, 49 are under 1 Kb and 34 are larger than 1 Kb.

Supplementary Table 6: NGS-derived intermediate-sized (< 1 Kb) somatic structural variations (SVs) detected in UP2153 from five SV-calling tooled MetaSV analysis

chr	start	end	ref	alt	gene	type	Length (bp)[1]	transcript	Variant impact	Impact severity
chr1	10444027	10444289	T	<DEL>	KIF1B	sv	–261	ENST00000377086	Downstream gene	LOW
chr1	39372962	39373291	G	<DEL>	RHBDL2	sv	–328	ENST00000289248	Intronic	LOW
chr1	47793273	47793697	C	<DEL>	None	sv	–423	-	Intergenic	LOW
chr1	238767492	238767730	G	<DEL>	None	sv	–237	-	Intergenic	LOW
chr10	701773	702289	T	<DUP>	DIP2C	sv	78	ENST00000280886	Intronic	LOW
chr10	15202557	15203019	G	<DEL>	NMT2	sv	–461	ENST00000378150	Intronic	LOW
chr11	134629277	134630047	C	<DUP>	RP11-469N6.1	sv	564	ENST00000513405	Intronic	LOW
chr12	2644149	2644756	G	<DEL>	CACNA1C	sv	–317	ENST00000480911	Intronic	LOW
chr12	118762836	118763043	C	<DUP>	TAOK3	sv	206	ENST00000539872	Intronic	LOW
**chr12**	**122240893**	**122241118**	**A**	**<DEL>**	**AC084018.1**	**sv**	**–224**	**ENST00000538335**	**Splice donor**	**HIGH**
chr15	82338398	82338709	T	<DEL>	MEX3B	sv	–310	ENST00000558133	5 prime UTR	LOW
chr17	73744180	73744716	C	<DEL>	ITGB4	sv	–309	ENST00000582629	Intronic	LOW
chr18	19563898	19563996	G	<DEL>	RP11-595B24.1	sv	–97	ENST00000584898	Intronic	LOW
chr18	71958579	71958832	T	<DEL>	CYB5A	sv	–252	ENST00000397914	Intronic	LOW
chr19	1111126	1111462	C	<DEL>	SBNO2	sv	–335	ENST00000438103	Intronic	LOW
**chr19**	**12113922**	**12114229**	**A**	**<DEL>**	**CTD-2006C1.2**	**sv**	**–306**	**ENST00000489336**	**Splice acceptor**	**HIGH**
chr19	12909754	12910081	G	<DEL>	PRDX2	sv	–296	ENST00000334482	Intronic	LOW
chr19	49126235	49126625	T	<DEL>	SPHK2	sv	–389	ENST00000426514	Intronic	LOW
chr19	58589595	58590074	G	<DEL>	ZNF135	sv	–478	ENST00000515535	Intronic	LOW
**chr2**	**26863227**	**26863484**	**C**	**<DEL>**	**CIB4**	**sv**	**–256**	**ENST00000288861**	**Exon loss**	**HIGH**
chr20	42596910	42597294	T	<DUP>	TOX2	sv	383	ENST00000442881	Intronic	LOW
**chr20**	**44035196**	**44035458**	**C**	**<DEL>**	**DBNDD2**	**sv**	**–261**	**ENST00000372717**	**Splice donor**	**HIGH**
chr21	44604625	44605096	A	<DEL>	None	sv	–470	-	Intergenic	LOW
chr21	47610261	47610444	T	<DEL>	LSS	sv	–182	ENST00000457828	3 prime UTR	LOW
**chr22**	**46449279**	**46449743**	**A**	**<DEL>**	**C22orf26**	**sv**	**–463**	**ENST00000333761**	**Splice donor**	**HIGH**
chr3	13432850	13433240	T	<DEL>	NUP210	sv	–389	ENST00000254508	Intronic	LOW
chr3	47215558	47215813	A	<DEL>	RP11-447D11.2	sv	–254	ENST00000429315	Intronic	LOW
**chr3**	**50303838**	**50304523**	**A**	**<DEL>**	**U73167.7**	**sv**	**–684**	**ENST00000421735**	**Splice acceptor**	**HIGH**
chr3	84155843	84156226	G	<DEL>	None	sv	–382	-	Intergenic	LOW
chr3	188741993	188742259	G	<DEL>	TPRG1	sv	–265	ENST00000433971	Intronic	LOW
chr5	72335455	72335792	T	<DEL>	FCHO2	sv	–336	ENST00000512348	Intronic	LOW
chr5	170797189	170797502	T	<DEL>	SNORA70	sv	–312	ENST00000384182	Downstream gene	LOW
chr5	177629598	177629811	T	<DEL>	HNRNPAB	sv	–212	ENST00000358344	Upstream gene	LOW
chr6	49957411	49957765	G	<DEL>	None	sv	–353	-	Intergenic	LOW
**chr6**	**137113135**	**137113469**	**A**	**<DEL>**	**MAP3K5**	**sv**	**–333**	**ENST00000359015**	**frameshift**	**HIGH**
chr6	166966360	166966565	T	<DEL>	RPS6KA2	sv	–204	ENST00000507350	Intronic	LOW
chr7	76054599	76054931	G	<DEL>	ZP3	sv	–331	ENST00000336517	Splice region	LOW
chr7	80653394	80653730	T	<DEL>	None	sv	–335	-	Intergenic	LOW
chr7	140046499	140046861	A	<DEL>	SLC37A3	sv	–361	ENST00000491357	Intronic	LOW
chr8	37968872	37969170	C	<DEL>	ASH2L	sv	–297	ENST00000545394	Intronic	LOW
chr8	64884874	64885023	T	<DEL>	RP11-32K4.1	sv	–148	ENST00000521958	Intronic	LOW
chr8	99065307	99065595	A	<DEL>	U6	sv	–287	ENST00000516946	Downstream gene	LOW
chr8	101272851	101272927	T	<DEL>	RNF19A	sv	–75	ENST00000341084	Intronic	LOW
chr8	145622164	145622360	A	<DEL>	CPSF1	sv	–195	ENST00000349769	Intronic	LOW
chr9	73219598	73219912	A	<DEL>	TRPM3	sv	–313	ENST00000377111	Intronic	LOW
chr9	96681504	96682421	A	<DEL>	None	sv	–787	-	Intergenic	LOW
chr9	123840186	123840649	A	<DEL>	CNTRL	sv	–462	ENST00000373855	Intronic	LOW
chrX	18431343	18431743	T	<DEL>	None	sv	–399	-	Intergenic	LOW
chrX	84663892	84664301	A	<DEL>	None	sv	–408	-	Intergenic	LOW

Abbreviations: Chr, Chromosome; Ref, Reference Hg19; SV, structural variant; DEL, deletion; DUP, duplication.

[1] Size of SVs detected ranged from 75 bp to 787 bp in length.

[2] Seven deletions (BOLD) were predicted to have high functional impact, including two splice acceptor variants, three splice donor variants, a frameshift variant and an exon loss variant.

Supplementary Table 7: NGS-derived large-sized (>1Kb) somatic structural variations (SVs) detected in UP2153 from five SV-calling tooled MetaSV analysis

Chr#	start	end	ref	alt	gene	SV length (bp)	transcript	Variant impact[1]	Impact severity[2]	Cancer associated (MalaCards)[3]
chr8	3124530	32550122	A	<DEL>	None	–29425612	-	Chromosomal deletion	LOW	-
chr11	102431957	121062399	C	<DEL>	None	–18630462	-	Chromosomal deletion	LOW	-
chr13	85131523	90495923	A	<DEL>	None	–5364399	-	Chromosomal deletion	LOW	-
chr6	8724385	13486055	A	<DEL>	None	–4761668	-	Chromosomal deletion	LOW	-
chr5	54648650	58145401	C	<DEL>	None	–3496685	-	Chromosomal deletion	LOW	-
chr6	20487815	23901338	T	<DEL>	None	–3413480	-	Chromosomal deletion	LOW	-
chr16	78144386	81061700	A	<DEL>	None	–2900940	-	Chromosomal deletion	LOW	-
chr1	234617142	236182971	G	<DEL>	NID1	–1565842	ENST00000366595	**Exon loss**	**HIGH**	Melanoma
chr4	146144154	146969903	A	<DEL>	SMAD1	–825829	ENST00000514778	**Exon loss**	**HIGH**	**Prostate Cancer**
chr11	130626054	131127355	G	<DEL>	SNX19	–501077	ENST00000426933	**Exon loss**	**HIGH**	-
chr4	151414835	151724869	G	<DEL>	LRBA	–309960	ENST00000509835	**Exon loss**	**HIGH**	-
chr5	32139643	32360524	C	<DEL>	ZFR	–220839	ENST00000265069	**Exon loss**	**HIGH**	Breast Cancer
chr9	94431990	94641458	A	<DEL>	ROR2	–209466	ENST00000375715	**Exon loss**	**HIGH**	Testicular Seminoma
chr5	167726412	167856907	G	<DEL>	WWC1	–130450	ENST00000393895	**Exon loss**	**HIGH**	Colorectal Adenoma
chr14	68966830	69025813	C	<DEL>	RAD51B	–58982	ENST00000556251	**Exon loss**	**HIGH**	**Prostate Cancer**
chr6	103332815	103365484	T	<DEL>	None	–32668	-	intergenic	LOW	-
chrX	102778833	102808923	T	<DEL>	RAB40A	–30056	ENST00000304236	Upstream gene	LOW	-
chr8	69851207	69880021	C	<DEL>	RP11-600K15.1	–28842	ENST00000518540	**Splice acceptor**	**HIGH**	-
chr20	15741796	15768150	G	<DEL>	MACROD2	–26353	ENST00000402914	Intronic	LOW	-
chr20	15069634	15092461	C	<DEL>	MACROD2	–22826	ENST00000217246	Intronic	LOW	-
chr6	57224594	57241911	G	<DEL>	PRIM2	–17333	ENST00000389488	Intronic	LOW	-
chr1	241771837	241789229	G	<DEL>	OPN3	–17313	ENST00000331838	**Exon loss**	**HIGH**	-
chr18	25206510	25222055	A	<DEL>	None	–15544	-	Intergenic	LOW	-
chr3	116680339	116691215	T	<DEL>	LSAMP	–10875	ENST00000474851	Intronic	LOW	-
chr1	195220898	195227236	T	<DEL>	None	–6337	-	Intergenic	LOW	-
chr11	70977350	70979230	G	<DEL>	None	–1879	-	Intergenic	LOW	-
chr19	57163590	57167140	T	<DUP>	None	3549	-	Intergenic	LOW	-
chr5	58338681	58354016	G	<DUP>	PDE4D	15334	ENST00000505453	Intronic	LOW	-
chr1	243894954	243956043	C	<DUP>	AKT3	61088	ENST00000552631	Intronic	LOW	-
chr2	202754668	203052192	G	<DUP>	CDK15	297523	ENST00000260967	Intronic	LOW	-
chr6	123746813	124249990	G	<DUP>	TRDN	503176	ENST00000334268	Intronic	LOW	-
chr11	124784700	125309242	T	<DUP>	HEPN1	507950	ENST00000408930	Upstream gene	LOW	-
chr13	85030989	90784755	T	<DUP>	None	5753765	-	Intergenic	LOW	-
chr6	87225397	87225398	C	<INV>	None	16477152	-	Intergenic	LOW	-

Abbreviations: chr, chromosome; ref, Reference Hg19; alt, alteration; DEL, deletion; DUP duplication; INV, inversion; SV, structural variation
Of 34 SVs >1 Kb detected, nine resulting in exon loss and one impacting the splice acceptor (Bold)[1], were predicted to have high functional impact (Bold)[2], with two impacting genes previously
reported to be impacted in prostate cancer (Bold)[3].

Supplementary Table 8.: Annotation of carcinogenic potential for 23 functionally damaging somatic SNVs identified in UP2153. See Supplementary_Table_8

Supplementary Table 9: ChainFinder results for UP2153 prostate tumor identifying a single coordinate chain event involving a deletion and two translocations on chromosomes 6 and 16.

SV id	First chr	str1[1]	pos1	Second chr	str2[1]	pos2	site1	site2	ChainFinder result[2]
1	6	0	8724386	6	1	13486054	intron 4 NR_038980 gene	intron 1 GFOD1 gene	Yes - chain 1
2	6	0	6644823	16	0	72230558	intron 3 LY86 gene	IGR 20kb away from PMFBP1 gene	Yes - chain 1
3	6	1	8724033	16	1	73211489	intron 4 NR_038980 gene	IGR 30kb away from C16orf47 gene	Yes - chain 1

Abbreviations: SV, structural variation; chr, chromosome; str, strand; pos, position.

[1] str1/2 means the strand direction of the first or second breakpoint (0 for forward, 1 for reverse)

2 Chain 1 has the following genes potentially deleted and/or rearranged: (i) Deletion interval (chr6:6644823-13486054): BMP6 C6orf105 C6orf114 C6orf52 CAGE1 DSP EDN1 EEF1E1 ELOVL2 ERVFRDE1 GCM2 GCNT2 GFOD1 HIVEP1 LOC221710 LY86 MAK MUTED NEDD9 PAK1IP1 PHACTR1 RIOK1 RREB1 SLC35B3 SNRNP48 SSR1 SYCP2L TBC1D7 TFAP2A TMEM14B TMEM14C TMEM170B TXNDC5; (ii) Deletion interval (chr16:72230558-73211489): ZFHX3; (iii) Breakpoint vicinity (chr6:6619823-6669823): LY86; (iv) Breakpoint vicinity (chr6:13461054-13511054): C6orf114 GFOD1; and (v) Breakpoint vicinity (chr16:72205558-72255558): PMFBP1

Supplementary Table 10: BioNano Irys NGM molecule and *de novo* assembled genome map statistics for patient UP2153

	UP2153 Blood	UP2153 Tumour
**Molecule Statistics^**		
Quantity (Mb)	269,668	234,103
#Molecules	1,062,593	1,155,817
Molecule N50 (Kb)	255.7	194.8
Avg. Label Density per 100 Kb	9.7	9.6
Breadth of coverage against Hg19 (%)	89.7	84.8
Effective Coverage against Hg19	56.3	28.7
**Assembled Genome Map Statistics**		
Total Genome Map Length (Mb)	2,886	2,702
#Genome Maps	2,943	6,128
Genome Map N50 (Mb)	1.335	0.519
Breadth of Coverage against Hg19 (%)	90.3	82.6
Effective molecule coverage	68.5	35.3

^Include only QC'd molecules used in *de novo* assembly

Note: Molecule Quantity is the estimated cumulative length of captured molecules. Molecule N50 is centre of mass of the length distribution of unassembled molecules, while genome map N50 is the centre of mass of the consensus genome map length distribution. Breadth of coverage is the fraction of the human reference genome, Hg19, covered (aligned), noting that the theoretical coverage is < 95% due to regions of the genome that do not have the Nt.BspQ1 enzymatic motif. Effective coverage is the average number of molecules that uniquely aligned to any site of a genome map.

Supplementary Table 11: NGM-derived somatic structural variations (SVs) in UP2153. See Supplementary _Table_11

Supplementary Table 12: *De novo* assemblies and statistics of their contigs and scaffolds observed in UP2153

Assembly statistics	Contig			Scaffold		
	Tumour (single lane)	Tumour (two lanes)	Blood (single lane)	Tumour (single lane)	Tumour (two lanes)	Blood (single lane)
**Count**	1,202,080	1,257,291	1,183,391	1,076,873	1,170,066	1,051,837
**n:500**	279,293	203,818	226,853	206,820	154,810	148,616
**L50**	30,168	21,081	23,954	19,337	14,622	12,362
**Min**	500	500	500	500	500	500
**N50**	26,860	40,017	34,473	41,939	56,903	67,039
**Max**	361,103	389,567	364,949	412,374	599,723	693,695
**Sum**	2,910,000,000	2,960,000,000	2,960,000,000	2,920,000,000	2,960,000,000	2,970,000,000

n:500 is the number of contigs/scaffolds larger than 500 bp. L50 is the number of contigs/scaffolds longer than the N50 length. N50 is the length of contig or scaffolds at 50% of the ordered genome assembly. Total is the total number of bases (A/C/G/T) found in all contigs/scaffolds > 500 bp.
