# Supplementary material for: Next generation mapping reveals novel large genomic rearrangements in prostate cancer
Source: Oncotarget. 2017 Mar 1;8(14):23588–602. doi: 10.18632/oncotarget.15802 (PMC5410329; doi:10.18632/oncotarget.15802)
Supplement: Supplementary file 2 [file oncotarget-08-23588-s002.docx]

| **Table S2. UP2153 germline alleles at 100 SNVs linked to prostate cancer risk** | | | | | | | | | |
| --- | --- | --- | --- | --- | --- | --- | --- | --- | --- |
| **SNV#** | **Chr#** | **position** | **dbsnp id** | **gene** | **status** | **Variant p-value^1^** | **hg19** | **UP2153 allele^2^** | **GWAS risk allele^2^** |
| 1 | chr1 | 10556097 | rs636291 | PEX14 | Germline variant | 6.82E-46 | G | **A/A** | **A** |
| 2 | chr1 | 150658287 | rs17599629 | GOLPH3L | Germline variant | 1.60E-19 | A | **A/G** | **G** |
| 3 | chr1 | 154834183 | rs1218582 | KCNN3 | Germline variant | 1.86E-13 | G | **G/A** | **G** |
| 4 | chr1 | 204518842 | rs4245739 | MDM4, PIK3C2B | Germline variant | 4.39E-56 | C | A/A | C |
| 5 | chr1 | 205757824 | rs1775148 | SLC41A1 | Germline variant | 4.32E-47 | C | T/T | C |
| 6 | chr10 | 51549496 | rs10993994 | MSMB | Germline variant | 6.25E-18 | T | **T/C** | **T** |
| 7 | chr10 | 104414221 | rs3850699 | TRIM8 | Germline variant | 1.55E-15 | A | **A/G** | **G** |
| 8 | chr11 | 2233574 | rs7127900 | Unknown | Germline variant | 2.71E-45 | A | G/G | A |
| 9 | chr11 | 58915110 | rs1938781 | FAM111A | Germline variant | 4.39E-56 | A | **G/G** | **G** |
| 10 | chr11 | 68994497 | rs7931342 | Unknown | Germline variant | 1.56E-13 | T | **T/G** | **T** |
| 11 | chr11 | 102401661 | rs11568818 | MMP7 | Germline variant | 1.04E-16 | T | **T/C** | **C** |
| 12 | chr12 | 49676010 | rs10875943 | TUBA1C/PRPH | Germline variant | 9.32E-20 | T | **T/C** | **C** |
| 13 | chr12 | 114685571 | rs1270884 | TBX5 | Germline variant | 9.75E-16 | A | **A/G** | **A** |
| 14 | chr13 | 73728139 | rs9600079 | Unknown | Germline variant | 1 | G | **G/T** | **T** |
| 15 | chr14 | 53372330 | rs8008270 | FERMT2 | Germline variant | 6.91E-52 | T | C/C | T |
| 16 | chr14 | 61122526 | rs7153648 | SIX1 | Germline variant | 2.07E-17 | C | **C/G** | **C** |
| 17 | chr14 | 69126744 | rs7141529 | RAD51L1 | Germline variant | 1.24E-15 | T | **T/C** | **C** |
| 18 | chr14 | 71092256 | rs8014671 | TTC9 | Germline variant | 1.10E-21 | G | **G/A** | **G** |
| 19 | chr17 | 36098040 | rs4430796 | HNF1B | Germline variant | 5.01E-12 | G | **G/A** | **A** |
| 20 | chr19 | 38735613 | rs8102476 | Unknown | Germline variant | 1.58E-15 | C | **C/T** | **C** |
| 21 | chr19 | 41985587 | rs11672691 | Unknown | Germline variant | 6.82E-46 | A | G/G | A |
| 22 | chr19 | 51364623 | rs2735839 | KLK2/KLK3 | Germline variant | 2.71E-45 | A | G/G | A |
| 23 | chr2 | 20888265 | rs13385191 | C2orf43 | Germline variant | 4.41E-17 | A | **A/G** | **G** |
| 24 | chr2 | 43553949 | rs1465618 | THADA | Germline variant | 4.35E-50 | T | C/C | T |
| 25 | chr2 | 85794297 | rs10187424 | GGCX/VAMP8 | Germline variant | 6.76E-43 | T | **C/C** | **C** |
| 26 | chr2 | 242382864 | rs3771570 | FARP2 | Germline variant | 2.51E-15 | C | **C/T** | **T** |
| 27 | chr20 | 62362563 | rs6062509 | ZGPAT | Germline variant | 7.13E-15 | G | **G/T** | **G** |
| 28 | chr21 | 42901421 | rs1041449 | TMPRSS2 | Germline variant | 1.08E-44 | A | **G/G** | **G** |
| 29 | chr22 | 43500212 | rs5759167 | BIL/TTLL1 | Germline variant | 1.73E-49 | G | **T/T** | **T** |
| 30 | chr3 | 87467332 | rs2055109 | Unknown | Germline variant | 1.07E-41 | C | T/T | C |
| 31 | chr3 | 113275624 | rs7611694 | SIDT1 | Germline variant | 4.32E-47 | A | **C/C** | **C** |
| 32 | chr3 | 128038373 | rs10934853 | EEFSEC | Germline variant | 2.22E-19 | C | **C/A** | **A** |
| 33 | chr3 | 170130102 | rs10936632 | CLDN11/SKIL | Germline variant | 2.67E-12 | C | **C/A** | **C** |
| 34 | chr4 | 74349158 | rs1894292 | AFM, RASSF6 | Germline variant | 1.11E-15 | G | **G/A** | **A** |
| 35 | chr4 | 95514609 | rs12500426 | PDLIM5 | Germline variant | 4.18E-38 | A | C/C | A |
| 36 | chr4 | 106061534 | rs7679673 | TET2 | Germline variant | 2.76E-54 | C | **A/A** | **A** |
| 37 | chr5 | 1280028 | rs2242652 | TERT | Germline variant | 4.51E-15 | G | **G/A** | **A** |
| 38 | chr5 | 44365545 | rs2121875 | FGF10 | Germline variant | 3.30E-15 | C | **C/A** | **C** |
| 39 | chr5 | 172939426 | rs6869841 | FAM44B (BOD1) | Germline variant | 4.38E-17 | C | **C/T** | **T** |
| 40 | chr6 | 30073776 | rs115457135 | TRIM31 | Germline variant | 9.76E-15 | G | **G/A** | **A** |
| 41 | chr6 | 32192331 | rs3096702 | NOTCH4 | Germline variant | 1.82E-14 | A | **A/G** | **A** |
| 42 | chr6 | 41536427 | rs1983891 | FOXP4 | Germline variant | 3.07E-16 | C | **C/T** | **T** |
| 43 | chr6 | 153441079 | rs1933488 | RSG17 | Germline variant | 7.91E-26 | A | **A/G** | **G** |
| 44 | chr7 | 20994491 | rs12155172 | SP8 | Germline variant | 2.75E-51 | A | G/G | A |
| 45 | chr7 | 47437244 | rs56232506 | TNS3 | Germline variant | 6.94E-55 | G | **A/A** | **A** |
| 46 | chr8 | 23438975 | rs2928679 | SLC25A37 | Germline variant | 1.49E-11 | A | **A/G** | **A** |
| 47 | chr8 | 127924659 | rs12543663 | Unknown | Germline variant | 6.94E-55 | C | A/A | C |
| 48 | chr8 | 128011937 | rs10086908 | Unknown | Germline variant | 1.60E-17 | T | **T/C** | **C** |
| 49 | chr8 | 128335673 | rs620861 | Unknown | Germline variant | 2.30E-14 | G | **G/A** | **A** |
| 50 | chr8 | 128485038 | rs1447295 | Unknown | Germline variant | 6.82E-46 | A | C/C | A |
| 51 | chr9 | 110156300 | rs817826 | RAD23B–KLF4 | Germline variant | 1.05E-22 | C | **C/T** | **C** |
| 52 | chrX | 9814135 | rs2405942 | SHROOM2 | Germline variant | 3.67E-23 | G | A/A | G |
| 53 | chrX | 51241672 | rs5945619 | NUDT11 | Germline variant | 3.51E-20 | C | T/T | C |
| 54 | chrX | 52896949 | rs2807031 | XAGE3 | Germline variant | 9.89E-23 | C | T/T | C |
| 55 | chrX | 67021550 | rs5919432 | AR | Germline variant | 6.32E-31 | C | T/T | C |
| 56 | chr10 | 46082985 | rs76934034 | MARCH8 | No call | N/A | T | **T/T** | **T** |
| 57 | chr10 | 122844709 | rs2252004 | Unknown | No call | N/A | C | **C/C** | **C** |
| 58 | chr10 | 126696872 | rs4962416 | CTBP2 | No call | N/A | T | T/T | C |
| 59 | chr11 | 113807181 | rs11214775 | HTR3B | No call | N/A | G | **G/G** | **G** |
| 60 | chr12 | 48419618 | rs80130819 | RP1-228P16.4 | No call | N/A | A | **A/A** | **A** |
| 61 | chr12 | 53273904 | rs902774 | KRT8 | No call | N/A | G | G/G | A |
| 62 | chr16 | 71691329 | rs12051443 | PHLPP2 | No call | N/A | G | G/G | A |
| 63 | chr17 | 618965 | rs684232 | VPS53, FAM57A | No call | N/A | T | T/T | C |
| 64 | chr17 | 36074979 | rs11649743 | HNF1B | No call | N/A | G | **G/G** | **G** |
| 65 | chr17 | 47345186 | rs11650494 | SPOP, HOXB13 | No call | N/A | G | G/G | A |
| 66 | chr17 | 47436749 | rs7210100 | ZNF652 | No call | N/A | G | **G/G** | **G** |
| 67 | chr17 | 69108753 | rs1859962 | Unknown | No call | N/A | G | **G/G** | **G** |
| 68 | chr18 | 76773973 | rs7241993 | SALL3 | No call | N/A | C | C/C | T |
| 69 | chr19 | 54797848 | rs103294 | LILRA3 | No call | N/A | C | **C/C** | **C** |
| 70 | chr2 | 10117868 | rs11902236 | TAF1B:GRHL1 | No call | N/A | C | C/C | T |
| 71 | chr2 | 10710730 | rs9287719 | NOL10 | No call | N/A | C | **C/C** | **C** |
| 72 | chr2 | 63131731 | rs721048 | EHBP1 | No call | N/A | G | G/G | A |
| 73 | chr2 | 173311553 | rs12621278 | ITGA6 | No call | N/A | A | A/A | G |
| 74 | chr2 | 238443226 | rs2292884 | MLPH | No call | N/A | A | A/A | G |
| 75 | chr20 | 49527922 | rs12480328 | ADNP | No call | N/A | T | **T/T** | **T** |
| 76 | chr20 | 61015611 | rs2427345 | GATAS, CABLES2 | No call | N/A | C | C/C | T |
| 77 | chr22 | 19757892 | rs2238776 | TBX1 | No call | N/A | G | **G/G** | **G** |
| 78 | chr22 | 40471188 | rs58133635 | TNRC6B | No call | N/A | C | **C/C** | **C** |
| 79 | chr3 | 87110674 | rs2660753 | Unknown | No call | N/A | T | **T/T** | **T** |
| 80 | chr3 | 141102833 | rs6763931 | ZBTB38 | No call | N/A | G | G/G | A |
| 81 | chr4 | 73855253 | rs10009409 | COX18 | No call | N/A | C | C/C | T |
| 82 | chr4 | 95562877 | rs17021918 | PDLIM5 | No call | N/A | C | C/C | T |
| 83 | chr5 | 1895829 | rs12653946 | IRX4 | No call | N/A | C | C/C | T |
| 84 | chr6 | 11219030 | rs4713266 | NEDD9 | No call | N/A | C | **C/C** | **C** |
| 85 | chr6 | 31118511 | rs130067 | CCHCR1 | No call | N/A | T | T/T | G |
| 86 | chr6 | 32400939 | rs115306967 | HLA-DRB6 | No call | N/A | G | **G/G** | **G** |
| 87 | chr6 | 76495882 | rs9443189 | MYO6 | No call | N/A | A | A/A | G |
| 88 | chr6 | 109285189 | rs2273669 | ARMC2, SESN1 | No call | N/A | A | A/A | G |
| 89 | chr6 | 117210052 | rs339331 | RFX6 | No call | N/A | T | **T/T** | **T** |
| 90 | chr6 | 160833664 | rs9364554 | SLC22A3 | No call | N/A | C | C/C | T |
| 91 | chr7 | 27976563 | rs10486567 | JAZF1 | No call | N/A | G | **G/G** | **G** |
| 92 | chr7 | 97816327 | rs6465657 | LMTK2 | No call | N/A | C | **C/C** | **C** |
| 93 | chr8 | 23526463 | rs1512268 | NKX3.1 | No call | N/A | T | **T/T** | **T** |
| 94 | chr8 | 25892142 | rs11135910 | EBF2 | No call | N/A | C | C/C | T |
| 95 | chr8 | 128124916 | rs16901979 | Unknown | No call | N/A | C | C/C | A |
| 96 | chr8 | 128413305 | rs6983267 | Unknown | No call | N/A | G | **G/G** | **G** |
| 97 | chr9 | 22041998 | rs17694493 | CDKN2B-AS1 | No call | N/A | C | C/C | G |
| 98 | chr9 | 124427373 | rs1571801 | DAB21P | No call | N/A | G | G/G | T |
| 99 | chrX | 70139850 | rs6625711 | SLC7A | No call | N/A | A | **A/A** | **A** |
| 100 | chrX | 70407983 | rs4844289 | NLGN3-BCYRN1 | No call | N/A | A | A/A | G |

**Abbreviations:** SNV, single nucleotide variant; chr, chromosome; GWAS, genome-wide association study

^1^ *P*-values depict variant significance at each genome position.

^2^ Alleles in bold have been associated with increased prostate cancer risk.
