# Supplementary material for: Next generation mapping reveals novel large genomic rearrangements in prostate cancer
Source: Oncotarget. 2017 Mar 1;8(14):23588–602. doi: 10.18632/oncotarget.15802 (PMC5410329; doi:10.18632/oncotarget.15802)
Supplement: Supplementary file 3 [file oncotarget-08-23588-s003.docx]

**Table S3. UP2153 germline SNVs/indels identified within *BRCA1* and *BRCA2* genes.**

**Part A: *BRCA1/2* SNVs (n=236)**

| **chr** | **start** | **end** | **ref** | **alt** | **gene** | **exon** | **Codon change** | **aa change** | **transcript** | **In dbsnp** | **Cosmic ids** | **Variant impact** | **Impact severity** | **Polyphen prediction** | **Polyphen score** | **Sift prediction** | **Sift score** |
| --- | --- | --- | --- | --- | --- | --- | --- | --- | --- | --- | --- | --- | --- | --- | --- | --- | --- |
| chr13 | 32889967 | 32889968 | G | A | BRCA2 |  |  |  | ENST00000380152 | 1 | None | intronic | LOW |  | None |  | None |
| chr13 | 32890025 | 32890026 | C | T | BRCA2 |  |  |  | ENST00000544455 | 1 | None | intronic | LOW |  | None |  | None |
| chr13 | 32890226 | 32890227 | G | T | BRCA2 |  |  |  | ENST00000530893 | 1 | None | intronic | LOW |  | None |  | None |
| chr13 | 32890571 | 32890572 | G | A | BRCA2 | 2/27 | none | none | ENST00000380152 | 1 | None | 5 prime UTR | LOW |  | None |  | None |
| chr13 | 32891304 | 32891305 | T | C | BRCA2 |  |  |  | ENST00000380152 | 1 | None | intronic | LOW |  | None |  | None |
| chr13 | 32892216 | 32892217 | C | T | BRCA2 |  |  |  | ENST00000380152 | 1 | None | intronic | LOW |  | None |  | None |
| chr13 | 32892693 | 32892694 | G | T | BRCA2 |  |  |  | ENST00000530893 | 1 | None | intronic | LOW |  | None |  | None |
| chr13 | 32892734 | 32892735 | T | C | BRCA2 |  |  |  | ENST00000380152 | 1 | None | intronic | LOW |  | None |  | None |
| chr13 | 32892819 | 32892820 | T | C | BRCA2 |  |  |  | ENST00000530893 | 1 | None | intronic | LOW |  | None |  | None |
| chr13 | 32893790 | 32893791 | A | G | BRCA2 |  |  |  | ENST00000380152 | 1 | None | intronic | LOW |  | None |  | None |
| chr13 | 32894737 | 32894738 | G | A | BRCA2 |  |  |  | ENST00000530893 | 1 | None | intronic | LOW |  | None |  | None |
| chr13 | 32895376 | 32895377 | G | C | BRCA2 |  |  |  | ENST00000380152 | 1 | None | intronic | LOW |  | None |  | None |
| chr13 | 32895470 | 32895471 | T | A | BRCA2 |  |  |  | ENST00000530893 | 1 | None | intronic | LOW |  | None |  | None |
| chr13 | 32895673 | 32895674 | C | T | BRCA2 |  |  |  | ENST00000380152 | 1 | None | intronic | LOW |  | None |  | None |
| chr13 | 32896675 | 32896676 | G | A | BRCA2 |  |  |  | ENST00000544455 | 1 | None | intronic | LOW |  | None |  | None |
| chr13 | 32896721 | 32896722 | C | T | BRCA2 |  |  |  | ENST00000530893 | 1 | None | intronic | LOW |  | None |  | None |
| chr13 | 32898237 | 32898238 | G | A | BRCA2 |  |  |  | ENST00000380152 | 1 | None | intronic | LOW |  | None |  | None |
| chr13 | 32899387 | 32899388 | A | C | BRCA2 |  |  |  | ENST00000544455 | 1 | None | intronic | LOW |  | None |  | None |
| chr13 | 32899837 | 32899838 | C | T | BRCA2 |  |  |  | ENST00000380152 | 1 | None | intronic | LOW |  | None |  | None |
| chr13 | 32899877 | 32899878 | C | T | BRCA2 |  |  |  | ENST00000530893 | 1 | None | intronic | LOW |  | None |  | None |
| chr13 | 32900148 | 32900149 | T | C | BRCA2 |  |  |  | ENST00000380152 | 1 | None | intronic | LOW |  | None |  | None |
| chr13 | 32900932 | 32900933 | T | A | BRCA2 |  |  |  | ENST00000380152 | 1 | None | intronic | LOW |  | None |  | None |
| chr13 | 32901429 | 32901430 | A | C | BRCA2 |  |  |  | ENST00000380152 | 1 | None | intronic | LOW |  | None |  | None |
| chr13 | 32902066 | 32902067 | A | G | BRCA2 |  |  |  | ENST00000380152 | 1 | None | intronic | LOW |  | None |  | None |
| chr13 | 32903684 | 32903685 | C | T | BRCA2 |  |  |  | ENST00000530893 | 1 | None | intronic | LOW |  | None |  | None |
| chr13 | 32905264 | 32905265 | G | A | BRCA2 |  |  |  | ENST00000530893 | 1 | None | intronic | LOW |  | None |  | None |
| chr13 | 32905627 | 32905628 | C | A | BRCA2 |  |  |  | ENST00000530893 | 1 | None | intronic | LOW |  | None |  | None |
| chr13 | 32905997 | 32905998 | A | G | BRCA2 |  |  |  | ENST00000380152 | 1 | None | intronic | LOW |  | None |  | None |
| chr13 | 32906479 | 32906480 | A | C | BRCA2 | 10/28 | Aat/Cat | N/H | ENST00000544455 | 1 | None | missense | MED | Probably damaging | 0.98 | tolerated | 0.12 |
| chr13 | 32906979 | 32906980 | A | G | BRCA2 | 10/28 | tcA/tcG | S | ENST00000544455 | 1 | None | synonymous | LOW |  | None |  | None |
| chr13 | 32907766 | 32907767 | C | T | BRCA2 |  |  |  | ENST00000380152 | 1 | None | intronic | LOW |  | None |  | None |
| chr13 | 32908105 | 32908106 | A | G | BRCA2 |  |  |  | ENST00000380152 | 1 | None | intronic | LOW |  | None |  | None |
| chr13 | 32908346 | 32908347 | C | T | BRCA2 |  |  |  | ENST00000544455 | 1 | None | intronic | LOW |  | None |  | None |
| chr13 | 32910055 | 32910056 | T | G | BRCA2 |  |  |  | ENST00000380152 | 1 | None | intronic | LOW |  | None |  | None |
| chr13 | 32910327 | 32910328 | T | C | BRCA2 |  |  |  | ENST00000380152 | 1 | None | intronic | LOW |  | None |  | None |
| chr13 | 32910350 | 32910351 | G | T | BRCA2 |  |  |  | ENST00000544455 | 1 | None | intronic | LOW |  | None |  | None |
| chr13 | 32910720 | 32910721 | T | C | BRCA2 | 11/28 | caT/caC | H | ENST00000544455 | 1 | None | synonymous | LOW |  | None |  | None |
| chr13 | 32911462 | 32911463 | A | G | BRCA2 | 11/27 | Aac/Gac | N/D | ENST00000380152 | 1 | None | missense | MED | benign | 0 | tolerated | 1 |
| chr13 | 32913054 | 32913055 | A | G | BRCA2 | 11/27 | ctA/ctG | L | ENST00000380152 | 1 | None | synonymous | LOW |  | None |  | None |
| chr13 | 32915004 | 32915005 | G | C | BRCA2 | 11/28 | gtG/gtC | V | ENST00000544455 | 1 | None | synonymous | LOW |  | None |  | None |
| chr13 | 32915805 | 32915806 | A | G | BRCA2 |  |  |  | ENST00000544455 | 1 | None | intronic | LOW |  | None |  | None |
| chr13 | 32915972 | 32915973 | C | G | BRCA2 |  |  |  | ENST00000380152 | 1 | None | intronic | LOW |  | None |  | None |
| chr13 | 32916788 | 32916789 | A | G | BRCA2 |  |  |  | ENST00000544455 | 1 | None | intronic | LOW |  | None |  | None |
| chr13 | 32918221 | 32918222 | A | G | BRCA2 |  |  |  | ENST00000380152 | 1 | None | intronic | LOW |  | None |  | None |
| chr13 | 32918940 | 32918941 | A | G | BRCA2 |  |  |  | ENST00000544455 | 1 | None | intronic | LOW |  | None |  | None |
| chr13 | 32918966 | 32918967 | G | A | BRCA2 |  |  |  | ENST00000380152 | 1 | None | intronic | LOW |  | None |  | None |
| chr13 | 32919229 | 32919230 | C | T | BRCA2 |  |  |  | ENST00000544455 | 1 | None | intronic | LOW |  | None |  | None |
| chr13 | 32919525 | 32919526 | A | T | BRCA2 |  |  |  | ENST00000544455 | 1 | None | intronic | LOW |  | None |  | None |
| chr13 | 32920015 | 32920016 | G | A | BRCA2 |  |  |  | ENST00000544455 | 1 | None | intronic | LOW |  | None |  | None |
| chr13 | 32920339 | 32920340 | G | T | BRCA2 |  |  |  | ENST00000544455 | 1 | None | intronic | LOW |  | None |  | None |
| chr13 | 32920843 | 32920844 | T | C | BRCA2 |  |  |  | ENST00000380152 | 1 | None | intronic | LOW |  | None |  | None |
| chr13 | 32921305 | 32921306 | G | A | BRCA2 |  |  |  | ENST00000544455 | 1 | None | intronic | LOW |  | None |  | None |
| chr13 | 32921361 | 32921362 | T | C | BRCA2 |  |  |  | ENST00000380152 | 1 | None | intronic | LOW |  | None |  | None |
| chr13 | 32921535 | 32921536 | A | G | BRCA2 |  |  |  | ENST00000544455 | 1 | None | intronic | LOW |  | None |  | None |
| chr13 | 32923689 | 32923690 | C | T | BRCA2 |  |  |  | ENST00000544455 | 1 | None | intronic | LOW |  | None |  | None |
| chr13 | 32923987 | 32923988 | A | G | BRCA2 |  |  |  | ENST00000544455 | 1 | None | intronic | LOW |  | None |  | None |
| chr13 | 32925256 | 32925257 | C | A | BRCA2 |  |  |  | ENST00000380152 | 1 | None | intronic | LOW |  | None |  | None |
| chr13 | 32926045 | 32926046 | C | G | BRCA2 |  |  |  | ENST00000544455 | 1 | None | intronic | LOW |  | None |  | None |
| chr13 | 32926653 | 32926654 | T | G | BRCA2 |  |  |  | ENST00000544455 | 1 | None | intronic | LOW |  | None |  | None |
| chr13 | 32926943 | 32926944 | A | G | BRCA2 |  |  |  | ENST00000380152 | 1 | None | intronic | LOW |  | None |  | None |
| chr13 | 32927893 | 32927894 | C | T | BRCA2 |  |  |  | ENST00000544455 | 1 | None | intronic | LOW |  | None |  | None |
| chr13 | 32928111 | 32928112 | G | A | BRCA2 |  |  |  | ENST00000380152 | 1 | None | intronic | LOW |  | None |  | None |
| chr13 | 32928201 | 32928202 | A | G | BRCA2 |  |  |  | ENST00000380152 | 1 | None | intronic | LOW |  | None |  | None |
| chr13 | 32928316 | 32928317 | T | C | BRCA2 |  |  |  | ENST00000544455 | 1 | None | intronic | LOW |  | None |  | None |
| chr13 | 32929386 | 32929387 | T | C | BRCA2 | 14/27 | gTa/gCa | V/A | ENST00000380152 | 1 | None | missense | MED | benign | 0 | tolerated | 0.9 |
| chr13 | 32929477 | 32929478 | C | T | BRCA2 |  |  |  | ENST00000544455 | 1 | None | intronic | LOW |  | None |  | None |
| chr13 | 32930935 | 32930936 | G | A | BRCA2 |  |  |  | ENST00000380152 | 1 | None | intronic | LOW |  | None |  | None |
| chr13 | 32933936 | 32933937 | A | G | BRCA2 |  |  |  | ENST00000380152 | 1 | None | intronic | LOW |  | None |  | None |
| chr13 | 32935405 | 32935406 | T | C | BRCA2 |  |  |  | ENST00000380152 | 1 | None | intronic | LOW |  | None |  | None |
| chr13 | 32936645 | 32936646 | T | C | BRCA2 |  |  |  | ENST00000544455 | 1 | None | intronic | LOW |  | None |  | None |
| chr13 | 32938172 | 32938173 | A | C | BRCA2 |  |  |  | ENST00000544455 | 1 | None | intronic | LOW |  | None |  | None |
| chr13 | 32939084 | 32939085 | C | T | BRCA2 |  |  |  | ENST00000380152 | 1 | None | intronic | LOW |  | None |  | None |
| chr13 | 32939285 | 32939286 | T | G | BRCA2 |  |  |  | ENST00000544455 | 1 | None | intronic | LOW |  | None |  | None |
| chr13 | 32939313 | 32939314 | C | T | BRCA2 |  |  |  | ENST00000544455 | 1 | None | intronic | LOW |  | None |  | None |
| chr13 | 32940344 | 32940345 | C | A | BRCA2 |  |  |  | ENST00000380152 | 1 | None | intronic | LOW |  | None |  | None |
| chr13 | 32940887 | 32940888 | C | T | BRCA2 |  |  |  | ENST00000544455 | 1 | None | intronic | LOW |  | None |  | None |
| chr13 | 32942436 | 32942437 | T | G | BRCA2 |  |  |  | ENST00000380152 | 1 | None | intronic | LOW |  | None |  | None |
| chr13 | 32942935 | 32942936 | T | G | BRCA2 |  |  |  | ENST00000544455 | 1 | None | intronic | LOW |  | None |  | None |
| chr13 | 32943134 | 32943135 | C | T | BRCA2 |  |  |  | ENST00000380152 | 1 | None | intronic | LOW |  | None |  | None |
| chr13 | 32943781 | 32943782 | C | T | BRCA2 |  |  |  | ENST00000380152 | 1 | None | intronic | LOW |  | None |  | None |
| chr13 | 32944097 | 32944098 | G | A | BRCA2 |  |  |  | ENST00000380152 | 1 | None | intronic | LOW |  | None |  | None |
| chr13 | 32945536 | 32945537 | A | T | BRCA2 |  |  |  | ENST00000544455 | 1 | None | intronic | LOW |  | None |  | None |
| chr13 | 32946010 | 32946011 | A | G | BRCA2 |  |  |  | ENST00000380152 | 1 | None | intronic | LOW |  | None |  | None |
| chr13 | 32946414 | 32946415 | C | T | BRCA2 |  |  |  | ENST00000544455 | 1 | None | intronic | LOW |  | None |  | None |
| chr13 | 32947660 | 32947661 | A | T | BRCA2 |  |  |  | ENST00000544455 | 1 | None | intronic | LOW |  | None |  | None |
| chr13 | 32949381 | 32949382 | T | C | BRCA2 |  |  |  | ENST00000544455 | 1 | None | intronic | LOW |  | None |  | None |
| chr13 | 32950256 | 32950257 | A | G | BRCA2 |  |  |  | ENST00000380152 | 1 | None | intronic | LOW |  | None |  | None |
| chr13 | 32951595 | 32951596 | A | C | BRCA2 |  |  |  | ENST00000380152 | 1 | None | intronic | LOW |  | None |  | None |
| chr13 | 32953181 | 32953182 | A | G | BRCA2 |  |  |  | ENST00000544455 | 1 | None | intronic | LOW |  | None |  | None |
| chr13 | 32953387 | 32953388 | T | C | BRCA2 |  |  |  | ENST00000380152 | 1 | None | intronic | LOW |  | None |  | None |
| chr13 | 32953549 | 32953550 | G | A | BRCA2 | 22/28 | Gcc/Acc | A/T | ENST00000544455 | 1 | None | missense | MED | Probably damaging | 0.956 | deleterious | 0 |
| chr13 | 32954560 | 32954561 | G | A | BRCA2 |  |  |  | ENST00000380152 | 1 | None | intronic | LOW |  | None |  | None |
| chr13 | 32954740 | 32954741 | T | A | BRCA2 |  |  |  | ENST00000544455 | 1 | None | intronic | LOW |  | None |  | None |
| chr13 | 32955396 | 32955397 | C | T | BRCA2 |  |  |  | ENST00000380152 | 1 | None | intronic | LOW |  | None |  | None |
| chr13 | 32956108 | 32956109 | G | A | BRCA2 |  |  |  | ENST00000380152 | 1 | None | intronic | LOW |  | None |  | None |
| chr13 | 32956282 | 32956283 | G | A | BRCA2 |  |  |  | ENST00000544455 | 1 | None | intronic | LOW |  | None |  | None |
| chr13 | 32959198 | 32959199 | C | T | BRCA2 |  |  |  | ENST00000380152 | 1 | None | intronic | LOW |  | None |  | None |
| chr13 | 32959838 | 32959839 | A | C | BRCA2 |  |  |  | ENST00000544455 | 1 | None | intronic | LOW |  | None |  | None |
| chr13 | 32960362 | 32960363 | T | G | BRCA2 |  |  |  | ENST00000380152 | 1 | None | intronic | LOW |  | None |  | None |
| chr13 | 32962044 | 32962045 | A | C | BRCA2 |  |  |  | ENST00000544455 | 1 | None | intronic | LOW |  | None |  | None |
| chr13 | 32962087 | 32962088 | C | T | BRCA2 |  |  |  | ENST00000380152 | 1 | None | intronic | LOW |  | None |  | None |
| chr13 | 32962317 | 32962318 | T | C | BRCA2 |  |  |  | ENST00000380152 | 1 | None | intronic | LOW |  | None |  | None |
| chr13 | 32962678 | 32962679 | T | A | BRCA2 |  |  |  | ENST00000380152 | 1 | None | intronic | LOW |  | None |  | None |
| chr13 | 32963706 | 32963707 | C | T | BRCA2 |  |  |  | ENST00000544455 | 1 | None | intronic | LOW |  | None |  | None |
| chr13 | 32965650 | 32965651 | T | C | BRCA2 |  |  |  | ENST00000380152 | 1 | None | intronic | LOW |  | None |  | None |
| chr13 | 32965763 | 32965764 | C | T | BRCA2 |  |  |  | ENST00000380152 | 1 | None | intronic | LOW |  | None |  | None |
| chr13 | 32966667 | 32966668 | G | A | BRCA2 |  |  |  | ENST00000544455 | 1 | None | intronic | LOW |  | None |  | None |
| chr13 | 32967245 | 32967246 | A | C | BRCA2 |  |  |  | ENST00000380152 | 1 | None | intronic | LOW |  | None |  | None |
| chr13 | 32967465 | 32967466 | A | G | BRCA2 |  |  |  | ENST00000544455 | 1 | None | intronic | LOW |  | None |  | None |
| chr13 | 32968590 | 32968591 | G | A | BRCA2 |  |  |  | ENST00000544455 | 1 | None | intronic | LOW |  | None |  | None |
| chr13 | 32968606 | 32968607 | A | G | BRCA2 |  |  |  | ENST00000380152 | 1 | None | intronic | LOW |  | None |  | None |
| chr13 | 32970030 | 32970031 | T | C | BRCA2 |  |  |  | ENST00000544455 | 1 | None | intronic | LOW |  | None |  | None |
| chr13 | 32970598 | 32970599 | A | G | BRCA2 |  |  |  | ENST00000380152 | 1 | None | intronic | LOW |  | None |  | None |
| chr13 | 32970735 | 32970736 | G | C | BRCA2 |  |  |  | ENST00000380152 | 1 | None | intronic | LOW |  | None |  | None |
| chr13 | 32971424 | 32971425 | T | C | BRCA2 |  |  |  | ENST00000544455 | 1 | None | intronic | LOW |  | None |  | None |
| chr13 | 32973275 | 32973276 | A | G | BRCA2 | 27/27 |  |  | ENST00000380152 | 1 | None | 3 prime UTR | LOW |  | None |  | None |
| chr17 | 41196407 | 41196408 | G | A | BRCA1 | 22/22 |  |  | ENST00000352993 | 1 | None | 3 prime UTR | LOW |  | None |  | None |
| chr17 | 41197273 | 41197274 | C | A | BRCA1 | 22/22 |  |  | ENST00000468300 | 1 | None | 3 prime UTR | LOW |  | None |  | None |
| chr17 | 41198620 | 41198621 | A | G | BRCA1 |  |  |  | ENST00000352993 | 1 | None | intronic | LOW |  | None |  | None |
| chr17 | 41199912 | 41199913 | T | C | BRCA1 |  |  |  | ENST00000354071 | 1 | None | intronic | LOW |  | None |  | None |
| chr17 | 41200108 | 41200109 | T | C | BRCA1 |  |  |  | ENST00000591849 | 1 | None | intronic | LOW |  | None |  | None |
| chr17 | 41200536 | 41200537 | T | C | BRCA1 |  |  |  | ENST00000354071 | 1 | None | intronic | LOW |  | None |  | None |
| chr17 | 41201701 | 41201702 | C | T | BRCA1 |  |  |  | ENST00000468300 | 1 | None | intronic | LOW |  | None |  | None |
| chr17 | 41202687 | 41202688 | G | A | BRCA1 |  |  |  | ENST00000468300 | 1 | None | intronic | LOW |  | None |  | None |
| chr17 | 41203324 | 41203325 | T | A | BRCA1 |  |  |  | ENST00000491747 | 1 | None | intronic | LOW |  | None |  | None |
| chr17 | 41203590 | 41203591 | T | C | BRCA1 |  |  |  | ENST00000491747 | 1 | None | intronic | LOW |  | None |  | None |
| chr17 | 41204376 | 41204377 | A | G | BRCA1 |  |  |  | ENST00000586385 | 1 | None | intronic | LOW |  | None |  | None |
| chr17 | 41204389 | 41204390 | T | C | BRCA1 |  |  |  | ENST00000468300 | 1 | None | intronic | LOW |  | None |  | None |
| chr17 | 41205771 | 41205772 | G | A | BRCA1 |  |  |  | ENST00000357654 | 1 | None | intronic | LOW |  | None |  | None |
| chr17 | 41205940 | 41205941 | A | G | BRCA1 |  |  |  | ENST00000591849 | 1 | None | intronic | LOW |  | None |  | None |
| chr17 | 41206055 | 41206056 | T | C | BRCA1 |  |  |  | ENST00000357654 | 1 | None | intronic | LOW |  | None |  | None |
| chr17 | 41209577 | 41209578 | T | C | BRCA1 |  |  |  | ENST00000357654 | 1 | None | intronic | LOW |  | None |  | None |
| chr17 | 41210395 | 41210396 | A | C | BRCA1 |  |  |  | ENST00000354071 | 1 | None | intronic | LOW |  | None |  | None |
| chr17 | 41211652 | 41211653 | A | G | BRCA1 |  |  |  | ENST00000586385 | 1 | None | intronic | LOW |  | None |  | None |
| chr17 | 41212168 | 41212169 | C | T | BRCA1 |  |  |  | ENST00000351666 | 1 | None | intronic | LOW |  | None |  | None |
| chr17 | 41212337 | 41212338 | A | C | BRCA1 |  |  |  | ENST00000586385 | 1 | None | intronic | LOW |  | None |  | None |
| chr17 | 41212546 | 41212547 | C | T | BRCA1 |  |  |  | ENST00000493795 | 1 | None | intronic | LOW |  | None |  | None |
| chr17 | 41212804 | 41212805 | C | T | BRCA1 |  |  |  | ENST00000357654 | 1 | None | intronic | LOW |  | None |  | None |
| chr17 | 41213625 | 41213626 | G | T | BRCA1 |  |  |  | ENST00000586385 | 1 | None | intronic | LOW |  | None |  | None |
| chr17 | 41213659 | 41213660 | T | C | BRCA1 |  |  |  | ENST00000493795 | 1 | None | intronic | LOW |  | None |  | None |
| chr17 | 41213892 | 41213893 | C | T | BRCA1 |  |  |  | ENST00000351666 | 1 | None | intronic | LOW |  | None |  | None |
| chr17 | 41213995 | 41213996 | C | T | BRCA1 |  |  |  | ENST00000352993 | 1 | None | intronic | LOW |  | None |  | None |
| chr17 | 41215824 | 41215825 | C | T | BRCA1 |  |  |  | ENST00000586385 | 1 | None | intronic | LOW |  | None |  | None |
| chr17 | 41216204 | 41216205 | G | T | BRCA1 |  |  |  | ENST00000471181 | 1 | None | intronic | LOW |  | None |  | None |
| chr17 | 41216932 | 41216933 | T | C | BRCA1 |  |  |  | ENST00000491747 | 1 | None | intronic | LOW |  | None |  | None |
| chr17 | 41217873 | 41217874 | C | T | BRCA1 |  |  |  | ENST00000357654 | 1 | None | intronic | LOW |  | None |  | None |
| chr17 | 41218332 | 41218333 | G | A | BRCA1 |  |  |  | ENST00000478531 | 1 | None | intronic | LOW |  | None |  | None |
| chr17 | 41218571 | 41218572 | T | C | BRCA1 |  |  |  | ENST00000309486 | 1 | None | intronic | LOW |  | None |  | None |
| chr17 | 41219340 | 41219341 | G | T | BRCA1 |  |  |  | ENST00000591849 | 1 | None | intronic | LOW |  | None |  | None |
| chr17 | 41219559 | 41219560 | C | T | BRCA1 |  |  |  | ENST00000586385 | 1 | COSN165430 | intronic | LOW |  | None |  | None |
| chr17 | 41219779 | 41219780 | T | C | BRCA1 |  |  |  | ENST00000309486 | 1 | None | intronic | LOW |  | None |  | None |
| chr17 | 41219803 | 41219804 | T | C | BRCA1 |  |  |  | ENST00000468300 | 1 | None | intronic | LOW |  | None |  | None |
| chr17 | 41220222 | 41220223 | A | G | BRCA1 |  |  |  | ENST00000586385 | 1 | None | intronic | LOW |  | None |  | None |
| chr17 | 41220287 | 41220288 | C | T | BRCA1 |  |  |  | ENST00000586385 | 1 | None | intronic | LOW |  | None |  | None |
| chr17 | 41222461 | 41222462 | A | G | BRCA1 |  |  |  | ENST00000354071 | 1 | None | intronic | LOW |  | None |  | None |
| chr17 | 41222722 | 41222723 | T | C | BRCA1 |  |  |  | ENST00000357654 | 1 | None | intronic | LOW |  | None |  | None |
| chr17 | 41223093 | 41223094 | T | C | BRCA1 | 14/22 | Agt/Ggt | S/G | ENST00000309486 | 1 | None | missense | MED | benign | 0.006 | tolerated | 0.23 |
| chr17 | 41224832 | 41224833 | G | C | BRCA1 |  |  |  | ENST00000484087 | 1 | None | intronic | LOW |  | None |  | None |
| chr17 | 41225838 | 41225839 | T | C | BRCA1 |  |  |  | ENST00000357654 | 1 | None | intronic | LOW |  | None |  | None |
| chr17 | 41226600 | 41226601 | G | C | BRCA1 |  |  |  | ENST00000309486 | 1 | None | intronic | LOW |  | None |  | None |
| chr17 | 41226674 | 41226675 | A | T | BRCA1 |  |  |  | ENST00000309486 | 1 | None | intronic | LOW |  | None |  | None |
| chr17 | 41229385 | 41229386 | T | C | BRCA1 |  |  |  | ENST00000309486 | 1 | None | intronic | LOW |  | None |  | None |
| chr17 | 41229772 | 41229773 | T | C | BRCA1 |  |  |  | ENST00000461574 | 1 | None | intronic | LOW |  | None |  | None |
| chr17 | 41229811 | 41229812 | A | G | BRCA1 |  |  |  | ENST00000354071 | 1 | None | intronic | LOW |  | None |  | None |
| chr17 | 41229856 | 41229857 | G | A | BRCA1 |  |  |  | ENST00000487825 | 1 | None | intronic | LOW |  | None |  | None |
| chr17 | 41229907 | 41229908 | T | A | BRCA1 |  |  |  | ENST00000357654 | 1 | None | intronic | LOW |  | None |  | None |
| chr17 | 41230227 | 41230228 | G | A | BRCA1 |  |  |  | ENST00000591534 | 1 | None | intronic | LOW |  | None |  | None |
| chr17 | 41230335 | 41230336 | A | G | BRCA1 |  |  |  | ENST00000493795 | 1 | None | intronic | LOW |  | None |  | None |
| chr17 | 41230375 | 41230376 | A | G | BRCA1 |  |  |  | ENST00000586385 | 1 | None | intronic | LOW |  | None |  | None |
| chr17 | 41230523 | 41230524 | T | G | BRCA1 |  |  |  | ENST00000357654 | 1 | None | intronic | LOW |  | None |  | None |
| chr17 | 41230536 | 41230537 | A | T | BRCA1 |  |  |  | ENST00000357654 | 1 | None | intronic | LOW |  | None |  | None |
| chr17 | 41230954 | 41230955 | G | A | BRCA1 |  |  |  | ENST00000351666 | 1 | None | intronic | LOW |  | None |  | None |
| chr17 | 41230989 | 41230990 | A | G | BRCA1 |  |  |  | ENST00000357654 | 1 | None | intronic | LOW |  | None |  | None |
| chr17 | 41231220 | 41231221 | A | C | BRCA1 |  |  |  | ENST00000478531 | 1 | None | intronic | LOW |  | None |  | None |
| chr17 | 41231515 | 41231516 | C | T | BRCA1 |  |  |  | ENST00000491747 | 1 | None | intronic | LOW |  | None |  | None |
| chr17 | 41231901 | 41231902 | G | A | BRCA1 |  |  |  | ENST00000357654 | 1 | None | intronic | LOW |  | None |  | None |
| chr17 | 41232343 | 41232344 | G | C | BRCA1 |  |  |  | ENST00000471181 | 1 | None | intronic | LOW |  | None |  | None |
| chr17 | 41232697 | 41232698 | C | T | BRCA1 |  |  |  | ENST00000354071 | 1 | None | intronic | LOW |  | None |  | None |
| chr17 | 41234469 | 41234470 | A | G | BRCA1 | 12/18 | tcT/tcC | S | ENST00000354071 | 1 | None | synonymous | LOW |  | None |  | None |
| chr17 | 41235798 | 41235799 | G | A | BRCA1 |  |  |  | ENST00000351666 | 1 | None | intronic | LOW |  | None |  | None |
| chr17 | 41237952 | 41237953 | G | A | BRCA1 |  |  |  | ENST00000351666 | 1 | None | intronic | LOW |  | None |  | None |
| chr17 | 41239471 | 41239472 | G | A | BRCA1 |  |  |  | ENST00000357654 | 1 | None | intronic | LOW |  | None |  | None |
| chr17 | 41239490 | 41239491 | T | C | BRCA1 |  |  |  | ENST00000357654 | 1 | None | intronic | LOW |  | None |  | None |
| chr17 | 41239627 | 41239628 | A | G | BRCA1 |  |  |  | ENST00000586385 | 1 | None | intronic | LOW |  | None |  | None |
| chr17 | 41240276 | 41240277 | T | C | BRCA1 |  |  |  | ENST00000471181 | 1 | None | intronic | LOW |  | None |  | None |
| chr17 | 41241389 | 41241390 | C | A | BRCA1 |  |  |  | ENST00000493795 | 1 | None | intronic | LOW |  | None |  | None |
| chr17 | 41241502 | 41241503 | T | C | BRCA1 |  |  |  | ENST00000354071 | 1 | None | intronic | LOW |  | None |  | None |
| chr17 | 41242284 | 41242285 | T | G | BRCA1 |  |  |  | ENST00000586385 | 1 | None | intronic | LOW |  | None |  | None |
| chr17 | 41243189 | 41243190 | T | G | BRCA1 |  |  |  | ENST00000468300 | 1 | None | intronic | LOW |  | None |  | None |
| chr17 | 41243202 | 41243203 | A | T | BRCA1 |  |  |  | ENST00000586385 | 0 | None | intronic | LOW |  | None |  | None |
| chr17 | 41243999 | 41244000 | T | C | BRCA1 | 9/22 | aAa/aGa | K/R | ENST00000309486 | 1 | COSM148277 | missense | MED | benign | 0.001 | tolerated | 1.0 |
| chr17 | 41244434 | 41244435 | T | C | BRCA1 | 9/22 | gAa/gGa | E/G | ENST00000309486 | 1 | None | missense | MED | benign | 0.033 | tolerated | 0.06 |
| chr17 | 41244935 | 41244936 | G | A | BRCA1 | 10/23 | cCg/cTg | P/L | ENST00000357654 | 1 | COSM148278 | missense | MED | benign | 0.0 | tolerated | 1.0 |
| chr17 | 41245236 | 41245237 | A | G | BRCA1 | 10/23 | Ttg/Ctg | L | ENST00000357654 | 1 | None | synonymous | LOW |  | None |  | None |
| chr17 | 41245465 | 41245466 | G | A | BRCA1 | 10/19 | agC/agT | S | ENST00000346315 | 1 | COSM148280 | synonymous | LOW |  | None |  | None |
| chr17 | 41247603 | 41247604 | A | C | BRCA1 |  |  |  | ENST00000487825 | 1 | None | intronic | LOW |  | None |  | None |
| chr17 | 41248163 | 41248164 | C | T | BRCA1 |  |  |  | ENST00000468300 | 1 | None | intronic | LOW |  | None |  | None |
| chr17 | 41248483 | 41248484 | G | C | BRCA1 |  |  |  | ENST00000493919 | 1 | None | intronic | LOW |  | None |  | None |
| chr17 | 41249093 | 41249094 | A | G | BRCA1 |  |  |  | ENST00000493919 | 1 | None | intronic | LOW |  | None |  | None |
| chr17 | 41250922 | 41250923 | T | C | BRCA1 |  |  |  | ENST00000491747 | 1 | None | intronic | LOW |  | None |  | None |
| chr17 | 41251494 | 41251495 | C | G | BRCA1 |  |  |  | ENST00000497488 | 1 | None | intronic | LOW |  | None |  | None |
| chr17 | 41251645 | 41251646 | T | A | BRCA1 |  |  |  | ENST00000484087 | 1 | None | intronic | LOW |  | None |  | None |
| chr17 | 41252574 | 41252575 | G | A | BRCA1 |  |  |  | ENST00000493795 | 1 | None | intronic | LOW |  | None |  | None |
| chr17 | 41254173 | 41254174 | A | G | BRCA1 |  |  |  | ENST00000591849 | 1 | None | intronic | LOW |  | None |  | None |
| chr17 | 41254404 | 41254405 | C | T | BRCA1 |  |  |  | ENST00000354071 | 1 | None | intronic | LOW |  | None |  | None |
| chr17 | 41254485 | 41254486 | T | G | BRCA1 |  |  |  | ENST00000477152 | 1 | None | intronic | LOW |  | None |  | None |
| chr17 | 41255101 | 41255102 | A | G | BRCA1 |  |  |  | ENST00000591849 | 1 | None | intronic | LOW |  | None |  | None |
| chr17 | 41255110 | 41255111 | A | T | BRCA1 |  |  |  | ENST00000357654 | 1 | None | intronic | LOW |  | None |  | None |
| chr17 | 41257133 | 41257134 | T | C | BRCA1 |  |  |  | ENST00000491747 | 1 | None | intronic | LOW |  | None |  | None |
| chr17 | 41257457 | 41257458 | A | C | BRCA1 |  |  |  | ENST00000470026 | 1 | None | intronic | LOW |  | None |  | None |
| chr17 | 41258042 | 41258043 | C | T | BRCA1 |  |  |  | ENST00000591849 | 1 | None | intronic | LOW |  | None |  | None |
| chr17 | 41259048 | 41259049 | C | T | BRCA1 |  |  |  | ENST00000354071 | 1 | None | intronic | LOW |  | None |  | None |
| chr17 | 41260807 | 41260808 | A | G | BRCA1 |  |  |  | ENST00000468300 | 1 | None | intronic | LOW |  | None |  | None |
| chr17 | 41261232 | 41261233 | C | T | BRCA1 |  |  |  | ENST00000591849 | 1 | None | intronic | LOW |  | None |  | None |
| chr17 | 41262356 | 41262357 | A | G | BRCA1 |  |  |  | ENST00000489037 | 1 | None | intronic | LOW |  | None |  | None |
| chr17 | 41263043 | 41263044 | A | G | BRCA1 |  |  |  | ENST00000477152 | 1 | None | intronic | LOW |  | None |  | None |
| chr17 | 41263565 | 41263566 | T | C | BRCA1 |  |  |  | ENST00000491747 | 1 | None | intronic | LOW |  | None |  | None |
| chr17 | 41264145 | 41264146 | G | A | BRCA1 |  |  |  | ENST00000471181 | 1 | None | intronic | LOW |  | None |  | None |
| chr17 | 41264363 | 41264364 | A | G | BRCA1 |  |  |  | ENST00000354071 | 1 | None | intronic | LOW |  | None |  | None |
| chr17 | 41265775 | 41265776 | A | G | BRCA1 |  |  |  | ENST00000493795 | 1 | None | intronic | LOW |  | None |  | None |
| chr17 | 41267049 | 41267050 | G | A | BRCA1 |  |  |  | ENST00000354071 | 1 | None | intronic | LOW |  | None |  | None |
| chr17 | 41270228 | 41270229 | T | G | BRCA1 |  |  |  | ENST00000493795 | 1 | None | intronic | LOW |  | None |  | None |
| chr17 | 41270276 | 41270277 | C | T | BRCA1 |  |  |  | ENST00000493919 | 1 | None | intronic | LOW |  | None |  | None |
| chr17 | 41270462 | 41270463 | G | A | BRCA1 |  |  |  | ENST00000468300 | 1 | None | intronic | LOW |  | None |  | None |
| chr17 | 41270665 | 41270666 | C | A | BRCA1 |  |  |  | ENST00000357654 | 1 | None | intronic | LOW |  | None |  | None |
| chr17 | 41273094 | 41273095 | G | A | BRCA1 |  |  |  | ENST00000491747 | 1 | None | intronic | LOW |  | None |  | None |
| chr17 | 41273347 | 41273348 | T | C | BRCA1 |  |  |  | ENST00000493795 | 1 | None | intronic | LOW |  | None |  | None |
| chr17 | 41273378 | 41273379 | G | C | BRCA1 |  |  |  | ENST00000357654 | 1 | None | intronic | LOW |  | None |  | None |
| chr17 | 41273536 | 41273537 | A | C | BRCA1 |  |  |  | ENST00000591849 | 1 | None | intronic | LOW |  | None |  | None |
| chr17 | 41274777 | 41274778 | G | A | BRCA1 |  |  |  | ENST00000586385 | 1 | None | intronic | LOW |  | None |  | None |
| chr17 | 41274905 | 41274906 | G | A | BRCA1 |  |  |  | ENST00000471181 | 1 | None | intronic | LOW |  | None |  | None |
| chr17 | 41275150 | 41275151 | G | C | BRCA1 |  |  |  | ENST00000591849 | 1 | None | intronic | LOW |  | None |  | None |
| chr17 | 41275644 | 41275645 | A | G | BRCA1 |  |  |  | ENST00000494123 | 1 | None | intronic | LOW |  | None |  | None |
| chr17 | 41276246 | 41276247 | A | G | BRCA1 |  |  |  | ENST00000357654 | 1 | None | intronic | LOW |  | None |  | None |
| chr17 | 41276347 | 41276348 | T | C | BRCA1 |  |  |  | ENST00000352993 | 1 | None | intronic | LOW |  | None |  | None |
| chr17 | 41277186 | 41277187 | G | C | BRCA1 |  |  |  | ENST00000346315 | 1 | None | intronic | LOW |  | None |  | None |

**Part B: *BRCA1/2* indels (n=53)**

| **chr** | **start** | **end** | **ref** | **alt** | **gene** | **transcript** | **In dbsnp** | **cosmic ids** | **Variant impact** | **Impact severity** | **polyphen score** | **sift score** |
| --- | --- | --- | --- | --- | --- | --- | --- | --- | --- | --- | --- | --- |
| chr13 | 32892575 | 32892577 | AT | A | BRCA2 | ENST00000544455 | 1 | None | intronic | LOW | None | None |
| chr13 | 32897287 | 32897290 | ATT | A | BRCA2 | ENST00000530893 | 1 | None | intronic | LOW | None | None |
| chr13 | 32916406 | 32916410 | CAAA | C | BRCA2 | ENST00000380152 | 0 | None | intronic | LOW | None | None |
| chr13 | 32918302 | 32918304 | GA | G | BRCA2 | ENST00000380152 | 1 | None | intronic | LOW | None | None |
| chr13 | 32918302 | 32918305 | GAA | G | BRCA2 | ENST00000544455 | 1 | None | intronic | LOW | None | None |
| chr13 | 32921147 | 32921148 | T | TTTTATAAAA | BRCA2 | ENST00000380152 | 1 | None | intronic | LOW | None | None |
| chr13 | 32923458 | 32923459 | C | CA | BRCA2 | ENST00000544455 | 1 | None | intronic | LOW | None | None |
| chr13 | 32939857 | 32939858 | C | CT | BRCA2 | ENST00000380152 | 1 | None | intronic | LOW | None | None |
| chr13 | 32941576 | 32941578 | GA | G | BRCA2 | ENST00000380152 | 1 | None | intronic | LOW | None | None |
| chr13 | 32941715 | 32941716 | A | AGATGGCTTG | BRCA2 | ENST00000380152 | 1 | None | intronic | LOW | None | None |
| chr13 | 32943341 | 32943344 | TAA | T | BRCA2 | ENST00000380152 | 1 | None | intronic | LOW | None | None |
| chr13 | 32943354 | 32943356 | AT | A | BRCA2 | ENST00000380152 | 1 | None | intronic | LOW | None | None |
| chr13 | 32947132 | 32947133 | C | CT | BRCA2 | ENST00000544455 | 1 | None | intronic | LOW | None | None |
| chr13 | 32949699 | 32949700 | C | CT | BRCA2 | ENST00000380152 | 1 | None | intronic | LOW | None | None |
| chr13 | 32951726 | 32951728 | CA | C | BRCA2 | ENST00000380152 | 1 | None | intronic | LOW | None | None |
| chr13 | 32961188 | 32961189 | G | GC | BRCA2 | ENST00000544455 | 1 | None | intronic | LOW | None | None |
| chr13 | 32962257 | 32962258 | C | CT | BRCA2 | ENST00000544455 | 1 | None | intronic | LOW | None | None |
| chr13 | 32962802 | 32962803 | C | CT | BRCA2 | ENST00000544455 | 1 | None | intronic | LOW | None | None |
| chr13 | 32971705 | 32971707 | AT | A | BRCA2 | ENST00000380152 | 1 | None | intronic | LOW | None | None |
| chr13 | 32973922 | 32973923 | C | CT | N4BP2L1/BRCA2 | ENST00000530622 | 1 | None | Downstream gene | LOW | None | None |
| chr17 | 41196820 | 41196823 | CTT | C | BRCA1 | ENST00000468300 | 1 | None | 3 prime UTR | LOW | None | None |
| chr17 | 41198773 | 41198774 | C | CA | BRCA1 | ENST00000346315 | 1 | None | intronic | LOW | None | None |
| chr17 | 41200702 | 41200704 | TG | T | BRCA1 | ENST00000354071 | 0 | None | intronic | LOW | None | None |
| chr17 | 41200703 | 41200704 | G | T | BRCA1 | ENST00000346315 | 0 | None | intronic | LOW | None | None |
| chr17 | 41206760 | 41206762 | AT | A | BRCA1 | ENST00000493795 | 1 | None | intronic | LOW | None | None |
| chr17 | 41214208 | 41214211 | CAA | C | BRCA1 | ENST00000352993 | 1 | None | intronic | LOW | None | None |
| chr17 | 41219852 | 41219853 | A | ATTTT | BRCA1 | ENST00000471181 | 1 | None | intronic | LOW | None | None |
| chr17 | 41223537 | 41223538 | G | GAATGTTCACTGTAACAATGCTTGT | BRCA1 | ENST00000491747 | 1 | None | intronic | LOW | None | None |
| chr17 | 41226735 | 41226741 | GGGGTT | G | BRCA1 | ENST00000357654 | 1 | None | intronic | LOW | None | None |
| chr17 | 41227082 | 41227083 | C | CGGAA | BRCA1 | ENST00000471181 | 1 | None | intronic | LOW | None | None |
| chr17 | 41229351 | 41229352 | C | CT | BRCA1 | ENST00000478531 | 1 | None | intronic | LOW | None | None |
| chr17 | 41229776 | 41229777 | G | GT | BRCA1 | ENST00000461574 | 1 | None | intronic | LOW | None | None |
| chr17 | 41230104 | 41230106 | CT | C | BRCA1 | ENST00000346315 | 1 | None | intronic | LOW | None | None |
| chr17 | 41238125 | 41238126 | T | TACACACACAC | BRCA1 | ENST00000591849 | 0 | None | intronic | LOW | None | None |
| chr17 | 41239914 | 41239916 | AT | A | BRCA1 | ENST00000487825 | 1 | None | intronic | LOW | None | None |
| chr17 | 41241567 | 41241568 | T | TC | BRCA1 | ENST00000586385 | 1 | None | intronic | LOW | None | None |
| chr17 | 41242074 | 41242078 | AAAG | A | BRCA1 | ENST00000478531 | 1 | None | intronic | LOW | None | None |
| chr17 | 41247121 | 41247122 | A | ACCT | BRCA1 | ENST00000471181 | 1 | None | intronic | LOW | None | None |
| chr17 | 41248587 | 41248589 | CA | C | BRCA1 | ENST00000487825 | 1 | None | intronic | LOW | None | None |
| chr17 | 41249362 | 41249364 | TA | T | BRCA1 | ENST00000477152 | 1 | None | intronic | LOW | None | None |
| chr17 | 41250046 | 41250047 | C | CT | BRCA1 | ENST00000493919 | 1 | None | intronic | LOW | None | None |
| chr17 | 41250677 | 41250678 | C | CT | BRCA1 | ENST00000471181 | 1 | None | intronic | LOW | None | None |
| chr17 | 41252663 | 41252664 | A | ATAT | BRCA1 | ENST00000497488 | 0 | None | intronic | LOW | None | None |
| chr17 | 41254964 | 41254965 | C | CT | BRCA1 | ENST00000471181 | 1 | None | intronic | LOW | None | None |
| chr17 | 41260351 | 41260352 | C | CA | BRCA1 | ENST00000357654 | 1 | None | intronic | LOW | None | None |
| chr17 | 41261057 | 41261058 | T | TCTATCTATCTACCTAC | BRCA1 | ENST00000478531 | 1 | None | intronic | LOW | None | None |
| chr17 | 41263116 | 41263117 | C | CA | BRCA1 | ENST00000493919 | 1 | None | intronic | LOW | None | None |
| chr17 | 41266406 | 41266407 | C | CT | BRCA1 | ENST00000470026 | 1 | None | intronic | LOW | None | None |
| chr17 | 41267517 | 41267519 | CA | C | BRCA1 | ENST00000497488 | 1 | None | intronic | LOW | None | None |
| chr17 | 41270777 | 41270778 | C | CT | BRCA1 | ENST00000352993 | 1 | None | intronic | LOW | None | None |
| chr17 | 41271292 | 41271294 | GA | G | BRCA1 | ENST00000309486 | 1 | None | intronic | LOW | None | None |
| chr17 | 41275080 | 41275081 | G | GA | BRCA1 | ENST00000491747 | 1 | None | intronic | LOW | None | None |
| chr17 | 41276763 | 41276769 | TTTTTG | T | BRCA1 | ENST00000478531 | 1 | None | intronic | LOW | None | None |

**Abbreviations**: chr, chromosome; start/end, nucleotide position; ref, reference sequence, al, alternate sequence / mutation; aa, amino acid; in dbSNP, 1=yes and 0=no.

Note that none of the indels are exonic and therefore none result in a codon or amino acid change, nor are they predictive to be functional as a consequence and no PolyPhen or Sift prediction occurred and these columns have therefore been excluded.
