# Supplementary material for: Next generation mapping reveals novel large genomic rearrangements in prostate cancer
Source: Oncotarget. 2017 Mar 1;8(14):23588–602. doi: 10.18632/oncotarget.15802 (PMC5410329; doi:10.18632/oncotarget.15802)
Supplement: Supplementary file 4 [file oncotarget-08-23588-s004.docx]

**Table S4. NGS-derived small somatic variants (SNVs and indels < 50 bp) detected in UP2153.**

**Part A: Somatic SNVs (n = 5,981)**

| chrom | start | end | ref | alt | gene | aa_change | transcript | in_dbsnp | cosmic_id | impact | impact_severity | polyphen pred | polyphen_score | sift_pred | sift_score | variant_freq |
| --- | --- | --- | --- | --- | --- | --- | --- | --- | --- | --- | --- | --- | --- | --- | --- | --- |
| chr1 | 725433 | 725434 | G | A | RP11-206L10.9 |  | ENST00000585768 | 0 | None | intron_variant | LOW |  | None |  | None | 0.098360656 |
| chr1 | 1016379 | 1016380 | T | C | C1orf159 |  | ENST00000421241 | 1 | None | downstream_gene_variant | LOW |  | None |  | None | 0.161290323 |
| chr1 | 1085175 | 1085176 | G | T | None |  |  | 0 | None | intergenic_variant | LOW |  | None |  | None | 0.243243243 |
| chr1 | 1134188 | 1134189 | A | C | TNFRSF18 |  | ENST00000328596 | 0 | None | downstream_gene_variant | LOW |  | None |  | None | 0.29787234 |
| chr1 | 1317790 | 1317791 | C | A | CCNL2 |  | ENST00000400809 | 1 | None | downstream_gene_variant | LOW |  | None |  | None | 0.071428571 |
| chr1 | 1412255 | 1412256 | G | T | ATAD3B |  | ENST00000378741 | 0 | None | intron_variant | LOW |  | None |  | None | 0.106060606 |
| chr1 | 1588542 | 1588543 | C | A | CDK11B |  | ENST00000340677 | 1 | None | intron_variant | LOW |  | None |  | None | 0.225352113 |
| chr1 | 2038119 | 2038120 | A | G | PRKCZ |  | ENST00000482686 | 0 | None | intron_variant | LOW |  | None |  | None | 0.23255814 |
| chr1 | 2073335 | 2073336 | G | A | PRKCZ |  | ENST00000400920 | 0 | None | intron_variant | LOW |  | None |  | None | 0.121621622 |
| chr1 | 2276572 | 2276573 | A | G | MORN1 |  | ENST00000378531 | 0 | None | intron_variant | LOW |  | None |  | None | 0.169014085 |
| chr1 | 2415223 | 2415224 | T | G | PLCH2 |  | ENST00000288766 | 1 | None | intron_variant | LOW |  | None |  | None | 0.185185185 |
| chr1 | 2563751 | 2563752 | G | A | MMEL1 |  | ENST00000288709 | 0 | None | intron_variant | LOW |  | None |  | None | 0.16 |
| chr1 | 2591669 | 2591670 | C | G | TTC34 |  | ENST00000401094 | 0 | None | intron_variant | LOW |  | None |  | None | 0.3125 |
| chr1 | 2617137 | 2617138 | G | C | TTC34 |  | ENST00000401095 | 0 | None | intron_variant | LOW |  | None |  | None | 0.033210332 |
| chr1 | 2624691 | 2624692 | A | G | TTC34 |  | ENST00000401095 | 0 | None | intron_variant | LOW |  | None |  | None | 0.111111111 |
| chr1 | 2627807 | 2627808 | G | T | TTC34 |  | ENST00000401095 | 0 | None | intron_variant | LOW |  | None |  | None | 0.18 |
| chr1 | 2627807 | 2627808 | G | C | TTC34 |  | ENST00000401094 | 1 | None | intron_variant | LOW |  | None |  | None | 0.40 |
| chr1 | 2627884 | 2627885 | T | C | TTC34 |  | ENST00000401094 | 0 | None | intron_variant | LOW |  | None |  | None | 0.082191781 |
| chr1 | 2685160 | 2685161 | C | A | TTC34 |  | ENST00000401095 | 0 | None | intron_variant | LOW |  | None |  | None | 0.193548387 |
| chr1 | 2715720 | 2715721 | G | A | TTC34 |  | ENST00000401094 | 0 | None | intron_variant | LOW |  | None |  | None | 0.133333333 |
| chr1 | 3556964 | 3556965 | G | A | WRAP73 |  | ENST00000465916 | 0 | None | intron_variant | LOW |  | None |  | None | 0.09375 |
| chr1 | 3611525 | 3611526 | G | C | TP73 |  | ENST00000604479 | 0 | None | intron_variant | LOW |  | None |  | None | 0.114285714 |
| chr1 | 4192707 | 4192708 | C | T | None |  |  | 0 | None | intergenic_variant | LOW |  | None |  | None | 0.296296296 |
| chr1 | 4945339 | 4945340 | T | C | None |  |  | 0 | None | intergenic_variant | LOW |  | None |  | None | 0.236363636 |
| chr1 | 5043006 | 5043007 | G | T | None |  |  | 0 | None | intergenic_variant | LOW |  | None |  | None | 0.122807018 |
| chr1 | 5075387 | 5075388 | C | T | None |  |  | 0 | None | intergenic_variant | LOW |  | None |  | None | 0.096774194 |
| chr1 | 5105624 | 5105625 | T | A | None |  |  | 0 | None | intergenic_variant | LOW |  | None |  | None | 0.102941176 |
| chr1 | 5105625 | 5105626 | G | T | None |  |  | 0 | None | intergenic_variant | LOW |  | None |  | None | 0.102941176 |
| chr1 | 5175737 | 5175738 | G | A | None |  |  | 0 | None | intergenic_variant | LOW |  | None |  | None | 0.125 |
| chr1 | 5490183 | 5490184 | C | A | None |  |  | 0 | None | intergenic_variant | LOW |  | None |  | None | 0.169230769 |
| chr1 | 5502554 | 5502555 | G | T | None |  |  | 0 | None | intergenic_variant | LOW |  | None |  | None | 0.208955224 |
| chr1 | 5715850 | 5715851 | C | A | RP11-154H17.1 |  | ENST00000413887 | 0 | None | intron_variant | LOW |  | None |  | None | 0.095890411 |
| chr1 | 6462420 | 6462421 | G | A | None |  |  | 0 | None | intergenic_variant | LOW |  | None |  | None | 0.177419355 |
| chr1 | 6516779 | 6516780 | C | T | ESPN |  | ENST00000377828 | 0 | None | intron_variant | LOW |  | None |  | None | 0.25 |
| chr1 | 7147067 | 7147068 | C | T | CAMTA1 |  | ENST00000303635 | 0 | None | intron_variant | LOW |  | None |  | None | 0.303030303 |
| chr1 | 7211192 | 7211193 | T | C | CAMTA1 |  | ENST00000439411 | 0 | None | intron_variant | LOW |  | None |  | None | 0.21875 |
| chr1 | 7356972 | 7356973 | G | C | CAMTA1 |  | ENST00000303635 | 0 | None | intron_variant | LOW |  | None |  | None | 0.245901639 |
| chr1 | 7751114 | 7751115 | A | C | CAMTA1 |  | ENST00000439411 | 0 | None | intron_variant | LOW |  | None |  | None | 0.261538462 |
| chr1 | 8652582 | 8652583 | G | A | RERE |  | ENST00000400908 | 0 | None | intron_variant | LOW |  | None |  | None | 0.275862069 |
| chr1 | 8903060 | 8903061 | G | A | None |  |  | 0 | None | intergenic_variant | LOW |  | None |  | None | 0.129032258 |
| chr1 | 8977858 | 8977859 | C | T | None |  |  | 0 | None | intergenic_variant | LOW |  | None |  | None | 0.233333333 |
| chr1 | 9169099 | 9169100 | T | G | GPR157 |  | ENST00000465853 | 0 | None | intron_variant | LOW |  | None |  | None | 0.135135135 |
| chr1 | 9387882 | 9387883 | C | G | SPSB1 |  | ENST00000328089 | 0 | None | intron_variant | LOW |  | None |  | None | 0.098591549 |
| chr1 | 10503951 | 10503952 | G | T | APITD1-CORT |  | ENST00000470413 | 0 | None | intron_variant | LOW |  | None |  | None | 0.148148148 |
| chr1 | 11697205 | 11697206 | C | T | None |  |  | 0 | None | intergenic_variant | LOW |  | None |  | None | 0.225806452 |
| chr1 | 11971994 | 11971995 | A | C | RNU5E-4P |  | ENST00000364931 | 0 | None | upstream_gene_variant | LOW |  | None |  | None | 0.263157895 |
| chr1 | 12268046 | 12268047 | C | T | TNFRSF1B |  | ENST00000376259 | 0 | None | 3_prime_UTR_variant | LOW |  | None |  | None | 0.196078431 |
| chr1 | 12538577 | 12538578 | A | G | VPS13D |  | ENST00000543766 | 0 | None | intron_variant | LOW |  | None |  | None | 0.207792208 |
| chr1 | 12582860 | 12582861 | C | T | RP5-888M10.2 |  | ENST00000416696 | 0 | None | upstream_gene_variant | LOW |  | None |  | None | 0.296296296 |
| chr1 | 13866766 | 13866767 | A | T | None |  |  | 0 | None | intergenic_variant | LOW |  | None |  | None | 0.138461538 |
| chr1 | 13872872 | 13872873 | G | T | None |  |  | 0 | None | intergenic_variant | LOW |  | None |  | None | 0.23943662 |
| chr1 | 14532432 | 14532433 | G | C | None |  |  | 0 | None | intergenic_variant | LOW |  | None |  | None | 0.125 |
| chr1 | 14585976 | 14585977 | G | A | None |  |  | 0 | None | intergenic_variant | LOW |  | None |  | None | 0.233333333 |
| chr1 | 16916199 | 16916200 | A | G | NBPF1 |  | ENST00000430580 | 0 | None | intron_variant | LOW |  | None |  | None | 0.065934066 |
| chr1 | 17027929 | 17027930 | G | C | ESPNP |  | ENST00000492551 | 1 | None | intron_variant | LOW |  | None |  | None | 0.284153005 |
| chr1 | 18045811 | 18045812 | T | G | RP11-473A10.2 |  | ENST00000430540 | 0 | None | intron_variant | LOW |  | None |  | None | 0.272727273 |
| chr1 | 18840956 | 18840957 | A | G | RP1-8B22.1 |  | ENST00000441355 | 0 | None | downstream_gene_variant | LOW |  | None |  | None | 0.296296296 |
| chr1 | 22445743 | 22445744 | C | T | WNT4 |  | ENST00000290167 | 1 | None | 3_prime_UTR_variant | LOW |  | None |  | None | 0.138888889 |
| chr1 | 22703663 | 22703664 | T | C | None |  |  | 0 | None | intergenic_variant | LOW |  | None |  | None | 0.095890411 |
| chr1 | 24831721 | 24831722 | C | T | RCAN3 |  | ENST00000374395 | 1 | None | intron_variant | LOW |  | None |  | None | 0.12962963 |
| chr1 | 26018080 | 26018081 | T | G | MAN1C1 |  | ENST00000263979 | 0 | None | intron_variant | LOW |  | None |  | None | 0.343283582 |
| chr1 | 26368583 | 26368584 | G | A | SLC30A2 |  | ENST00000374276 | 0 | None | intron_variant | LOW |  | None |  | None | 0.269230769 |
| chr1 | 27315511 | 27315512 | G | A | TRNP1 |  | ENST00000522111 | 0 | None | upstream_gene_variant | LOW |  | None |  | None | 0.377358491 |
| chr1 | 27952322 | 27952323 | G | A | FGR |  | ENST00000545953 | 0 | None | intron_variant | LOW |  | None |  | None | 0.157894737 |
| chr1 | 28093732 | 28093733 | A | G | FAM76A |  | ENST00000373954 | 0 | None | downstream_gene_variant | LOW |  | None |  | None | 0.31147541 |
| chr1 | 28633793 | 28633794 | G | A | None |  |  | 0 | None | intergenic_variant | LOW |  | None |  | None | 0.105263158 |
| chr1 | 29088620 | 29088621 | C | G | YTHDF2 |  | ENST00000541996 | 0 | None | intron_variant | LOW |  | None |  | None | 0.301587302 |
| chr1 | 29155458 | 29155459 | G | A | OPRD1 |  | ENST00000234961 | 1 | None | intron_variant | LOW |  | None |  | None | 0.109090909 |
| chr1 | 30111082 | 30111083 | G | T | None |  |  | 0 | None | intergenic_variant | LOW |  | None |  | None | 0.25 |
| chr1 | 30542510 | 30542511 | G | A | None |  |  | 0 | None | intergenic_variant | LOW |  | None |  | None | 0.101694915 |
| chr1 | 30549086 | 30549087 | A | C | None |  |  | 0 | None | intergenic_variant | LOW |  | None |  | None | 0.166666667 |
| chr1 | 30656023 | 30656024 | T | C | None |  |  | 0 | None | intergenic_variant | LOW |  | None |  | None | 0.310344828 |
| chr1 | 30873024 | 30873025 | C | T | None |  |  | 0 | None | intergenic_variant | LOW |  | None |  | None | 0.134615385 |
| chr1 | 30932054 | 30932055 | C | A | None |  |  | 0 | None | intergenic_variant | LOW |  | None |  | None | 0.166666667 |
| chr1 | 33503766 | 33503767 | A | C | AK2 |  | ENST00000548033 | 0 | None | upstream_gene_variant | LOW |  | None |  | None | 0.088235294 |
| chr1 | 33780551 | 33780552 | A | T | A3GALT2 |  | ENST00000442999 | 1 | None | intron_variant | LOW |  | None |  | None | 0.194029851 |
| chr1 | 35000172 | 35000173 | G | A | None |  |  | 1 | None | intergenic_variant | LOW |  | None |  | None | 0.287671233 |
| chr1 | 37255647 | 37255648 | C | T | None |  |  | 0 | None | intergenic_variant | LOW |  | None |  | None | 0.314285714 |
| chr1 | 37424166 | 37424167 | C | T | GRIK3 |  | ENST00000373093 | 0 | None | intron_variant | LOW |  | None |  | None | 0.107142857 |
| chr1 | 37870487 | 37870488 | A | G | None |  |  | 0 | None | intergenic_variant | LOW |  | None |  | None | 0.134328358 |
| chr1 | 40550774 | 40550775 | A | G | PPT1 |  | ENST00000433473 | 0 | None | intron_variant | LOW |  | None |  | None | 0.086956522 |
| chr1 | 40550811 | 40550812 | T | C | PPT1 |  | ENST00000433473 | 0 | None | intron_variant | LOW |  | None |  | None | 0.151515152 |
| chr1 | 40979288 | 40979289 | T | G | EXO5 |  | ENST00000418186 | 0 | None | intron_variant | LOW |  | None |  | None | 0.235294118 |
| chr1 | 42279767 | 42279768 | A | C | HIVEP3 |  | ENST00000372583 | 0 | None | intron_variant | LOW |  | None |  | None | 0.205479452 |
| chr1 | 43216258 | 43216259 | C | T | LEPRE1 |  | ENST00000296388 | 0 | None | intron_variant | LOW |  | None |  | None | 0.159090909 |
| chr1 | 45289209 | 45289210 | C | T | PTCH2 |  | ENST00000447098 | 0 | None | intron_variant | LOW |  | None |  | None | 0.166666667 |
| chr1 | 46187811 | 46187812 | A | G | IPP |  | ENST00000359942 | 0 | None | intron_variant | LOW |  | None |  | None | 0.2 |
| chr1 | 48766760 | 48766761 | A | G | SPATA6 |  | ENST00000396199 | 0 | None | intron_variant | LOW |  | None |  | None | 0.101694915 |
| chr1 | 48866626 | 48866627 | T | G | SPATA6 |  | ENST00000371847 | 0 | None | intron_variant | LOW |  | None |  | None | 0.189655172 |
| chr1 | 49333331 | 49333332 | G | T | AGBL4 |  | ENST00000416121 | 0 | None | intron_variant | LOW |  | None |  | None | 0.219512195 |
| chr1 | 50089677 | 50089678 | T | A | AGBL4 |  | ENST00000371838 | 0 | None | intron_variant | LOW |  | None |  | None | 0.194444444 |
| chr1 | 50335772 | 50335773 | A | T | AGBL4 |  | ENST00000371838 | 0 | None | intron_variant | LOW |  | None |  | None | 0.238095238 |
| chr1 | 50337226 | 50337227 | T | C | AGBL4 |  | ENST00000371839 | 0 | None | intron_variant | LOW |  | None |  | None | 0.230769231 |
| chr1 | 50666299 | 50666300 | C | T | ELAVL4 |  | ENST00000371827 | 0 | None | intron_variant | LOW |  | None |  | None | 0.271186441 |
| chr1 | 50684084 | 50684085 | C | G | RP11-567C20.3 |  | ENST00000440897 | 0 | None | intron_variant | LOW |  | None |  | None | 0.347826087 |
| chr1 | 51775347 | 51775348 | A | C | TTC39A |  | ENST00000371750 | 0 | None | intron_variant | LOW |  | None |  | None | 0.392156863 |
| chr1 | 52497257 | 52497258 | A | G | TXNDC12 |  | ENST00000371626 | 0 | None | intron_variant | LOW |  | None |  | None | 0.173913043 |
| chr1 | 52497274 | 52497275 | G | C | TXNDC12 |  | ENST00000371626 | 0 | None | intron_variant | LOW |  | None |  | None | 0.145833333 |
| chr1 | 52979120 | 52979121 | T | A | ZCCHC11 |  | ENST00000257177 | 0 | None | intron_variant | LOW |  | None |  | None | 0.196078431 |
| chr1 | 54277010 | 54277011 | T | C | NDC1 |  | ENST00000371429 | 0 | None | intron_variant | LOW |  | None |  | None | 0.283018868 |
| chr1 | 56509501 | 56509502 | G | T | None |  |  | 0 | None | intergenic_variant | LOW |  | None |  | None | 0.307692308 |
| chr1 | 56785630 | 56785631 | A | G | None |  |  | 0 | None | intergenic_variant | LOW |  | None |  | None | 0.333333333 |
| chr1 | 59498895 | 59498896 | A | T | RP4-794H19.4 |  | ENST00000438195 | 0 | None | intron_variant | LOW |  | None |  | None | 0.246376812 |
| chr1 | 60446252 | 60446253 | C | T | None |  |  | 0 | None | intergenic_variant | LOW |  | None |  | None | 0.205882353 |
| chr1 | 61751274 | 61751275 | G | A | NFIA |  | ENST00000371189 | 0 | None | intron_variant | LOW |  | None |  | None | 0.354166667 |
| chr1 | 62110349 | 62110350 | A | T | None |  |  | 0 | None | intergenic_variant | LOW |  | None |  | None | 0.22 |
| chr1 | 62483203 | 62483204 | C | G | INADL |  | ENST00000543708 | 0 | None | intron_variant | LOW |  | None |  | None | 0.2 |
| chr1 | 63511589 | 63511590 | C | T | None |  |  | 0 | None | intergenic_variant | LOW |  | None |  | None | 0.114285714 |
| chr1 | 63669868 | 63669869 | C | A | LINC00466 |  | ENST00000455304 | 0 | None | intron_variant | LOW |  | None |  | None | 0.25 |
| chr1 | 64251024 | 64251025 | T | G | ROR1 |  | ENST00000371079 | 0 | None | intron_variant | LOW |  | None |  | None | 0.216666667 |
| chr1 | 64607367 | 64607368 | T | G | ROR1 |  | ENST00000371080 | 0 | None | intron_variant | LOW |  | None |  | None | 0.180327869 |
| chr1 | 66881898 | 66881899 | G | T | None |  |  | 0 | None | intergenic_variant | LOW |  | None |  | None | 0.2 |
| chr1 | 67465968 | 67465969 | C | G | SLC35D1 |  | ENST00000235345 | 0 | None | 3_prime_UTR_variant | LOW |  | None |  | None | 0.133333333 |
| chr1 | 70926554 | 70926555 | G | A | None |  |  | 0 | None | intergenic_variant | LOW |  | None |  | None | 0.176470588 |
| chr1 | 73880990 | 73880991 | A | C | None |  |  | 0 | None | intergenic_variant | LOW |  | None |  | None | 0.212765957 |
| chr1 | 73925034 | 73925035 | G | C | None |  |  | 0 | None | intergenic_variant | LOW |  | None |  | None | 0.1875 |
| chr1 | 74853705 | 74853706 | A | G | TNNI3K |  | ENST00000370891 | 0 | None | intron_variant | LOW |  | None |  | None | 0.294117647 |
| chr1 | 75768805 | 75768806 | T | A | SLC44A5 |  | ENST00000370855 | 0 | None | intron_variant | LOW |  | None |  | None | 0.264150943 |
| chr1 | 75962853 | 75962854 | C | T | SLC44A5 |  | ENST00000370855 | 1 | None | intron_variant | LOW |  | None |  | None | 0.209302326 |
| chr1 | 77312724 | 77312725 | G | C | None |  |  | 0 | None | intergenic_variant | LOW |  | None |  | None | 0.278688525 |
| chr1 | 79316602 | 79316603 | C | T | None |  |  | 0 | None | intergenic_variant | LOW |  | None |  | None | 0.244444444 |
| chr1 | 79612842 | 79612843 | C | A | None |  |  | 0 | None | intergenic_variant | LOW |  | None |  | None | 0.224489796 |
| chr1 | 79645723 | 79645724 | G | A | None |  |  | 1 | None | intergenic_variant | LOW |  | None |  | None | 0.195652174 |
| chr1 | 80407909 | 80407910 | T | C | None |  |  | 0 | None | intergenic_variant | LOW |  | None |  | None | 0.186046512 |
| chr1 | 81117274 | 81117275 | C | A | RP5-887A10.1 |  | ENST00000418041 | 0 | None | downstream_gene_variant | LOW |  | None |  | None | 0.306122449 |
| chr1 | 81870932 | 81870933 | A | G | LPHN2 |  | ENST00000370725 | 1 | None | intron_variant | LOW |  | None |  | None | 0.260869565 |
| chr1 | 84248470 | 84248471 | C | A | RP11-475O6.1 |  | ENST00000417975 | 0 | None | intron_variant | LOW |  | None |  | None | 0.179487179 |
| chr1 | 86479172 | 86479173 | G | A | COL24A1 |  | ENST00000436319 | 0 | None | intron_variant | LOW |  | None |  | None | 0.3125 |
| chr1 | 86537276 | 86537277 | G | A | COL24A1 |  | ENST00000370571 | 0 | None | intron_variant | LOW |  | None |  | None | 0.289473684 |
| chr1 | 88362416 | 88362417 | G | T | RP5-1027O11.1 |  | ENST00000437598 | 0 | None | downstream_gene_variant | LOW |  | None |  | None | 0.195121951 |
| chr1 | 94355479 | 94355480 | T | A | GCLM |  | ENST00000370238 | 0 | None | intron_variant | LOW |  | None |  | None | 0.29787234 |
| chr1 | 96579802 | 96579803 | C | A | None |  |  | 0 | None | intergenic_variant | LOW |  | None |  | None | 0.239130435 |
| chr1 | 97077646 | 97077647 | C | T | None |  |  | 1 | None | intergenic_variant | LOW |  | None |  | None | 0.227272727 |
| chr1 | 97645161 | 97645162 | G | A | DPYD |  | ENST00000370192 | 0 | None | intron_variant | LOW |  | None |  | None | 0.363636364 |
| chr1 | 98384122 | 98384123 | C | A | DPYD |  | ENST00000423006 | 0 | None | intron_variant | LOW |  | None |  | None | 0.195652174 |
| chr1 | 98871509 | 98871510 | G | A | None |  |  | 0 | None | intergenic_variant | LOW |  | None |  | None | 0.261904762 |
| chr1 | 101159495 | 101159496 | T | A | None |  |  | 0 | None | intergenic_variant | LOW |  | None |  | None | 0.212121212 |
| chr1 | 102067363 | 102067364 | G | A | None |  |  | 0 | None | intergenic_variant | LOW |  | None |  | None | 0.208333333 |
| chr1 | 102578654 | 102578655 | C | A | None |  |  | 0 | None | intergenic_variant | LOW |  | None |  | None | 0.163265306 |
| chr1 | 102608935 | 102608936 | A | G | None |  |  | 0 | None | intergenic_variant | LOW |  | None |  | None | 0.25 |
| chr1 | 103309073 | 103309074 | A | G | RP5-936J12.1 |  | ENST00000414418 | 0 | None | intron_variant | LOW |  | None |  | None | 0.368421053 |
| chr1 | 104757689 | 104757690 | A | T | None |  |  | 0 | None | intergenic_variant | LOW |  | None |  | None | 0.275 |
| chr1 | 105011019 | 105011020 | C | T | None |  |  | 1 | None | intergenic_variant | LOW |  | None |  | None | 0.184210526 |
| chr1 | 107760708 | 107760709 | A | G | NTNG1 |  | ENST00000370061 | 0 | None | intron_variant | LOW |  | None |  | None | 0.451612903 |
| chr1 | 108875151 | 108875152 | G | A | SLC25A24P1 |  | ENST00000411846 | 0 | None | intron_variant | LOW |  | None |  | None | 0.25 |
| chr1 | 111195226 | 111195227 | C | T | RP11-284N8.3 |  | ENST00000566942 | 0 | None | downstream_gene_variant | LOW |  | None |  | None | 0.145454545 |
| chr1 | 112791907 | 112791908 | G | A | RP5-965F6.2 |  | ENST00000427290 | 0 | None | intron_variant | LOW |  | None |  | None | 0.177777778 |
| chr1 | 115654622 | 115654623 | C | T | None |  |  | 0 | None | intergenic_variant | LOW |  | None |  | None | 0.196078431 |
| chr1 | 115828171 | 115828172 | A | C | NGF |  | ENST00000369512 | 0 | None | downstream_gene_variant | LOW |  | None |  | None | 0.178571429 |
| chr1 | 115840227 | 115840228 | C | T | NGF |  | ENST00000369512 | 0 | None | intron_variant | LOW |  | None |  | None | 0.097222222 |
| chr1 | 116810627 | 116810628 | C | T | None |  |  | 0 | None | intergenic_variant | LOW |  | None |  | None | 0.151515152 |
| chr1 | 117192040 | 117192041 | C | T | IGSF3 |  | ENST00000369483 | 0 | None | intron_variant | LOW |  | None |  | None | 0.283018868 |
| chr1 | 117221339 | 117221340 | C | A | None |  |  | 0 | None | intergenic_variant | LOW |  | None |  | None | 0.133333333 |
| chr1 | 117423458 | 117423459 | G | A | None |  |  | 0 | None | intergenic_variant | LOW |  | None |  | None | 0.177419355 |
| chr1 | 117496566 | 117496567 | C | T | PTGFRN |  | ENST00000393203 | 0 | None | intron_variant | LOW |  | None |  | None | 0.15 |
| chr1 | 117824751 | 117824752 | A | C | None |  |  | 0 | None | intergenic_variant | LOW |  | None |  | None | 0.326923077 |
| chr1 | 118766506 | 118766507 | T | A | None |  |  | 0 | None | intergenic_variant | LOW |  | None |  | None | 0.195652174 |
| chr1 | 119722335 | 119722336 | T | C | RP11-418J17.1 |  | ENST00000413531 | 0 | None | intron_variant | LOW |  | None |  | None | 0.128205128 |
| chr1 | 120484226 | 120484227 | A | C | NOTCH2 | V/G | ENST00000256646 | 0 | None | missense_variant | MED | probably_damaging | 1 | deleterious | 0 | 0.205128205 |
| chr1 | 120542357 | 120542358 | A | T | NOTCH2 |  | ENST00000602566 | 1 | None | intron_variant | LOW |  | None |  | None | 0.111111111 |
| chr1 | 120604179 | 120604180 | T | C | NOTCH2 |  | ENST00000256646 | 0 | None | intron_variant | LOW |  | None |  | None | 0.060606061 |
| chr1 | 120615549 | 120615550 | T | C | NOTCH2 |  | ENST00000256646 | 1 | None | upstream_gene_variant | LOW |  | None |  | None | 0.117647059 |
| chr1 | 121138180 | 121138181 | C | G | RP11-343N15.1 |  | ENST00000437515 | 1 | None | intron_variant | LOW |  | None |  | None | 0.114754098 |
| chr1 | 121372962 | 121372963 | T | A | None |  |  | 1 | None | intergenic_variant | LOW |  | None |  | None | 0.090909091 |
| chr1 | 121375645 | 121375646 | A | G | None |  |  | 0 | None | intergenic_variant | LOW |  | None |  | None | 0.085470085 |
| chr1 | 121382469 | 121382470 | G | A | None |  |  | 0 | None | intergenic_variant | LOW |  | None |  | None | 0.077844311 |
| chr1 | 142547719 | 142547720 | T | C | None |  |  | 1 | None | intergenic_variant | LOW |  | None |  | None | 0.076923077 |
| chr1 | 142564871 | 142564872 | G | A | RP11-417J8.2 |  | ENST00000415979 | 0 | None | downstream_gene_variant | LOW |  | None |  | None | 0.088983051 |
| chr1 | 142564882 | 142564883 | A | G | RP11-417J8.2 |  | ENST00000415979 | 0 | None | downstream_gene_variant | LOW |  | None |  | None | 0.077625571 |
| chr1 | 142564919 | 142564920 | A | G | RP11-417J8.2 |  | ENST00000415979 | 1 | None | downstream_gene_variant | LOW |  | None |  | None | 0.115577889 |
| chr1 | 142644737 | 142644738 | T | G | RP11-417J8.6 |  | ENST00000369381 | 0 | None | intron_variant | LOW |  | None |  | None | 0.049261084 |
| chr1 | 142644760 | 142644761 | G | A | RP11-417J8.3 |  | ENST00000426408 | 0 | None | intron_variant | LOW |  | None |  | None | 0.056074766 |
| chr1 | 142675909 | 142675910 | G | A | RP11-417J8.3 |  | ENST00000400755 | 1 | None | intron_variant | LOW |  | None |  | None | 0.055248619 |
| chr1 | 142675928 | 142675929 | C | T | RP11-417J8.6 |  | ENST00000610091 | 0 | None | intron_variant | LOW |  | None |  | None | 0.04519774 |
| chr1 | 142675936 | 142675937 | G | A | RP11-417J8.6 |  | ENST00000610091 | 0 | None | intron_variant | LOW |  | None |  | None | 0.045977011 |
| chr1 | 142794838 | 142794839 | C | T | RP11-423O2.2 |  | ENST00000433591 | 1 | None | upstream_gene_variant | LOW |  | None |  | None | 0.113636364 |
| chr1 | 142801037 | 142801038 | G | T | RP11-423O2.5 |  | ENST00000423385 | 1 | None | downstream_gene_variant | LOW |  | None |  | None | 0.126984127 |
| chr1 | 142899644 | 142899645 | G | T | None |  |  | 0 | None | intergenic_variant | LOW |  | None |  | None | 0.045226131 |
| chr1 | 142899645 | 142899646 | G | T | None |  |  | 0 | None | intergenic_variant | LOW |  | None |  | None | 0.045 |
| chr1 | 142904776 | 142904777 | G | T | None |  |  | 1 | None | intergenic_variant | LOW |  | None |  | None | 0.058252427 |
| chr1 | 142955923 | 142955924 | A | T | RP11-423O2.7 |  | ENST00000424640 | 1 | None | intron_variant | LOW |  | None |  | None | 0.058441558 |
| chr1 | 143134577 | 143134578 | G | C | RP11-782C8.1 |  | ENST00000428930 | 0 | None | intron_variant | LOW |  | None |  | None | 0.119266055 |
| chr1 | 143176602 | 143176603 | A | T | RP11-782C8.2 |  | ENST00000422129 | 0 | None | intron_variant | LOW |  | None |  | None | 0.08974359 |
| chr1 | 143178727 | 143178728 | C | T | RP11-782C8.1 |  | ENST00000428930 | 1 | None | intron_variant | LOW |  | None |  | None | 0.087248322 |
| chr1 | 143179749 | 143179750 | A | G | RP11-782C8.1 |  | ENST00000447407 | 1 | None | intron_variant | LOW |  | None |  | None | 0.098214286 |
| chr1 | 143199646 | 143199647 | T | C | RP11-782C8.1 |  | ENST00000428930 | 1 | None | intron_variant | LOW |  | None |  | None | 0.068027211 |
| chr1 | 143204548 | 143204549 | G | C | RP11-782C8.1 |  | ENST00000428930 | 0 | None | intron_variant | LOW |  | None |  | None | 0.098039216 |
| chr1 | 143222970 | 143222971 | C | T | RP11-782C8.1 |  | ENST00000438000 | 0 | None | intron_variant | LOW |  | None |  | None | 0.087719298 |
| chr1 | 143222971 | 143222972 | T | G | RP11-782C8.1 |  | ENST00000428930 | 0 | None | intron_variant | LOW |  | None |  | None | 0.088495575 |
| chr1 | 143232566 | 143232567 | A | G | RP11-782C8.5 |  | ENST00000422716 | 0 | None | intron_variant | LOW |  | None |  | None | 0.156862745 |
| chr1 | 143232570 | 143232571 | C | T | RP11-782C8.5 |  | ENST00000422716 | 0 | None | intron_variant | LOW |  | None |  | None | 0.16 |
| chr1 | 143243241 | 143243242 | C | T | RP11-782C8.5 |  | ENST00000429121 | 1 | None | upstream_gene_variant | LOW |  | None |  | None | 0.12 |
| chr1 | 143243241 | 143243242 | C | A | RP11-782C8.5 |  | ENST00000422716 | 1 | None | upstream_gene_variant | LOW |  | None |  | None | 0.22 |
| chr1 | 143256906 | 143256907 | G | A | None |  |  | 0 | None | intergenic_variant | LOW |  | None |  | None | 0.070866142 |
| chr1 | 143258725 | 143258726 | A | C | None |  |  | 0 | None | intergenic_variant | LOW |  | None |  | None | 0.078651685 |
| chr1 | 143258761 | 143258762 | C | G | None |  |  | 1 | None | intergenic_variant | LOW |  | None |  | None | 0.105882353 |
| chr1 | 143265932 | 143265933 | C | A | None |  |  | 0 | None | intergenic_variant | LOW |  | None |  | None | 0.088235294 |
| chr1 | 143359273 | 143359274 | A | G | RP11-435B5.4 |  | ENST00000424068 | 1 | None | intron_variant | LOW |  | None |  | None | 0.125 |
| chr1 | 143375866 | 143375867 | T | C | RP11-435B5.3 |  | ENST00000430699 | 1 | None | intron_variant | LOW |  | None |  | None | 0.075471698 |
| chr1 | 143417668 | 143417669 | G | A | RP11-435B5.4 |  | ENST00000443548 | 0 | None | intron_variant | LOW |  | None |  | None | 0.06 |
| chr1 | 143476875 | 143476876 | T | C | None |  |  | 1 | None | intergenic_variant | LOW |  | None |  | None | 0.075757576 |
| chr1 | 143525177 | 143525178 | A | T | RP11-435B5.7 |  | ENST00000418607 | 0 | None | downstream_gene_variant | LOW |  | None |  | None | 0.054054054 |
| chr1 | 143529967 | 143529968 | A | T | RP11-435B5.7 |  | ENST00000418607 | 0 | None | intron_variant | LOW |  | None |  | None | 0.0625 |
| chr1 | 144051857 | 144051858 | T | C | SRGAP2B |  | ENST00000467933 | 1 | None | intron_variant | LOW |  | None |  | None | 0.118181818 |
| chr1 | 144061817 | 144061818 | G | A | SRGAP2B |  | ENST00000467933 | 0 | None | intron_variant | LOW |  | None |  | None | 0.088607595 |
| chr1 | 144590911 | 144590912 | C | T | RP11-640M9.2 |  | ENST00000428365 | 0 | None | upstream_gene_variant | LOW |  | None |  | None | 0.082089552 |
| chr1 | 144590954 | 144590955 | C | T | RP11-640M9.2 |  | ENST00000421407 | 0 | None | upstream_gene_variant | LOW |  | None |  | None | 0.059701493 |
| chr1 | 144599185 | 144599186 | C | T | RP11-640M9.2 |  | ENST00000428365 | 1 | None | intron_variant | LOW |  | None |  | None | 0.164705882 |
| chr1 | 144619465 | 144619466 | A | G | RP11-640M9.2 |  | ENST00000446541 | 1 | None | intron_variant | LOW |  | None |  | None | 0.08988764 |
| chr1 | 144702196 | 144702197 | A | G | None |  |  | 0 | None | intergenic_variant | LOW |  | None |  | None | 0.127272727 |
| chr1 | 144812776 | 144812777 | G | T | NBPF9 |  | ENST00000281815 | 0 | None | intron_variant | LOW |  | None |  | None | 0.03 |
| chr1 | 144812776 | 144812777 | G | C | NBPF9 |  | ENST00000440491 | 1 | None | intron_variant | LOW |  | None |  | None | 0.10 |
| chr1 | 145311566 | 145311567 | T | A | NBPF10 |  | ENST00000369338 | 0 | None | intron_variant | LOW |  | None |  | None | 0.076923077 |
| chr1 | 145379323 | 145379324 | C | A | RP11-458D21.1 |  | ENST00000419817 | 1 | None | intron_variant | LOW |  | None |  | None | 0.19 |
| chr1 | 145379323 | 145379324 | C | T | RP11-458D21.1 |  | ENST00000610154 | 1 | None | intron_variant | LOW |  | None |  | None | 0.24 |
| chr1 | 146472486 | 146472487 | G | A | NBPF12 |  | ENST00000442909 | 1 | None | downstream_gene_variant | LOW |  | None |  | None | 0.159574468 |
| chr1 | 146487900 | 146487901 | C | G | NBPF13P |  | ENST00000444680 | 1 | None | downstream_gene_variant | LOW |  | None |  | None | 0.127272727 |
| chr1 | 146823372 | 146823373 | A | G | None |  |  | 0 | None | intergenic_variant | LOW |  | None |  | None | 0.134615385 |
| chr1 | 146840321 | 146840322 | G | A | None |  |  | 0 | None | intergenic_variant | LOW |  | None |  | None | 0.145454545 |
| chr1 | 148192334 | 148192335 | C | T | RP11-289I10.2 |  | ENST00000545938 | 1 | None | upstream_gene_variant | LOW |  | None |  | None | 0.215384615 |
| chr1 | 148530811 | 148530812 | C | T | None |  |  | 0 | None | intergenic_variant | LOW |  | None |  | None | 0.120689655 |
| chr1 | 148569201 | 148569202 | A | G | NBPF15 |  | ENST00000442702 | 1 | None | intron_variant | LOW |  | None |  | None | 0.134831461 |
| chr1 | 148829503 | 148829504 | C | A | None |  |  | 0 | None | intergenic_variant | LOW |  | None |  | None | 0.129310345 |
| chr1 | 149022215 | 149022216 | C | A | None |  |  | 1 | None | intergenic_variant | LOW |  | None |  | None | 0.106796117 |
| chr1 | 149027737 | 149027738 | A | T | None |  |  | 0 | None | intergenic_variant | LOW |  | None |  | None | 0.057471264 |
| chr1 | 149029995 | 149029996 | T | A | None |  |  | 0 | None | intergenic_variant | LOW |  | None |  | None | 0.070866142 |
| chr1 | 150451583 | 150451584 | A | G | RPRD2 |  | ENST00000401000 | 0 | None | downstream_gene_variant | LOW |  | None |  | None | 0.161290323 |
| chr1 | 150842885 | 150842886 | T | A | ARNT |  | ENST00000354396 | 0 | None | intron_variant | LOW |  | None |  | None | 0.24137931 |
| chr1 | 150921046 | 150921047 | G | A | SETDB1 |  | ENST00000368969 | 0 | None | intron_variant | LOW |  | None |  | None | 0.315789474 |
| chr1 | 150950732 | 150950733 | C | A | CERS2 |  | ENST00000271688 | 0 | None | upstream_gene_variant | LOW |  | None |  | None | 0.137931034 |
| chr1 | 151018288 | 151018289 | C | T | BNIPL | R/* | ENST00000368931 | 0 | None | stop_gained | HIGH |  | None |  | None | 0.174603175 |
| chr1 | 152548516 | 152548517 | C | T | LCE3D |  | ENST00000368787 | 0 | None | downstream_gene_variant | LOW |  | None |  | None | 0.22 |
| chr1 | 153313167 | 153313168 | G | T | PGLYRP4 |  | ENST00000359650 | 0 | None | intron_variant | LOW |  | None |  | None | 0.230769231 |
| chr1 | 153712600 | 153712601 | G | A | INTS3 |  | ENST00000435409 | 0 | None | intron_variant | LOW |  | None |  | None | 0.15 |
| chr1 | 154683099 | 154683100 | G | A | KCNN3 |  | ENST00000361147 | 1 | None | intron_variant | LOW |  | None |  | None | 0.147058824 |
| chr1 | 155841018 | 155841019 | G | C | SYT11 |  | ENST00000368324 | 0 | None | intron_variant | LOW |  | None |  | None | 0.344262295 |
| chr1 | 155876569 | 155876570 | A | G | RIT1 |  | ENST00000539040 | 1 | None | intron_variant | LOW |  | None |  | None | 0.210526316 |
| chr1 | 156432905 | 156432906 | A | G | MEF2D |  | ENST00000360595 | 0 | None | downstream_gene_variant | LOW |  | None |  | None | 0.12962963 |
| chr1 | 157237371 | 157237372 | G | A | None |  |  | 0 | None | intergenic_variant | LOW |  | None |  | None | 0.258064516 |
| chr1 | 157765479 | 157765480 | A | T | FCRL1 |  | ENST00000358292 | 0 | None | 3_prime_UTR_variant | LOW |  | None |  | None | 0.229508197 |
| chr1 | 158061128 | 158061129 | C | A | KIRREL |  | ENST00000416935 | 0 | None | intron_variant | LOW |  | None |  | None | 0.186440678 |
| chr1 | 158100209 | 158100210 | C | G | RP11-404O13.1 |  | ENST00000414848 | 0 | None | downstream_gene_variant | LOW |  | None |  | None | 0.185714286 |
| chr1 | 158122000 | 158122001 | T | A | None |  |  | 0 | None | intergenic_variant | LOW |  | None |  | None | 0.195652174 |
| chr1 | 158165938 | 158165939 | G | T | RP11-404O13.5 |  | ENST00000415019 | 0 | None | downstream_gene_variant | LOW |  | None |  | None | 0.148148148 |
| chr1 | 158442102 | 158442103 | C | G | RP11-144L1.4 |  | ENST00000419738 | 0 | None | downstream_gene_variant | LOW |  | None |  | None | 0.212765957 |
| chr1 | 158513720 | 158513721 | G | A | OR6Y1 |  | ENST00000302617 | 0 | None | downstream_gene_variant | LOW |  | None |  | None | 0.152173913 |
| chr1 | 158576824 | 158576825 | C | G | OR10Z1 | I/M | ENST00000361284 | 0 | None | missense_variant | MED | possibly_damaging | 0.511 | deleterious | 0.02 | 0.240740741 |
| chr1 | 158696305 | 158696306 | G | A | OR6K4P |  | ENST00000423179 | 0 | None | upstream_gene_variant | LOW |  | None |  | None | 0.169811321 |
| chr1 | 158785029 | 158785030 | G | A | None |  |  | 0 | None | intergenic_variant | LOW |  | None |  | None | 0.26984127 |
| chr1 | 159228693 | 159228694 | A | T | None |  |  | 0 | None | intergenic_variant | LOW |  | None |  | None | 0.191489362 |
| chr1 | 159670105 | 159670106 | C | G | CRPP1 |  | ENST00000412857 | 0 | None | downstream_gene_variant | LOW |  | None |  | None | 0.204081633 |
| chr1 | 160611282 | 160611283 | C | T | SLAMF1 |  | ENST00000235739 | 0 | None | intron_variant | LOW |  | None |  | None | 0.186440678 |
| chr1 | 161215400 | 161215401 | G | T | None |  |  | 0 | None | intergenic_variant | LOW |  | None |  | None | 0.223880597 |
| chr1 | 163410636 | 163410637 | A | T | None |  |  | 0 | None | intergenic_variant | LOW |  | None |  | None | 0.125 |
| chr1 | 163891812 | 163891813 | A | G | U3 |  | ENST00000391236 | 0 | None | upstream_gene_variant | LOW |  | None |  | None | 0.230769231 |
| chr1 | 164055397 | 164055398 | T | A | None |  |  | 1 | None | intergenic_variant | LOW |  | None |  | None | 0.132075472 |
| chr1 | 165002308 | 165002309 | G | T | None |  |  | 0 | None | intergenic_variant | LOW |  | None |  | None | 0.1875 |
| chr1 | 166219027 | 166219028 | C | A | None |  |  | 0 | None | intergenic_variant | LOW |  | None |  | None | 0.1 |
| chr1 | 166465871 | 166465872 | A | G | None |  |  | 0 | None | intergenic_variant | LOW |  | None |  | None | 0.226415094 |
| chr1 | 166588538 | 166588539 | G | T | FMO9P |  | ENST00000477875 | 0 | None | intron_variant | LOW |  | None |  | None | 0.254901961 |
| chr1 | 167061089 | 167061090 | G | C | DUSP27 |  | ENST00000361200 | 0 | None | upstream_gene_variant | LOW |  | None |  | None | 0.169014085 |
| chr1 | 167404571 | 167404572 | T | C | CD247 |  | ENST00000362089 | 1 | None | intron_variant | LOW |  | None |  | None | 0.235294118 |
| chr1 | 168532467 | 168532468 | G | A | None |  |  | 0 | None | intergenic_variant | LOW |  | None |  | None | 0.2 |
| chr1 | 168616220 | 168616221 | C | T | None |  |  | 0 | None | intergenic_variant | LOW |  | None |  | None | 0.116666667 |
| chr1 | 169058963 | 169058964 | A | G | LINC00970 |  | ENST00000366408 | 1 | None | upstream_gene_variant | LOW |  | None |  | None | 0.06 |
| chr1 | 169191248 | 169191249 | A | G | NME7 |  | ENST00000472647 | 0 | None | intron_variant | LOW |  | None |  | None | 0.170731707 |
| chr1 | 170030391 | 170030392 | G | T | KIFAP3 |  | ENST00000367767 | 0 | None | intron_variant | LOW |  | None |  | None | 0.245901639 |
| chr1 | 170172916 | 170172917 | A | G | RP11-297H3.3 |  | ENST00000439184 | 0 | None | intron_variant | LOW |  | None |  | None | 0.4 |
| chr1 | 170716942 | 170716943 | G | A | None |  |  | 0 | None | intergenic_variant | LOW |  | None |  | None | 0.210526316 |
| chr1 | 170719207 | 170719208 | G | T | None |  |  | 0 | None | intergenic_variant | LOW |  | None |  | None | 0.28125 |
| chr1 | 170751693 | 170751694 | G | T | None |  |  | 0 | None | intergenic_variant | LOW |  | None |  | None | 0.255319149 |
| chr1 | 170943894 | 170943895 | G | T | MROH9 |  | ENST00000367759 | 0 | None | intron_variant | LOW |  | None |  | None | 0.228070175 |
| chr1 | 171716793 | 171716794 | G | T | RP4-560B9.5 |  | ENST00000413827 | 0 | None | downstream_gene_variant | LOW |  | None |  | None | 0.169811321 |
| chr1 | 172438401 | 172438402 | C | T | C1orf105 |  | ENST00000367725 | 0 | None | downstream_gene_variant | LOW |  | None |  | None | 0.171875 |
| chr1 | 173294178 | 173294179 | C | T | None |  |  | 1 | None | intergenic_variant | LOW |  | None |  | None | 0.116666667 |
| chr1 | 174263502 | 174263503 | G | T | RABGAP1L |  | ENST00000367689 | 0 | None | intron_variant | LOW |  | None |  | None | 0.170212766 |
| chr1 | 174914603 | 174914604 | G | A | RABGAP1L |  | ENST00000392064 | 0 | None | intron_variant | LOW |  | None |  | None | 0.155172414 |
| chr1 | 175260958 | 175260959 | T | A | None |  |  | 0 | None | intergenic_variant | LOW |  | None |  | None | 0.105263158 |
| chr1 | 175336978 | 175336979 | A | G | TNR |  | ENST00000263525 | 0 | None | intron_variant | LOW |  | None |  | None | 0.186440678 |
| chr1 | 175438206 | 175438207 | T | C | TNR |  | ENST00000367674 | 0 | None | intron_variant | LOW |  | None |  | None | 0.226415094 |
| chr1 | 175778609 | 175778610 | T | A | None |  |  | 0 | None | intergenic_variant | LOW |  | None |  | None | 0.259259259 |
| chr1 | 176302394 | 176302395 | A | G | None |  |  | 0 | None | intergenic_variant | LOW |  | None |  | None | 0.291666667 |
| chr1 | 176610034 | 176610035 | G | A | PAPPA2 |  | ENST00000367662 | 1 | None | intron_variant | LOW |  | None |  | None | 0.243902439 |
| chr1 | 176799975 | 176799976 | C | A | PAPPA2 |  | ENST00000367662 | 0 | None | intron_variant | LOW |  | None |  | None | 0.253521127 |
| chr1 | 176943430 | 176943431 | T | A | ASTN1 |  | ENST00000367657 | 0 | None | intron_variant | LOW |  | None |  | None | 0.192982456 |
| chr1 | 176997865 | 176997866 | G | A | ASTN1 |  | ENST00000367657 | 0 | None | intron_variant | LOW |  | None |  | None | 0.245901639 |
| chr1 | 178430105 | 178430106 | G | A | RASAL2 |  | ENST00000448150 | 0 | None | intron_variant | LOW |  | None |  | None | 0.280701754 |
| chr1 | 178568126 | 178568127 | G | T | None |  |  | 0 | None | intergenic_variant | LOW |  | None |  | None | 0.314814815 |
| chr1 | 180156309 | 180156310 | C | T | QSOX1 |  | ENST00000367602 | 0 | None | intron_variant | LOW |  | None |  | None | 0.180555556 |
| chr1 | 180609230 | 180609231 | A | T | XPR1 |  | ENST00000367589 | 0 | None | intron_variant | LOW |  | None |  | None | 0.163934426 |
| chr1 | 180680324 | 180680325 | G | A | XPR1 |  | ENST00000367590 | 0 | None | intron_variant | LOW |  | None |  | None | 0.109756098 |
| chr1 | 181122074 | 181122075 | T | C | None |  |  | 0 | None | intergenic_variant | LOW |  | None |  | None | 0.204081633 |
| chr1 | 182347916 | 182347917 | A | T | GLUL |  | ENST00000331872 | 0 | None | downstream_gene_variant | LOW |  | None |  | None | 0.260869565 |
| chr1 | 185275680 | 185275681 | T | C | IVNS1ABP |  | ENST00000392007 | 0 | None | 5_prime_UTR_variant | LOW |  | None |  | None | 0.173913043 |
| chr1 | 185835780 | 185835781 | G | A | HMCN1 |  | ENST00000367492 | 0 | None | intron_variant | LOW |  | None |  | None | 0.181818182 |
| chr1 | 186619511 | 186619512 | C | T | None |  |  | 0 | None | intergenic_variant | LOW |  | None |  | None | 0.15 |
| chr1 | 187982243 | 187982244 | A | G | None |  |  | 0 | None | intergenic_variant | LOW |  | None |  | None | 0.274193548 |
| chr1 | 188572765 | 188572766 | A | G | None |  |  | 0 | None | intergenic_variant | LOW |  | None |  | None | 0.159090909 |
| chr1 | 188948809 | 188948810 | T | G | LINC01035 |  | ENST00000445072 | 0 | None | intron_variant | LOW |  | None |  | None | 0.181818182 |
| chr1 | 189275688 | 189275689 | A | G | None |  |  | 0 | None | intergenic_variant | LOW |  | None |  | None | 0.357142857 |
| chr1 | 189480954 | 189480955 | T | A | None |  |  | 0 | None | intergenic_variant | LOW |  | None |  | None | 0.224489796 |
| chr1 | 189767141 | 189767142 | C | T | RP11-398M15.1 |  | ENST00000419614 | 0 | None | intron_variant | LOW |  | None |  | None | 0.192982456 |
| chr1 | 189905626 | 189905627 | A | T | None |  |  | 0 | None | intergenic_variant | LOW |  | None |  | None | 0.269230769 |
| chr1 | 189905630 | 189905631 | A | G | None |  |  | 0 | None | intergenic_variant | LOW |  | None |  | None | 0.254901961 |
| chr1 | 190966180 | 190966181 | G | C | None |  |  | 0 | None | intergenic_variant | LOW |  | None |  | None | 0.169811321 |
| chr1 | 191153362 | 191153363 | G | A | RP11-309H21.4 |  | ENST00000603579 | 0 | None | upstream_gene_variant | LOW |  | None |  | None | 0.245614035 |
| chr1 | 191697993 | 191697994 | G | A | None |  |  | 0 | None | intergenic_variant | LOW |  | None |  | None | 0.138461538 |
| chr1 | 191937343 | 191937344 | A | C | RP11-541F9.2 |  | ENST00000430776 | 0 | None | intron_variant | LOW |  | None |  | None | 0.14893617 |
| chr1 | 192004355 | 192004356 | T | A | None |  |  | 0 | None | intergenic_variant | LOW |  | None |  | None | 0.131578947 |
| chr1 | 192068676 | 192068677 | A | T | None |  |  | 0 | None | intergenic_variant | LOW |  | None |  | None | 0.234042553 |
| chr1 | 192126476 | 192126477 | G | T | RGS18 |  | ENST00000367460 | 0 | None | upstream_gene_variant | LOW |  | None |  | None | 0.244897959 |
| chr1 | 192690704 | 192690705 | T | A | MIR4426 |  | ENST00000443181 | 0 | None | downstream_gene_variant | LOW |  | None |  | None | 0.185185185 |
| chr1 | 193718306 | 193718307 | C | T | None |  |  | 0 | None | intergenic_variant | LOW |  | None |  | None | 0.217391304 |
| chr1 | 193918490 | 193918491 | C | G | None |  |  | 0 | None | intergenic_variant | LOW |  | None |  | None | 0.352941176 |
| chr1 | 194503806 | 194503807 | C | T | None |  |  | 0 | None | intergenic_variant | LOW |  | None |  | None | 0.137931034 |
| chr1 | 194571382 | 194571383 | T | C | None |  |  | 0 | None | intergenic_variant | LOW |  | None |  | None | 0.25 |
| chr1 | 194711492 | 194711493 | A | T | None |  |  | 0 | None | intergenic_variant | LOW |  | None |  | None | 0.263157895 |
| chr1 | 194757044 | 194757045 | G | T | None |  |  | 0 | None | intergenic_variant | LOW |  | None |  | None | 0.352941176 |
| chr1 | 194886920 | 194886921 | G | T | None |  |  | 0 | None | intergenic_variant | LOW |  | None |  | None | 0.163265306 |
| chr1 | 194908124 | 194908125 | T | A | None |  |  | 0 | None | intergenic_variant | LOW |  | None |  | None | 0.162162162 |
| chr1 | 195450931 | 195450932 | C | A | None |  |  | 0 | None | intergenic_variant | LOW |  | None |  | None | 0.305084746 |
| chr1 | 195882691 | 195882692 | G | A | None |  |  | 0 | None | intergenic_variant | LOW |  | None |  | None | 0.203389831 |
| chr1 | 196470490 | 196470491 | A | T | KCNT2 |  | ENST00000367431 | 0 | None | intron_variant | LOW |  | None |  | None | 0.28 |
| chr1 | 196542098 | 196542099 | G | T | KCNT2 |  | ENST00000294725 | 0 | None | intron_variant | LOW |  | None |  | None | 0.192307692 |
| chr1 | 196880762 | 196880763 | A | G | CFHR4 |  | ENST00000367418 | 0 | None | intron_variant | LOW |  | None |  | None | 0.214285714 |
| chr1 | 196909411 | 196909412 | G | A | CFHR2 |  | ENST00000367421 | 0 | None | intron_variant | LOW |  | None |  | None | 0.157894737 |
| chr1 | 197197562 | 197197563 | G | T | CRB1 |  | ENST00000535699 | 0 | None | intron_variant | LOW |  | None |  | None | 0.285714286 |
| chr1 | 197978230 | 197978231 | T | A | None |  |  | 0 | None | intergenic_variant | LOW |  | None |  | None | 0.210526316 |
| chr1 | 198219449 | 198219450 | T | G | NEK7 |  | ENST00000391974 | 0 | None | intron_variant | LOW |  | None |  | None | 0.112676056 |
| chr1 | 198845848 | 198845849 | T | G | MIR181A1HG |  | ENST00000432296 | 0 | None | intron_variant | LOW |  | None |  | None | 0.142857143 |
| chr1 | 199043527 | 199043528 | A | T | RP11-16L9.4 |  | ENST00000432488 | 0 | None | intron_variant | LOW |  | None |  | None | 0.235294118 |
| chr1 | 199078415 | 199078416 | T | A | None |  |  | 0 | None | intergenic_variant | LOW |  | None |  | None | 0.283018868 |
| chr1 | 199170517 | 199170518 | C | A | RP11-382E9.1 |  | ENST00000452199 | 0 | None | intron_variant | LOW |  | None |  | None | 0.130434783 |
| chr1 | 199865906 | 199865907 | A | T | None |  |  | 0 | None | intergenic_variant | LOW |  | None |  | None | 0.134615385 |
| chr1 | 201535176 | 201535177 | C | T | None |  |  | 0 | None | intergenic_variant | LOW |  | None |  | None | 0.230769231 |
| chr1 | 202830506 | 202830507 | A | C | RP11-480I12.5 |  | ENST00000443294 | 0 | None | intron_variant | LOW |  | None |  | None | 0.216666667 |
| chr1 | 203251556 | 203251557 | C | T | RP11-134P9.3 |  | ENST00000412772 | 0 | None | upstream_gene_variant | LOW |  | None |  | None | 0.194444444 |
| chr1 | 203732984 | 203732985 | A | T | LAX1 |  | ENST00000367217 | 1 | None | upstream_gene_variant | LOW |  | None |  | None | 0.137931034 |
| chr1 | 204099935 | 204099936 | C | T | ETNK2 |  | ENST00000422699 | 0 | None | downstream_gene_variant | LOW |  | None |  | None | 0.228070175 |
| chr1 | 208052688 | 208052689 | T | G | CD34 |  | ENST00000485761 | 0 | None | downstream_gene_variant | LOW |  | None |  | None | 0.164179104 |
| chr1 | 209233995 | 209233996 | T | A | None |  |  | 0 | None | intergenic_variant | LOW |  | None |  | None | 0.322580645 |
| chr1 | 209261285 | 209261286 | G | T | None |  |  | 0 | None | intergenic_variant | LOW |  | None |  | None | 0.155172414 |
| chr1 | 209566699 | 209566700 | T | C | None |  |  | 0 | None | intergenic_variant | LOW |  | None |  | None | 0.203703704 |
| chr1 | 210974333 | 210974334 | A | G | KCNH1 |  | ENST00000271751 | 0 | None | intron_variant | LOW |  | None |  | None | 0.2 |
| chr1 | 210981779 | 210981780 | A | C | KCNH1 |  | ENST00000367007 | 0 | None | intron_variant | LOW |  | None |  | None | 0.25 |
| chr1 | 211233767 | 211233768 | G | A | KCNH1 |  | ENST00000271751 | 0 | None | intron_variant | LOW |  | None |  | None | 0.162162162 |
| chr1 | 212566057 | 212566058 | G | A | TMEM206 |  | ENST00000261455 | 0 | None | intron_variant | LOW |  | None |  | None | 0.122807018 |
| chr1 | 213857610 | 213857611 | G | T | None |  |  | 0 | None | intergenic_variant | LOW |  | None |  | None | 0.153846154 |
| chr1 | 215043883 | 215043884 | T | C | GAPDHP24 |  | ENST00000328713 | 1 | None | upstream_gene_variant | LOW |  | None |  | None | 0.188679245 |
| chr1 | 215043924 | 215043925 | A | G | GAPDHP24 |  | ENST00000328713 | 1 | None | upstream_gene_variant | LOW |  | None |  | None | 0.180327869 |
| chr1 | 215179875 | 215179876 | T | A | KCNK2 |  | ENST00000391895 | 0 | None | intron_variant | LOW |  | None |  | None | 0.386363636 |
| chr1 | 215438182 | 215438183 | T | A | None |  |  | 1 | None | intergenic_variant | LOW |  | None |  | None | 0.172413793 |
| chr1 | 215457267 | 215457268 | A | C | None |  |  | 0 | None | intergenic_variant | LOW |  | None |  | None | 0.27027027 |
| chr1 | 216237753 | 216237754 | A | T | USH2A |  | ENST00000366943 | 0 | None | intron_variant | LOW |  | None |  | None | 0.30 |
| chr1 | 216237753 | 216237754 | A | G | USH2A |  | ENST00000366943 | 1 | None | intron_variant | LOW |  | None |  | None | 0.65 |
| chr1 | 216460916 | 216460917 | A | T | USH2A |  | ENST00000366942 | 0 | None | intron_variant | LOW |  | None |  | None | 0.166666667 |
| chr1 | 216821133 | 216821134 | T | C | ESRRG |  | ENST00000475275 | 0 | None | intron_variant | LOW |  | None |  | None | 0.262295082 |
| chr1 | 216823603 | 216823604 | A | T | ESRRG |  | ENST00000408911 | 0 | None | intron_variant | LOW |  | None |  | None | 0.183673469 |
| chr1 | 217431147 | 217431148 | G | A | None |  |  | 0 | None | intergenic_variant | LOW |  | None |  | None | 0.133333333 |
| chr1 | 218050892 | 218050893 | C | A | None |  |  | 0 | None | intergenic_variant | LOW |  | None |  | None | 0.108695652 |
| chr1 | 221330731 | 221330732 | C | T | None |  |  | 0 | None | intergenic_variant | LOW |  | None |  | None | 0.230769231 |
| chr1 | 223410190 | 223410191 | C | T | SUSD4 |  | ENST00000454695 | 0 | None | intron_variant | LOW |  | None |  | None | 0.234375 |
| chr1 | 223587833 | 223587834 | C | T | None |  |  | 0 | None | intergenic_variant | LOW |  | None |  | None | 0.204081633 |
| chr1 | 224203055 | 224203056 | G | A | RP11-504P24.4 |  | ENST00000540997 | 0 | None | downstream_gene_variant | LOW |  | None |  | None | 0.086956522 |
| chr1 | 224289445 | 224289446 | G | A | None |  |  | 1 | None | intergenic_variant | LOW |  | None |  | None | 0.184210526 |
| chr1 | 224838316 | 224838317 | C | T | CNIH3 |  | ENST00000272133 | 0 | None | intron_variant | LOW |  | None |  | None | 0.292307692 |
| chr1 | 225600411 | 225600412 | C | T | AC092811.1 |  | ENST00000366845 | 0 | None | 5_prime_UTR_variant | LOW |  | None |  | None | 0.125 |
| chr1 | 225915113 | 225915114 | G | A | RP11-145A3.2 |  | ENST00000428148 | 0 | None | intron_variant | LOW |  | None |  | None | 0.158730159 |
| chr1 | 226834557 | 226834558 | A | C | ITPKB |  | ENST00000429204 | 0 | None | intron_variant | LOW |  | None |  | None | 0.186440678 |
| chr1 | 227040157 | 227040158 | T | A | None |  |  | 0 | None | intergenic_variant | LOW |  | None |  | None | 0.160714286 |
| chr1 | 228091164 | 228091165 | A | G | None |  |  | 0 | None | intergenic_variant | LOW |  | None |  | None | 0.1 |
| chr1 | 228309559 | 228309560 | A | T | None |  |  | 1 | None | intergenic_variant | LOW |  | None |  | None | 0.203389831 |
| chr1 | 229085967 | 229085968 | C | G | None |  |  | 1 | None | intergenic_variant | LOW |  | None |  | None | 0.102941176 |
| chr1 | 229655716 | 229655717 | A | T | ABCB10 |  | ENST00000344517 | 0 | None | intron_variant | LOW |  | None |  | None | 0.255319149 |
| chr1 | 230242088 | 230242089 | C | G | GALNT2 |  | ENST00000543760 | 0 | None | intron_variant | LOW |  | None |  | None | 0.275862069 |
| chr1 | 230446464 | 230446465 | T | C | AL691479.1 |  | ENST00000366088 | 0 | None | downstream_gene_variant | LOW |  | None |  | None | 0.147540984 |
| chr1 | 231250119 | 231250120 | C | T | RP5-1097F14.1 |  | ENST00000435008 | 0 | None | downstream_gene_variant | LOW |  | None |  | None | 0.183333333 |
| chr1 | 231307312 | 231307313 | A | G | TRIM67 |  | ENST00000444294 | 0 | None | intron_variant | LOW |  | None |  | None | 0.265306122 |
| chr1 | 233011988 | 233011989 | C | A | None |  |  | 0 | None | intergenic_variant | LOW |  | None |  | None | 0.272727273 |
| chr1 | 233044985 | 233044986 | G | A | None |  |  | 1 | None | intergenic_variant | LOW |  | None |  | None | 0.195652174 |
| chr1 | 233511485 | 233511486 | T | C | MLK4 |  | ENST00000366624 | 0 | None | intron_variant | LOW |  | None |  | None | 0.181818182 |
| chr1 | 234332638 | 234332639 | G | A | SLC35F3 |  | ENST00000366618 | 0 | None | intron_variant | LOW |  | None |  | None | 0.12962963 |
| chr1 | 235631133 | 235631134 | G | C | B3GALNT2 |  | ENST00000366600 | 0 | None | intron_variant | LOW |  | None |  | None | 0.13559322 |
| chr1 | 235681756 | 235681757 | T | C | RP4-534P7.2 |  | ENST00000457471 | 0 | None | upstream_gene_variant | LOW |  | None |  | None | 0.37254902 |
| chr1 | 235681766 | 235681767 | C | T | RP4-534P7.2 |  | ENST00000457471 | 0 | None | upstream_gene_variant | LOW |  | None |  | None | 0.244897959 |
| chr1 | 235681769 | 235681770 | G | A | RP4-534P7.2 |  | ENST00000457471 | 0 | None | upstream_gene_variant | LOW |  | None |  | None | 0.230769231 |
| chr1 | 235714586 | 235714587 | T | C | GNG4 |  | ENST00000391854 | 0 | None | 3_prime_UTR_variant | LOW |  | None |  | None | 0.14 |
| chr1 | 236180973 | 236180974 | G | A | NID1 |  | ENST00000366595 | 0 | None | intron_variant | LOW |  | None |  | None | 0.473684211 |
| chr1 | 237088970 | 237088971 | G | A | None |  |  | 0 | None | intergenic_variant | LOW |  | None |  | None | 0.262295082 |
| chr1 | 237257419 | 237257420 | C | T | RYR2 |  | ENST00000366574 | 0 | None | intron_variant | LOW |  | None |  | None | 0.224137931 |
| chr1 | 237409899 | 237409900 | C | T | RYR2 |  | ENST00000366574 | 0 | None | intron_variant | LOW |  | None |  | None | 0.290909091 |
| chr1 | 237819600 | 237819601 | C | T | RYR2 |  | ENST00000366574 | 0 | None | intron_variant | LOW |  | None |  | None | 0.169491525 |
| chr1 | 238007333 | 238007334 | C | T | None |  |  | 1 | None | intergenic_variant | LOW |  | None |  | None | 0.183333333 |
| chr1 | 238107222 | 238107223 | A | C | MTRNR2L11 |  | ENST00000604646 | 1 | None | 3_prime_UTR_variant | LOW |  | None |  | None | 0.09 |
| chr1 | 238107225 | 238107226 | T | C | MTRNR2L11 |  | ENST00000604646 | 0 | None | 3_prime_UTR_variant | LOW |  | None |  | None | 0.80 |
| chr1 | 238149729 | 238149730 | C | T | None |  |  | 0 | None | intergenic_variant | LOW |  | None |  | None | 0.230769231 |
| chr1 | 238152532 | 238152533 | C | T | None |  |  | 0 | None | intergenic_variant | LOW |  | None |  | None | 0.14893617 |
| chr1 | 238154789 | 238154790 | C | T | None |  |  | 0 | None | intergenic_variant | LOW |  | None |  | None | 0.142857143 |
| chr1 | 238193880 | 238193881 | A | C | None |  |  | 0 | None | intergenic_variant | LOW |  | None |  | None | 0.205882353 |
| chr1 | 238205251 | 238205252 | G | T | None |  |  | 0 | None | intergenic_variant | LOW |  | None |  | None | 0.140350877 |
| chr1 | 238254041 | 238254042 | T | G | None |  |  | 0 | None | intergenic_variant | LOW |  | None |  | None | 0.162162162 |
| chr1 | 238490282 | 238490283 | G | T | RNU6-725P |  | ENST00000516562 | 0 | None | upstream_gene_variant | LOW |  | None |  | None | 0.118644068 |
| chr1 | 238508055 | 238508056 | G | A | None |  |  | 0 | None | intergenic_variant | LOW |  | None |  | None | 0.186046512 |
| chr1 | 238720212 | 238720213 | C | A | None |  |  | 0 | None | intergenic_variant | LOW |  | None |  | None | 0.130434783 |
| chr1 | 238816091 | 238816092 | C | T | None |  |  | 0 | None | intergenic_variant | LOW |  | None |  | None | 0.13559322 |
| chr1 | 238836970 | 238836971 | A | T | None |  |  | 0 | None | intergenic_variant | LOW |  | None |  | None | 0.169491525 |
| chr1 | 238932863 | 238932864 | A | T | None |  |  | 0 | None | intergenic_variant | LOW |  | None |  | None | 0.244897959 |
| chr1 | 239063343 | 239063344 | C | G | None |  |  | 0 | None | intergenic_variant | LOW |  | None |  | None | 0.204081633 |
| chr1 | 239316735 | 239316736 | T | G | None |  |  | 0 | None | intergenic_variant | LOW |  | None |  | None | 0.12244898 |
| chr1 | 239364370 | 239364371 | T | C | None |  |  | 0 | None | intergenic_variant | LOW |  | None |  | None | 0.2 |
| chr1 | 239613927 | 239613928 | G | A | CHRM3 |  | ENST00000468573 | 0 | None | intron_variant | LOW |  | None |  | None | 0.238095238 |
| chr1 | 239797371 | 239797372 | G | A | CHRM3 |  | ENST00000255380 | 0 | None | intron_variant | LOW |  | None |  | None | 0.134615385 |
| chr1 | 240072077 | 240072078 | G | A | CHRM3 | V/I | ENST00000255380 | 1 | COSM74151 | missense_variant | MED | benign | 0.006 | tolerated | 0.21 | 0.227272727 |
| chr1 | 240274118 | 240274119 | T | A | FMN2 |  | ENST00000447095 | 1 | None | intron_variant | LOW |  | None |  | None | 0.229166667 |
| chr1 | 240312276 | 240312277 | A | T | FMN2 |  | ENST00000447095 | 0 | None | intron_variant | LOW |  | None |  | None | 0.155555556 |
| chr1 | 240602342 | 240602343 | C | T | FMN2 |  | ENST00000319653 | 0 | None | intron_variant | LOW |  | None |  | None | 0.19047619 |
| chr1 | 240650376 | 240650377 | C | T | GREM2 |  | ENST00000318160 | 0 | None | downstream_gene_variant | LOW |  | None |  | None | 0.178571429 |
| chr1 | 240650742 | 240650743 | C | G | GREM2 |  | ENST00000318160 | 0 | None | downstream_gene_variant | LOW |  | None |  | None | 0.175438596 |
| chr1 | 240651394 | 240651395 | C | T | GREM2 |  | ENST00000318160 | 0 | None | downstream_gene_variant | LOW |  | None |  | None | 0.232142857 |
| chr1 | 240652134 | 240652135 | C | T | GREM2 |  | ENST00000318160 | 0 | None | downstream_gene_variant | LOW |  | None |  | None | 0.122807018 |
| chr1 | 240652409 | 240652410 | C | A | GREM2 |  | ENST00000318160 | 0 | None | downstream_gene_variant | LOW |  | None |  | None | 0.189655172 |
| chr1 | 240653113 | 240653114 | C | T | GREM2 |  | ENST00000318160 | 0 | None | 3_prime_UTR_variant | LOW |  | None |  | None | 0.203703704 |
| chr1 | 240653248 | 240653249 | C | G | GREM2 |  | ENST00000318160 | 0 | None | 3_prime_UTR_variant | LOW |  | None |  | None | 0.240740741 |
| chr1 | 240654258 | 240654259 | C | G | GREM2 |  | ENST00000318160 | 0 | None | 3_prime_UTR_variant | LOW |  | None |  | None | 0.282608696 |
| chr1 | 240656024 | 240656025 | C | T | GREM2 |  | ENST00000318160 | 0 | None | 3_prime_UTR_variant | LOW |  | None |  | None | 0.253333333 |
| chr1 | 240657704 | 240657705 | C | T | GREM2 |  | ENST00000318160 | 0 | None | intron_variant | LOW |  | None |  | None | 0.2 |
| chr1 | 240658579 | 240658580 | C | T | GREM2 |  | ENST00000318160 | 0 | None | intron_variant | LOW |  | None |  | None | 0.384615385 |
| chr1 | 240659826 | 240659827 | C | T | GREM2 |  | ENST00000318160 | 0 | None | intron_variant | LOW |  | None |  | None | 0.181818182 |
| chr1 | 240659975 | 240659976 | C | T | GREM2 |  | ENST00000318160 | 0 | None | intron_variant | LOW |  | None |  | None | 0.204081633 |
| chr1 | 241680676 | 241680677 | G | A | FH |  | ENST00000366560 | 0 | None | intron_variant | LOW |  | None |  | None | 0.206896552 |
| chr1 | 241789347 | 241789348 | G | A | OPN3 |  | ENST00000366554 | 0 | None | intron_variant | LOW |  | None |  | None | 0.142857143 |
| chr1 | 241790971 | 241790972 | G | A | OPN3 |  | ENST00000366554 | 0 | None | intron_variant | LOW |  | None |  | None | 0.223880597 |
| chr1 | 241791086 | 241791087 | G | C | OPN3 |  | ENST00000331838 | 0 | None | intron_variant | LOW |  | None |  | None | 0.211538462 |
| chr1 | 241791343 | 241791344 | G | C | OPN3 |  | ENST00000366554 | 0 | None | intron_variant | LOW |  | None |  | None | 0.125 |
| chr1 | 241792110 | 241792111 | G | C | OPN3 |  | ENST00000366554 | 0 | None | intron_variant | LOW |  | None |  | None | 0.208333333 |
| chr1 | 241793369 | 241793370 | G | C | CHML |  | ENST00000366553 | 0 | None | 3_prime_UTR_variant | LOW |  | None |  | None | 0.181818182 |
| chr1 | 241793503 | 241793504 | G | A | CHML |  | ENST00000366553 | 0 | None | 3_prime_UTR_variant | LOW |  | None |  | None | 0.156862745 |
| chr1 | 241795385 | 241795386 | G | A | CHML |  | ENST00000366553 | 0 | None | 3_prime_UTR_variant | LOW |  | None |  | None | 0.118644068 |
| chr1 | 241795810 | 241795811 | G | A | CHML |  | ENST00000366553 | 0 | None | 3_prime_UTR_variant | LOW |  | None |  | None | 0.16 |
| chr1 | 242569642 | 242569643 | A | C | PLD5 |  | ENST00000442594 | 0 | None | intron_variant | LOW |  | None |  | None | 0.163265306 |
| chr1 | 242844493 | 242844494 | A | G | None |  |  | 0 | None | intergenic_variant | LOW |  | None |  | None | 0.244897959 |
| chr1 | 242966870 | 242966871 | G | A | None |  |  | 0 | None | intergenic_variant | LOW |  | None |  | None | 0.103448276 |
| chr1 | 243518224 | 243518225 | A | C | SDCCAG8 |  | ENST00000435549 | 0 | None | intron_variant | LOW |  | None |  | None | 0.140350877 |
| chr1 | 244754505 | 244754506 | C | G | C1orf101 |  | ENST00000428042 | 0 | None | intron_variant | LOW |  | None |  | None | 0.1 |
| chr1 | 244900927 | 244900928 | A | G | None |  |  | 0 | None | intergenic_variant | LOW |  | None |  | None | 0.133333333 |
| chr1 | 244944878 | 244944879 | A | G | None |  |  | 0 | None | intergenic_variant | LOW |  | None |  | None | 0.177419355 |
| chr1 | 245392559 | 245392560 | C | T | KIF26B |  | ENST00000407071 | 0 | None | intron_variant | LOW |  | None |  | None | 0.174603175 |
| chr1 | 246414778 | 246414779 | T | G | SMYD3 |  | ENST00000490107 | 0 | None | intron_variant | LOW |  | None |  | None | 0.28 |
| chr1 | 246949229 | 246949230 | C | T | RP11-439E19.8 |  | ENST00000451123 | 0 | None | intron_variant | LOW |  | None |  | None | 0.07079646 |
| chr1 | 247537241 | 247537242 | G | A | None |  |  | 0 | None | intergenic_variant | LOW |  | None |  | None | 0.173076923 |
| chr1 | 248601535 | 248601536 | T | A | OR2T7 |  | ENST00000460972 | 1 | None | upstream_gene_variant | LOW |  | None |  | None | 0.103448276 |
| chr10 | 74131 | 74132 | C | T | None |  |  | 1 | None | intergenic_variant | LOW |  | None |  | None | 0.097560976 |
| chr10 | 75754 | 75755 | G | A | None |  |  | 1 | None | intergenic_variant | LOW |  | None |  | None | 0.094594595 |
| chr10 | 131561 | 131562 | C | A | IL9RP2 |  | ENST00000423948 | 0 | None | non_coding_exon_variant | LOW |  | None |  | None | 0.202898551 |
| chr10 | 829617 | 829618 | C | A | None |  |  | 0 | None | intergenic_variant | LOW |  | None |  | None | 0.189189189 |
| chr10 | 1344846 | 1344847 | G | T | ADARB2 |  | ENST00000381312 | 0 | None | intron_variant | LOW |  | None |  | None | 0.127272727 |
| chr10 | 1344881 | 1344882 | G | T | ADARB2 |  | ENST00000381312 | 0 | None | intron_variant | LOW |  | None |  | None | 0.127272727 |
| chr10 | 1344904 | 1344905 | G | T | ADARB2 |  | ENST00000381312 | 0 | None | intron_variant | LOW |  | None |  | None | 0.14 |
| chr10 | 1344912 | 1344913 | C | A | ADARB2 |  | ENST00000381312 | 0 | None | intron_variant | LOW |  | None |  | None | 0.156862745 |
| chr10 | 1516368 | 1516369 | C | T | ADARB2 |  | ENST00000381312 | 0 | None | intron_variant | LOW |  | None |  | None | 0.09375 |
| chr10 | 1600987 | 1600988 | A | G | ADARB2 |  | ENST00000381312 | 0 | None | intron_variant | LOW |  | None |  | None | 0.076923077 |
| chr10 | 1717639 | 1717640 | A | G | ADARB2 |  | ENST00000381312 | 0 | None | intron_variant | LOW |  | None |  | None | 0.194444444 |
| chr10 | 2242120 | 2242121 | C | T | RNU6-576P |  | ENST00000390854 | 1 | None | downstream_gene_variant | LOW |  | None |  | None | 0.127659574 |
| chr10 | 2479270 | 2479271 | G | A | None |  |  | 0 | None | intergenic_variant | LOW |  | None |  | None | 0.166666667 |
| chr10 | 2497887 | 2497888 | T | A | AC024908.1 |  | ENST00000580586 | 0 | None | upstream_gene_variant | LOW |  | None |  | None | 0.183333333 |
| chr10 | 2716047 | 2716048 | G | A | None |  |  | 0 | None | intergenic_variant | LOW |  | None |  | None | 0.271186441 |
| chr10 | 2801137 | 2801138 | G | C | None |  |  | 1 | None | intergenic_variant | LOW |  | None |  | None | 0.132075472 |
| chr10 | 3294849 | 3294850 | G | A | RP11-298E9.6 |  | ENST00000417149 | 1 | None | intron_variant | LOW |  | None |  | None | 0.086956522 |
| chr10 | 3756655 | 3756656 | A | T | None |  |  | 0 | None | intergenic_variant | LOW |  | None |  | None | 0.166666667 |
| chr10 | 5049827 | 5049828 | A | C | AKR1C2 |  | ENST00000407674 | 1 | None | intron_variant | LOW |  | None |  | None | 0.153846154 |
| chr10 | 5623248 | 5623249 | T | C | None |  |  | 0 | None | intergenic_variant | LOW |  | None |  | None | 0.1875 |
| chr10 | 6114738 | 6114739 | A | G | RP11-414H17.2 |  | ENST00000397237 | 0 | None | upstream_gene_variant | LOW |  | None |  | None | 0.157894737 |
| chr10 | 8408670 | 8408671 | A | T | None |  |  | 0 | None | intergenic_variant | LOW |  | None |  | None | 0.105263158 |
| chr10 | 9093260 | 9093261 | T | A | None |  |  | 0 | None | intergenic_variant | LOW |  | None |  | None | 0.212765957 |
| chr10 | 9182746 | 9182747 | C | T | None |  |  | 0 | None | intergenic_variant | LOW |  | None |  | None | 0.24137931 |
| chr10 | 9265224 | 9265225 | G | T | None |  |  | 0 | None | intergenic_variant | LOW |  | None |  | None | 0.217391304 |
| chr10 | 9318697 | 9318698 | A | G | LINC00709 |  | ENST00000458168 | 1 | None | intron_variant | LOW |  | None |  | None | 0.193548387 |
| chr10 | 9465158 | 9465159 | T | C | None |  |  | 0 | None | intergenic_variant | LOW |  | None |  | None | 0.109090909 |
| chr10 | 9465164 | 9465165 | C | T | None |  |  | 1 | None | intergenic_variant | LOW |  | None |  | None | 0.105263158 |
| chr10 | 9465203 | 9465204 | T | G | None |  |  | 0 | None | intergenic_variant | LOW |  | None |  | None | 0.101449275 |
| chr10 | 9498998 | 9498999 | G | T | None |  |  | 0 | None | intergenic_variant | LOW |  | None |  | None | 0.238095238 |
| chr10 | 9911390 | 9911391 | C | T | None |  |  | 0 | None | intergenic_variant | LOW |  | None |  | None | 0.113207547 |
| chr10 | 10117773 | 10117774 | C | A | None |  |  | 0 | None | intergenic_variant | LOW |  | None |  | None | 0.22 |
| chr10 | 10118606 | 10118607 | A | T | None |  |  | 0 | None | intergenic_variant | LOW |  | None |  | None | 0.16 |
| chr10 | 10202364 | 10202365 | T | C | None |  |  | 0 | None | intergenic_variant | LOW |  | None |  | None | 0.291666667 |
| chr10 | 10202365 | 10202366 | G | T | None |  |  | 0 | None | intergenic_variant | LOW |  | None |  | None | 0.270833333 |
| chr10 | 10353347 | 10353348 | T | A | None |  |  | 1 | None | intergenic_variant | LOW |  | None |  | None | 0.115384615 |
| chr10 | 10378659 | 10378660 | A | T | None |  |  | 0 | None | intergenic_variant | LOW |  | None |  | None | 0.333333333 |
| chr10 | 11606407 | 11606408 | A | C | USP6NL |  | ENST00000609853 | 0 | None | 3_prime_UTR_variant | LOW |  | None |  | None | 0.117647059 |
| chr10 | 12194403 | 12194404 | C | T | SEC61A2 |  | ENST00000298428 | 0 | None | intron_variant | LOW |  | None |  | None | 0.138888889 |
| chr10 | 12455579 | 12455580 | G | A | CAMK1D |  | ENST00000378845 | 0 | None | intron_variant | LOW |  | None |  | None | 0.064102564 |
| chr10 | 12619411 | 12619412 | G | A | CAMK1D |  | ENST00000378847 | 0 | None | intron_variant | LOW |  | None |  | None | 0.220588235 |
| chr10 | 12760707 | 12760708 | G | C | CAMK1D |  | ENST00000378847 | 0 | None | intron_variant | LOW |  | None |  | None | 0.23255814 |
| chr10 | 14139443 | 14139444 | T | G | FRMD4A |  | ENST00000378503 | 0 | None | intron_variant | LOW |  | None |  | None | 0.224137931 |
| chr10 | 14570096 | 14570097 | G | A | FAM107B |  | ENST00000489100 | 0 | None | intron_variant | LOW |  | None |  | None | 0.227272727 |
| chr10 | 14577077 | 14577078 | G | T | FAM107B |  | ENST00000378465 | 0 | None | intron_variant | LOW |  | None |  | None | 0.160714286 |
| chr10 | 14952665 | 14952666 | A | C | DCLRE1C |  | ENST00000357717 | 0 | None | intron_variant | LOW |  | None |  | None | 0.205882353 |
| chr10 | 15657755 | 15657756 | G | A | ITGA8 |  | ENST00000378076 | 0 | None | intron_variant | LOW |  | None |  | None | 0.246153846 |
| chr10 | 15900638 | 15900639 | A | G | FAM188A |  | ENST00000277632 | 0 | None | intron_variant | LOW |  | None |  | None | 0.288461538 |
| chr10 | 16121924 | 16121925 | A | G | None |  |  | 0 | None | intergenic_variant | LOW |  | None |  | None | 0.181818182 |
| chr10 | 16273944 | 16273945 | G | A | None |  |  | 0 | None | intergenic_variant | LOW |  | None |  | None | 0.2 |
| chr10 | 16528057 | 16528058 | C | A | PTER |  | ENST00000378000 | 0 | None | intron_variant | LOW |  | None |  | None | 0.245283019 |
| chr10 | 16637748 | 16637749 | C | T | RSU1 |  | ENST00000345264 | 0 | None | intron_variant | LOW |  | None |  | None | 0.228070175 |
| chr10 | 16822470 | 16822471 | G | A | RSU1 |  | ENST00000377921 | 0 | None | intron_variant | LOW |  | None |  | None | 0.153846154 |
| chr10 | 18929412 | 18929413 | T | C | NSUN6 |  | ENST00000377304 | 0 | None | intron_variant | LOW |  | None |  | None | 0.25 |
| chr10 | 19079255 | 19079256 | A | G | None |  |  | 0 | None | intergenic_variant | LOW |  | None |  | None | 0.166666667 |
| chr10 | 19141808 | 19141809 | G | T | None |  |  | 0 | None | intergenic_variant | LOW |  | None |  | None | 0.176470588 |
| chr10 | 19199207 | 19199208 | T | C | None |  |  | 0 | None | intergenic_variant | LOW |  | None |  | None | 0.229166667 |
| chr10 | 19252126 | 19252127 | G | T | None |  |  | 0 | None | intergenic_variant | LOW |  | None |  | None | 0.209677419 |
| chr10 | 19252127 | 19252128 | G | T | None |  |  | 0 | None | intergenic_variant | LOW |  | None |  | None | 0.196721311 |
| chr10 | 19804596 | 19804597 | T | C | C10orf112 |  | ENST00000454679 | 0 | None | intron_variant | LOW |  | None |  | None | 0.36 |
| chr10 | 20190170 | 20190171 | G | T | PLXDC2 |  | ENST00000377252 | 0 | None | intron_variant | LOW |  | None |  | None | 0.203125 |
| chr10 | 20248154 | 20248155 | A | G | PLXDC2 |  | ENST00000377242 | 0 | None | intron_variant | LOW |  | None |  | None | 0.25862069 |
| chr10 | 21094546 | 21094547 | G | A | NEBL |  | ENST00000377159 | 0 | None | intron_variant | LOW |  | None |  | None | 0.3 |
| chr10 | 21978546 | 21978547 | T | C | MLLT10 |  | ENST00000420525 | 1 | None | intron_variant | LOW |  | None |  | None | 0.157894737 |
| chr10 | 22089323 | 22089324 | T | G | DNAJC1 |  | ENST00000376980 | 0 | None | intron_variant | LOW |  | None |  | None | 0.184615385 |
| chr10 | 23811925 | 23811926 | T | C | None |  |  | 0 | None | intergenic_variant | LOW |  | None |  | None | 0.128571429 |
| chr10 | 24341197 | 24341198 | T | C | KIAA1217 |  | ENST00000376462 | 0 | None | intron_variant | LOW |  | None |  | None | 0.287878788 |
| chr10 | 24605671 | 24605672 | A | G | KIAA1217 |  | ENST00000376452 | 0 | None | intron_variant | LOW |  | None |  | None | 0.186046512 |
| chr10 | 25491289 | 25491290 | G | A | GPR158 |  | ENST00000376351 | 0 | None | intron_variant | LOW |  | None |  | None | 0.234042553 |
| chr10 | 25663985 | 25663986 | G | A | GPR158 |  | ENST00000376351 | 0 | None | intron_variant | LOW |  | None |  | None | 0.189189189 |
| chr10 | 26428599 | 26428600 | G | A | MYO3A |  | ENST00000543632 | 0 | None | intron_variant | LOW |  | None |  | None | 0.217391304 |
| chr10 | 26622027 | 26622028 | G | T | None |  |  | 0 | None | intergenic_variant | LOW |  | None |  | None | 0.185185185 |
| chr10 | 26653317 | 26653318 | G | T | None |  |  | 0 | None | intergenic_variant | LOW |  | None |  | None | 0.375 |
| chr10 | 26847321 | 26847322 | G | A | APBB1IP |  | ENST00000376236 | 1 | None | intron_variant | LOW |  | None |  | None | 0.265306122 |
| chr10 | 26861610 | 26861611 | T | G | APBB1IP |  | ENST00000376236 | 0 | None | downstream_gene_variant | LOW |  | None |  | None | 0.16 |
| chr10 | 26861618 | 26861619 | G | A | APBB1IP |  | ENST00000376236 | 0 | None | downstream_gene_variant | LOW |  | None |  | None | 0.175675676 |
| chr10 | 26861627 | 26861628 | C | T | APBB1IP |  | ENST00000376236 | 1 | None | downstream_gene_variant | LOW |  | None |  | None | 0.191780822 |
| chr10 | 27455434 | 27455435 | T | C | MASTL |  | ENST00000375946 | 0 | None | intron_variant | LOW |  | None |  | None | 0.177777778 |
| chr10 | 27855306 | 27855307 | A | T | None |  |  | 0 | None | intergenic_variant | LOW |  | None |  | None | 0.246376812 |
| chr10 | 28184968 | 28184969 | G | T | ARMC4 |  | ENST00000305242 | 0 | None | intron_variant | LOW |  | None |  | None | 0.293103448 |
| chr10 | 28203626 | 28203627 | A | G | ARMC4 |  | ENST00000545014 | 0 | None | intron_variant | LOW |  | None |  | None | 0.321428571 |
| chr10 | 29784003 | 29784004 | G | A | SVIL |  | ENST00000375400 | 1 | None | intron_variant | LOW |  | None |  | None | 0.106060606 |
| chr10 | 30198917 | 30198918 | G | A | None |  |  | 0 | None | intergenic_variant | LOW |  | None |  | None | 0.298245614 |
| chr10 | 30881907 | 30881908 | G | C | None |  |  | 0 | None | intergenic_variant | LOW |  | None |  | None | 0.309090909 |
| chr10 | 31086763 | 31086764 | G | C | None |  |  | 0 | None | intergenic_variant | LOW |  | None |  | None | 0.098360656 |
| chr10 | 31904637 | 31904638 | C | T | None |  |  | 0 | None | intergenic_variant | LOW |  | None |  | None | 0.338461538 |
| chr10 | 31937802 | 31937803 | C | T | None |  |  | 0 | None | intergenic_variant | LOW |  | None |  | None | 0.203389831 |
| chr10 | 32112871 | 32112872 | G | A | ARHGAP12 |  | ENST00000375250 | 0 | None | intron_variant | LOW |  | None |  | None | 0.155555556 |
| chr10 | 32880654 | 32880655 | G | T | C10orf68 |  | ENST00000375028 | 0 | None | intron_variant | LOW |  | None |  | None | 0.339285714 |
| chr10 | 35395716 | 35395717 | A | G | RP11-297A16.2 |  | ENST00000457255 | 0 | None | intron_variant | LOW |  | None |  | None | 0.202702703 |
| chr10 | 36619900 | 36619901 | G | A | None |  |  | 0 | None | intergenic_variant | LOW |  | None |  | None | 0.142857143 |
| chr10 | 36619902 | 36619903 | C | T | None |  |  | 0 | None | intergenic_variant | LOW |  | None |  | None | 0.142857143 |
| chr10 | 36687704 | 36687705 | C | T | None |  |  | 0 | None | intergenic_variant | LOW |  | None |  | None | 0.220338983 |
| chr10 | 36928849 | 36928850 | C | T | None |  |  | 0 | None | intergenic_variant | LOW |  | None |  | None | 0.204081633 |
| chr10 | 37771657 | 37771658 | A | C | None |  |  | 0 | None | intergenic_variant | LOW |  | None |  | None | 0.193548387 |
| chr10 | 37822890 | 37822891 | C | T | TACC1P1 |  | ENST00000425494 | 0 | None | intron_variant | LOW |  | None |  | None | 0.277777778 |
| chr10 | 38115725 | 38115726 | G | T | ZNF248 |  | ENST00000357328 | 0 | None | downstream_gene_variant | LOW |  | None |  | None | 0.203389831 |
| chr10 | 38318469 | 38318470 | A | C | ZNF33A |  | ENST00000469037 | 0 | None | intron_variant | LOW |  | None |  | None | 0.164179104 |
| chr10 | 38616212 | 38616213 | A | G | RP11-672F9.1 |  | ENST00000450980 | 1 | None | intron_variant | LOW |  | None |  | None | 0.22 |
| chr10 | 38616212 | 38616213 | A | C | RP11-672F9.1 |  | ENST00000450980 | 1 | None | intron_variant | LOW |  | None |  | None | 0.31 |
| chr10 | 38873331 | 38873332 | A | C | None |  |  | 1 | None | intergenic_variant | LOW |  | None |  | None | 0.16 |
| chr10 | 38873331 | 38873332 | A | G | None |  |  | 1 | None | intergenic_variant | LOW |  | None |  | None | 0.38 |
| chr10 | 38887863 | 38887864 | G | A | None |  |  | 0 | None | intergenic_variant | LOW |  | None |  | None | 0.130434783 |
| chr10 | 38962404 | 38962405 | C | T | SLC9B1P3 |  | ENST00000452667 | 0 | None | intron_variant | LOW |  | None |  | None | 0.109375 |
| chr10 | 38964492 | 38964493 | A | T | SLC9B1P3 |  | ENST00000452667 | 1 | None | intron_variant | LOW |  | None |  | None | 0.18627451 |
| chr10 | 39054711 | 39054712 | T | C | RP11-453N3.1 |  | ENST00000423458 | 1 | None | downstream_gene_variant | LOW |  | None |  | None | 0.162162162 |
| chr10 | 39068158 | 39068159 | C | T | None |  |  | 0 | None | intergenic_variant | LOW |  | None |  | None | 0.089430894 |
| chr10 | 39069091 | 39069092 | C | T | None |  |  | 1 | None | intergenic_variant | LOW |  | None |  | None | 0.148148148 |
| chr10 | 39069122 | 39069123 | A | G | None |  |  | 1 | None | intergenic_variant | LOW |  | None |  | None | 0.232142857 |
| chr10 | 39133567 | 39133568 | T | C | None |  |  | 0 | None | intergenic_variant | LOW |  | None |  | None | 0.05785124 |
| chr10 | 42363100 | 42363101 | C | T | None |  |  | 1 | None | intergenic_variant | LOW |  | None |  | None | 0.1 |
| chr10 | 42372647 | 42372648 | A | C | None |  |  | 0 | None | intergenic_variant | LOW |  | None |  | None | 0.084269663 |
| chr10 | 42408022 | 42408023 | T | C | None |  |  | 0 | None | intergenic_variant | LOW |  | None |  | None | 0.153846154 |
| chr10 | 42446013 | 42446014 | A | T | None |  |  | 0 | None | intergenic_variant | LOW |  | None |  | None | 0.158730159 |
| chr10 | 42524092 | 42524093 | C | T | None |  |  | 0 | None | intergenic_variant | LOW |  | None |  | None | 0.114754098 |
| chr10 | 42544760 | 42544761 | A | G | None |  |  | 1 | None | intergenic_variant | LOW |  | None |  | None | 0.16091954 |
| chr10 | 42602980 | 42602981 | A | G | None |  |  | 1 | None | intergenic_variant | LOW |  | None |  | None | 0.074074074 |
| chr10 | 42603021 | 42603022 | T | C | None |  |  | 1 | None | intergenic_variant | LOW |  | None |  | None | 0.096153846 |
| chr10 | 42611183 | 42611184 | T | C | None |  |  | 0 | None | intergenic_variant | LOW |  | None |  | None | 0.107594937 |
| chr10 | 42613542 | 42613543 | G | A | None |  |  | 1 | None | intergenic_variant | LOW |  | None |  | None | 0.12 |
| chr10 | 42613542 | 42613543 | G | T | None |  |  | 0 | None | intergenic_variant | LOW |  | None |  | None | 0.18 |
| chr10 | 42651406 | 42651407 | G | A | None |  |  | 0 | None | intergenic_variant | LOW |  | None |  | None | 0.19 |
| chr10 | 42651406 | 42651407 | G | T | None |  |  | 1 | None | intergenic_variant | LOW |  | None |  | None | 0.28 |
| chr10 | 42680091 | 42680092 | C | G | IGKV1OR10-1 |  | ENST00000442306 | 0 | None | upstream_gene_variant | LOW |  | None |  | None | 0.225352113 |
| chr10 | 42680128 | 42680129 | A | C | IGKV1OR10-1 |  | ENST00000442306 | 0 | None | upstream_gene_variant | LOW |  | None |  | None | 0.303797468 |
| chr10 | 42747926 | 42747927 | T | C | None |  |  | 0 | None | intergenic_variant | LOW |  | None |  | None | 0.180451128 |
| chr10 | 42786353 | 42786354 | A | G | None |  |  | 1 | None | intergenic_variant | LOW |  | None |  | None | 0.214285714 |
| chr10 | 42786371 | 42786372 | A | G | None |  |  | 1 | None | intergenic_variant | LOW |  | None |  | None | 0.267857143 |
| chr10 | 42794790 | 42794791 | C | T | None |  |  | 0 | None | intergenic_variant | LOW |  | None |  | None | 0.098765432 |
| chr10 | 42856280 | 42856281 | A | G | RP11-313J2.1 |  | ENST00000609841 | 1 | None | intron_variant | LOW |  | None |  | None | 0.139534884 |
| chr10 | 44069317 | 44069318 | A | G | ZNF239 |  | ENST00000535642 | 0 | None | intron_variant | LOW |  | None |  | None | 0.266666667 |
| chr10 | 44090078 | 44090079 | A | G | None |  |  | 0 | None | intergenic_variant | LOW |  | None |  | None | 0.184210526 |
| chr10 | 44328664 | 44328665 | T | G | None |  |  | 0 | None | intergenic_variant | LOW |  | None |  | None | 0.275862069 |
| chr10 | 44397758 | 44397759 | C | T | RP11-168P8.5 |  | ENST00000438454 | 0 | None | intron_variant | LOW |  | None |  | None | 0.094594595 |
| chr10 | 45387590 | 45387591 | T | A | TMEM72-AS1 |  | ENST00000450287 | 0 | None | intron_variant | LOW |  | None |  | None | 0.276595745 |
| chr10 | 45528396 | 45528397 | T | C | CEP164P1 |  | ENST00000456938 | 0 | None | intron_variant | LOW |  | None |  | None | 0.228571429 |
| chr10 | 45904655 | 45904656 | A | G | ALOX5 |  | ENST00000374391 | 0 | None | intron_variant | LOW |  | None |  | None | 0.101449275 |
| chr10 | 48635847 | 48635848 | G | A | None |  |  | 0 | None | intergenic_variant | LOW |  | None |  | None | 0.307692308 |
| chr10 | 49815070 | 49815071 | G | T | ARHGAP22 |  | ENST00000435790 | 0 | None | intron_variant | LOW |  | None |  | None | 0.183333333 |
| chr10 | 50292770 | 50292771 | C | G | VSTM4 |  | ENST00000332853 | 0 | None | intron_variant | LOW |  | None |  | None | 0.134615385 |
| chr10 | 50870200 | 50870201 | A | G | CHAT |  | ENST00000455728 | 0 | None | intron_variant | LOW |  | None |  | None | 0.214285714 |
| chr10 | 52442791 | 52442792 | C | T | NUTM2HP |  | ENST00000442076 | 0 | None | non_coding_exon_variant | LOW |  | None |  | None | 0.152542373 |
| chr10 | 52482667 | 52482668 | G | T | PGGT1BP1 |  | ENST00000455321 | 0 | None | downstream_gene_variant | LOW |  | None |  | None | 0.275862069 |
| chr10 | 52805248 | 52805249 | A | T | PRKG1 |  | ENST00000373985 | 0 | None | intron_variant | LOW |  | None |  | None | 0.127659574 |
| chr10 | 52979060 | 52979061 | G | A | PRKG1 |  | ENST00000373980 | 0 | None | intron_variant | LOW |  | None |  | None | 0.22 |
| chr10 | 52980086 | 52980087 | C | T | PRKG1 |  | ENST00000373985 | 0 | None | intron_variant | LOW |  | None |  | None | 0.263888889 |
| chr10 | 54115304 | 54115305 | C | T | None |  |  | 0 | None | intergenic_variant | LOW |  | None |  | None | 0.20754717 |
| chr10 | 54349813 | 54349814 | G | A | RP11-556E13.1 |  | ENST00000443523 | 0 | None | intron_variant | LOW |  | None |  | None | 0.209302326 |
| chr10 | 54476083 | 54476084 | C | A | RP11-556E13.1 |  | ENST00000443523 | 0 | None | intron_variant | LOW |  | None |  | None | 0.181818182 |
| chr10 | 54525703 | 54525704 | C | A | MBL2 |  | ENST00000373968 | 0 | None | 3_prime_UTR_variant | LOW |  | None |  | None | 0.192307692 |
| chr10 | 54827280 | 54827281 | T | A | None |  |  | 0 | None | intergenic_variant | LOW |  | None |  | None | 0.295454545 |
| chr10 | 55424325 | 55424326 | A | T | None |  |  | 0 | None | intergenic_variant | LOW |  | None |  | None | 0.170731707 |
| chr10 | 55523866 | 55523867 | C | T | RP11-449J3.3 |  | ENST00000426885 | 0 | None | upstream_gene_variant | LOW |  | None |  | None | 0.103448276 |
| chr10 | 55870154 | 55870155 | A | T | PCDH15 |  | ENST00000395433 | 0 | None | intron_variant | LOW |  | None |  | None | 0.146341463 |
| chr10 | 55921335 | 55921336 | A | T | PCDH15 |  | ENST00000373957 | 0 | None | intron_variant | LOW |  | None |  | None | 0.11627907 |
| chr10 | 56506632 | 56506633 | A | T | PCDH15 |  | ENST00000395442 | 0 | None | intron_variant | LOW |  | None |  | None | 0.186046512 |
| chr10 | 56983831 | 56983832 | C | T | PCDH15 |  | ENST00000373957 | 0 | None | intron_variant | LOW |  | None |  | None | 0.227272727 |
| chr10 | 57347751 | 57347752 | G | A | PCDH15 |  | ENST00000373957 | 1 | None | intron_variant | LOW |  | None |  | None | 0.22 |
| chr10 | 57433133 | 57433134 | C | T | None |  |  | 0 | None | intergenic_variant | LOW |  | None |  | None | 0.2 |
| chr10 | 57477840 | 57477841 | A | C | None |  |  | 0 | None | intergenic_variant | LOW |  | None |  | None | 0.131147541 |
| chr10 | 58739070 | 58739071 | T | C | None |  |  | 0 | None | intergenic_variant | LOW |  | None |  | None | 0.137254902 |
| chr10 | 59088389 | 59088390 | A | G | None |  |  | 0 | None | intergenic_variant | LOW |  | None |  | None | 0.2 |
| chr10 | 59272237 | 59272238 | G | A | RP11-550A9.1 |  | ENST00000434711 | 0 | None | upstream_gene_variant | LOW |  | None |  | None | 0.350877193 |
| chr10 | 59340156 | 59340157 | T | C | None |  |  | 0 | None | intergenic_variant | LOW |  | None |  | None | 0.225 |
| chr10 | 59386932 | 59386933 | G | A | None |  |  | 0 | None | intergenic_variant | LOW |  | None |  | None | 0.245283019 |
| chr10 | 59523760 | 59523761 | C | T | None |  |  | 0 | None | intergenic_variant | LOW |  | None |  | None | 0.183333333 |
| chr10 | 59569014 | 59569015 | G | A | None |  |  | 0 | None | intergenic_variant | LOW |  | None |  | None | 0.333333333 |
| chr10 | 59906924 | 59906925 | C | A | None |  |  | 0 | None | intergenic_variant | LOW |  | None |  | None | 0.175438596 |
| chr10 | 61754817 | 61754818 | A | C | None |  |  | 0 | None | intergenic_variant | LOW |  | None |  | None | 0.259259259 |
| chr10 | 61979068 | 61979069 | A | C | ANK3 |  | ENST00000280772 | 0 | None | intron_variant | LOW |  | None |  | None | 0.180327869 |
| chr10 | 62431650 | 62431651 | C | T | ANK3 |  | ENST00000373827 | 0 | None | intron_variant | LOW |  | None |  | None | 0.227272727 |
| chr10 | 62817372 | 62817373 | C | A | None |  |  | 1 | None | intergenic_variant | LOW |  | None |  | None | 0.244444444 |
| chr10 | 62842620 | 62842621 | A | G | None |  |  | 0 | None | intergenic_variant | LOW |  | None |  | None | 0.14 |
| chr10 | 63212479 | 63212480 | G | T | TMEM26 |  | ENST00000399293 | 0 | None | intron_variant | LOW |  | None |  | None | 0.245283019 |
| chr10 | 64456710 | 64456711 | C | T | None |  |  | 0 | None | intergenic_variant | LOW |  | None |  | None | 0.293103448 |
| chr10 | 64948254 | 64948255 | G | A | JMJD1C |  | ENST00000542921 | 0 | None | intron_variant | LOW |  | None |  | None | 0.24137931 |
| chr10 | 65067170 | 65067171 | G | A | JMJD1C |  | ENST00000399251 | 0 | None | intron_variant | LOW |  | None |  | None | 0.208333333 |
| chr10 | 65472350 | 65472351 | A | G | RP11-170M17.1 |  | ENST00000444770 | 0 | None | intron_variant | LOW |  | None |  | None | 0.278481013 |
| chr10 | 65599953 | 65599954 | C | A | RP11-170M17.1 |  | ENST00000444770 | 0 | None | intron_variant | LOW |  | None |  | None | 0.127272727 |
| chr10 | 66926924 | 66926925 | G | A | MYL6P3 |  | ENST00000436904 | 0 | None | downstream_gene_variant | LOW |  | None |  | None | 0.156862745 |
| chr10 | 67200936 | 67200937 | C | T | None |  |  | 0 | None | intergenic_variant | LOW |  | None |  | None | 0.105263158 |
| chr10 | 67371751 | 67371752 | A | T | RP11-222A11.1 |  | ENST00000601979 | 0 | None | intron_variant | LOW |  | None |  | None | 0.28358209 |
| chr10 | 67769372 | 67769373 | A | T | CTNNA3 |  | ENST00000373744 | 0 | None | intron_variant | LOW |  | None |  | None | 0.155172414 |
| chr10 | 67863847 | 67863848 | G | A | CTNNA3 |  | ENST00000373744 | 0 | None | intron_variant | LOW |  | None |  | None | 0.155172414 |
| chr10 | 67981601 | 67981602 | G | T | CTNNA3 |  | ENST00000373744 | 0 | None | intron_variant | LOW |  | None |  | None | 0.276595745 |
| chr10 | 68107648 | 68107649 | G | T | CTNNA3 |  | ENST00000373744 | 0 | None | intron_variant | LOW |  | None |  | None | 0.166666667 |
| chr10 | 68259204 | 68259205 | G | A | CTNNA3 |  | ENST00000433211 | 0 | None | intron_variant | LOW |  | None |  | None | 0.282608696 |
| chr10 | 68485180 | 68485181 | G | A | CTNNA3 |  | ENST00000433211 | 0 | None | intron_variant | LOW |  | None |  | None | 0.223880597 |
| chr10 | 68805626 | 68805627 | C | A | LRRTM3 |  | ENST00000361320 | 0 | None | intron_variant | LOW |  | None |  | None | 0.271186441 |
| chr10 | 70506069 | 70506070 | A | G | CCAR1 |  | ENST00000265872 | 0 | None | intron_variant | LOW |  | None |  | None | 0.136986301 |
| chr10 | 71511137 | 71511138 | C | T | None |  |  | 0 | None | intergenic_variant | LOW |  | None |  | None | 0.179104478 |
| chr10 | 71575312 | 71575313 | C | T | COL13A1 |  | ENST00000398973 | 0 | None | intron_variant | LOW |  | None |  | None | 0.288461538 |
| chr10 | 71585743 | 71585744 | G | A | COL13A1 |  | ENST00000398973 | 0 | None | intron_variant | LOW |  | None |  | None | 0.25 |
| chr10 | 73675308 | 73675309 | G | A | None |  |  | 0 | None | intergenic_variant | LOW |  | None |  | None | 0.22972973 |
| chr10 | 74186020 | 74186021 | G | T | MICU1 |  | ENST00000489666 | 0 | None | intron_variant | LOW |  | None |  | None | 0.1 |
| chr10 | 74590651 | 74590652 | T | C | MCU |  | ENST00000536019 | 0 | None | intron_variant | LOW |  | None |  | None | 0.241935484 |
| chr10 | 75017658 | 75017659 | G | A | TTC18 |  | ENST00000401621 | 0 | None | intron_variant | LOW |  | None |  | None | 0.15 |
| chr10 | 78501025 | 78501026 | A | G | None |  |  | 0 | None | intergenic_variant | LOW |  | None |  | None | 0.192982456 |
| chr10 | 78748003 | 78748004 | C | A | KCNMA1 |  | ENST00000372437 | 0 | None | intron_variant | LOW |  | None |  | None | 0.241935484 |
| chr10 | 79826076 | 79826077 | G | A | GNAI2P2 |  | ENST00000417003 | 0 | None | downstream_gene_variant | LOW |  | None |  | None | 0.166666667 |
| chr10 | 80479654 | 80479655 | C | G | None |  |  | 0 | None | intergenic_variant | LOW |  | None |  | None | 0.166666667 |
| chr10 | 80479677 | 80479678 | G | C | None |  |  | 0 | None | intergenic_variant | LOW |  | None |  | None | 0.169230769 |
| chr10 | 82879324 | 82879325 | C | G | None |  |  | 0 | None | intergenic_variant | LOW |  | None |  | None | 0.163265306 |
| chr10 | 82884088 | 82884089 | G | T | None |  |  | 1 | None | intergenic_variant | LOW |  | None |  | None | 0.170212766 |
| chr10 | 83313253 | 83313254 | C | A | None |  |  | 0 | None | intergenic_variant | LOW |  | None |  | None | 0.106060606 |
| chr10 | 83319813 | 83319814 | T | A | None |  |  | 0 | None | intergenic_variant | LOW |  | None |  | None | 0.181818182 |
| chr10 | 83851792 | 83851793 | T | C | NRG3 |  | ENST00000556918 | 0 | None | intron_variant | LOW |  | None |  | None | 0.16 |
| chr10 | 86232208 | 86232209 | C | T | CCSER2 |  | ENST00000224756 | 0 | None | intron_variant | LOW |  | None |  | None | 0.173076923 |
| chr10 | 86837899 | 86837900 | T | A | None |  |  | 0 | None | intergenic_variant | LOW |  | None |  | None | 0.147058824 |
| chr10 | 87010590 | 87010591 | A | T | None |  |  | 0 | None | intergenic_variant | LOW |  | None |  | None | 0.177777778 |
| chr10 | 87262950 | 87262951 | G | T | None |  |  | 0 | None | intergenic_variant | LOW |  | None |  | None | 0.1875 |
| chr10 | 87323238 | 87323239 | G | A | RN7SKP84 |  | ENST00000516921 | 0 | None | downstream_gene_variant | LOW |  | None |  | None | 0.22 |
| chr10 | 87519634 | 87519635 | A | G | GRID1 |  | ENST00000327946 | 0 | None | intron_variant | LOW |  | None |  | None | 0.25 |
| chr10 | 89697021 | 89697022 | T | G | PTEN |  | ENST00000371953 | 0 | None | intron_variant | LOW |  | None |  | None | 0.196721311 |
| chr10 | 91466258 | 91466259 | C | T | KIF20B |  | ENST00000447580 | 0 | None | intron_variant | LOW |  | None |  | None | 0.212121212 |
| chr10 | 93877230 | 93877231 | T | A | CPEB3 |  | ENST00000412050 | 0 | None | intron_variant | LOW |  | None |  | None | 0.217391304 |
| chr10 | 94356307 | 94356308 | T | C | KIF11 |  | ENST00000260731 | 1 | None | intron_variant | LOW |  | None |  | None | 0.140350877 |
| chr10 | 94563665 | 94563666 | T | A | None |  |  | 0 | None | intergenic_variant | LOW |  | None |  | None | 0.260869565 |
| chr10 | 94847529 | 94847530 | G | A | None |  |  | 0 | None | intergenic_variant | LOW |  | None |  | None | 0.278688525 |
| chr10 | 95405603 | 95405604 | A | G | PDE6C |  | ENST00000371447 | 0 | None | intron_variant | LOW |  | None |  | None | 0.267857143 |
| chr10 | 96553943 | 96553944 | C | T | CYP2C19 |  | ENST00000371321 | 1 | None | intron_variant | LOW |  | None |  | None | 0.244897959 |
| chr10 | 96785671 | 96785672 | C | A | None |  |  | 0 | None | intergenic_variant | LOW |  | None |  | None | 0.208955224 |
| chr10 | 98790792 | 98790793 | G | A | ARHGAP19-SLIT1 |  | ENST00000453547 | 0 | None | intron_variant | LOW |  | None |  | None | 0.260273973 |
| chr10 | 99166048 | 99166049 | C | A | RRP12 |  | ENST00000370992 | 0 | None | upstream_gene_variant | LOW |  | None |  | None | 0.2 |
| chr10 | 99288624 | 99288625 | C | G | UBTD1 |  | ENST00000370664 | 0 | None | intron_variant | LOW |  | None |  | None | 0.176470588 |
| chr10 | 99460408 | 99460409 | A | G | None |  |  | 1 | None | intergenic_variant | LOW |  | None |  | None | 0.086956522 |
| chr10 | 101835774 | 101835775 | C | T | CPN1 | E/K | ENST00000370418 | 0 | None | missense_variant | MED | probably_damaging | 0.978 | tolerated | 0.79 | 0.185185185 |
| chr10 | 102143561 | 102143562 | A | G | LINC00263 |  | ENST00000454935 | 1 | None | downstream_gene_variant | LOW |  | None |  | None | 0.138461538 |
| chr10 | 102435063 | 102435064 | A | G | None |  |  | 1 | None | intergenic_variant | LOW |  | None |  | None | 0.171875 |
| chr10 | 104267517 | 104267518 | G | T | SUFU |  | ENST00000369902 | 0 | None | intron_variant | LOW |  | None |  | None | 0.246153846 |
| chr10 | 105053372 | 105053373 | G | A | INA |  | ENST00000369849 | 0 | None | downstream_gene_variant | LOW |  | None |  | None | 0.148148148 |
| chr10 | 105977688 | 105977689 | A | G | WDR96 |  | ENST00000357060 | 0 | None | intron_variant | LOW |  | None |  | None | 0.153846154 |
| chr10 | 105987678 | 105987679 | G | A | WDR96 |  | ENST00000357060 | 0 | None | intron_variant | LOW |  | None |  | None | 0.261904762 |
| chr10 | 107062231 | 107062232 | G | A | None |  |  | 0 | None | intergenic_variant | LOW |  | None |  | None | 0.216666667 |
| chr10 | 107069138 | 107069139 | T | A | None |  |  | 0 | None | intergenic_variant | LOW |  | None |  | None | 0.242424242 |
| chr10 | 107084339 | 107084340 | G | A | None |  |  | 0 | None | intergenic_variant | LOW |  | None |  | None | 0.133333333 |
| chr10 | 107090159 | 107090160 | A | T | None |  |  | 0 | None | intergenic_variant | LOW |  | None |  | None | 0.195121951 |
| chr10 | 107498103 | 107498104 | C | A | None |  |  | 0 | None | intergenic_variant | LOW |  | None |  | None | 0.2 |
| chr10 | 107522862 | 107522863 | G | T | None |  |  | 0 | None | intergenic_variant | LOW |  | None |  | None | 0.244444444 |
| chr10 | 107573370 | 107573371 | G | A | RP11-56I23.1 |  | ENST00000447757 | 0 | None | intron_variant | LOW |  | None |  | None | 0.196428571 |
| chr10 | 107583287 | 107583288 | T | A | RP11-56I23.1 |  | ENST00000447757 | 0 | None | upstream_gene_variant | LOW |  | None |  | None | 0.203389831 |
| chr10 | 107823328 | 107823329 | C | A | None |  |  | 0 | None | intergenic_variant | LOW |  | None |  | None | 0.245901639 |
| chr10 | 107888972 | 107888973 | G | C | None |  |  | 0 | None | intergenic_variant | LOW |  | None |  | None | 0.119402985 |
| chr10 | 107927738 | 107927739 | A | C | RP11-298H24.1 |  | ENST00000449761 | 1 | None | intron_variant | LOW |  | None |  | None | 0.166666667 |
| chr10 | 107927952 | 107927953 | G | T | RP11-298H24.1 |  | ENST00000452286 | 0 | None | intron_variant | LOW |  | None |  | None | 0.155555556 |
| chr10 | 108025117 | 108025118 | G | A | None |  |  | 0 | None | intergenic_variant | LOW |  | None |  | None | 0.285714286 |
| chr10 | 108156462 | 108156463 | A | C | None |  |  | 0 | None | intergenic_variant | LOW |  | None |  | None | 0.211538462 |
| chr10 | 108238458 | 108238459 | G | A | None |  |  | 1 | None | intergenic_variant | LOW |  | None |  | None | 0.266666667 |
| chr10 | 108706999 | 108707000 | G | T | SORCS1 |  | ENST00000344440 | 0 | None | intron_variant | LOW |  | None |  | None | 0.132075472 |
| chr10 | 108873305 | 108873306 | G | A | SORCS1 |  | ENST00000344440 | 0 | None | intron_variant | LOW |  | None |  | None | 0.188405797 |
| chr10 | 109631627 | 109631628 | T | G | RP11-215N21.1 |  | ENST00000425050 | 1 | None | intron_variant | LOW |  | None |  | None | 0.24 |
| chr10 | 110332645 | 110332646 | T | G | None |  |  | 0 | None | intergenic_variant | LOW |  | None |  | None | 0.92 |
| chr10 | 110419903 | 110419904 | G | T | None |  |  | 0 | None | intergenic_variant | LOW |  | None |  | None | 0.235294118 |
| chr10 | 110645003 | 110645004 | T | A | None |  |  | 0 | None | intergenic_variant | LOW |  | None |  | None | 0.196078431 |
| chr10 | 110839322 | 110839323 | G | T | None |  |  | 0 | None | intergenic_variant | LOW |  | None |  | None | 0.115384615 |
| chr10 | 110840593 | 110840594 | A | T | None |  |  | 0 | None | intergenic_variant | LOW |  | None |  | None | 0.174603175 |
| chr10 | 111451185 | 111451186 | A | C | None |  |  | 0 | None | intergenic_variant | LOW |  | None |  | None | 0.280701754 |
| chr10 | 111734342 | 111734343 | G | A | None |  |  | 1 | None | intergenic_variant | LOW |  | None |  | None | 0.166666667 |
| chr10 | 111919026 | 111919027 | G | A | None |  |  | 1 | None | intergenic_variant | LOW |  | None |  | None | 0.177777778 |
| chr10 | 113029236 | 113029237 | G | T | None |  |  | 0 | None | intergenic_variant | LOW |  | None |  | None | 0.203125 |
| chr10 | 113357745 | 113357746 | C | G | None |  |  | 0 | None | intergenic_variant | LOW |  | None |  | None | 0.117647059 |
| chr10 | 113447438 | 113447439 | T | C | None |  |  | 0 | None | intergenic_variant | LOW |  | None |  | None | 0.1875 |
| chr10 | 113512229 | 113512230 | T | C | None |  |  | 0 | None | intergenic_variant | LOW |  | None |  | None | 0.158730159 |
| chr10 | 114146544 | 114146545 | C | T | ACSL5 |  | ENST00000356116 | 0 | None | intron_variant | LOW |  | None |  | None | 0.191780822 |
| chr10 | 115126898 | 115126899 | C | G | None |  |  | 0 | None | intergenic_variant | LOW |  | None |  | None | 0.181818182 |
| chr10 | 115378734 | 115378735 | G | A | NRAP |  | ENST00000369358 | 0 | None | intron_variant | LOW |  | None |  | None | 0.259259259 |
| chr10 | 116330235 | 116330236 | A | G | ABLIM1 |  | ENST00000369252 | 0 | None | intron_variant | LOW |  | None |  | None | 0.184615385 |
| chr10 | 117736598 | 117736599 | C | A | None |  |  | 0 | None | intergenic_variant | LOW |  | None |  | None | 0.25 |
| chr10 | 117886707 | 117886708 | C | G | GFRA1 |  | ENST00000369236 | 0 | None | intron_variant | LOW |  | None |  | None | 0.147540984 |
| chr10 | 118163191 | 118163192 | C | A | None |  |  | 0 | None | intergenic_variant | LOW |  | None |  | None | 0.2 |
| chr10 | 118235398 | 118235399 | A | T | PNLIPRP3 |  | ENST00000369230 | 0 | None | intron_variant | LOW |  | None |  | None | 0.195652174 |
| chr10 | 119475963 | 119475964 | G | C | None |  |  | 0 | None | intergenic_variant | LOW |  | None |  | None | 0.148148148 |
| chr10 | 120196754 | 120196755 | C | T | None |  |  | 1 | None | intergenic_variant | LOW |  | None |  | None | 0.254545455 |
| chr10 | 121003082 | 121003083 | T | C | GRK5 |  | ENST00000392870 | 0 | None | intron_variant | LOW |  | None |  | None | 0.09375 |
| chr10 | 121272066 | 121272067 | T | A | RGS10 |  | ENST00000392865 | 1 | None | intron_variant | LOW |  | None |  | None | 0.084507042 |
| chr10 | 122751657 | 122751658 | C | T | None |  |  | 0 | None | intergenic_variant | LOW |  | None |  | None | 0.232142857 |
| chr10 | 122833125 | 122833126 | A | G | None |  |  | 0 | None | intergenic_variant | LOW |  | None |  | None | 0.230769231 |
| chr10 | 123402031 | 123402032 | C | A | None |  |  | 0 | None | intergenic_variant | LOW |  | None |  | None | 0.265625 |
| chr10 | 123764878 | 123764879 | G | A | TACC2 |  | ENST00000515603 | 0 | None | intron_variant | LOW |  | None |  | None | 0.208333333 |
| chr10 | 124480118 | 124480119 | C | T | None |  |  | 0 | None | intergenic_variant | LOW |  | None |  | None | 0.189655172 |
| chr10 | 124821613 | 124821614 | T | C | ACADSB |  | ENST00000358776 | 0 | None | downstream_gene_variant | LOW |  | None |  | None | 0.275862069 |
| chr10 | 125014077 | 125014078 | C | T | None |  |  | 1 | None | intergenic_variant | LOW |  | None |  | None | 0.058333333 |
| chr10 | 125480588 | 125480589 | C | A | CPXM2 |  | ENST00000368854 | 0 | None | intron_variant | LOW |  | None |  | None | 0.25 |
| chr10 | 125579615 | 125579616 | G | A | CPXM2 |  | ENST00000241305 | 1 | None | intron_variant | LOW |  | None |  | None | 0.224137931 |
| chr10 | 125844812 | 125844813 | G | C | CHST15 |  | ENST00000421115 | 0 | None | intron_variant | LOW |  | None |  | None | 0.176470588 |
| chr10 | 126382034 | 126382035 | C | A | FAM53B |  | ENST00000337318 | 0 | None | intron_variant | LOW |  | None |  | None | 0.164179104 |
| chr10 | 126440652 | 126440653 | C | T | RP11-12J10.3 |  | ENST00000494792 | 0 | None | intron_variant | LOW |  | None |  | None | 0.203125 |
| chr10 | 126550394 | 126550395 | G | A | None |  |  | 1 | None | intergenic_variant | LOW |  | None |  | None | 0.180327869 |
| chr10 | 126984215 | 126984216 | G | A | None |  |  | 0 | None | intergenic_variant | LOW |  | None |  | None | 0.191176471 |
| chr10 | 127333411 | 127333412 | C | G | TEX36 |  | ENST00000526819 | 0 | None | intron_variant | LOW |  | None |  | None | 0.156862745 |
| chr10 | 127588748 | 127588749 | C | A | FANK1 |  | ENST00000368695 | 1 | None | intron_variant | LOW |  | None |  | None | 0.042780749 |
| chr10 | 127601362 | 127601363 | T | C | FANK1 |  | ENST00000449042 | 1 | None | intron_variant | LOW |  | None |  | None | 0.11 |
| chr10 | 127601362 | 127601363 | T | A | FANK1 |  | ENST00000449042 | 1 | None | intron_variant | LOW |  | None |  | None | 0.15 |
| chr10 | 128485769 | 128485770 | C | T | None |  |  | 0 | None | intergenic_variant | LOW |  | None |  | None | 0.283018868 |
| chr10 | 129505274 | 129505275 | T | C | None |  |  | 0 | None | intergenic_variant | LOW |  | None |  | None | 0.241935484 |
| chr10 | 129813062 | 129813063 | C | T | PTPRE |  | ENST00000455661 | 0 | None | intron_variant | LOW |  | None |  | None | 0.152542373 |
| chr10 | 130299837 | 130299838 | A | G | None |  |  | 0 | None | intergenic_variant | LOW |  | None |  | None | 0.227848101 |
| chr10 | 131044257 | 131044258 | C | T | None |  |  | 0 | None | intergenic_variant | LOW |  | None |  | None | 0.260273973 |
| chr10 | 131113249 | 131113250 | G | A | None |  |  | 0 | None | intergenic_variant | LOW |  | None |  | None | 0.227272727 |
| chr10 | 131325600 | 131325601 | T | G | MGMT |  | ENST00000306010 | 0 | None | intron_variant | LOW |  | None |  | None | 0.191780822 |
| chr10 | 131517432 | 131517433 | G | A | MGMT |  | ENST00000306010 | 0 | None | intron_variant | LOW |  | None |  | None | 0.095890411 |
| chr10 | 131740510 | 131740511 | C | A | EBF3 |  | ENST00000355311 | 0 | None | intron_variant | LOW |  | None |  | None | 0.203125 |
| chr10 | 131740511 | 131740512 | C | G | EBF3 |  | ENST00000355311 | 0 | None | intron_variant | LOW |  | None |  | None | 0.203125 |
| chr10 | 131859641 | 131859642 | T | C | LINC00959 |  | ENST00000456581 | 0 | None | downstream_gene_variant | LOW |  | None |  | None | 0.153846154 |
| chr10 | 132389203 | 132389204 | T | C | None |  |  | 0 | None | intergenic_variant | LOW |  | None |  | None | 0.174603175 |
| chr10 | 132507166 | 132507167 | C | G | None |  |  | 0 | None | intergenic_variant | LOW |  | None |  | None | 0.176470588 |
| chr10 | 133078751 | 133078752 | C | G | TCERG1L |  | ENST00000368642 | 0 | None | intron_variant | LOW |  | None |  | None | 0.113207547 |
| chr10 | 133118897 | 133118898 | C | A | None |  |  | 0 | None | intergenic_variant | LOW |  | None |  | None | 0.173913043 |
| chr10 | 133493550 | 133493551 | T | G | AL450307.2 |  | ENST00000579404 | 0 | None | downstream_gene_variant | LOW |  | None |  | None | 0.203703704 |
| chr10 | 133883954 | 133883955 | C | T | None |  |  | 0 | None | intergenic_variant | LOW |  | None |  | None | 0.216666667 |
| chr10 | 134417507 | 134417508 | C | T | INPP5A |  | ENST00000423490 | 0 | None | intron_variant | LOW |  | None |  | None | 0.130434783 |
| chr10 | 134494173 | 134494174 | C | G | INPP5A |  | ENST00000423490 | 0 | None | intron_variant | LOW |  | None |  | None | 0.162162162 |
| chr10 | 134639537 | 134639538 | T | C | TTC40 |  | ENST00000368586 | 0 | None | intron_variant | LOW |  | None |  | None | 0.196078431 |
| chr10 | 135031201 | 135031202 | G | C | KNDC1 |  | ENST00000304613 | 1 | None | intron_variant | LOW |  | None |  | None | 0.203703704 |
| chr10 | 135190656 | 135190657 | G | A | PAOX |  | ENST00000357296 | 0 | None | upstream_gene_variant | LOW |  | None |  | None | 0.227848101 |
| chr10 | 135444350 | 135444351 | G | T | FRG2B |  | ENST00000425520 | 1 | None | upstream_gene_variant | LOW |  | None |  | None | 0.12 |
| chr10 | 135444350 | 135444351 | G | C | FRG2B |  | ENST00000425520 | 1 | None | upstream_gene_variant | LOW |  | None |  | None | 0.24 |
| chr10 | 135449253 | 135449254 | C | G | RARRES2P2 |  | ENST00000394842 | 1 | None | downstream_gene_variant | LOW |  | None |  | None | 0.25 |
| chr10 | 135449253 | 135449254 | C | A | RARRES2P2 |  | ENST00000394842 | 1 | None | downstream_gene_variant | LOW |  | None |  | None | 0.30 |
| chr10 | 135470362 | 135470363 | T | C | AL845259.1 |  | ENST00000408181 | 0 | None | downstream_gene_variant | LOW |  | None |  | None | 0.054945055 |
| chr10 | 135523421 | 135523422 | G | A | None |  |  | 0 | None | intergenic_variant | LOW |  | None |  | None | 0.091836735 |
| chr11 | 190148 | 190149 | A | C | BET1L |  | ENST00000410108 | 1 | None | intron_variant | LOW |  | None |  | None | 0.068965517 |
| chr11 | 806317 | 806318 | A | G | RPLP2 |  | ENST00000530797 | 0 | None | upstream_gene_variant | LOW |  | None |  | None | 0.245283019 |
| chr11 | 961232 | 961233 | A | G | AP2A2 |  | ENST00000534485 | 0 | None | intron_variant | LOW |  | None |  | None | 0.136363636 |
| chr11 | 1093506 | 1093507 | A | G | MUC2 | M/V | ENST00000441003 | 1 | None | missense_variant | MED | unknown | 0 |  | None | 0.12745098 |
| chr11 | 1093618 | 1093619 | T | C | MUC2 | M/T | ENST00000359061 | 0 | None | missense_variant | MED | unknown | 0 |  | None | 0.174603175 |
| chr11 | 1508066 | 1508067 | G | A | MOB2 |  | ENST00000329957 | 0 | None | upstream_gene_variant | LOW |  | None |  | None | 0.148148148 |
| chr11 | 1706937 | 1706938 | G | A | FAM99B |  | ENST00000382166 | 1 | None | upstream_gene_variant | LOW |  | None |  | None | 0.30 |
| chr11 | 1706937 | 1706938 | G | C | AP006285.6 |  | ENST00000436539 | 1 | None | upstream_gene_variant | LOW |  | None |  | None | 0.42 |
| chr11 | 1835900 | 1835901 | G | A | None |  |  | 0 | None | intergenic_variant | LOW |  | None |  | None | 0.232876712 |
| chr11 | 2570549 | 2570550 | G | A | KCNQ1 |  | ENST00000496887 | 0 | None | intron_variant | LOW |  | None |  | None | 0.211764706 |
| chr11 | 2720846 | 2720847 | G | C | KCNQ1 |  | ENST00000155840 | 0 | None | intron_variant | LOW |  | None |  | None | 0.132075472 |
| chr11 | 3221855 | 3221856 | A | C | None |  |  | 0 | None | intergenic_variant | LOW |  | None |  | None | 0.203389831 |
| chr11 | 4027171 | 4027172 | G | T | STIM1 |  | ENST00000525055 | 0 | None | intron_variant | LOW |  | None |  | None | 0.272727273 |
| chr11 | 5323370 | 5323371 | C | A | HBE1 |  | ENST00000380237 | 0 | None | intron_variant | LOW |  | None |  | None | 0.235294118 |
| chr11 | 6039421 | 6039422 | A | G | RNA5SP329 |  | ENST00000365277 | 0 | None | downstream_gene_variant | LOW |  | None |  | None | 0.215686275 |
| chr11 | 6776761 | 6776762 | T | C | None |  |  | 0 | None | intergenic_variant | LOW |  | None |  | None | 0.156862745 |
| chr11 | 7009159 | 7009160 | C | A | ZNF215 |  | ENST00000529903 | 0 | None | downstream_gene_variant | LOW |  | None |  | None | 0.254901961 |
| chr11 | 8198548 | 8198549 | C | T | RP11-379P15.1 |  | ENST00000499752 | 0 | None | non_coding_exon_variant | LOW |  | None |  | None | 0.266666667 |
| chr11 | 8420319 | 8420320 | C | T | STK33 |  | ENST00000358872 | 0 | None | intron_variant | LOW |  | None |  | None | 0.155555556 |
| chr11 | 9062042 | 9062043 | C | T | SCUBE2 |  | ENST00000457346 | 1 | None | intron_variant | LOW |  | None |  | None | 0.193548387 |
| chr11 | 9423782 | 9423783 | T | C | IPO7 |  | ENST00000379719 | 0 | None | intron_variant | LOW |  | None |  | None | 0.120689655 |
| chr11 | 9731133 | 9731134 | C | T | SWAP70 |  | ENST00000318950 | 0 | None | intron_variant | LOW |  | None |  | None | 0.15942029 |
| chr11 | 9831580 | 9831581 | T | C | SBF2 |  | ENST00000530741 | 0 | None | intron_variant | LOW |  | None |  | None | 0.203703704 |
| chr11 | 10293083 | 10293084 | T | C | SBF2 |  | ENST00000256190 | 0 | None | intron_variant | LOW |  | None |  | None | 0.173913043 |
| chr11 | 10562094 | 10562095 | G | C | RNF141 |  | ENST00000533412 | 0 | None | intron_variant | LOW |  | None |  | None | 0.241935484 |
| chr11 | 12030145 | 12030146 | C | A | DKK3 |  | ENST00000534511 | 0 | None | 5_prime_UTR_variant | LOW |  | None |  | None | 0.174603175 |
| chr11 | 12089620 | 12089621 | G | A | RP13-631K18.3 |  | ENST00000476130 | 0 | None | intron_variant | LOW |  | None |  | None | 0.288135593 |
| chr11 | 13269565 | 13269566 | T | G | None |  |  | 0 | None | intergenic_variant | LOW |  | None |  | None | 0.240740741 |
| chr11 | 13723162 | 13723163 | A | C | FAR1 |  | ENST00000532701 | 0 | None | intron_variant | LOW |  | None |  | None | 0.217391304 |
| chr11 | 15138416 | 15138417 | C | G | INSC |  | ENST00000424273 | 1 | None | intron_variant | LOW |  | None |  | None | 0.315789474 |
| chr11 | 16425677 | 16425678 | C | T | SOX6 |  | ENST00000396356 | 0 | None | intron_variant | LOW |  | None |  | None | 0.229166667 |
| chr11 | 18254396 | 18254397 | A | T | SAA4 |  | ENST00000278222 | 0 | None | intron_variant | LOW |  | None |  | None | 0.153846154 |
| chr11 | 18741019 | 18741020 | T | A | IGSF22 |  | ENST00000513874 | 0 | None | intron_variant | LOW |  | None |  | None | 0.3 |
| chr11 | 19366731 | 19366732 | G | C | None |  |  | 0 | None | intergenic_variant | LOW |  | None |  | None | 0.136363636 |
| chr11 | 19748958 | 19748959 | A | T | NAV2 |  | ENST00000396087 | 0 | None | intron_variant | LOW |  | None |  | None | 0.291666667 |
| chr11 | 20150579 | 20150580 | C | A | None |  |  | 0 | None | intergenic_variant | LOW |  | None |  | None | 0.196428571 |
| chr11 | 20160203 | 20160204 | C | A | None |  |  | 0 | None | intergenic_variant | LOW |  | None |  | None | 0.246153846 |
| chr11 | 20920676 | 20920677 | T | G | NELL1 |  | ENST00000325319 | 0 | None | intron_variant | LOW |  | None |  | None | 0.13559322 |
| chr11 | 21239891 | 21239892 | A | T | NELL1 |  | ENST00000357134 | 0 | None | intron_variant | LOW |  | None |  | None | 0.152173913 |
| chr11 | 21466701 | 21466702 | T | A | NELL1 |  | ENST00000357134 | 0 | None | intron_variant | LOW |  | None |  | None | 0.288461538 |
| chr11 | 21477104 | 21477105 | C | T | NELL1 |  | ENST00000532434 | 0 | None | intron_variant | LOW |  | None |  | None | 0.179487179 |
| chr11 | 21884141 | 21884142 | T | A | None |  |  | 0 | None | intergenic_variant | LOW |  | None |  | None | 0.195121951 |
| chr11 | 22218440 | 22218441 | A | G | ANO5 |  | ENST00000324559 | 0 | None | intron_variant | LOW |  | None |  | None | 0.137254902 |
| chr11 | 22400012 | 22400013 | C | A | SLC17A6 |  | ENST00000263160 | 0 | None | 3_prime_UTR_variant | LOW |  | None |  | None | 0.234042553 |
| chr11 | 22590896 | 22590897 | C | T | None |  |  | 0 | None | intergenic_variant | LOW |  | None |  | None | 0.2 |
| chr11 | 22717849 | 22717850 | G | A | GAS2 |  | ENST00000532398 | 0 | None | intron_variant | LOW |  | None |  | None | 0.28 |
| chr11 | 22900392 | 22900393 | T | A | RP11-17A1.3 |  | ENST00000528701 | 0 | None | intron_variant | LOW |  | None |  | None | 0.264150943 |
| chr11 | 23577323 | 23577324 | C | T | None |  |  | 0 | None | intergenic_variant | LOW |  | None |  | None | 0.164179104 |
| chr11 | 23738175 | 23738176 | C | G | None |  |  | 0 | None | intergenic_variant | LOW |  | None |  | None | 0.096774194 |
| chr11 | 23761159 | 23761160 | T | A | RP11-945A11.1 |  | ENST00000534068 | 0 | None | intron_variant | LOW |  | None |  | None | 0.215686275 |
| chr11 | 24118858 | 24118859 | T | C | None |  |  | 0 | None | intergenic_variant | LOW |  | None |  | None | 0.176470588 |
| chr11 | 24118882 | 24118883 | A | G | None |  |  | 0 | None | intergenic_variant | LOW |  | None |  | None | 0.21875 |
| chr11 | 24120522 | 24120523 | A | G | None |  |  | 0 | None | intergenic_variant | LOW |  | None |  | None | 0.230769231 |
| chr11 | 24240533 | 24240534 | C | T | None |  |  | 0 | None | intergenic_variant | LOW |  | None |  | None | 0.156862745 |
| chr11 | 25126508 | 25126509 | G | A | None |  |  | 0 | None | intergenic_variant | LOW |  | None |  | None | 0.4 |
| chr11 | 25562374 | 25562375 | T | A | AC015820.1 |  | ENST00000580516 | 0 | None | downstream_gene_variant | LOW |  | None |  | None | 0.2 |
| chr11 | 25562414 | 25562415 | C | T | AC015820.1 |  | ENST00000580516 | 0 | None | downstream_gene_variant | LOW |  | None |  | None | 0.206896552 |
| chr11 | 25562420 | 25562421 | C | T | AC015820.1 |  | ENST00000580516 | 0 | None | downstream_gene_variant | LOW |  | None |  | None | 0.2 |
| chr11 | 25562461 | 25562462 | T | C | AC015820.1 |  | ENST00000580516 | 0 | None | downstream_gene_variant | LOW |  | None |  | None | 0.174603175 |
| chr11 | 25562474 | 25562475 | A | C | AC015820.1 |  | ENST00000580516 | 0 | None | downstream_gene_variant | LOW |  | None |  | None | 0.172413793 |
| chr11 | 25562497 | 25562498 | G | A | AC015820.1 |  | ENST00000580516 | 0 | None | downstream_gene_variant | LOW |  | None |  | None | 0.13559322 |
| chr11 | 25562498 | 25562499 | T | C | AC015820.1 |  | ENST00000580516 | 0 | None | downstream_gene_variant | LOW |  | None |  | None | 0.137931034 |
| chr11 | 26447702 | 26447703 | T | A | ANO3 |  | ENST00000256737 | 0 | None | intron_variant | LOW |  | None |  | None | 0.395348837 |
| chr11 | 26701226 | 26701227 | C | A | SLC5A12 |  | ENST00000396005 | 0 | None | intron_variant | LOW |  | None |  | None | 0.188679245 |
| chr11 | 27489127 | 27489128 | A | C | LGR4 |  | ENST00000379214 | 1 | None | intron_variant | LOW |  | None |  | None | 0.139534884 |
| chr11 | 28308686 | 28308687 | T | G | METTL15 |  | ENST00000303459 | 1 | None | intron_variant | LOW |  | None |  | None | 0.166666667 |
| chr11 | 29083072 | 29083073 | T | C | RP11-115J23.1 |  | ENST00000511073 | 0 | None | intron_variant | LOW |  | None |  | None | 0.196078431 |
| chr11 | 29464900 | 29464901 | C | A | RP11-460B17.3 |  | ENST00000525097 | 0 | None | intron_variant | LOW |  | None |  | None | 0.196428571 |
| chr11 | 29641238 | 29641239 | T | C | RP5-1027O15.1 |  | ENST00000527492 | 0 | None | intron_variant | LOW |  | None |  | None | 0.272727273 |
| chr11 | 29737700 | 29737701 | A | C | CTD-3138F19.1 |  | ENST00000530249 | 0 | None | intron_variant | LOW |  | None |  | None | 0.206349206 |
| chr11 | 29975009 | 29975010 | C | G | None |  |  | 0 | None | intergenic_variant | LOW |  | None |  | None | 0.127272727 |
| chr11 | 31449525 | 31449526 | T | A | DNAJC24 |  | ENST00000465995 | 0 | None | intron_variant | LOW |  | None |  | None | 0.173913043 |
| chr11 | 31666870 | 31666871 | T | C | ELP4 |  | ENST00000395934 | 0 | None | intron_variant | LOW |  | None |  | None | 0.178571429 |
| chr11 | 31666871 | 31666872 | A | T | ELP4 |  | ENST00000395934 | 0 | None | intron_variant | LOW |  | None |  | None | 0.181818182 |
| chr11 | 31762461 | 31762462 | C | A | ELP4 |  | ENST00000395934 | 0 | None | intron_variant | LOW |  | None |  | None | 0.1 |
| chr11 | 32096022 | 32096023 | G | T | RCN1 |  | ENST00000530348 | 0 | None | intron_variant | LOW |  | None |  | None | 0.132075472 |
| chr11 | 32547879 | 32547880 | C | A | None |  |  | 0 | None | intergenic_variant | LOW |  | None |  | None | 0.264150943 |
| chr11 | 32807007 | 32807008 | C | T | CCDC73 |  | ENST00000335185 | 0 | None | intron_variant | LOW |  | None |  | None | 0.339285714 |
| chr11 | 33582620 | 33582621 | C | T | KIAA1549L |  | ENST00000526400 | 0 | None | intron_variant | LOW |  | None |  | None | 0.160714286 |
| chr11 | 33856431 | 33856432 | T | G | None |  |  | 0 | None | intergenic_variant | LOW |  | None |  | None | 0.306666667 |
| chr11 | 34680036 | 34680037 | G | A | EHF |  | ENST00000450654 | 0 | None | intron_variant | LOW |  | None |  | None | 0.262295082 |
| chr11 | 35604252 | 35604253 | C | T | RP5-945I17.2 |  | ENST00000527684 | 0 | None | intron_variant | LOW |  | None |  | None | 0.12 |
| chr11 | 35705547 | 35705548 | C | T | TRIM44 |  | ENST00000299413 | 0 | None | intron_variant | LOW |  | None |  | None | 0.217948718 |
| chr11 | 36194476 | 36194477 | A | C | LDLRAD3 |  | ENST00000528989 | 0 | None | intron_variant | LOW |  | None |  | None | 0.265625 |
| chr11 | 36194478 | 36194479 | A | G | LDLRAD3 |  | ENST00000315571 | 0 | None | intron_variant | LOW |  | None |  | None | 0.25 |
| chr11 | 38990781 | 38990782 | C | G | None |  |  | 0 | None | intergenic_variant | LOW |  | None |  | None | 0.254901961 |
| chr11 | 39054718 | 39054719 | T | G | None |  |  | 0 | None | intergenic_variant | LOW |  | None |  | None | 0.177419355 |
| chr11 | 39100459 | 39100460 | C | G | None |  |  | 0 | None | intergenic_variant | LOW |  | None |  | None | 0.150943396 |
| chr11 | 39526154 | 39526155 | C | T | None |  |  | 0 | None | intergenic_variant | LOW |  | None |  | None | 0.142857143 |
| chr11 | 39591429 | 39591430 | T | A | None |  |  | 0 | None | intergenic_variant | LOW |  | None |  | None | 0.37254902 |
| chr11 | 39667851 | 39667852 | C | A | None |  |  | 0 | None | intergenic_variant | LOW |  | None |  | None | 0.173076923 |
| chr11 | 39760284 | 39760285 | C | T | None |  |  | 0 | None | intergenic_variant | LOW |  | None |  | None | 0.2 |
| chr11 | 39789284 | 39789285 | C | T | None |  |  | 0 | None | intergenic_variant | LOW |  | None |  | None | 0.3125 |
| chr11 | 40106707 | 40106708 | T | C | RP11-40H19.1 |  | ENST00000525564 | 0 | None | upstream_gene_variant | LOW |  | None |  | None | 0.282608696 |
| chr11 | 40205346 | 40205347 | C | G | LRRC4C |  | ENST00000527150 | 0 | None | intron_variant | LOW |  | None |  | None | 0.191489362 |
| chr11 | 40442334 | 40442335 | T | A | LRRC4C |  | ENST00000528697 | 0 | None | intron_variant | LOW |  | None |  | None | 0.169811321 |
| chr11 | 40649896 | 40649897 | G | A | LRRC4C |  | ENST00000530763 | 0 | None | intron_variant | LOW |  | None |  | None | 0.296875 |
| chr11 | 40830501 | 40830502 | T | A | LRRC4C |  | ENST00000530763 | 0 | None | intron_variant | LOW |  | None |  | None | 0.12 |
| chr11 | 41090159 | 41090160 | A | T | LRRC4C |  | ENST00000528697 | 0 | None | intron_variant | LOW |  | None |  | None | 0.259259259 |
| chr11 | 41941911 | 41941912 | T | A | None |  |  | 0 | None | intergenic_variant | LOW |  | None |  | None | 0.234042553 |
| chr11 | 42098824 | 42098825 | C | T | RP11-148I19.1 |  | ENST00000534059 | 0 | None | intron_variant | LOW |  | None |  | None | 0.223880597 |
| chr11 | 42488352 | 42488353 | T | A | None |  |  | 0 | None | intergenic_variant | LOW |  | None |  | None | 0.240740741 |
| chr11 | 42909899 | 42909900 | C | A | None |  |  | 0 | None | intergenic_variant | LOW |  | None |  | None | 0.218181818 |
| chr11 | 43138088 | 43138089 | G | T | HNRNPKP3 |  | ENST00000533565 | 0 | None | downstream_gene_variant | LOW |  | None |  | None | 0.2 |
| chr11 | 43525022 | 43525023 | T | A | None |  |  | 0 | None | intergenic_variant | LOW |  | None |  | None | 0.189655172 |
| chr11 | 43645069 | 43645070 | G | T | HSD17B12 |  | ENST00000526615 | 0 | None | intron_variant | LOW |  | None |  | None | 0.212121212 |
| chr11 | 43653269 | 43653270 | T | C | HSD17B12 |  | ENST00000526615 | 0 | None | intron_variant | LOW |  | None |  | None | 0.109375 |
| chr11 | 44540090 | 44540091 | G | T | None |  |  | 0 | None | intergenic_variant | LOW |  | None |  | None | 0.166666667 |
| chr11 | 44685145 | 44685146 | T | A | None |  |  | 0 | None | intergenic_variant | LOW |  | None |  | None | 0.105263158 |
| chr11 | 45489511 | 45489512 | C | T | RP11-430H10.4 |  | ENST00000528445 | 0 | None | intron_variant | LOW |  | None |  | None | 0.147540984 |
| chr11 | 46277301 | 46277302 | C | T | CTD-2589M5.4 |  | ENST00000527239 | 0 | None | downstream_gene_variant | LOW |  | None |  | None | 0.220779221 |
| chr11 | 46606924 | 46606925 | C | A | AMBRA1 |  | ENST00000533727 | 0 | None | intron_variant | LOW |  | None |  | None | 0.224489796 |
| chr11 | 48729538 | 48729539 | C | G | None |  |  | 0 | None | intergenic_variant | LOW |  | None |  | None | 0.230769231 |
| chr11 | 48782308 | 48782309 | A | T | None |  |  | 0 | None | intergenic_variant | LOW |  | None |  | None | 0.152173913 |
| chr11 | 48899082 | 48899083 | T | G | RP11-56P9.5 |  | ENST00000525520 | 0 | None | upstream_gene_variant | LOW |  | None |  | None | 0.082568807 |
| chr11 | 49256463 | 49256464 | T | A | None |  |  | 0 | None | intergenic_variant | LOW |  | None |  | None | 0.296296296 |
| chr11 | 50143229 | 50143230 | T | A | RP11-347H15.1 |  | ENST00000533593 | 0 | None | intron_variant | LOW |  | None |  | None | 0.209302326 |
| chr11 | 50278242 | 50278243 | C | G | RP11-574M7.1 |  | ENST00000528459 | 0 | None | intron_variant | LOW |  | None |  | None | 0.266666667 |
| chr11 | 50383409 | 50383410 | T | C | RP11-574M7.2 |  | ENST00000532521 | 0 | None | downstream_gene_variant | LOW |  | None |  | None | 0.163265306 |
| chr11 | 50503920 | 50503921 | T | C | None |  |  | 0 | None | intergenic_variant | LOW |  | None |  | None | 0.178571429 |
| chr11 | 50571365 | 50571366 | C | T | None |  |  | 0 | None | intergenic_variant | LOW |  | None |  | None | 0.111111111 |
| chr11 | 50728976 | 50728977 | T | G | None |  |  | 1 | None | intergenic_variant | LOW |  | None |  | None | 0.186440678 |
| chr11 | 50755489 | 50755490 | C | T | None |  |  | 0 | None | intergenic_variant | LOW |  | None |  | None | 0.075 |
| chr11 | 50764036 | 50764037 | T | C | None |  |  | 0 | None | intergenic_variant | LOW |  | None |  | None | 0.067307692 |
| chr11 | 51301842 | 51301843 | G | C | None |  |  | 0 | None | intergenic_variant | LOW |  | None |  | None | 0.22 |
| chr11 | 51387284 | 51387285 | A | T | None |  |  | 0 | None | intergenic_variant | LOW |  | None |  | None | 0.24 |
| chr11 | 51406342 | 51406343 | A | G | None |  |  | 0 | None | intergenic_variant | LOW |  | None |  | None | 0.206896552 |
| chr11 | 51558437 | 51558438 | G | A | None |  |  | 0 | None | intergenic_variant | LOW |  | None |  | None | 0.2 |
| chr11 | 51591819 | 51591820 | A | G | None |  |  | 0 | None | intergenic_variant | LOW |  | None |  | None | 0.085889571 |
| chr11 | 54933433 | 54933434 | T | A | None |  |  | 0 | None | intergenic_variant | LOW |  | None |  | None | 0.238095238 |
| chr11 | 54974126 | 54974127 | T | A | None |  |  | 0 | None | intergenic_variant | LOW |  | None |  | None | 0.159090909 |
| chr11 | 54982350 | 54982351 | G | T | None |  |  | 0 | None | intergenic_variant | LOW |  | None |  | None | 0.048 |
| chr11 | 55240513 | 55240514 | C | T | OR4A14P |  | ENST00000525705 | 0 | None | upstream_gene_variant | LOW |  | None |  | None | 0.210526316 |
| chr11 | 55302913 | 55302914 | G | A | OR4C14P |  | ENST00000440929 | 0 | None | upstream_gene_variant | LOW |  | None |  | None | 0.179104478 |
| chr11 | 55360359 | 55360360 | A | C | None |  |  | 0 | None | intergenic_variant | LOW |  | None |  | None | 0.230769231 |
| chr11 | 55366657 | 55366658 | A | T | OR4C11 |  | ENST00000302231 | 0 | None | downstream_gene_variant | LOW |  | None |  | None | 0.518518519 |
| chr11 | 55521468 | 55521469 | G | T | OR5D17P |  | ENST00000531492 | 0 | None | upstream_gene_variant | LOW |  | None |  | None | 0.236363636 |
| chr11 | 55568648 | 55568649 | G | T | OR5D14 |  | ENST00000335605 | 0 | None | downstream_gene_variant | LOW |  | None |  | None | 0.20754717 |
| chr11 | 55568925 | 55568926 | G | T | OR5D14 |  | ENST00000335605 | 0 | None | downstream_gene_variant | LOW |  | None |  | None | 0.24 |
| chr11 | 55597189 | 55597190 | G | T | OR5L2 |  | ENST00000378397 | 0 | None | downstream_gene_variant | LOW |  | None |  | None | 0.258064516 |
| chr11 | 55854130 | 55854131 | A | G | OR5BE1P |  | ENST00000425977 | 0 | None | downstream_gene_variant | LOW |  | None |  | None | 0.173913043 |
| chr11 | 55854227 | 55854228 | T | A | OR5BE1P |  | ENST00000425977 | 0 | None | downstream_gene_variant | LOW |  | None |  | None | 0.159090909 |
| chr11 | 57210725 | 57210726 | C | T | None |  |  | 0 | None | intergenic_variant | LOW |  | None |  | None | 0.386363636 |
| chr11 | 57328005 | 57328006 | A | G | UBE2L6 |  | ENST00000526659 | 0 | None | intron_variant | LOW |  | None |  | None | 0.228571429 |
| chr11 | 61122340 | 61122341 | T | C | CYB561A3 |  | ENST00000537364 | 1 | None | intron_variant | LOW |  | None |  | None | 0.25 |
| chr11 | 62019443 | 62019444 | G | A | RP11-703H8.9 |  | ENST00000529875 | 0 | None | intron_variant | LOW |  | None |  | None | 0.1 |
| chr11 | 63080635 | 63080636 | C | T | SLC22A10 |  | ENST00000535888 | 1 | None | intron_variant | LOW |  | None |  | None | 0.25 |
| chr11 | 65160720 | 65160721 | C | G | FRMD8 |  | ENST00000526201 | 0 | None | intron_variant | LOW |  | None |  | None | 0.235294118 |
| chr11 | 65263628 | 65263629 | G | T | MALAT1 |  | ENST00000534336 | 0 | None | upstream_gene_variant | LOW |  | None |  | None | 0.25 |
| chr11 | 66145720 | 66145721 | A | G | None |  |  | 0 | None | intergenic_variant | LOW |  | None |  | None | 0.22972973 |
| chr11 | 66765928 | 66765929 | T | G | U3 |  | ENST00000516900 | 0 | None | upstream_gene_variant | LOW |  | None |  | None | 0.142857143 |
| chr11 | 67475749 | 67475750 | C | T | UNC93B5 |  | ENST00000530315 | 1 | None | downstream_gene_variant | LOW |  | None |  | None | 0.098765432 |
| chr11 | 67501592 | 67501593 | C | A | AP003385.1 |  | ENST00000580148 | 0 | None | downstream_gene_variant | LOW |  | None |  | None | 0.131578947 |
| chr11 | 67653967 | 67653968 | C | T | RP11-119D9.1 |  | ENST00000533670 | 0 | None | upstream_gene_variant | LOW |  | None |  | None | 0.166666667 |
| chr11 | 67653994 | 67653995 | C | T | RP11-119D9.1 |  | ENST00000533670 | 0 | None | upstream_gene_variant | LOW |  | None |  | None | 0.155172414 |
| chr11 | 68054921 | 68054922 | T | C | None |  |  | 0 | None | intergenic_variant | LOW |  | None |  | None | 0.253731343 |
| chr11 | 68859869 | 68859870 | G | A | TPCN2 |  | ENST00000294309 | 1 | None | downstream_gene_variant | LOW |  | None |  | None | NA |
| chr11 | 68859874 | 68859875 | C | A | TPCN2 |  | ENST00000294309 | 1 | None | downstream_gene_variant | LOW |  | None |  | None | NA |
| chr11 | 69179519 | 69179520 | A | G | MYEOV |  | ENST00000541137 | 0 | None | intron_variant | LOW |  | None |  | None | 0.206896552 |
| chr11 | 70374434 | 70374435 | A | G | SHANK2 |  | ENST00000338508 | 0 | None | intron_variant | LOW |  | None |  | None | 0.126984127 |
| chr11 | 70463925 | 70463926 | C | T | SHANK2 |  | ENST00000449116 | 0 | None | intron_variant | LOW |  | None |  | None | 0.206349206 |
| chr11 | 71222126 | 71222127 | T | G | NADSYN1 |  | ENST00000527963 | 0 | None | intron_variant | LOW |  | None |  | None | 0.16 |
| chr11 | 71466278 | 71466279 | A | T | ENPP7P8 |  | ENST00000527856 | 1 | None | intron_variant | LOW |  | None |  | None | 0.177419355 |
| chr11 | 71466285 | 71466286 | C | G | ENPP7P8 |  | ENST00000527856 | 1 | None | intron_variant | LOW |  | None |  | None | 0.171875 |
| chr11 | 71747340 | 71747341 | A | G | NUMA1 |  | ENST00000543937 | 0 | None | intron_variant | LOW |  | None |  | None | 0.166666667 |
| chr11 | 72335959 | 72335960 | A | G | PDE2A |  | ENST00000544570 | 0 | None | intron_variant | LOW |  | None |  | None | 0.295774648 |
| chr11 | 74186161 | 74186162 | C | T | RP11-702H23.4 |  | ENST00000533008 | 0 | None | intron_variant | LOW |  | None |  | None | 0.206896552 |
| chr11 | 75056624 | 75056625 | A | C | ARRB1 |  | ENST00000420843 | 0 | None | intron_variant | LOW |  | None |  | None | 0.2 |
| chr11 | 75121776 | 75121777 | C | T | RPS3 |  | ENST00000527446 | 0 | None | intron_variant | LOW |  | None |  | None | 0.129032258 |
| chr11 | 75871599 | 75871600 | C | T | None |  |  | 0 | None | intergenic_variant | LOW |  | None |  | None | 0.269662921 |
| chr11 | 76046727 | 76046728 | T | C | None |  |  | 0 | None | intergenic_variant | LOW |  | None |  | None | 0.226415094 |
| chr11 | 76331914 | 76331915 | A | C | RP11-672A2.6 |  | ENST00000528781 | 0 | None | downstream_gene_variant | LOW |  | None |  | None | 0.2 |
| chr11 | 77274597 | 77274598 | G | A | CLNS1A |  | ENST00000526761 | 0 | None | intron_variant | LOW |  | None |  | None | 0.084507042 |
| chr11 | 78053477 | 78053478 | C | T | GAB2 |  | ENST00000361507 | 0 | None | intron_variant | LOW |  | None |  | None | 0.166666667 |
| chr11 | 78100357 | 78100358 | T | A | GAB2 |  | ENST00000361507 | 0 | None | intron_variant | LOW |  | None |  | None | 0.15 |
| chr11 | 78100379 | 78100380 | G | C | GAB2 |  | ENST00000530915 | 0 | None | intron_variant | LOW |  | None |  | None | 0.18 |
| chr11 | 78339247 | 78339248 | C | A | None |  |  | 0 | None | intergenic_variant | LOW |  | None |  | None | 0.175 |
| chr11 | 78440820 | 78440821 | A | G | TENM4 |  | ENST00000278550 | 0 | None | intron_variant | LOW |  | None |  | None | 0.203389831 |
| chr11 | 78476132 | 78476133 | G | T | TENM4 |  | ENST00000278550 | 0 | None | intron_variant | LOW |  | None |  | None | 0.234042553 |
| chr11 | 79206028 | 79206029 | G | A | None |  |  | 0 | None | intergenic_variant | LOW |  | None |  | None | 0.111111111 |
| chr11 | 80453573 | 80453574 | G | A | None |  |  | 0 | None | intergenic_variant | LOW |  | None |  | None | 0.25 |
| chr11 | 80531182 | 80531183 | C | G | None |  |  | 0 | None | intergenic_variant | LOW |  | None |  | None | 0.333333333 |
| chr11 | 81133239 | 81133240 | A | T | None |  |  | 0 | None | intergenic_variant | LOW |  | None |  | None | 0.265306122 |
| chr11 | 81250624 | 81250625 | T | C | None |  |  | 0 | None | intergenic_variant | LOW |  | None |  | None | 0.134615385 |
| chr11 | 81250630 | 81250631 | T | C | None |  |  | 0 | None | intergenic_variant | LOW |  | None |  | None | 0.16 |
| chr11 | 81440776 | 81440777 | G | T | None |  |  | 0 | None | intergenic_variant | LOW |  | None |  | None | 0.12244898 |
| chr11 | 81555403 | 81555404 | G | A | RP11-876F14.2 |  | ENST00000605877 | 0 | None | upstream_gene_variant | LOW |  | None |  | None | 0.269230769 |
| chr11 | 81555747 | 81555748 | A | C | RP11-876F14.2 |  | ENST00000605877 | 0 | None | upstream_gene_variant | LOW |  | None |  | None | 0.16 |
| chr11 | 81704067 | 81704068 | G | A | RP11-179A16.1 |  | ENST00000530896 | 0 | None | intron_variant | LOW |  | None |  | None | 0.224137931 |
| chr11 | 81945347 | 81945348 | T | A | RP11-179A16.1 |  | ENST00000500502 | 0 | None | intron_variant | LOW |  | None |  | None | 0.173913043 |
| chr11 | 83019751 | 83019752 | A | G | RP11-727A23.10 |  | ENST00000530045 | 0 | None | intron_variant | LOW |  | None |  | None | 0.142857143 |
| chr11 | 84826146 | 84826147 | C | T | DLG2 |  | ENST00000543673 | 0 | None | intron_variant | LOW |  | None |  | None | 0.166666667 |
| chr11 | 87430180 | 87430181 | C | A | RP11-720D4.3 |  | ENST00000532606 | 0 | None | non_coding_exon_variant | LOW |  | None |  | None | 0.211538462 |
| chr11 | 87515225 | 87515226 | A | C | None |  |  | 0 | None | intergenic_variant | LOW |  | None |  | None | 0.25 |
| chr11 | 87830963 | 87830964 | A | G | RP11-164N3.2 |  | ENST00000531454 | 0 | None | intron_variant | LOW |  | None |  | None | 0.209302326 |
| chr11 | 87890712 | 87890713 | C | G | RAB38 |  | ENST00000531138 | 0 | None | intron_variant | LOW |  | None |  | None | 0.235294118 |
| chr11 | 88076020 | 88076021 | T | G | None |  |  | 0 | None | intergenic_variant | LOW |  | None |  | None | 0.197183099 |
| chr11 | 88781564 | 88781565 | G | A | GRM5 |  | ENST00000418177 | 0 | None | intron_variant | LOW |  | None |  | None | 0.1875 |
| chr11 | 89181183 | 89181184 | G | C | NOX4 |  | ENST00000534731 | 0 | None | intron_variant | LOW |  | None |  | None | 0.245614035 |
| chr11 | 89908771 | 89908772 | G | A | NAALAD2 |  | ENST00000375944 | 0 | None | intron_variant | LOW |  | None |  | None | 0.2 |
| chr11 | 89960144 | 89960145 | C | T | CHORDC1 |  | ENST00000530765 | 0 | None | upstream_gene_variant | LOW |  | None |  | None | 0.208333333 |
| chr11 | 90259380 | 90259381 | A | T | DISC1FP1 |  | ENST00000562678 | 0 | None | intron_variant | LOW |  | None |  | None | 0.178571429 |
| chr11 | 90269636 | 90269637 | G | A | DISC1FP1 |  | ENST00000563681 | 0 | None | intron_variant | LOW |  | None |  | None | 0.254545455 |
| chr11 | 90365727 | 90365728 | A | T | DISC1FP1 |  | ENST00000562678 | 0 | None | intron_variant | LOW |  | None |  | None | 0.218181818 |
| chr11 | 90517178 | 90517179 | C | T | DISC1FP1 |  | ENST00000562678 | 0 | None | intron_variant | LOW |  | None |  | None | 0.14893617 |
| chr11 | 90597279 | 90597280 | G | T | DISC1FP1 |  | ENST00000562245 | 0 | None | intron_variant | LOW |  | None |  | None | 0.146341463 |
| chr11 | 90968823 | 90968824 | G | T | None |  |  | 0 | None | intergenic_variant | LOW |  | None |  | None | 0.239130435 |
| chr11 | 91292769 | 91292770 | A | C | None |  |  | 0 | None | intergenic_variant | LOW |  | None |  | None | 0.204545455 |
| chr11 | 91558737 | 91558738 | G | A | None |  |  | 0 | None | intergenic_variant | LOW |  | None |  | None | 0.175438596 |
| chr11 | 92332618 | 92332619 | T | C | FAT3 |  | ENST00000525166 | 0 | None | intron_variant | LOW |  | None |  | None | 0.18 |
| chr11 | 93680491 | 93680492 | C | T | None |  |  | 0 | None | intergenic_variant | LOW |  | None |  | None | 0.155555556 |
| chr11 | 93927455 | 93927456 | T | A | None |  |  | 0 | None | intergenic_variant | LOW |  | None |  | None | 0.161290323 |
| chr11 | 95200988 | 95200989 | C | T | None |  |  | 0 | None | intergenic_variant | LOW |  | None |  | None | 0.2 |
| chr11 | 95899344 | 95899345 | A | T | MAML2 |  | ENST00000524717 | 1 | None | intron_variant | LOW |  | None |  | None | 0.142857143 |
| chr11 | 96517868 | 96517869 | A | T | RP11-360K13.1 |  | ENST00000527528 | 0 | None | intron_variant | LOW |  | None |  | None | 0.209302326 |
| chr11 | 96670838 | 96670839 | T | C | None |  |  | 0 | None | intergenic_variant | LOW |  | None |  | None | 0.24 |
| chr11 | 96929967 | 96929968 | G | T | None |  |  | 0 | None | intergenic_variant | LOW |  | None |  | None | 0.213114754 |
| chr11 | 96929968 | 96929969 | G | T | None |  |  | 0 | None | intergenic_variant | LOW |  | None |  | None | 0.2 |
| chr11 | 96985197 | 96985198 | A | G | None |  |  | 0 | None | intergenic_variant | LOW |  | None |  | None | 0.20754717 |
| chr11 | 97094471 | 97094472 | A | T | RP11-882G5.1 |  | ENST00000529088 | 0 | None | intron_variant | LOW |  | None |  | None | 0.265306122 |
| chr11 | 97409302 | 97409303 | T | C | None |  |  | 0 | None | intergenic_variant | LOW |  | None |  | None | 0.178082192 |
| chr11 | 97499056 | 97499057 | G | A | None |  |  | 0 | None | intergenic_variant | LOW |  | None |  | None | 0.173913043 |
| chr11 | 97500993 | 97500994 | G | T | None |  |  | 0 | None | intergenic_variant | LOW |  | None |  | None | 0.183333333 |
| chr11 | 97658323 | 97658324 | C | A | None |  |  | 0 | None | intergenic_variant | LOW |  | None |  | None | 0.192307692 |
| chr11 | 98173840 | 98173841 | G | A | None |  |  | 0 | None | intergenic_variant | LOW |  | None |  | None | 0.285714286 |
| chr11 | 98354362 | 98354363 | T | A | None |  |  | 0 | None | intergenic_variant | LOW |  | None |  | None | 0.263157895 |
| chr11 | 98429288 | 98429289 | G | T | None |  |  | 0 | None | intergenic_variant | LOW |  | None |  | None | 0.132075472 |
| chr11 | 98495553 | 98495554 | T | C | None |  |  | 0 | None | intergenic_variant | LOW |  | None |  | None | 0.092307692 |
| chr11 | 98520081 | 98520082 | C | T | None |  |  | 0 | None | intergenic_variant | LOW |  | None |  | None | 0.224489796 |
| chr11 | 98623894 | 98623895 | C | A | None |  |  | 0 | None | intergenic_variant | LOW |  | None |  | None | 0.178571429 |
| chr11 | 98624186 | 98624187 | A | G | None |  |  | 0 | None | intergenic_variant | LOW |  | None |  | None | 0.306451613 |
| chr11 | 98681954 | 98681955 | T | G | None |  |  | 0 | None | intergenic_variant | LOW |  | None |  | None | 0.265306122 |
| chr11 | 98833460 | 98833461 | T | A | None |  |  | 0 | None | intergenic_variant | LOW |  | None |  | None | 0.142857143 |
| chr11 | 98919316 | 98919317 | C | T | CNTN5 |  | ENST00000527185 | 0 | None | intron_variant | LOW |  | None |  | None | 0.09375 |
| chr11 | 99034690 | 99034691 | C | A | CNTN5 |  | ENST00000527185 | 0 | None | intron_variant | LOW |  | None |  | None | 0.158730159 |
| chr11 | 100915251 | 100915252 | C | A | PGR |  | ENST00000534013 | 0 | None | intron_variant | LOW |  | None |  | None | 0.233333333 |
| chr11 | 101294817 | 101294818 | C | A | None |  |  | 0 | None | intergenic_variant | LOW |  | None |  | None | 0.218181818 |
| chr11 | 101414366 | 101414367 | G | T | TRPC6 |  | ENST00000360497 | 0 | None | intron_variant | LOW |  | None |  | None | 0.203389831 |
| chr11 | 102992835 | 102992836 | G | T | DYNC2H1 |  | ENST00000375735 | 0 | None | intron_variant | LOW |  | None |  | None | 0.348837209 |
| chr11 | 102992837 | 102992838 | C | A | DYNC2H1 |  | ENST00000398093 | 0 | None | intron_variant | LOW |  | None |  | None | 0.363636364 |
| chr11 | 103279330 | 103279331 | A | G | DYNC2H1 |  | ENST00000398093 | 1 | None | intron_variant | LOW |  | None |  | None | 0.63 |
| chr11 | 103301039 | 103301040 | T | C | DYNC2H1 |  | ENST00000334267 | 0 | None | intron_variant | LOW |  | None |  | None | 0.272727273 |
| chr11 | 103542623 | 103542624 | C | T | RP11-563P16.1 |  | ENST00000533459 | 0 | None | downstream_gene_variant | LOW |  | None |  | None | 0.12 |
| chr11 | 103895571 | 103895572 | T | C | PDGFD |  | ENST00000393158 | 0 | None | intron_variant | LOW |  | None |  | None | 0.243243243 |
| chr11 | 104282096 | 104282097 | G | A | None |  |  | 0 | None | intergenic_variant | LOW |  | None |  | None | 0.352941176 |
| chr11 | 104690123 | 104690124 | A | G | None |  |  | 0 | None | intergenic_variant | LOW |  | None |  | None | 0.236842105 |
| chr11 | 105096702 | 105096703 | G | T | RP11-94P11.4 |  | ENST00000528811 | 0 | None | intron_variant | LOW |  | None |  | None | 0.382352941 |
| chr11 | 105129462 | 105129463 | T | A | RP11-94P11.4 |  | ENST00000528811 | 0 | None | intron_variant | LOW |  | None |  | None | 0.151515152 |
| chr11 | 105144760 | 105144761 | G | T | RP11-94P11.4 |  | ENST00000528811 | 0 | None | intron_variant | LOW |  | None |  | None | 0.311111111 |
| chr11 | 105529582 | 105529583 | C | T | GRIA4 |  | ENST00000428631 | 0 | None | intron_variant | LOW |  | None |  | None | 0.388888889 |
| chr11 | 105734504 | 105734505 | A | G | GRIA4 |  | ENST00000393127 | 0 | None | intron_variant | LOW |  | None |  | None | 0.191489362 |
| chr11 | 105792981 | 105792982 | A | G | GRIA4 |  | ENST00000393127 | 0 | None | intron_variant | LOW |  | None |  | None | 0.183673469 |
| chr11 | 106527306 | 106527307 | G | C | None |  |  | 0 | None | intergenic_variant | LOW |  | None |  | None | 0.263157895 |
| chr11 | 106789241 | 106789242 | C | T | GUCY1A2 |  | ENST00000282249 | 0 | None | intron_variant | LOW |  | None |  | None | 0.233333333 |
| chr11 | 107628350 | 107628351 | A | T | None |  |  | 0 | None | intergenic_variant | LOW |  | None |  | None | 0.277777778 |
| chr11 | 108530189 | 108530190 | A | T | None |  |  | 1 | None | intergenic_variant | LOW |  | None |  | None | 0.139534884 |
| chr11 | 109186736 | 109186737 | G | T | None |  |  | 0 | None | intergenic_variant | LOW |  | None |  | None | 0.1875 |
| chr11 | 110375218 | 110375219 | G | T | None |  |  | 0 | None | intergenic_variant | LOW |  | None |  | None | 0.25 |
| chr11 | 110420836 | 110420837 | G | C | None |  |  | 0 | None | intergenic_variant | LOW |  | None |  | None | 0.136363636 |
| chr11 | 110872412 | 110872413 | C | T | None |  |  | 0 | None | intergenic_variant | LOW |  | None |  | None | 0.166666667 |
| chr11 | 110932206 | 110932207 | A | T | None |  |  | 0 | None | intergenic_variant | LOW |  | None |  | None | 0.2 |
| chr11 | 113289087 | 113289088 | G | C | DRD2 |  | ENST00000362072 | 0 | None | intron_variant | LOW |  | None |  | None | 0.228070175 |
| chr11 | 113347550 | 113347551 | G | A | DRD2 |  | ENST00000542616 | 0 | None | upstream_gene_variant | LOW |  | None |  | None | 0.218181818 |
| chr11 | 113875748 | 113875749 | G | T | None |  |  | 0 | None | intergenic_variant | LOW |  | None |  | None | 0.272727273 |
| chr11 | 114966563 | 114966564 | G | A | None |  |  | 0 | None | intergenic_variant | LOW |  | None |  | None | 0.133333333 |
| chr11 | 116328909 | 116328910 | C | T | None |  |  | 0 | None | intergenic_variant | LOW |  | None |  | None | 0.183673469 |
| chr11 | 116941033 | 116941034 | G | T | SIK3 |  | ENST00000375288 | 0 | None | intron_variant | LOW |  | None |  | None | 0.22 |
| chr11 | 116966120 | 116966121 | T | G | SIK3 |  | ENST00000446921 | 0 | None | intron_variant | LOW |  | None |  | None | 0.348837209 |
| chr11 | 118843481 | 118843482 | G | T | FOXR1 |  | ENST00000317011 | 0 | None | intron_variant | LOW |  | None |  | None | 0.254901961 |
| chr11 | 121560184 | 121560185 | G | T | None |  |  | 0 | None | intergenic_variant | LOW |  | None |  | None | 0.2 |
| chr11 | 121684011 | 121684012 | G | A | None |  |  | 0 | None | intergenic_variant | LOW |  | None |  | None | 0.196078431 |
| chr11 | 121728776 | 121728777 | A | T | None |  |  | 0 | None | intergenic_variant | LOW |  | None |  | None | 0.2 |
| chr11 | 121989752 | 121989753 | C | G | BLID |  | ENST00000560104 | 0 | None | upstream_gene_variant | LOW |  | None |  | None | 0.333333333 |
| chr11 | 122204070 | 122204071 | A | T | RP11-820L6.1 |  | ENST00000529733 | 0 | None | intron_variant | LOW |  | None |  | None | 0.162790698 |
| chr11 | 122689154 | 122689155 | C | T | UBASH3B |  | ENST00000284273 | 0 | None | downstream_gene_variant | LOW |  | None |  | None | 0.233333333 |
| chr11 | 122690263 | 122690264 | G | C | None |  |  | 0 | None | intergenic_variant | LOW |  | None |  | None | 0.150943396 |
| chr11 | 123496782 | 123496783 | T | A | GRAMD1B |  | ENST00000529432 | 0 | None | 3_prime_UTR_variant | LOW |  | None |  | None | 0.177419355 |
| chr11 | 123666247 | 123666248 | G | A | None |  |  | 0 | None | intergenic_variant | LOW |  | None |  | None | 0.302325581 |
| chr11 | 124856942 | 124856943 | T | A | CCDC15 |  | ENST00000344762 | 0 | None | intron_variant | LOW |  | None |  | None | 0.358208955 |
| chr11 | 125075635 | 125075636 | A | T | PKNOX2 |  | ENST00000531212 | 0 | None | intron_variant | LOW |  | None |  | None | 0.181818182 |
| chr11 | 125462261 | 125462262 | G | A | STT3A |  | ENST00000527606 | 0 | None | intron_variant | LOW |  | None |  | None | 0.228070175 |
| chr11 | 126906845 | 126906846 | C | A | RP11-168K9.1 |  | ENST00000530177 | 0 | None | intron_variant | LOW |  | None |  | None | 0.26 |
| chr11 | 127273866 | 127273867 | C | G | RN7SKP279 |  | ENST00000411383 | 0 | None | upstream_gene_variant | LOW |  | None |  | None | 0.266666667 |
| chr11 | 127323980 | 127323981 | G | A | None |  |  | 0 | None | intergenic_variant | LOW |  | None |  | None | 0.227272727 |
| chr11 | 129180496 | 129180497 | G | A | None |  |  | 0 | None | intergenic_variant | LOW |  | None |  | None | 0.228070175 |
| chr11 | 129403795 | 129403796 | G | C | RPS27P20 |  | ENST00000492051 | 0 | None | upstream_gene_variant | LOW |  | None |  | None | 0.285714286 |
| chr11 | 129614354 | 129614355 | C | T | None |  |  | 0 | None | intergenic_variant | LOW |  | None |  | None | 0.152542373 |
| chr11 | 131121328 | 131121329 | G | A | AP002856.5 |  | ENST00000606885 | 1 | None | upstream_gene_variant | LOW |  | None |  | None | 0.105263158 |
| chr11 | 131229183 | 131229184 | G | T | None |  |  | 0 | None | intergenic_variant | LOW |  | None |  | None | 0.213114754 |
| chr11 | 131311895 | 131311896 | T | C | NTM |  | ENST00000374791 | 0 | None | intron_variant | LOW |  | None |  | None | 0.26984127 |
| chr11 | 131520996 | 131520997 | C | T | NTM |  | ENST00000539799 | 0 | None | intron_variant | LOW |  | None |  | None | 0.227272727 |
| chr11 | 131523783 | 131523784 | A | T | NTM |  | ENST00000436745 | 0 | None | intron_variant | LOW |  | None |  | None | 0.259259259 |
| chr11 | 131807587 | 131807588 | T | G | NTM |  | ENST00000374791 | 0 | None | intron_variant | LOW |  | None |  | None | 0.220338983 |
| chr11 | 132087305 | 132087306 | G | T | NTM |  | ENST00000374791 | 0 | None | intron_variant | LOW |  | None |  | None | 0.192982456 |
| chr11 | 132559904 | 132559905 | G | A | OPCML |  | ENST00000524381 | 0 | None | intron_variant | LOW |  | None |  | None | 0.178571429 |
| chr11 | 133062645 | 133062646 | C | T | OPCML |  | ENST00000524381 | 0 | None | intron_variant | LOW |  | None |  | None | 0.150943396 |
| chr11 | 133283575 | 133283576 | C | T | OPCML |  | ENST00000524381 | 0 | None | intron_variant | LOW |  | None |  | None | 0.185185185 |
| chr11 | 133394090 | 133394091 | A | T | OPCML |  | ENST00000524381 | 0 | None | intron_variant | LOW |  | None |  | None | 0.25 |
| chr11 | 133464961 | 133464962 | C | A | None |  |  | 0 | None | intergenic_variant | LOW |  | None |  | None | 0.2 |
| chr11 | 133874737 | 133874738 | C | A | None |  |  | 0 | None | intergenic_variant | LOW |  | None |  | None | 0.287878788 |
| chr11 | 134489574 | 134489575 | G | A | None |  |  | 0 | None | intergenic_variant | LOW |  | None |  | None | 0.30952381 |
| chr12 | 64800 | 64801 | T | C | RP11-598F7.1 |  | ENST00000504074 | 0 | None | upstream_gene_variant | LOW |  | None |  | None | 0.102564103 |
| chr12 | 77157 | 77158 | T | C | AC215219.1 |  | ENST00000458783 | 0 | None | upstream_gene_variant | LOW |  | None |  | None | 0.082644628 |
| chr12 | 519598 | 519599 | G | A | CCDC77 |  | ENST00000422000 | 0 | None | intron_variant | LOW |  | None |  | None | 0.130434783 |
| chr12 | 1441717 | 1441718 | T | A | ERC1 |  | ENST00000543086 | 0 | None | intron_variant | LOW |  | None |  | None | 0.264150943 |
| chr12 | 2211368 | 2211369 | G | A | CACNA1C |  | ENST00000399637 | 0 | None | intron_variant | LOW |  | None |  | None | 0.272727273 |
| chr12 | 2876806 | 2876807 | C | T | RP11-885B4.2 |  | ENST00000536497 | 0 | None | intron_variant | LOW |  | None |  | None | 0.266666667 |
| chr12 | 4237774 | 4237775 | A | T | None |  |  | 0 | None | intergenic_variant | LOW |  | None |  | None | 0.224137931 |
| chr12 | 4356721 | 4356722 | C | T | RP11-264F23.3 |  | ENST00000539135 | 0 | None | downstream_gene_variant | LOW |  | None |  | None | 0.14893617 |
| chr12 | 4362886 | 4362887 | G | A | RP11-264F23.3 |  | ENST00000539135 | 0 | None | intron_variant | LOW |  | None |  | None | 0.103448276 |
| chr12 | 5272620 | 5272621 | C | A | None |  |  | 0 | None | intergenic_variant | LOW |  | None |  | None | 0.163934426 |
| chr12 | 7563347 | 7563348 | C | T | CD163L1 |  | ENST00000396630 | 0 | None | intron_variant | LOW |  | None |  | None | 0.117647059 |
| chr12 | 8100673 | 8100674 | A | C | None |  |  | 0 | None | intergenic_variant | LOW |  | None |  | None | 0.192307692 |
| chr12 | 8462980 | 8462981 | A | G | LINC00937 |  | ENST00000544461 | 1 | None | intron_variant | LOW |  | None |  | None | 0.122807018 |
| chr12 | 8462997 | 8462998 | T | C | LINC00937 |  | ENST00000544461 | 0 | None | intron_variant | LOW |  | None |  | None | 0.114754098 |
| chr12 | 8463039 | 8463040 | A | T | LINC00937 |  | ENST00000544461 | 0 | None | intron_variant | LOW |  | None |  | None | 0.111111111 |
| chr12 | 8530146 | 8530147 | C | G | LINC00937 |  | ENST00000420040 | 0 | None | intron_variant | LOW |  | None |  | None | 0.230769231 |
| chr12 | 8756741 | 8756742 | G | A | AICDA |  | ENST00000543081 | 0 | None | 3_prime_UTR_variant | LOW |  | None |  | None | 0.192982456 |
| chr12 | 10179042 | 10179043 | G | A | CLEC9A |  | ENST00000355819 | 0 | None | upstream_gene_variant | LOW |  | None |  | None | 0.267857143 |
| chr12 | 12325320 | 12325321 | T | G | LRP6 |  | ENST00000261349 | 0 | None | intron_variant | LOW |  | None |  | None | 0.196721311 |
| chr12 | 12900216 | 12900217 | C | G | APOLD1 |  | ENST00000326765 | 0 | None | intron_variant | LOW |  | None |  | None | 0.15 |
| chr12 | 12900524 | 12900525 | C | G | APOLD1 |  | ENST00000326765 | 0 | None | intron_variant | LOW |  | None |  | None | 0.172413793 |
| chr12 | 13249875 | 13249876 | T | A | GSG1 |  | ENST00000432710 | 0 | None | intron_variant | LOW |  | None |  | None | 0.12244898 |
| chr12 | 13685398 | 13685399 | C | T | RP11-4N23.1 |  | ENST00000543347 | 0 | None | intron_variant | LOW |  | None |  | None | 0.1875 |
| chr12 | 13898257 | 13898258 | C | T | GRIN2B |  | ENST00000609686 | 0 | None | intron_variant | LOW |  | None |  | None | 0.161290323 |
| chr12 | 14392215 | 14392216 | A | T | MRPS18CP4 |  | ENST00000535821 | 0 | None | downstream_gene_variant | LOW |  | None |  | None | 0.3 |
| chr12 | 15236808 | 15236809 | C | T | None |  |  | 0 | None | intergenic_variant | LOW |  | None |  | None | 0.222222222 |
| chr12 | 15472903 | 15472904 | T | C | RERG |  | ENST00000393736 | 1 | None | intron_variant | LOW |  | None |  | None | 0.295454545 |
| chr12 | 17793387 | 17793388 | G | T | None |  |  | 0 | None | intergenic_variant | LOW |  | None |  | None | 0.166666667 |
| chr12 | 18028417 | 18028418 | A | T | None |  |  | 0 | None | intergenic_variant | LOW |  | None |  | None | 0.227272727 |
| chr12 | 18102499 | 18102500 | G | T | None |  |  | 0 | None | intergenic_variant | LOW |  | None |  | None | 0.192982456 |
| chr12 | 18465145 | 18465146 | G | T | PIK3C2G |  | ENST00000266497 | 0 | None | intron_variant | LOW |  | None |  | None | 0.155555556 |
| chr12 | 18821608 | 18821609 | G | A | None |  |  | 0 | None | intergenic_variant | LOW |  | None |  | None | 0.169811321 |
| chr12 | 20640602 | 20640603 | T | A | PDE3A |  | ENST00000359062 | 1 | None | intron_variant | LOW |  | None |  | None | 0.122807018 |
| chr12 | 21571085 | 21571086 | G | A | SLCO1A2 |  | ENST00000435179 | 0 | None | intron_variant | LOW |  | None |  | None | 0.175438596 |
| chr12 | 21571086 | 21571087 | C | G | SLCO1A2 |  | ENST00000435179 | 0 | None | intron_variant | LOW |  | None |  | None | 0.175438596 |
| chr12 | 22489908 | 22489909 | C | T | ST8SIA1 |  | ENST00000381424 | 0 | None | upstream_gene_variant | LOW |  | None |  | None | 0.238095238 |
| chr12 | 23980750 | 23980751 | G | T | SOX5 |  | ENST00000545921 | 0 | None | intron_variant | LOW |  | None |  | None | 0.159090909 |
| chr12 | 24079512 | 24079513 | C | A | SOX5 |  | ENST00000538083 | 0 | None | intron_variant | LOW |  | None |  | None | 0.166666667 |
| chr12 | 24161663 | 24161664 | C | G | None |  |  | 0 | None | intergenic_variant | LOW |  | None |  | None | 0.245283019 |
| chr12 | 27341023 | 27341024 | G | T | None |  |  | 0 | None | intergenic_variant | LOW |  | None |  | None | 0.283333333 |
| chr12 | 27670551 | 27670552 | A | G | None |  |  | 0 | None | intergenic_variant | LOW |  | None |  | None | 0.359375 |
| chr12 | 28307831 | 28307832 | A | G | CCDC91 |  | ENST00000538586 | 0 | None | intron_variant | LOW |  | None |  | None | 0.218181818 |
| chr12 | 28599557 | 28599558 | A | G | CCDC91 |  | ENST00000536154 | 0 | None | intron_variant | LOW |  | None |  | None | 0.1875 |
| chr12 | 28678901 | 28678902 | T | G | CCDC91 |  | ENST00000381256 | 0 | None | intron_variant | LOW |  | None |  | None | 0.176470588 |
| chr12 | 29048145 | 29048146 | C | T | None |  |  | 0 | None | intergenic_variant | LOW |  | None |  | None | 0.105263158 |
| chr12 | 30361859 | 30361860 | G | C | RP11-776A13.1 |  | ENST00000549055 | 0 | None | intron_variant | LOW |  | None |  | None | 0.157142857 |
| chr12 | 30361864 | 30361865 | C | T | RP11-776A13.1 |  | ENST00000549055 | 1 | None | intron_variant | LOW |  | None |  | None | 0.130434783 |
| chr12 | 30854961 | 30854962 | C | A | None |  |  | 0 | None | intergenic_variant | LOW |  | None |  | None | 0.12962963 |
| chr12 | 31238097 | 31238098 | A | G | DDX11 |  | ENST00000438391 | 1 | None | intron_variant | LOW |  | None |  | None | 0.113924051 |
| chr12 | 31790440 | 31790441 | C | G | None |  |  | 0 | None | intergenic_variant | LOW |  | None |  | None | 0.189189189 |
| chr12 | 32249721 | 32249722 | C | T | RP11-843B15.3 |  | ENST00000605512 | 0 | None | downstream_gene_variant | LOW |  | None |  | None | 0.137931034 |
| chr12 | 33368533 | 33368534 | G | T | None |  |  | 0 | None | intergenic_variant | LOW |  | None |  | None | 0.206896552 |
| chr12 | 33852204 | 33852205 | G | A | None |  |  | 0 | None | intergenic_variant | LOW |  | None |  | None | 0.158730159 |
| chr12 | 33853965 | 33853966 | C | A | None |  |  | 0 | None | intergenic_variant | LOW |  | None |  | None | 0.272727273 |
| chr12 | 33915372 | 33915373 | C | G | None |  |  | 0 | None | intergenic_variant | LOW |  | None |  | None | 0.195652174 |
| chr12 | 34016419 | 34016420 | C | T | None |  |  | 0 | None | intergenic_variant | LOW |  | None |  | None | 0.188679245 |
| chr12 | 34228562 | 34228563 | T | G | None |  |  | 0 | None | intergenic_variant | LOW |  | None |  | None | 0.12 |
| chr12 | 34324038 | 34324039 | C | T | TUBB8P4 |  | ENST00000511806 | 0 | None | upstream_gene_variant | LOW |  | None |  | None | 0.0875 |
| chr12 | 34324080 | 34324081 | G | A | TUBB8P4 |  | ENST00000511806 | 0 | None | upstream_gene_variant | LOW |  | None |  | None | 0.108433735 |
| chr12 | 34324103 | 34324104 | T | C | TUBB8P4 |  | ENST00000511806 | 0 | None | upstream_gene_variant | LOW |  | None |  | None | 0.08988764 |
| chr12 | 34324128 | 34324129 | C | A | TUBB8P4 |  | ENST00000511806 | 0 | None | upstream_gene_variant | LOW |  | None |  | None | 0.085106383 |
| chr12 | 34446952 | 34446953 | C | T | None |  |  | 0 | None | intergenic_variant | LOW |  | None |  | None | 0.208333333 |
| chr12 | 34466648 | 34466649 | C | A | None |  |  | 0 | None | intergenic_variant | LOW |  | None |  | None | 0.112676056 |
| chr12 | 34508775 | 34508776 | T | G | None |  |  | 0 | None | intergenic_variant | LOW |  | None |  | None | 0.174603175 |
| chr12 | 34751861 | 34751862 | G | T | None |  |  | 0 | None | intergenic_variant | LOW |  | None |  | None | 0.191489362 |
| chr12 | 37864117 | 37864118 | G | A | None |  |  | 0 | None | intergenic_variant | LOW |  | None |  | None | 0.136363636 |
| chr12 | 37864119 | 37864120 | A | G | None |  |  | 0 | None | intergenic_variant | LOW |  | None |  | None | 0.138461538 |
| chr12 | 37876604 | 37876605 | C | T | None |  |  | 0 | None | intergenic_variant | LOW |  | None |  | None | 0.06557377 |
| chr12 | 37877993 | 37877994 | C | A | None |  |  | 0 | None | intergenic_variant | LOW |  | None |  | None | 0.070175439 |
| chr12 | 37900420 | 37900421 | A | G | None |  |  | 0 | None | intergenic_variant | LOW |  | None |  | None | 0.194444444 |
| chr12 | 37907644 | 37907645 | T | C | None |  |  | 1 | None | intergenic_variant | LOW |  | None |  | None | 0.314285714 |
| chr12 | 38144218 | 38144219 | T | C | None |  |  | 0 | None | intergenic_variant | LOW |  | None |  | None | 0.106060606 |
| chr12 | 38177876 | 38177877 | T | C | None |  |  | 1 | None | intergenic_variant | LOW |  | None |  | None | 0.117647059 |
| chr12 | 38177877 | 38177878 | G | C | None |  |  | 1 | None | intergenic_variant | LOW |  | None |  | None | 0.12 |
| chr12 | 38296114 | 38296115 | C | A | None |  |  | 0 | None | intergenic_variant | LOW |  | None |  | None | 0.166666667 |
| chr12 | 38323601 | 38323602 | C | T | None |  |  | 1 | None | intergenic_variant | LOW |  | None |  | None | 0.222222222 |
| chr12 | 38457028 | 38457029 | T | G | None |  |  | 0 | None | intergenic_variant | LOW |  | None |  | None | 0.14 |
| chr12 | 38457028 | 38457029 | T | A | None |  |  | 0 | None | intergenic_variant | LOW |  | None |  | None | 0.18 |
| chr12 | 38550478 | 38550479 | G | T | RNA5SP358 |  | ENST00000362973 | 0 | None | downstream_gene_variant | LOW |  | None |  | None | 0.131147541 |
| chr12 | 38604055 | 38604056 | T | G | RP11-266O15.4 |  | ENST00000548633 | 0 | None | upstream_gene_variant | LOW |  | None |  | None | 0.1875 |
| chr12 | 38623495 | 38623496 | C | T | None |  |  | 0 | None | intergenic_variant | LOW |  | None |  | None | 0.181818182 |
| chr12 | 38728740 | 38728741 | C | T | None |  |  | 0 | None | intergenic_variant | LOW |  | None |  | None | 0.20754717 |
| chr12 | 39901992 | 39901993 | G | A | None |  |  | 0 | None | intergenic_variant | LOW |  | None |  | None | 0.196969697 |
| chr12 | 40951041 | 40951042 | G | A | MUC19 |  | ENST00000542482 | 0 | None | intron_variant | LOW |  | None |  | None | 0.288888889 |
| chr12 | 41810680 | 41810681 | G | T | PDZRN4 |  | ENST00000402685 | 0 | None | intron_variant | LOW |  | None |  | None | 0.215686275 |
| chr12 | 41888157 | 41888158 | T | C | PDZRN4 |  | ENST00000298919 | 0 | None | intron_variant | LOW |  | None |  | None | 0.267857143 |
| chr12 | 41888304 | 41888305 | G | T | PDZRN4 |  | ENST00000298919 | 0 | None | intron_variant | LOW |  | None |  | None | 0.2 |
| chr12 | 42236318 | 42236319 | C | T | RP11-630C16.2 |  | ENST00000550874 | 0 | None | intron_variant | LOW |  | None |  | None | 0.14893617 |
| chr12 | 42266257 | 42266258 | T | C | RP11-630C16.2 |  | ENST00000550874 | 0 | None | intron_variant | LOW |  | None |  | None | 0.193548387 |
| chr12 | 42700946 | 42700947 | T | C | PPHLN1 |  | ENST00000549190 | 0 | None | intron_variant | LOW |  | None |  | None | 0.125 |
| chr12 | 42775808 | 42775809 | T | C | PPHLN1 |  | ENST00000552761 | 0 | None | intron_variant | LOW |  | None |  | None | 0.169491525 |
| chr12 | 43403114 | 43403115 | G | C | None |  |  | 0 | None | intergenic_variant | LOW |  | None |  | None | 0.166666667 |
| chr12 | 43891200 | 43891201 | G | T | ADAMTS20 |  | ENST00000389420 | 0 | None | intron_variant | LOW |  | None |  | None | 0.255813953 |
| chr12 | 43911894 | 43911895 | A | C | ADAMTS20 |  | ENST00000553158 | 0 | None | intron_variant | LOW |  | None |  | None | 0.157894737 |
| chr12 | 43933163 | 43933164 | T | G | ADAMTS20 |  | ENST00000553158 | 0 | None | intron_variant | LOW |  | None |  | None | 0.176470588 |
| chr12 | 47015709 | 47015710 | G | C | RP11-446N19.1 |  | ENST00000607353 | 0 | None | intron_variant | LOW |  | None |  | None | 0.160714286 |
| chr12 | 48603883 | 48603884 | C | A | None |  |  | 1 | None | intergenic_variant | LOW |  | None |  | None | 0.160714286 |
| chr12 | 48603887 | 48603888 | G | A | None |  |  | 1 | None | intergenic_variant | LOW |  | None |  | None | 0.157894737 |
| chr12 | 49122219 | 49122220 | G | A | LINC00935 |  | ENST00000548380 | 0 | None | intron_variant | LOW |  | None |  | None | 0.283018868 |
| chr12 | 50062318 | 50062319 | G | A | FMNL3 | P/S | ENST00000293590 | 0 | None | missense_variant | MED | probably_damaging | 0.998 | deleterious | 0 | 0.203703704 |
| chr12 | 50763703 | 50763704 | G | C | FAM186A |  | ENST00000543111 | 0 | None | intron_variant | LOW |  | None |  | None | 0.130434783 |
| chr12 | 50763745 | 50763746 | A | G | FAM186A |  | ENST00000543111 | 0 | None | intron_variant | LOW |  | None |  | None | 0.107142857 |
| chr12 | 52300807 | 52300808 | A | C | ACVRL1 |  | ENST00000551576 | 0 | None | intron_variant | LOW |  | None |  | None | 0.309090909 |
| chr12 | 52621763 | 52621764 | C | A | KRT7 |  | ENST00000546666 | 0 | None | upstream_gene_variant | LOW |  | None |  | None | 0.225806452 |
| chr12 | 52621784 | 52621785 | G | C | KRT7 |  | ENST00000546666 | 1 | None | upstream_gene_variant | LOW |  | None |  | None | 0.275862069 |
| chr12 | 53288303 | 53288304 | A | G | KRT8 |  | ENST00000546897 | 0 | None | downstream_gene_variant | LOW |  | None |  | None | 0.22 |
| chr12 | 53661087 | 53661088 | T | C | ESPL1 |  | ENST00000257934 | 0 | None | upstream_gene_variant | LOW |  | None |  | None | 0.115384615 |
| chr12 | 53755225 | 53755226 | C | T | None |  |  | 0 | None | intergenic_variant | LOW |  | None |  | None | 0.164556962 |
| chr12 | 53857262 | 53857263 | A | C | PCBP2 |  | ENST00000603815 | 0 | None | intron_variant | LOW |  | None |  | None | 0.197183099 |
| chr12 | 54248066 | 54248067 | T | C | None |  |  | 0 | None | intergenic_variant | LOW |  | None |  | None | 0.203389831 |
| chr12 | 54456915 | 54456916 | T | A | RP11-834C11.3 |  | ENST00000515617 | 1 | None | intron_variant | LOW |  | None |  | None | 0.169811321 |
| chr12 | 54875955 | 54875956 | C | A | RP11-753H16.3 |  | ENST00000550474 | 0 | None | intron_variant | LOW |  | None |  | None | 0.170212766 |
| chr12 | 55605287 | 55605288 | G | A | None |  |  | 1 | None | intergenic_variant | LOW |  | None |  | None | 0.09375 |
| chr12 | 55707650 | 55707651 | A | C | OR6C72P |  | ENST00000379669 | 0 | None | downstream_gene_variant | LOW |  | None |  | None | 0.213114754 |
| chr12 | 56597712 | 56597713 | A | T | RNF41 |  | ENST00000549038 | 0 | None | downstream_gene_variant | LOW |  | None |  | None | 0.117647059 |
| chr12 | 56597721 | 56597722 | C | G | RNF41 |  | ENST00000552244 | 0 | None | downstream_gene_variant | LOW |  | None |  | None | 0.122807018 |
| chr12 | 57456563 | 57456564 | T | C | TMEM194A |  | ENST00000300128 | 0 | None | intron_variant | LOW |  | None |  | None | 0.150943396 |
| chr12 | 57957930 | 57957931 | G | A | KIF5A | R/Q | ENST00000455537 | 0 | COSM169125,COSM694954 | missense_variant | MED | probably_damaging | 1 | deleterious | 0 | 0.232142857 |
| chr12 | 59296849 | 59296850 | C | T | LRIG3 |  | ENST00000379141 | 0 | None | intron_variant | LOW |  | None |  | None | 0.206349206 |
| chr12 | 59893984 | 59893985 | A | G | None |  |  | 0 | None | intergenic_variant | LOW |  | None |  | None | 0.240740741 |
| chr12 | 60697733 | 60697734 | T | C | None |  |  | 0 | None | intergenic_variant | LOW |  | None |  | None | 0.162162162 |
| chr12 | 60987608 | 60987609 | A | C | None |  |  | 0 | None | intergenic_variant | LOW |  | None |  | None | 0.203703704 |
| chr12 | 62405546 | 62405547 | A | G | FAM19A2 |  | ENST00000551449 | 1 | None | intron_variant | LOW |  | None |  | None | 0.089552239 |
| chr12 | 63135670 | 63135671 | A | G | PPM1H |  | ENST00000228705 | 0 | None | intron_variant | LOW |  | None |  | None | 0.197530864 |
| chr12 | 63796347 | 63796348 | A | G | None |  |  | 0 | None | intergenic_variant | LOW |  | None |  | None | 0.109375 |
| chr12 | 63796348 | 63796349 | A | G | None |  |  | 0 | None | intergenic_variant | LOW |  | None |  | None | 0.109375 |
| chr12 | 65663254 | 65663255 | C | T | None |  |  | 0 | None | intergenic_variant | LOW |  | None |  | None | 0.169491525 |
| chr12 | 65942256 | 65942257 | A | G | RP11-230G5.2 |  | ENST00000537250 | 0 | None | intron_variant | LOW |  | None |  | None | 0.139784946 |
| chr12 | 66723776 | 66723777 | G | T | HELB |  | ENST00000247815 | 0 | None | intron_variant | LOW |  | None |  | None | 0.224489796 |
| chr12 | 66791539 | 66791540 | C | T | GRIP1 |  | ENST00000398016 | 0 | None | intron_variant | LOW |  | None |  | None | 0.19047619 |
| chr12 | 66998791 | 66998792 | A | T | GRIP1 |  | ENST00000545666 | 0 | None | intron_variant | LOW |  | None |  | None | 0.333333333 |
| chr12 | 68115366 | 68115367 | G | C | RP11-43N5.1 |  | ENST00000542219 | 0 | None | intron_variant | LOW |  | None |  | None | 0.203389831 |
| chr12 | 68460230 | 68460231 | T | G | IFNG-AS1 |  | ENST00000536914 | 0 | None | intron_variant | LOW |  | None |  | None | 0.188679245 |
| chr12 | 69212127 | 69212128 | A | G | MDM2 |  | ENST00000539479 | 0 | None | intron_variant | LOW |  | None |  | None | 0.12962963 |
| chr12 | 70417137 | 70417138 | C | T | RP11-611E13.2 |  | ENST00000549419 | 0 | None | intron_variant | LOW |  | None |  | None | 0.147540984 |
| chr12 | 71041080 | 71041081 | G | A | PTPRR |  | ENST00000378778 | 0 | None | intron_variant | LOW |  | None |  | None | 0.222222222 |
| chr12 | 72629575 | 72629576 | G | A | TRHDE |  | ENST00000548156 | 0 | None | intron_variant | LOW |  | None |  | None | 0.204545455 |
| chr12 | 73358482 | 73358483 | T | G | None |  |  | 0 | None | intergenic_variant | LOW |  | None |  | None | 0.174603175 |
| chr12 | 73543986 | 73543987 | C | T | None |  |  | 0 | None | intergenic_variant | LOW |  | None |  | None | 0.215686275 |
| chr12 | 73697133 | 73697134 | T | C | None |  |  | 0 | None | intergenic_variant | LOW |  | None |  | None | 0.235294118 |
| chr12 | 73786101 | 73786102 | T | C | None |  |  | 0 | None | intergenic_variant | LOW |  | None |  | None | 0.244897959 |
| chr12 | 74005516 | 74005517 | C | T | None |  |  | 0 | None | intergenic_variant | LOW |  | None |  | None | 0.2 |
| chr12 | 74655926 | 74655927 | C | T | RP11-274M17.3 |  | ENST00000552046 | 0 | None | intron_variant | LOW |  | None |  | None | 0.25 |
| chr12 | 75189832 | 75189833 | G | A | None |  |  | 0 | None | intergenic_variant | LOW |  | None |  | None | 0.188679245 |
| chr12 | 75782345 | 75782346 | T | C | CAPS2 |  | ENST00000442339 | 0 | None | intron_variant | LOW |  | None |  | None | 0.192307692 |
| chr12 | 76728518 | 76728519 | C | T | None |  |  | 0 | None | intergenic_variant | LOW |  | None |  | None | 0.298245614 |
| chr12 | 77369107 | 77369108 | T | A | None |  |  | 0 | None | intergenic_variant | LOW |  | None |  | None | 0.186046512 |
| chr12 | 78042434 | 78042435 | T | C | None |  |  | 0 | None | intergenic_variant | LOW |  | None |  | None | 0.235294118 |
| chr12 | 78130037 | 78130038 | T | C | None |  |  | 0 | None | intergenic_variant | LOW |  | None |  | None | 0.15 |
| chr12 | 78813888 | 78813889 | A | T | RP11-754N21.1 |  | ENST00000552230 | 0 | None | intron_variant | LOW |  | None |  | None | 0.25 |
| chr12 | 78976490 | 78976491 | A | T | None |  |  | 0 | None | intergenic_variant | LOW |  | None |  | None | 0.14 |
| chr12 | 80355288 | 80355289 | A | G | None |  |  | 0 | None | intergenic_variant | LOW |  | None |  | None | 0.185185185 |
| chr12 | 80669907 | 80669908 | C | A | OTOGL |  | ENST00000458043 | 0 | None | intron_variant | LOW |  | None |  | None | 0.166666667 |
| chr12 | 82121687 | 82121688 | C | G | PPFIA2 |  | ENST00000552948 | 0 | None | intron_variant | LOW |  | None |  | None | 0.346153846 |
| chr12 | 82288571 | 82288572 | G | A | None |  |  | 0 | None | intergenic_variant | LOW |  | None |  | None | 0.1875 |
| chr12 | 83789038 | 83789039 | T | C | None |  |  | 0 | None | intergenic_variant | LOW |  | None |  | None | 0.177419355 |
| chr12 | 84466397 | 84466398 | G | A | None |  |  | 0 | None | intergenic_variant | LOW |  | None |  | None | 0.282608696 |
| chr12 | 84638577 | 84638578 | G | C | None |  |  | 0 | None | intergenic_variant | LOW |  | None |  | None | 0.166666667 |
| chr12 | 84889441 | 84889442 | T | C | None |  |  | 0 | None | intergenic_variant | LOW |  | None |  | None | 0.175438596 |
| chr12 | 84941258 | 84941259 | G | T | None |  |  | 0 | None | intergenic_variant | LOW |  | None |  | None | 0.244897959 |
| chr12 | 85336976 | 85336977 | A | C | RP11-1079J22.1 |  | ENST00000549459 | 0 | None | upstream_gene_variant | LOW |  | None |  | None | 0.166666667 |
| chr12 | 85829410 | 85829411 | C | A | None |  |  | 0 | None | intergenic_variant | LOW |  | None |  | None | 0.234042553 |
| chr12 | 85948514 | 85948515 | G | A | None |  |  | 0 | None | intergenic_variant | LOW |  | None |  | None | 0.133333333 |
| chr12 | 86626836 | 86626837 | G | T | MGAT4C |  | ENST00000549405 | 0 | None | intron_variant | LOW |  | None |  | None | 0.178571429 |
| chr12 | 87033245 | 87033246 | A | T | MGAT4C |  | ENST00000549405 | 0 | None | intron_variant | LOW |  | None |  | None | 0.181818182 |
| chr12 | 87207137 | 87207138 | G | T | MGAT4C |  | ENST00000549405 | 0 | None | intron_variant | LOW |  | None |  | None | 0.081081081 |
| chr12 | 87547401 | 87547402 | C | T | None |  |  | 0 | None | intergenic_variant | LOW |  | None |  | None | 0.229166667 |
| chr12 | 88079262 | 88079263 | T | C | None |  |  | 0 | None | intergenic_variant | LOW |  | None |  | None | 0.106382979 |
| chr12 | 88329967 | 88329968 | C | T | None |  |  | 0 | None | intergenic_variant | LOW |  | None |  | None | 0.204081633 |
| chr12 | 89311632 | 89311633 | G | C | None |  |  | 0 | None | intergenic_variant | LOW |  | None |  | None | 0.14516129 |
| chr12 | 89618806 | 89618807 | A | C | RP11-13A1.3 |  | ENST00000549278 | 0 | None | intron_variant | LOW |  | None |  | None | 0.24137931 |
| chr12 | 89922531 | 89922532 | C | T | POC1B |  | ENST00000549504 | 0 | None | upstream_gene_variant | LOW |  | None |  | None | 0.149253731 |
| chr12 | 90415555 | 90415556 | A | T | None |  |  | 0 | None | intergenic_variant | LOW |  | None |  | None | 0.133333333 |
| chr12 | 90819010 | 90819011 | A | C | None |  |  | 0 | None | intergenic_variant | LOW |  | None |  | None | 0.3125 |
| chr12 | 91897460 | 91897461 | T | C | None |  |  | 0 | None | intergenic_variant | LOW |  | None |  | None | 0.098360656 |
| chr12 | 95885609 | 95885610 | T | C | METAP2 |  | ENST00000546753 | 0 | None | intron_variant | LOW |  | None |  | None | 0.123076923 |
| chr12 | 96355773 | 96355774 | G | A | AMDHD1 |  | ENST00000266736 | 0 | None | intron_variant | LOW |  | None |  | None | 0.081081081 |
| chr12 | 97051306 | 97051307 | A | T | C12orf55 |  | ENST00000524981 | 0 | None | intron_variant | LOW |  | None |  | None | 0.127272727 |
| chr12 | 98368917 | 98368918 | A | G | None |  |  | 0 | None | intergenic_variant | LOW |  | None |  | None | 0.255319149 |
| chr12 | 98639000 | 98639001 | T | A | RP11-690J15.1 |  | ENST00000548344 | 0 | None | intron_variant | LOW |  | None |  | None | 0.130434783 |
| chr12 | 99576462 | 99576463 | G | A | ANKS1B |  | ENST00000550778 | 0 | None | intron_variant | LOW |  | None |  | None | 0.255319149 |
| chr12 | 99728211 | 99728212 | T | G | ANKS1B |  | ENST00000547776 | 0 | None | intron_variant | LOW |  | None |  | None | 0.224489796 |
| chr12 | 100161958 | 100161959 | T | G | ANKS1B |  | ENST00000547776 | 0 | None | intron_variant | LOW |  | None |  | None | 0.235294118 |
| chr12 | 103096263 | 103096264 | A | T | None |  |  | 0 | None | intergenic_variant | LOW |  | None |  | None | 0.150943396 |
| chr12 | 103389530 | 103389531 | A | G | None |  |  | 0 | None | intergenic_variant | LOW |  | None |  | None | 0.111111111 |
| chr12 | 105424289 | 105424290 | A | C | ALDH1L2 |  | ENST00000258494 | 0 | None | intron_variant | LOW |  | None |  | None | 0.164179104 |
| chr12 | 108485564 | 108485565 | G | T | None |  |  | 0 | None | intergenic_variant | LOW |  | None |  | None | 0.229508197 |
| chr12 | 108904534 | 108904535 | T | C | FICD |  | ENST00000552695 | 0 | None | upstream_gene_variant | LOW |  | None |  | None | 0.117647059 |
| chr12 | 109274167 | 109274168 | G | A | DAO |  | ENST00000547166 | 0 | None | splice_donor_variant | HIGH |  | None |  | None | 0.228070175 |
| chr12 | 109594022 | 109594023 | T | G | ACACB |  | ENST00000338432 | 0 | None | intron_variant | LOW |  | None |  | None | 0.142857143 |
| chr12 | 109722650 | 109722651 | C | G | FOXN4 |  | ENST00000468516 | 0 | None | intron_variant | LOW |  | None |  | None | 0.203125 |
| chr12 | 109767708 | 109767709 | C | A | None |  |  | 0 | None | intergenic_variant | LOW |  | None |  | None | 0.295081967 |
| chr12 | 110610777 | 110610778 | T | C | IFT81 |  | ENST00000552912 | 0 | None | intron_variant | LOW |  | None |  | None | 0.153846154 |
| chr12 | 112051042 | 112051043 | G | T | None |  |  | 0 | None | intergenic_variant | LOW |  | None |  | None | 0.101694915 |
| chr12 | 113021663 | 113021664 | C | A | RPH3A |  | ENST00000546703 | 0 | None | intron_variant | LOW |  | None |  | None | 0.134615385 |
| chr12 | 113456370 | 113456371 | G | A | RP1-71H24.1 |  | ENST00000552784 | 0 | None | upstream_gene_variant | LOW |  | None |  | None | 0.098591549 |
| chr12 | 113456409 | 113456410 | C | T | RP1-71H24.1 |  | ENST00000552784 | 1 | None | upstream_gene_variant | LOW |  | None |  | None | 0.132075472 |
| chr12 | 113794955 | 113794956 | C | A | SLC8B1 |  | ENST00000549372 | 0 | None | intron_variant | LOW |  | None |  | None | 0.131147541 |
| chr12 | 114166549 | 114166550 | C | T | RP11-438N16.1 |  | ENST00000550905 | 0 | None | intron_variant | LOW |  | None |  | None | 0.153846154 |
| chr12 | 114242988 | 114242989 | C | A | None |  |  | 0 | None | intergenic_variant | LOW |  | None |  | None | 0.204081633 |
| chr12 | 114607262 | 114607263 | G | T | None |  |  | 0 | None | intergenic_variant | LOW |  | None |  | None | 0.150943396 |
| chr12 | 117211394 | 117211395 | G | A | RNFT2 |  | ENST00000257575 | 0 | None | intron_variant | LOW |  | None |  | None | 0.235294118 |
| chr12 | 117702964 | 117702965 | T | A | NOS1 |  | ENST00000344089 | 0 | None | intron_variant | LOW |  | None |  | None | 0.206349206 |
| chr12 | 117845517 | 117845518 | T | G | NOS1 |  | ENST00000549189 | 0 | None | intron_variant | LOW |  | None |  | None | 0.136363636 |
| chr12 | 117973542 | 117973543 | T | A | KSR2 |  | ENST00000302438 | 0 | None | intron_variant | LOW |  | None |  | None | 0.15 |
| chr12 | 117973543 | 117973544 | A | T | KSR2 |  | ENST00000302438 | 0 | None | intron_variant | LOW |  | None |  | None | 0.140350877 |
| chr12 | 118436556 | 118436557 | A | G | None |  |  | 0 | None | intergenic_variant | LOW |  | None |  | None | 0.102941176 |
| chr12 | 118946798 | 118946799 | C | T | None |  |  | 0 | None | intergenic_variant | LOW |  | None |  | None | 0.116666667 |
| chr12 | 119034829 | 119034830 | C | T | None |  |  | 0 | None | intergenic_variant | LOW |  | None |  | None | 0.212121212 |
| chr12 | 119331876 | 119331877 | T | A | RP11-357K6.3 |  | ENST00000544515 | 0 | None | intron_variant | LOW |  | None |  | None | 0.235294118 |
| chr12 | 119927662 | 119927663 | C | T | CCDC60 |  | ENST00000327554 | 0 | None | intron_variant | LOW |  | None |  | None | 0.132075472 |
| chr12 | 120447782 | 120447783 | C | T | CCDC64 |  | ENST00000397558 | 0 | None | intron_variant | LOW |  | None |  | None | 0.186440678 |
| chr12 | 120969635 | 120969636 | C | A | COQ5 |  | ENST00000551769 | 0 | None | intron_variant | LOW |  | None |  | None | 0.160714286 |
| chr12 | 121438109 | 121438110 | C | T | HNF1A |  | ENST00000544413 | 0 | None | intron_variant | LOW |  | None |  | None | 0.188679245 |
| chr12 | 121716781 | 121716782 | T | C | CAMKK2 |  | ENST00000446440 | 0 | None | intron_variant | LOW |  | None |  | None | 0.14 |
| chr12 | 123900961 | 123900962 | A | C | RILPL2 |  | ENST00000280571 | 0 | None | intron_variant | LOW |  | None |  | None | 0.183333333 |
| chr12 | 124032280 | 124032281 | A | T | None |  |  | 0 | None | intergenic_variant | LOW |  | None |  | None | 0.211538462 |
| chr12 | 124235785 | 124235786 | G | C | ATP6V0A2 |  | ENST00000330342 | 0 | None | intron_variant | LOW |  | None |  | None | 0.177419355 |
| chr12 | 124577196 | 124577197 | G | A | FAM101A |  | ENST00000540762 | 0 | None | intron_variant | LOW |  | None |  | None | 0.139534884 |
| chr12 | 124736617 | 124736618 | T | G | FAM101A |  | ENST00000540762 | 0 | None | intron_variant | LOW |  | None |  | None | 0.259259259 |
| chr12 | 124736628 | 124736629 | G | C | FAM101A |  | ENST00000540762 | 0 | None | intron_variant | LOW |  | None |  | None | 0.26 |
| chr12 | 124743542 | 124743543 | C | T | FAM101A |  | ENST00000540762 | 0 | None | intron_variant | LOW |  | None |  | None | 0.208333333 |
| chr12 | 124962969 | 124962970 | C | G | NCOR2 |  | ENST00000397355 | 0 | None | intron_variant | LOW |  | None |  | None | 0.192982456 |
| chr12 | 125116888 | 125116889 | G | A | None |  |  | 0 | None | intergenic_variant | LOW |  | None |  | None | 0.131147541 |
| chr12 | 125824756 | 125824757 | G | A | TMEM132B |  | ENST00000299308 | 0 | None | intron_variant | LOW |  | None |  | None | 0.28358209 |
| chr12 | 126148361 | 126148362 | A | G | TMEM132B |  | ENST00000299308 | 0 | None | downstream_gene_variant | LOW |  | None |  | None | 0.153846154 |
| chr12 | 126383499 | 126383500 | T | C | RP4-800O15.3 |  | ENST00000604518 | 0 | None | downstream_gene_variant | LOW |  | None |  | None | 0.271186441 |
| chr12 | 126989576 | 126989577 | C | T | None |  |  | 0 | None | intergenic_variant | LOW |  | None |  | None | 0.105263158 |
| chr12 | 127246167 | 127246168 | C | G | LINC00944 |  | ENST00000540684 | 0 | None | intron_variant | LOW |  | None |  | None | 0.140625 |
| chr12 | 127271766 | 127271767 | G | T | None |  |  | 0 | None | intergenic_variant | LOW |  | None |  | None | 0.2 |
| chr12 | 127585067 | 127585068 | T | C | None |  |  | 0 | None | intergenic_variant | LOW |  | None |  | None | 0.208333333 |
| chr12 | 127619731 | 127619732 | G | A | None |  |  | 0 | None | intergenic_variant | LOW |  | None |  | None | 0.18 |
| chr12 | 127650674 | 127650675 | G | C | AC079949.1 |  | ENST00000488123 | 1 | None | upstream_gene_variant | LOW |  | None |  | None | 0.76 |
| chr12 | 127780101 | 127780102 | C | T | None |  |  | 0 | None | intergenic_variant | LOW |  | None |  | None | 0.111111111 |
| chr12 | 127783280 | 127783281 | C | T | None |  |  | 0 | None | intergenic_variant | LOW |  | None |  | None | 0.175438596 |
| chr12 | 127783287 | 127783288 | C | A | None |  |  | 0 | None | intergenic_variant | LOW |  | None |  | None | 0.192307692 |
| chr12 | 127783312 | 127783313 | A | C | None |  |  | 0 | None | intergenic_variant | LOW |  | None |  | None | 0.224489796 |
| chr12 | 128038153 | 128038154 | G | A | None |  |  | 0 | None | intergenic_variant | LOW |  | None |  | None | 0.195121951 |
| chr12 | 128601844 | 128601845 | T | A | RP13-653N12.1 |  | ENST00000536341 | 1 | None | intron_variant | LOW |  | None |  | None | 0.20754717 |
| chr12 | 128796612 | 128796613 | C | T | TMEM132C |  | ENST00000435159 | 0 | None | intron_variant | LOW |  | None |  | None | 0.317073171 |
| chr12 | 128898966 | 128898967 | T | A | TMEM132C |  | ENST00000435159 | 0 | None | intron_variant | LOW |  | None |  | None | 0.142857143 |
| chr12 | 128978346 | 128978347 | C | A | TMEM132C |  | ENST00000435159 | 0 | None | intron_variant | LOW |  | None |  | None | 0.146341463 |
| chr12 | 130162459 | 130162460 | A | T | TMEM132D |  | ENST00000422113 | 0 | None | intron_variant | LOW |  | None |  | None | 0.228070175 |
| chr12 | 130506215 | 130506216 | C | A | RP11-474D1.4 |  | ENST00000561864 | 0 | None | downstream_gene_variant | LOW |  | None |  | None | 0.231884058 |
| chr12 | 130564738 | 130564739 | A | C | None |  |  | 0 | None | intergenic_variant | LOW |  | None |  | None | 0.171428571 |
| chr12 | 130800410 | 130800411 | G | C | None |  |  | 0 | None | intergenic_variant | LOW |  | None |  | None | 0.142857143 |
| chr12 | 130986339 | 130986340 | C | A | RIMBP2 |  | ENST00000535703 | 0 | None | intron_variant | LOW |  | None |  | None | 0.105263158 |
| chr12 | 131451527 | 131451528 | A | T | GPR133 |  | ENST00000542091 | 0 | None | intron_variant | LOW |  | None |  | None | 0.127272727 |
| chr12 | 131475820 | 131475821 | C | A | GPR133 |  | ENST00000542091 | 0 | None | intron_variant | LOW |  | None |  | None | 0.237288136 |
| chr12 | 131502934 | 131502935 | G | A | GPR133 |  | ENST00000376682 | 0 | None | intron_variant | LOW |  | None |  | None | 0.144927536 |
| chr12 | 131690577 | 131690578 | C | T | RP11-638F5.1 |  | ENST00000376678 | 0 | None | intron_variant | LOW |  | None |  | None | 0.16 |
| chr12 | 132023695 | 132023696 | C | T | None |  |  | 0 | None | intergenic_variant | LOW |  | None |  | None | 0.181818182 |
| chr12 | 133574040 | 133574041 | A | G | ZNF26 |  | ENST00000540238 | 0 | None | intron_variant | LOW |  | None |  | None | 0.168831169 |
| chr12 | 133648834 | 133648835 | C | T | None |  |  | 0 | None | intergenic_variant | LOW |  | None |  | None | 0.142857143 |
| chr13 | 19100912 | 19100913 | G | T | None |  |  | 0 | None | intergenic_variant | LOW |  | None |  | None | 0.102040816 |
| chr13 | 19100940 | 19100941 | C | T | None |  |  | 0 | None | intergenic_variant | LOW |  | None |  | None | 0.111111111 |
| chr13 | 19428483 | 19428484 | C | T | ANKRD20A9P |  | ENST00000457997 | 1 | None | intron_variant | LOW |  | None |  | None | 0.117647059 |
| chr13 | 19446858 | 19446859 | C | A | RNU6-55P |  | ENST00000411092 | 1 | None | upstream_gene_variant | LOW |  | None |  | None | 0.056910569 |
| chr13 | 19728718 | 19728719 | A | T | SMPD4P2 |  | ENST00000440167 | 0 | None | intron_variant | LOW |  | None |  | None | 0.229508197 |
| chr13 | 19728719 | 19728720 | G | T | SMPD4P2 |  | ENST00000440167 | 0 | None | intron_variant | LOW |  | None |  | None | 0.245901639 |
| chr13 | 19865877 | 19865878 | T | C | ANKRD26P3 |  | ENST00000454044 | 0 | None | intron_variant | LOW |  | None |  | None | 0.191489362 |
| chr13 | 20025982 | 20025983 | G | A | TPTE2 |  | ENST00000390680 | 1 | None | intron_variant | LOW |  | None |  | None | 0.181818182 |
| chr13 | 20351093 | 20351094 | T | C | PSPC1 |  | ENST00000427943 | 0 | None | intron_variant | LOW |  | None |  | None | 0.177777778 |
| chr13 | 21967700 | 21967701 | A | G | ZDHHC20 |  | ENST00000400590 | 0 | None | intron_variant | LOW |  | None |  | None | 0.162790698 |
| chr13 | 22233175 | 22233176 | G | C | None |  |  | 0 | None | intergenic_variant | LOW |  | None |  | None | 0.25 |
| chr13 | 22768518 | 22768519 | C | A | None |  |  | 0 | None | intergenic_variant | LOW |  | None |  | None | 0.134615385 |
| chr13 | 22783844 | 22783845 | T | A | None |  |  | 0 | None | intergenic_variant | LOW |  | None |  | None | 0.263157895 |
| chr13 | 22859833 | 22859834 | C | A | None |  |  | 0 | None | intergenic_variant | LOW |  | None |  | None | 0.179487179 |
| chr13 | 23086110 | 23086111 | C | T | None |  |  | 0 | None | intergenic_variant | LOW |  | None |  | None | 0.120689655 |
| chr13 | 23438957 | 23438958 | G | A | None |  |  | 0 | None | intergenic_variant | LOW |  | None |  | None | 0.290909091 |
| chr13 | 23465640 | 23465641 | C | A | LINC00621 |  | ENST00000577004 | 0 | None | downstream_gene_variant | LOW |  | None |  | None | 0.245614035 |
| chr13 | 23851387 | 23851388 | C | T | SGCG |  | ENST00000545013 | 0 | None | intron_variant | LOW |  | None |  | None | 0.309090909 |
| chr13 | 24963893 | 24963894 | C | T | None |  |  | 0 | None | intergenic_variant | LOW |  | None |  | None | 0.146666667 |
| chr13 | 25459668 | 25459669 | C | T | CENPJ |  | ENST00000381884 | 0 | None | intron_variant | LOW |  | None |  | None | 0.303571429 |
| chr13 | 25622154 | 25622155 | T | C | None |  |  | 0 | None | intergenic_variant | LOW |  | None |  | None | 0.173076923 |
| chr13 | 26494675 | 26494676 | G | A | ATP8A2 |  | ENST00000381655 | 1 | None | intron_variant | LOW |  | None |  | None | 0.197183099 |
| chr13 | 28640011 | 28640012 | A | G | FLT3 |  | ENST00000537084 | 1 | None | intron_variant | LOW |  | None |  | None | 0.315789474 |
| chr13 | 28985515 | 28985516 | C | A | FLT1 |  | ENST00000539099 | 0 | None | intron_variant | LOW |  | None |  | None | 0.3 |
| chr13 | 29595795 | 29595796 | C | G | MTUS2 |  | ENST00000431530 | 0 | None | upstream_gene_variant | LOW |  | None |  | None | 0.173913043 |
| chr13 | 29879666 | 29879667 | A | T | MTUS2 |  | ENST00000431530 | 0 | None | intron_variant | LOW |  | None |  | None | 0.133333333 |
| chr13 | 29933852 | 29933853 | C | T | MTUS2 |  | ENST00000431530 | 0 | None | intron_variant | LOW |  | None |  | None | 0.26 |
| chr13 | 29991313 | 29991314 | G | A | MTUS2 |  | ENST00000431530 | 0 | None | intron_variant | LOW |  | None |  | None | 0.274509804 |
| chr13 | 30458716 | 30458717 | T | C | LINC00297 |  | ENST00000453470 | 0 | None | intron_variant | LOW |  | None |  | None | 0.25 |
| chr13 | 32388677 | 32388678 | C | T | None |  |  | 0 | None | intergenic_variant | LOW |  | None |  | None | 0.238095238 |
| chr13 | 32794105 | 32794106 | T | G | FRY |  | ENST00000380250 | 0 | None | intron_variant | LOW |  | None |  | None | 0.127659574 |
| chr13 | 34211554 | 34211555 | G | A | RP11-141M1.3 |  | ENST00000454681 | 0 | None | intron_variant | LOW |  | None |  | None | 0.151515152 |
| chr13 | 34221100 | 34221101 | T | A | RP11-141M1.3 |  | ENST00000454681 | 0 | None | intron_variant | LOW |  | None |  | None | 0.277777778 |
| chr13 | 34478159 | 34478160 | G | A | RFC3 |  | ENST00000434425 | 0 | None | intron_variant | LOW |  | None |  | None | 0.261538462 |
| chr13 | 36721540 | 36721541 | C | G | None |  |  | 0 | None | intergenic_variant | LOW |  | None |  | None | 0.170212766 |
| chr13 | 36912933 | 36912934 | G | T | SPG20 |  | ENST00000438666 | 0 | None | intron_variant | LOW |  | None |  | None | 0.116666667 |
| chr13 | 36918012 | 36918013 | C | T | SPG20 |  | ENST00000451493 | 0 | None | intron_variant | LOW |  | None |  | None | 0.155172414 |
| chr13 | 37095889 | 37095890 | C | T | None |  |  | 0 | None | intergenic_variant | LOW |  | None |  | None | 0.241935484 |
| chr13 | 37603491 | 37603492 | C | T | SUPT20H |  | ENST00000542180 | 0 | None | intron_variant | LOW |  | None |  | None | 0.320754717 |
| chr13 | 38075019 | 38075020 | C | T | None |  |  | 0 | None | intergenic_variant | LOW |  | None |  | None | 0.175438596 |
| chr13 | 38489873 | 38489874 | T | G | None |  |  | 0 | None | intergenic_variant | LOW |  | None |  | None | 0.290909091 |
| chr13 | 38593296 | 38593297 | G | A | None |  |  | 0 | None | intergenic_variant | LOW |  | None |  | None | 0.18 |
| chr13 | 38875448 | 38875449 | C | T | None |  |  | 0 | None | intergenic_variant | LOW |  | None |  | None | 0.28125 |
| chr13 | 39202662 | 39202663 | T | C | None |  |  | 0 | None | intergenic_variant | LOW |  | None |  | None | 0.214285714 |
| chr13 | 39301211 | 39301212 | G | A | FREM2 |  | ENST00000280481 | 1 | None | intron_variant | LOW |  | None |  | None | 0.333333333 |
| chr13 | 40993786 | 40993787 | G | A | None |  |  | 0 | None | intergenic_variant | LOW |  | None |  | None | 0.191489362 |
| chr13 | 41062199 | 41062200 | G | C | None |  |  | 0 | None | intergenic_variant | LOW |  | None |  | None | 0.204081633 |
| chr13 | 41280302 | 41280303 | A | T | None |  |  | 0 | None | intergenic_variant | LOW |  | None |  | None | 0.256410256 |
| chr13 | 41395495 | 41395496 | G | A | TPTE2P5 |  | ENST00000432905 | 0 | None | downstream_gene_variant | LOW |  | None |  | None | 0.384615385 |
| chr13 | 42708156 | 42708157 | A | T | DGKH |  | ENST00000379274 | 0 | None | intron_variant | LOW |  | None |  | None | 0.254901961 |
| chr13 | 43285234 | 43285235 | G | A | None |  |  | 0 | None | intergenic_variant | LOW |  | None |  | None | 0.105263158 |
| chr13 | 45940613 | 45940614 | C | T | TPT1-AS1 |  | ENST00000524062 | 0 | None | intron_variant | LOW |  | None |  | None | 0.113207547 |
| chr13 | 46269799 | 46269800 | G | C | None |  |  | 0 | None | intergenic_variant | LOW |  | None |  | None | 0.196428571 |
| chr13 | 47784106 | 47784107 | G | A | None |  |  | 1 | None | intergenic_variant | LOW |  | None |  | None | 0.266666667 |
| chr13 | 48039624 | 48039625 | T | A | None |  |  | 0 | None | intergenic_variant | LOW |  | None |  | None | 0.166666667 |
| chr13 | 49511448 | 49511449 | G | A | None |  |  | 0 | None | intergenic_variant | LOW |  | None |  | None | 0.152173913 |
| chr13 | 49911478 | 49911479 | A | G | CAB39L |  | ENST00000355854 | 0 | None | intron_variant | LOW |  | None |  | None | 0.306122449 |
| chr13 | 50113313 | 50113314 | T | A | RCBTB1 |  | ENST00000258646 | 1 | None | intron_variant | LOW |  | None |  | None | 0.204081633 |
| chr13 | 50699591 | 50699592 | C | T | DLEU1 |  | ENST00000463474 | 0 | None | intron_variant | LOW |  | None |  | None | 0.132075472 |
| chr13 | 52860728 | 52860729 | T | G | RP11-248G5.8 |  | ENST00000451298 | 0 | None | intron_variant | LOW |  | None |  | None | 0.114035088 |
| chr13 | 53259397 | 53259398 | T | C | SUGT1 |  | ENST00000535397 | 0 | None | intron_variant | LOW |  | None |  | None | 0.162162162 |
| chr13 | 56306439 | 56306440 | G | A | None |  |  | 1 | None | intergenic_variant | LOW |  | None |  | None | 0.255813953 |
| chr13 | 56520675 | 56520676 | A | T | None |  |  | 0 | None | intergenic_variant | LOW |  | None |  | None | 0.317073171 |
| chr13 | 56680913 | 56680914 | A | T | None |  |  | 0 | None | intergenic_variant | LOW |  | None |  | None | 0.153846154 |
| chr13 | 57148364 | 57148365 | C | T | None |  |  | 0 | None | intergenic_variant | LOW |  | None |  | None | 0.266666667 |
| chr13 | 57533018 | 57533019 | C | A | None |  |  | 0 | None | intergenic_variant | LOW |  | None |  | None | 0.30952381 |
| chr13 | 58066700 | 58066701 | C | G | None |  |  | 1 | None | intergenic_variant | LOW |  | None |  | None | 0.139534884 |
| chr13 | 58066714 | 58066715 | G | C | None |  |  | 1 | None | intergenic_variant | LOW |  | None |  | None | 0.130434783 |
| chr13 | 58066775 | 58066776 | A | C | None |  |  | 1 | None | intergenic_variant | LOW |  | None |  | None | 0.166666667 |
| chr13 | 59124567 | 59124568 | A | G | None |  |  | 0 | None | intergenic_variant | LOW |  | None |  | None | 0.205882353 |
| chr13 | 59142078 | 59142079 | C | A | None |  |  | 0 | None | intergenic_variant | LOW |  | None |  | None | 0.256410256 |
| chr13 | 59290176 | 59290177 | G | T | None |  |  | 0 | None | intergenic_variant | LOW |  | None |  | None | 0.324324324 |
| chr13 | 59379036 | 59379037 | C | T | None |  |  | 0 | None | intergenic_variant | LOW |  | None |  | None | 0.352941176 |
| chr13 | 60294879 | 60294880 | T | A | DIAPH3 |  | ENST00000400330 | 0 | None | intron_variant | LOW |  | None |  | None | 0.296875 |
| chr13 | 60696220 | 60696221 | C | G | DIAPH3 |  | ENST00000400324 | 0 | None | intron_variant | LOW |  | None |  | None | 0.34 |
| chr13 | 62859400 | 62859401 | G | T | None |  |  | 0 | None | intergenic_variant | LOW |  | None |  | None | 0.125 |
| chr13 | 64202098 | 64202099 | C | T | None |  |  | 0 | None | intergenic_variant | LOW |  | None |  | None | 0.365853659 |
| chr13 | 64811226 | 64811227 | A | G | None |  |  | 0 | None | intergenic_variant | LOW |  | None |  | None | 0.368421053 |
| chr13 | 64926248 | 64926249 | C | A | None |  |  | 0 | None | intergenic_variant | LOW |  | None |  | None | 0.393939394 |
| chr13 | 65948322 | 65948323 | G | A | None |  |  | 0 | None | intergenic_variant | LOW |  | None |  | None | 0.285714286 |
| chr13 | 66210818 | 66210819 | G | A | None |  |  | 0 | None | intergenic_variant | LOW |  | None |  | None | 0.27027027 |
| chr13 | 66444022 | 66444023 | C | T | LINC01052 |  | ENST00000437777 | 0 | None | intron_variant | LOW |  | None |  | None | 0.270833333 |
| chr13 | 66716194 | 66716195 | G | A | None |  |  | 0 | None | intergenic_variant | LOW |  | None |  | None | 0.101449275 |
| chr13 | 68064324 | 68064325 | C | A | None |  |  | 0 | None | intergenic_variant | LOW |  | None |  | None | 0.142857143 |
| chr13 | 68206102 | 68206103 | G | A | None |  |  | 0 | None | intergenic_variant | LOW |  | None |  | None | 0.304347826 |
| chr13 | 68321349 | 68321350 | A | C | None |  |  | 0 | None | intergenic_variant | LOW |  | None |  | None | 0.28125 |
| chr13 | 69047697 | 69047698 | T | C | None |  |  | 0 | None | intergenic_variant | LOW |  | None |  | None | 0.317073171 |
| chr13 | 69339626 | 69339627 | C | A | None |  |  | 1 | None | intergenic_variant | LOW |  | None |  | None | 0.27027027 |
| chr13 | 70042159 | 70042160 | T | C | None |  |  | 0 | None | intergenic_variant | LOW |  | None |  | None | 0.289473684 |
| chr13 | 70090542 | 70090543 | C | T | None |  |  | 0 | None | intergenic_variant | LOW |  | None |  | None | 0.333333333 |
| chr13 | 70469033 | 70469034 | C | A | KLHL1 |  | ENST00000377844 | 0 | None | intron_variant | LOW |  | None |  | None | 0.314285714 |
| chr13 | 71036132 | 71036133 | C | A | RNU6-54P |  | ENST00000365563 | 0 | None | downstream_gene_variant | LOW |  | None |  | None | 0.142857143 |
| chr13 | 71549652 | 71549653 | C | G | None |  |  | 0 | None | intergenic_variant | LOW |  | None |  | None | 0.178571429 |
| chr13 | 72407200 | 72407201 | T | C | DACH1 |  | ENST00000305425 | 0 | None | intron_variant | LOW |  | None |  | None | 0.205128205 |
| chr13 | 72940774 | 72940775 | T | A | None |  |  | 0 | None | intergenic_variant | LOW |  | None |  | None | 0.238095238 |
| chr13 | 76441615 | 76441616 | C | A | C13orf45 |  | ENST00000318245 | 0 | None | upstream_gene_variant | LOW |  | None |  | None | 0.272727273 |
| chr13 | 77851881 | 77851882 | G | A | MYCBP2 |  | ENST00000407578 | 0 | None | intron_variant | LOW |  | None |  | None | 0.14893617 |
| chr13 | 78750846 | 78750847 | C | T | RNF219-AS1 |  | ENST00000606124 | 1 | None | intron_variant | LOW |  | None |  | None | 0.261904762 |
| chr13 | 79261036 | 79261037 | C | A | None |  |  | 0 | None | intergenic_variant | LOW |  | None |  | None | 0.233333333 |
| chr13 | 79520016 | 79520017 | T | G | None |  |  | 0 | None | intergenic_variant | LOW |  | None |  | None | 0.2 |
| chr13 | 80162299 | 80162300 | G | T | None |  |  | 0 | None | intergenic_variant | LOW |  | None |  | None | 0.11627907 |
| chr13 | 80952980 | 80952981 | A | T | None |  |  | 0 | None | intergenic_variant | LOW |  | None |  | None | 0.222222222 |
| chr13 | 81445213 | 81445214 | G | A | None |  |  | 0 | None | intergenic_variant | LOW |  | None |  | None | 0.242424242 |
| chr13 | 81970994 | 81970995 | A | C | None |  |  | 0 | None | intergenic_variant | LOW |  | None |  | None | 0.306122449 |
| chr13 | 82701272 | 82701273 | C | T | None |  |  | 0 | None | intergenic_variant | LOW |  | None |  | None | 0.2 |
| chr13 | 82770481 | 82770482 | C | A | None |  |  | 0 | None | intergenic_variant | LOW |  | None |  | None | 0.291666667 |
| chr13 | 82867021 | 82867022 | C | T | None |  |  | 0 | None | intergenic_variant | LOW |  | None |  | None | 0.14893617 |
| chr13 | 82867041 | 82867042 | C | T | None |  |  | 0 | None | intergenic_variant | LOW |  | None |  | None | 0.12 |
| chr13 | 83780661 | 83780662 | C | A | None |  |  | 0 | None | intergenic_variant | LOW |  | None |  | None | 0.166666667 |
| chr13 | 83970564 | 83970565 | G | A | None |  |  | 0 | None | intergenic_variant | LOW |  | None |  | None | 0.245283019 |
| chr13 | 86054499 | 86054500 | T | C | LINC00351 |  | ENST00000424926 | 0 | None | intron_variant | LOW |  | None |  | None | 0.153846154 |
| chr13 | 86189515 | 86189516 | C | G | None |  |  | 0 | None | intergenic_variant | LOW |  | None |  | None | 0.333333333 |
| chr13 | 86623657 | 86623658 | C | A | None |  |  | 0 | None | intergenic_variant | LOW |  | None |  | None | 0.196078431 |
| chr13 | 87119064 | 87119065 | A | T | None |  |  | 0 | None | intergenic_variant | LOW |  | None |  | None | 0.446808511 |
| chr13 | 87340160 | 87340161 | T | C | None |  |  | 0 | None | intergenic_variant | LOW |  | None |  | None | 0.205128205 |
| chr13 | 87705309 | 87705310 | G | T | None |  |  | 0 | None | intergenic_variant | LOW |  | None |  | None | 0.181818182 |
| chr13 | 87906865 | 87906866 | C | T | None |  |  | 0 | None | intergenic_variant | LOW |  | None |  | None | 0.189189189 |
| chr13 | 87929724 | 87929725 | C | T | None |  |  | 0 | None | intergenic_variant | LOW |  | None |  | None | 0.2 |
| chr13 | 89169237 | 89169238 | T | C | None |  |  | 0 | None | intergenic_variant | LOW |  | None |  | None | 0.139534884 |
| chr13 | 89169344 | 89169345 | T | C | None |  |  | 1 | None | intergenic_variant | LOW |  | None |  | None | 0.208333333 |
| chr13 | 89169352 | 89169353 | G | C | None |  |  | 1 | None | intergenic_variant | LOW |  | None |  | None | 0.204081633 |
| chr13 | 89169372 | 89169373 | C | T | None |  |  | 0 | None | intergenic_variant | LOW |  | None |  | None | 0.24 |
| chr13 | 89169377 | 89169378 | T | G | None |  |  | 0 | None | intergenic_variant | LOW |  | None |  | None | 0.254901961 |
| chr13 | 89224045 | 89224046 | A | T | None |  |  | 0 | None | intergenic_variant | LOW |  | None |  | None | 0.285714286 |
| chr13 | 89338812 | 89338813 | G | T | None |  |  | 0 | None | intergenic_variant | LOW |  | None |  | None | 0.177777778 |
| chr13 | 89475954 | 89475955 | G | A | None |  |  | 0 | None | intergenic_variant | LOW |  | None |  | None | 0.447368421 |
| chr13 | 91532965 | 91532966 | G | T | None |  |  | 0 | None | intergenic_variant | LOW |  | None |  | None | 0.155172414 |
| chr13 | 91616071 | 91616072 | T | G | None |  |  | 0 | None | intergenic_variant | LOW |  | None |  | None | 0.20754717 |
| chr13 | 91616167 | 91616168 | G | C | None |  |  | 0 | None | intergenic_variant | LOW |  | None |  | None | 0.269230769 |
| chr13 | 91705050 | 91705051 | G | C | None |  |  | 0 | None | intergenic_variant | LOW |  | None |  | None | 0.109090909 |
| chr13 | 93423892 | 93423893 | G | A | GPC5 |  | ENST00000377067 | 0 | None | intron_variant | LOW |  | None |  | None | 0.292307692 |
| chr13 | 93478487 | 93478488 | G | A | GPC5 |  | ENST00000377067 | 0 | None | intron_variant | LOW |  | None |  | None | 0.109090909 |
| chr13 | 93478510 | 93478511 | T | G | GPC5 |  | ENST00000377067 | 1 | None | intron_variant | LOW |  | None |  | None | 0.103448276 |
| chr13 | 93750370 | 93750371 | C | T | None |  |  | 0 | None | intergenic_variant | LOW |  | None |  | None | 0.203703704 |
| chr13 | 94407202 | 94407203 | C | T | GPC6 |  | ENST00000377047 | 0 | None | intron_variant | LOW |  | None |  | None | 0.230769231 |
| chr13 | 95500271 | 95500272 | A | T | None |  |  | 0 | None | intergenic_variant | LOW |  | None |  | None | 0.163636364 |
| chr13 | 95500281 | 95500282 | G | A | None |  |  | 0 | None | intergenic_variant | LOW |  | None |  | None | 0.138461538 |
| chr13 | 97542517 | 97542518 | G | A | HSP90AB6P |  | ENST00000442053 | 0 | None | upstream_gene_variant | LOW |  | None |  | None | 0.245901639 |
| chr13 | 98328939 | 98328940 | C | A | RP11-120E13.1 |  | ENST00000437334 | 0 | None | intron_variant | LOW |  | None |  | None | 0.174603175 |
| chr13 | 98392340 | 98392341 | C | T | None |  |  | 0 | None | intergenic_variant | LOW |  | None |  | None | 0.213114754 |
| chr13 | 100436339 | 100436340 | A | G | CLYBL |  | ENST00000339105 | 0 | None | intron_variant | LOW |  | None |  | None | 0.257142857 |
| chr13 | 101701177 | 101701178 | T | C | NALCN |  | ENST00000251127 | 0 | None | downstream_gene_variant | LOW |  | None |  | None | 0.272727273 |
| chr13 | 102106262 | 102106263 | C | A | ITGBL1 | S/Y | ENST00000376180 | 0 | None | missense_variant | MED | possibly_damaging | 0.881 | deleterious | 0.01 | 0.134615385 |
| chr13 | 102908960 | 102908961 | A | G | FGF14 |  | ENST00000376131 | 0 | None | intron_variant | LOW |  | None |  | None | 0.145833333 |
| chr13 | 103113106 | 103113107 | G | A | None |  |  | 0 | None | intergenic_variant | LOW |  | None |  | None | 0.191489362 |
| chr13 | 103607064 | 103607065 | G | A | None |  |  | 0 | None | intergenic_variant | LOW |  | None |  | None | 0.228070175 |
| chr13 | 104100007 | 104100008 | G | A | ATP6V1G1P7 |  | ENST00000415853 | 0 | None | upstream_gene_variant | LOW |  | None |  | None | 0.173076923 |
| chr13 | 105322005 | 105322006 | C | G | None |  |  | 0 | None | intergenic_variant | LOW |  | None |  | None | 0.163934426 |
| chr13 | 105541358 | 105541359 | T | A | None |  |  | 0 | None | intergenic_variant | LOW |  | None |  | None | 0.203703704 |
| chr13 | 105558099 | 105558100 | A | G | None |  |  | 0 | None | intergenic_variant | LOW |  | None |  | None | 0.145454545 |
| chr13 | 105786288 | 105786289 | A | C | None |  |  | 0 | None | intergenic_variant | LOW |  | None |  | None | 0.170212766 |
| chr13 | 106154184 | 106154185 | G | C | DAOA-AS1 |  | ENST00000448407 | 0 | None | intron_variant | LOW |  | None |  | None | 0.254545455 |
| chr13 | 107498438 | 107498439 | G | T | None |  |  | 0 | None | intergenic_variant | LOW |  | None |  | None | 0.142857143 |
| chr13 | 107901625 | 107901626 | T | G | FAM155A |  | ENST00000375915 | 0 | None | intron_variant | LOW |  | None |  | None | 0.254545455 |
| chr13 | 108149612 | 108149613 | C | A | FAM155A |  | ENST00000375915 | 0 | None | intron_variant | LOW |  | None |  | None | 0.222222222 |
| chr13 | 108236565 | 108236566 | A | T | FAM155A |  | ENST00000375915 | 0 | None | intron_variant | LOW |  | None |  | None | 0.139534884 |
| chr13 | 108590775 | 108590776 | T | C | None |  |  | 0 | None | intergenic_variant | LOW |  | None |  | None | 0.166666667 |
| chr13 | 108854738 | 108854739 | G | C | None |  |  | 0 | None | intergenic_variant | LOW |  | None |  | None | 0.333333333 |
| chr13 | 109404832 | 109404833 | C | T | MYO16 |  | ENST00000356711 | 0 | None | intron_variant | LOW |  | None |  | None | 0.117647059 |
| chr13 | 109442577 | 109442578 | C | T | MYO16 |  | ENST00000356711 | 0 | None | intron_variant | LOW |  | None |  | None | 0.234375 |
| chr13 | 110165909 | 110165910 | A | G | None |  |  | 0 | None | intergenic_variant | LOW |  | None |  | None | 0.126984127 |
| chr13 | 111021330 | 111021331 | G | A | COL4A2 |  | ENST00000360467 | 0 | None | intron_variant | LOW |  | None |  | None | 0.137254902 |
| chr13 | 111266174 | 111266175 | A | C | CARKD |  | ENST00000458711 | 0 | None | upstream_gene_variant | LOW |  | None |  | None | 0.285714286 |
| chr13 | 111473788 | 111473789 | C | T | None |  |  | 1 | None | intergenic_variant | LOW |  | None |  | None | 0.175 |
| chr13 | 111496571 | 111496572 | C | T | None |  |  | 0 | None | intergenic_variant | LOW |  | None |  | None | 0.25 |
| chr13 | 111720714 | 111720715 | C | G | None |  |  | 0 | None | intergenic_variant | LOW |  | None |  | None | 0.206349206 |
| chr13 | 111825096 | 111825097 | A | G | ARHGEF7 |  | ENST00000375739 | 0 | None | intron_variant | LOW |  | None |  | None | 0.220588235 |
| chr13 | 112615127 | 112615128 | G | A | None |  |  | 1 | None | intergenic_variant | LOW |  | None |  | None | 0.163636364 |
| chr13 | 112934102 | 112934103 | G | A | None |  |  | 0 | None | intergenic_variant | LOW |  | None |  | None | 0.090909091 |
| chr13 | 112934177 | 112934178 | T | A | None |  |  | 1 | None | intergenic_variant | LOW |  | None |  | None | 0.154471545 |
| chr13 | 112965475 | 112965476 | T | A | LINC01043 |  | ENST00000565936 | 1 | None | upstream_gene_variant | LOW |  | None |  | None | 0.130081301 |
| chr13 | 113215941 | 113215942 | T | C | TUBGCP3 |  | ENST00000375669 | 0 | None | intron_variant | LOW |  | None |  | None | 0.25862069 |
| chr13 | 113736370 | 113736371 | A | G | MCF2L |  | ENST00000375597 | 0 | None | intron_variant | LOW |  | None |  | None | 0.192307692 |
| chr13 | 113739966 | 113739967 | A | C | MCF2L |  | ENST00000434480 | 0 | None | intron_variant | LOW |  | None |  | None | 0.239130435 |
| chr13 | 113870447 | 113870448 | A | G | CUL4A |  | ENST00000326335 | 0 | None | intron_variant | LOW |  | None |  | None | 0.176470588 |
| chr13 | 115097941 | 115097942 | G | A | LINC01054 |  | ENST00000446989 | 0 | None | intron_variant | LOW |  | None |  | None | 0.224137931 |
| chr14 | 19500540 | 19500541 | C | T | MED15P1 |  | ENST00000552968 | 1 | None | intron_variant | LOW |  | None |  | None | 0.19 |
| chr14 | 19607881 | 19607882 | A | G | CTD-2314B22.1 |  | ENST00000546734 | 1 | None | upstream_gene_variant | LOW |  | None |  | None | 0.158730159 |
| chr14 | 19780969 | 19780970 | A | T | None |  |  | 1 | None | intergenic_variant | LOW |  | None |  | None | 0.246376812 |
| chr14 | 19819342 | 19819343 | C | T | RP11-146E13.4 |  | ENST00000548109 | 1 | None | intron_variant | LOW |  | None |  | None | 0.069148936 |
| chr14 | 19821628 | 19821629 | A | G | RP11-146E13.4 |  | ENST00000548109 | 0 | None | intron_variant | LOW |  | None |  | None | 0.086956522 |
| chr14 | 19873183 | 19873184 | A | G | CTD-2314B22.3 |  | ENST00000551334 | 1 | None | intron_variant | LOW |  | None |  | None | 0.089108911 |
| chr14 | 19942977 | 19942978 | G | A | CTD-2311B13.7 |  | ENST00000547399 | 0 | None | intron_variant | LOW |  | None |  | None | 0.1875 |
| chr14 | 19970853 | 19970854 | C | G | CTD-2311B13.7 |  | ENST00000547399 | 1 | None | non_coding_exon_variant | LOW |  | None |  | None | 0.181818182 |
| chr14 | 19979365 | 19979366 | C | G | POTEM |  | ENST00000551509 | 1 | None | downstream_gene_variant | LOW |  | None |  | None | 0.142857143 |
| chr14 | 20090261 | 20090262 | G | A | RP11-597A11.1 |  | ENST00000548217 | 0 | None | intron_variant | LOW |  | None |  | None | 0.075949367 |
| chr14 | 20706180 | 20706181 | T | G | OR11H4 |  | ENST00000315409 | 0 | None | upstream_gene_variant | LOW |  | None |  | None | NA |
| chr14 | 20706189 | 20706190 | T | C | OR11H4 |  | ENST00000315409 | 0 | None | upstream_gene_variant | LOW |  | None |  | None | NA |
| chr14 | 21586455 | 21586456 | C | T | RNU6-252P |  | ENST00000363232 | 0 | None | upstream_gene_variant | LOW |  | None |  | None | 0.206349206 |
| chr14 | 22237838 | 22237839 | G | A | TRAV6 |  | ENST00000390428 | 0 | None | downstream_gene_variant | LOW |  | None |  | None | 0.215686275 |
| chr14 | 22994722 | 22994723 | G | T | TRAJ14 |  | ENST00000390523 | 0 | None | upstream_gene_variant | LOW |  | None |  | None | 0.223880597 |
| chr14 | 23361757 | 23361758 | G | A | REM2 |  | ENST00000267396 | 0 | None | downstream_gene_variant | LOW |  | None |  | None | 0.118644068 |
| chr14 | 24682419 | 24682420 | G | A | CHMP4A |  | ENST00000530996 | 0 | None | intron_variant | LOW |  | None |  | None | 0.253731343 |
| chr14 | 26031951 | 26031952 | G | C | None |  |  | 0 | None | intergenic_variant | LOW |  | None |  | None | 0.15 |
| chr14 | 26319651 | 26319652 | G | T | RP11-314P15.2 |  | ENST00000546412 | 0 | None | intron_variant | LOW |  | None |  | None | 0.25 |
| chr14 | 26967065 | 26967066 | C | A | NOVA1 |  | ENST00000549571 | 0 | None | intron_variant | LOW |  | None |  | None | 0.107692308 |
| chr14 | 26967144 | 26967145 | T | A | NOVA1 |  | ENST00000549571 | 0 | None | intron_variant | LOW |  | None |  | None | 0.184615385 |
| chr14 | 28017259 | 28017260 | A | G | CTD-3006G17.2 |  | ENST00000554904 | 0 | None | intron_variant | LOW |  | None |  | None | 0.170212766 |
| chr14 | 28037719 | 28037720 | C | A | CTD-3006G17.2 |  | ENST00000554904 | 0 | None | intron_variant | LOW |  | None |  | None | 0.28 |
| chr14 | 28914183 | 28914184 | G | A | None |  |  | 0 | None | intergenic_variant | LOW |  | None |  | None | 0.14893617 |
| chr14 | 28997760 | 28997761 | C | A | None |  |  | 0 | None | intergenic_variant | LOW |  | None |  | None | 0.196078431 |
| chr14 | 29000375 | 29000376 | C | A | None |  |  | 0 | None | intergenic_variant | LOW |  | None |  | None | 0.212765957 |
| chr14 | 29614187 | 29614188 | G | T | None |  |  | 0 | None | intergenic_variant | LOW |  | None |  | None | 0.145833333 |
| chr14 | 29727414 | 29727415 | T | C | None |  |  | 0 | None | intergenic_variant | LOW |  | None |  | None | 0.192982456 |
| chr14 | 31162872 | 31162873 | C | T | SCFD1 |  | ENST00000421551 | 0 | None | intron_variant | LOW |  | None |  | None | 0.25 |
| chr14 | 31720121 | 31720122 | T | G | RP11-596D21.1 |  | ENST00000551799 | 1 | None | intron_variant | LOW |  | None |  | None | 0.195121951 |
| chr14 | 32061303 | 32061304 | G | C | NUBPL |  | ENST00000550649 | 0 | None | intron_variant | LOW |  | None |  | None | 0.155555556 |
| chr14 | 32061304 | 32061305 | C | T | NUBPL |  | ENST00000550649 | 0 | None | intron_variant | LOW |  | None |  | None | 0.155555556 |
| chr14 | 33224419 | 33224420 | A | T | AKAP6 |  | ENST00000280979 | 0 | None | intron_variant | LOW |  | None |  | None | 0.234042553 |
| chr14 | 33426594 | 33426595 | G | T | NPAS3 |  | ENST00000357798 | 0 | None | intron_variant | LOW |  | None |  | None | 0.140625 |
| chr14 | 34960959 | 34960960 | C | T | None |  |  | 0 | None | intergenic_variant | LOW |  | None |  | None | 0.193548387 |
| chr14 | 35072689 | 35072690 | T | C | SNX6 |  | ENST00000396534 | 1 | None | intron_variant | LOW |  | None |  | None | 0.153846154 |
| chr14 | 35172815 | 35172816 | A | G | None |  |  | 0 | None | intergenic_variant | LOW |  | None |  | None | 0.103448276 |
| chr14 | 35388003 | 35388004 | G | T | RP11-85K15.2 |  | ENST00000555015 | 0 | None | downstream_gene_variant | LOW |  | None |  | None | 0.16 |
| chr14 | 38059332 | 38059333 | T | A | FOXA1 |  | ENST00000250448 | 0 | None | 3_prime_UTR_variant | LOW |  | None |  | None | 0.163636364 |
| chr14 | 38211928 | 38211929 | C | G | TTC6 |  | ENST00000553443 | 0 | None | intron_variant | LOW |  | None |  | None | 0.223880597 |
| chr14 | 38787655 | 38787656 | T | C | None |  |  | 0 | None | intergenic_variant | LOW |  | None |  | None | 0.227272727 |
| chr14 | 39184354 | 39184355 | A | C | None |  |  | 1 | None | intergenic_variant | LOW |  | None |  | None | 0.212121212 |
| chr14 | 39858327 | 39858328 | C | T | CTAGE5 |  | ENST00000341502 | 0 | None | downstream_gene_variant | LOW |  | None |  | None | 0.088235294 |
| chr14 | 40527434 | 40527435 | G | T | None |  |  | 0 | None | intergenic_variant | LOW |  | None |  | None | 0.135135135 |
| chr14 | 41069543 | 41069544 | C | A | None |  |  | 0 | None | intergenic_variant | LOW |  | None |  | None | 0.25862069 |
| chr14 | 41280641 | 41280642 | T | A | None |  |  | 0 | None | intergenic_variant | LOW |  | None |  | None | 0.313953488 |
| chr14 | 41288702 | 41288703 | G | A | None |  |  | 0 | None | intergenic_variant | LOW |  | None |  | None | 0.183673469 |
| chr14 | 41340890 | 41340891 | C | T | None |  |  | 0 | None | intergenic_variant | LOW |  | None |  | None | 0.156862745 |
| chr14 | 41624332 | 41624333 | A | G | None |  |  | 0 | None | intergenic_variant | LOW |  | None |  | None | 0.214285714 |
| chr14 | 41774387 | 41774388 | T | A | None |  |  | 0 | None | intergenic_variant | LOW |  | None |  | None | 0.226415094 |
| chr14 | 41991509 | 41991510 | T | C | None |  |  | 0 | None | intergenic_variant | LOW |  | None |  | None | 0.14893617 |
| chr14 | 42142445 | 42142446 | A | T | LRFN5 |  | ENST00000298119 | 0 | None | intron_variant | LOW |  | None |  | None | 0.176470588 |
| chr14 | 42185501 | 42185502 | G | A | LRFN5 |  | ENST00000298119 | 0 | None | intron_variant | LOW |  | None |  | None | 0.19047619 |
| chr14 | 42827706 | 42827707 | A | T | CTD-2307P3.1 |  | ENST00000557251 | 0 | None | upstream_gene_variant | LOW |  | None |  | None | 0.150943396 |
| chr14 | 43535769 | 43535770 | A | G | None |  |  | 0 | None | intergenic_variant | LOW |  | None |  | None | 0.25 |
| chr14 | 43994060 | 43994061 | A | G | None |  |  | 0 | None | intergenic_variant | LOW |  | None |  | None | 0.196078431 |
| chr14 | 44253807 | 44253808 | G | A | None |  |  | 0 | None | intergenic_variant | LOW |  | None |  | None | 0.255319149 |
| chr14 | 44990110 | 44990111 | A | G | RP11-163M18.1 |  | ENST00000555433 | 0 | None | intron_variant | LOW |  | None |  | None | 0.181818182 |
| chr14 | 45009928 | 45009929 | G | C | RP11-163M18.1 |  | ENST00000555433 | 0 | None | downstream_gene_variant | LOW |  | None |  | None | 0.130434783 |
| chr14 | 45593757 | 45593758 | A | T | FKBP3 |  | ENST00000216330 | 0 | None | intron_variant | LOW |  | None |  | None | 0.192307692 |
| chr14 | 46258764 | 46258765 | C | T | None |  |  | 0 | None | intergenic_variant | LOW |  | None |  | None | 0.157894737 |
| chr14 | 46792029 | 46792030 | G | T | LINC00871 |  | ENST00000556886 | 0 | None | intron_variant | LOW |  | None |  | None | 0.215384615 |
| chr14 | 46978377 | 46978378 | C | A | None |  |  | 0 | None | intergenic_variant | LOW |  | None |  | None | 0.191489362 |
| chr14 | 46978423 | 46978424 | C | A | None |  |  | 0 | None | intergenic_variant | LOW |  | None |  | None | 0.137254902 |
| chr14 | 47328382 | 47328383 | T | A | MDGA2 |  | ENST00000426342 | 0 | None | intron_variant | LOW |  | None |  | None | 0.259259259 |
| chr14 | 47360878 | 47360879 | G | A | MDGA2 |  | ENST00000357362 | 0 | None | intron_variant | LOW |  | None |  | None | 0.232142857 |
| chr14 | 47419641 | 47419642 | A | T | MDGA2 |  | ENST00000399232 | 0 | None | intron_variant | LOW |  | None |  | None | 0.279069767 |
| chr14 | 47652788 | 47652789 | T | A | MDGA2 |  | ENST00000399232 | 0 | None | intron_variant | LOW |  | None |  | None | 0.28 |
| chr14 | 47684754 | 47684755 | T | A | MDGA2 |  | ENST00000399232 | 0 | None | intron_variant | LOW |  | None |  | None | 0.150943396 |
| chr14 | 47759446 | 47759447 | G | A | MDGA2 |  | ENST00000439988 | 0 | None | intron_variant | LOW |  | None |  | None | 0.171428571 |
| chr14 | 47834400 | 47834401 | G | A | MDGA2 |  | ENST00000439988 | 0 | None | intron_variant | LOW |  | None |  | None | 0.142857143 |
| chr14 | 47901004 | 47901005 | A | T | MDGA2 |  | ENST00000439988 | 0 | None | intron_variant | LOW |  | None |  | None | 0.209677419 |
| chr14 | 47924448 | 47924449 | C | T | MDGA2 |  | ENST00000399232 | 0 | None | intron_variant | LOW |  | None |  | None | 0.173913043 |
| chr14 | 48151831 | 48151832 | A | G | None |  |  | 0 | None | intergenic_variant | LOW |  | None |  | None | 0.2 |
| chr14 | 48211353 | 48211354 | C | G | None |  |  | 0 | None | intergenic_variant | LOW |  | None |  | None | 0.2 |
| chr14 | 48303199 | 48303200 | A | G | None |  |  | 0 | None | intergenic_variant | LOW |  | None |  | None | 0.204081633 |
| chr14 | 48423405 | 48423406 | C | T | None |  |  | 0 | None | intergenic_variant | LOW |  | None |  | None | 0.214285714 |
| chr14 | 48527621 | 48527622 | T | A | None |  |  | 0 | None | intergenic_variant | LOW |  | None |  | None | 0.220338983 |
| chr14 | 48576049 | 48576050 | A | G | None |  |  | 0 | None | intergenic_variant | LOW |  | None |  | None | 0.25 |
| chr14 | 48671473 | 48671474 | G | C | None |  |  | 0 | None | intergenic_variant | LOW |  | None |  | None | 0.227272727 |
| chr14 | 49113471 | 49113472 | A | C | None |  |  | 0 | None | intergenic_variant | LOW |  | None |  | None | 0.166666667 |
| chr14 | 49113472 | 49113473 | C | A | None |  |  | 0 | None | intergenic_variant | LOW |  | None |  | None | 0.163265306 |
| chr14 | 49267305 | 49267306 | A | G | None |  |  | 0 | None | intergenic_variant | LOW |  | None |  | None | 0.244444444 |
| chr14 | 51423510 | 51423511 | T | C | RP11-218E20.3 |  | ENST00000553648 | 0 | None | non_coding_exon_variant | LOW |  | None |  | None | 0.183098592 |
| chr14 | 51825320 | 51825321 | T | C | RP11-255G12.2 |  | ENST00000555649 | 1 | None | intron_variant | LOW |  | None |  | None | 0.153846154 |
| chr14 | 51954158 | 51954159 | C | T | FRMD6 |  | ENST00000356218 | 0 | None | upstream_gene_variant | LOW |  | None |  | None | 0.163934426 |
| chr14 | 53263562 | 53263563 | C | A | RP11-589M4.3 |  | ENST00000554548 | 0 | None | intron_variant | LOW |  | None |  | None | 0.095238095 |
| chr14 | 54573797 | 54573798 | C | T | None |  |  | 0 | None | intergenic_variant | LOW |  | None |  | None | 0.20754717 |
| chr14 | 54769334 | 54769335 | C | A | None |  |  | 0 | None | intergenic_variant | LOW |  | None |  | None | 0.20754717 |
| chr14 | 55914701 | 55914702 | C | T | TBPL2 |  | ENST00000556755 | 0 | None | intron_variant | LOW |  | None |  | None | 0.206896552 |
| chr14 | 56964522 | 56964523 | C | A | TMEM260 |  | ENST00000556810 | 0 | None | intron_variant | LOW |  | None |  | None | 0.186440678 |
| chr14 | 57136620 | 57136621 | T | A | RP11-1085N6.3 |  | ENST00000554597 | 0 | None | intron_variant | LOW |  | None |  | None | 0.265306122 |
| chr14 | 57471900 | 57471901 | G | A | OTX2-AS1 |  | ENST00000554725 | 0 | None | intron_variant | LOW |  | None |  | None | 0.23880597 |
| chr14 | 57987348 | 57987349 | A | T | None |  |  | 0 | None | intergenic_variant | LOW |  | None |  | None | 0.265306122 |
| chr14 | 58125628 | 58125629 | G | T | SLC35F4 |  | ENST00000556826 | 0 | None | intron_variant | LOW |  | None |  | None | 0.178571429 |
| chr14 | 58639761 | 58639762 | G | A | C14orf37 |  | ENST00000554218 | 1 | None | intron_variant | LOW |  | None |  | None | 0.25 |
| chr14 | 60215084 | 60215085 | T | G | RTN1 |  | ENST00000267484 | 0 | None | intron_variant | LOW |  | None |  | None | 0.237288136 |
| chr14 | 60509496 | 60509497 | C | A | LRRC9 |  | ENST00000445360 | 0 | None | intron_variant | LOW |  | None |  | None | 0.2 |
| chr14 | 61460545 | 61460546 | A | T | SLC38A6 |  | ENST00000533744 | 0 | None | intron_variant | LOW |  | None |  | None | 0.125 |
| chr14 | 62085363 | 62085364 | A | C | RP11-47I22.4 |  | ENST00000556347 | 0 | None | intron_variant | LOW |  | None |  | None | 0.307692308 |
| chr14 | 62454461 | 62454462 | C | T | SYT16 |  | ENST00000446982 | 1 | None | intron_variant | LOW |  | None |  | None | 0.193548387 |
| chr14 | 62863004 | 62863005 | C | T | None |  |  | 0 | None | intergenic_variant | LOW |  | None |  | None | 0.340425532 |
| chr14 | 63131756 | 63131757 | A | G | None |  |  | 0 | None | intergenic_variant | LOW |  | None |  | None | 0.278688525 |
| chr14 | 63442162 | 63442163 | T | G | KCNH5 |  | ENST00000394968 | 0 | None | intron_variant | LOW |  | None |  | None | 0.377777778 |
| chr14 | 68377752 | 68377753 | T | G | RAD51B |  | ENST00000390683 | 0 | None | intron_variant | LOW |  | None |  | None | 0.255813953 |
| chr14 | 68892695 | 68892696 | C | T | RAD51B |  | ENST00000488612 | 1 | None | intron_variant | LOW |  | None |  | None | 0.292682927 |
| chr14 | 69278996 | 69278997 | G | A | None |  |  | 0 | None | intergenic_variant | LOW |  | None |  | None | 0.208955224 |
| chr14 | 72476768 | 72476769 | C | A | RGS6 |  | ENST00000555571 | 0 | None | intron_variant | LOW |  | None |  | None | 0.225806452 |
| chr14 | 72555412 | 72555413 | G | A | RGS6 |  | ENST00000343854 | 0 | None | intron_variant | LOW |  | None |  | None | 0.188679245 |
| chr14 | 72629548 | 72629549 | G | A | RGS6 |  | ENST00000343854 | 0 | None | intron_variant | LOW |  | None |  | None | 0.19047619 |
| chr14 | 73053430 | 73053431 | C | T | RP3-514A23.2 |  | ENST00000555303 | 0 | None | intron_variant | LOW |  | None |  | None | 0.173076923 |
| chr14 | 73065806 | 73065807 | C | A | RP3-514A23.2 |  | ENST00000555303 | 0 | None | upstream_gene_variant | LOW |  | None |  | None | 0.231707317 |
| chr14 | 73780541 | 73780542 | A | C | NUMB |  | ENST00000554521 | 0 | None | intron_variant | LOW |  | None |  | None | 0.295454545 |
| chr14 | 74981260 | 74981261 | G | A | LTBP2 |  | ENST00000261978 | 0 | None | intron_variant | LOW |  | None |  | None | 0.271186441 |
| chr14 | 75536073 | 75536074 | G | T | ZC2HC1C |  | ENST00000524913 | 0 | None | 5_prime_UTR_variant | LOW |  | None |  | None | 0.237288136 |
| chr14 | 76402244 | 76402245 | A | C | TTLL5 |  | ENST00000298832 | 0 | None | intron_variant | LOW |  | None |  | None | 0.154929577 |
| chr14 | 78048886 | 78048887 | G | C | SPTLC2 |  | ENST00000554901 | 0 | None | intron_variant | LOW |  | None |  | None | 0.123076923 |
| chr14 | 79619904 | 79619905 | C | A | NRXN3 |  | ENST00000335750 | 0 | None | intron_variant | LOW |  | None |  | None | 0.254545455 |
| chr14 | 80587932 | 80587933 | C | A | None |  |  | 0 | None | intergenic_variant | LOW |  | None |  | None | 0.209302326 |
| chr14 | 80667464 | 80667465 | T | C | DIO2 |  | ENST00000557010 | 0 | None | 3_prime_UTR_variant | LOW |  | None |  | None | 0.211538462 |
| chr14 | 80890874 | 80890875 | T | C | DIO2-AS1 |  | ENST00000553979 | 0 | None | intron_variant | LOW |  | None |  | None | 0.229166667 |
| chr14 | 81205615 | 81205616 | C | A | CEP128 |  | ENST00000281129 | 0 | None | intron_variant | LOW |  | None |  | None | 0.264150943 |
| chr14 | 82167234 | 82167235 | A | G | None |  |  | 0 | None | intergenic_variant | LOW |  | None |  | None | 0.206349206 |
| chr14 | 82224996 | 82224997 | G | A | RP11-666E17.1 |  | ENST00000554814 | 0 | None | intron_variant | LOW |  | None |  | None | 0.170731707 |
| chr14 | 82825307 | 82825308 | T | C | None |  |  | 0 | None | intergenic_variant | LOW |  | None |  | None | 0.186046512 |
| chr14 | 83535414 | 83535415 | G | T | None |  |  | 0 | None | intergenic_variant | LOW |  | None |  | None | 0.193548387 |
| chr14 | 84084111 | 84084112 | T | C | None |  |  | 0 | None | intergenic_variant | LOW |  | None |  | None | 0.186046512 |
| chr14 | 84387142 | 84387143 | G | T | None |  |  | 0 | None | intergenic_variant | LOW |  | None |  | None | 0.224137931 |
| chr14 | 84510359 | 84510360 | C | T | None |  |  | 0 | None | intergenic_variant | LOW |  | None |  | None | 0.203389831 |
| chr14 | 84979479 | 84979480 | C | T | None |  |  | 0 | None | intergenic_variant | LOW |  | None |  | None | 0.175438596 |
| chr14 | 84982667 | 84982668 | T | G | None |  |  | 0 | None | intergenic_variant | LOW |  | None |  | None | 0.230769231 |
| chr14 | 86148309 | 86148310 | C | G | None |  |  | 0 | None | intergenic_variant | LOW |  | None |  | None | 0.272727273 |
| chr14 | 86367843 | 86367844 | C | A | None |  |  | 0 | None | intergenic_variant | LOW |  | None |  | None | 0.209302326 |
| chr14 | 87083334 | 87083335 | C | A | None |  |  | 0 | None | intergenic_variant | LOW |  | None |  | None | 0.193548387 |
| chr14 | 87182440 | 87182441 | C | A | None |  |  | 0 | None | intergenic_variant | LOW |  | None |  | None | 0.28125 |
| chr14 | 87914326 | 87914327 | A | G | RP11-594C13.1 |  | ENST00000557070 | 0 | None | intron_variant | LOW |  | None |  | None | 0.203389831 |
| chr14 | 88272320 | 88272321 | C | T | RP11-1152H15.1 |  | ENST00000554305 | 0 | None | intron_variant | LOW |  | None |  | None | 0.2 |
| chr14 | 88825466 | 88825467 | T | C | None |  |  | 0 | None | intergenic_variant | LOW |  | None |  | None | 0.134615385 |
| chr14 | 90643969 | 90643970 | A | C | KCNK13 |  | ENST00000282146 | 0 | None | intron_variant | LOW |  | None |  | None | 0.142857143 |
| chr14 | 90724222 | 90724223 | C | T | PSMC1 |  | ENST00000543772 | 0 | None | intron_variant | LOW |  | None |  | None | 0.31147541 |
| chr14 | 91222289 | 91222290 | T | C | TTC7B |  | ENST00000557766 | 0 | None | intron_variant | LOW |  | None |  | None | 0.196428571 |
| chr14 | 91824491 | 91824492 | G | A | CCDC88C |  | ENST00000389857 | 0 | None | intron_variant | LOW |  | None |  | None | 0.271428571 |
| chr14 | 93382758 | 93382759 | G | T | None |  |  | 0 | None | intergenic_variant | LOW |  | None |  | None | 0.166666667 |
| chr14 | 93920944 | 93920945 | T | C | UNC79 |  | ENST00000555664 | 0 | None | intron_variant | LOW |  | None |  | None | 0.203125 |
| chr14 | 94040543 | 94040544 | T | C | UNC79 |  | ENST00000393151 | 0 | None | intron_variant | LOW |  | None |  | None | 0.203389831 |
| chr14 | 94152939 | 94152940 | G | T | UNC79 | C/F | ENST00000393151 | 0 | COSM1516231,COSM1516230 | missense_variant | MED | benign | 0.135 | deleterious | 0 | 0.204545455 |
| chr14 | 94725377 | 94725378 | T | G | PPP4R4 |  | ENST00000304338 | 0 | None | intron_variant | LOW |  | None |  | None | 0.222222222 |
| chr14 | 95839693 | 95839694 | C | G | None |  |  | 0 | None | intergenic_variant | LOW |  | None |  | None | 0.140625 |
| chr14 | 96870507 | 96870508 | A | C | AK7 |  | ENST00000267584 | 1 | None | intron_variant | LOW |  | None |  | None | 0.271186441 |
| chr14 | 97510929 | 97510930 | A | G | None |  |  | 0 | None | intergenic_variant | LOW |  | None |  | None | 0.192982456 |
| chr14 | 97824370 | 97824371 | A | G | None |  |  | 0 | None | intergenic_variant | LOW |  | None |  | None | 0.232142857 |
| chr14 | 97951192 | 97951193 | C | G | CTD-2506J14.1 |  | ENST00000554862 | 0 | None | intron_variant | LOW |  | None |  | None | 0.163265306 |
| chr14 | 98136609 | 98136610 | G | T | RP11-76E12.1 |  | ENST00000554197 | 0 | None | intron_variant | LOW |  | None |  | None | 0.2 |
| chr14 | 98768396 | 98768397 | G | A | None |  |  | 0 | None | intergenic_variant | LOW |  | None |  | None | 0.285714286 |
| chr14 | 98923573 | 98923574 | G | A | None |  |  | 0 | None | intergenic_variant | LOW |  | None |  | None | 0.230769231 |
| chr14 | 99306243 | 99306244 | T | A | AL132796.1 |  | ENST00000421617 | 0 | None | upstream_gene_variant | LOW |  | None |  | None | 0.188405797 |
| chr14 | 100288600 | 100288601 | A | G | EML1 |  | ENST00000556835 | 0 | None | intron_variant | LOW |  | None |  | None | 0.138888889 |
| chr14 | 100656433 | 100656434 | C | A | None |  |  | 0 | None | intergenic_variant | LOW |  | None |  | None | 0.13559322 |
| chr14 | 100669231 | 100669232 | G | A | RP11-638I2.4 |  | ENST00000554537 | 0 | None | downstream_gene_variant | LOW |  | None |  | None | 0.202702703 |
| chr14 | 101064705 | 101064706 | A | T | None |  |  | 0 | None | intergenic_variant | LOW |  | None |  | None | 0.14516129 |
| chr14 | 101154331 | 101154332 | T | C | None |  |  | 0 | None | intergenic_variant | LOW |  | None |  | None | 0.140350877 |
| chr14 | 101455827 | 101455828 | G | A | SNORD114-29 |  | ENST00000364819 | 0 | None | upstream_gene_variant | LOW |  | None |  | None | 0.326923077 |
| chr14 | 101690058 | 101690059 | G | A | None |  |  | 0 | None | intergenic_variant | LOW |  | None |  | None | 0.180555556 |
| chr14 | 101828865 | 101828866 | G | A | None |  |  | 0 | None | intergenic_variant | LOW |  | None |  | None | 0.262295082 |
| chr14 | 103403598 | 103403599 | T | A | CDC42BPB |  | ENST00000361246 | 0 | None | intron_variant | LOW |  | None |  | None | 0.173913043 |
| chr14 | 103652190 | 103652191 | C | T | LINC00605 |  | ENST00000514902 | 0 | None | downstream_gene_variant | LOW |  | None |  | None | 0.157142857 |
| chr14 | 103829918 | 103829919 | C | T | HMGB3P26 |  | ENST00000560051 | 0 | None | downstream_gene_variant | LOW |  | None |  | None | 0.333333333 |
| chr14 | 104425688 | 104425689 | C | T | TDRD9 |  | ENST00000339063 | 0 | None | intron_variant | LOW |  | None |  | None | 0.160714286 |
| chr14 | 104507647 | 104507648 | G | T | TDRD9 |  | ENST00000339063 | 0 | None | intron_variant | LOW |  | None |  | None | 0.225352113 |
| chr14 | 105413965 | 105413966 | C | T | AHNAK2 | E/K | ENST00000333244 | 1 | None | missense_variant | MED | possibly_damaging | 0.51 | tolerated | 0.57 | 0.095890411 |
| chr14 | 106289581 | 106289582 | G | C | None |  |  | 0 | None | intergenic_variant | LOW |  | None |  | None | 0.296875 |
| chr14 | 106718257 | 106718258 | T | G | IGHV3-22 |  | ENST00000520721 | 0 | None | upstream_gene_variant | LOW |  | None |  | None | 0.159574468 |
| chr14 | 107154741 | 107154742 | G | A | IGHVIII-67-4 |  | ENST00000524281 | 0 | None | upstream_gene_variant | LOW |  | None |  | None | 0.191780822 |
| chr15 | 20003174 | 20003175 | C | A | None |  |  | 0 | None | intergenic_variant | LOW |  | None |  | None | 0.14 |
| chr15 | 20024489 | 20024490 | A | G | None |  |  | 0 | None | intergenic_variant | LOW |  | None |  | None | 0.099009901 |
| chr15 | 20077660 | 20077661 | T | C | None |  |  | 0 | None | intergenic_variant | LOW |  | None |  | None | 0.130434783 |
| chr15 | 20113850 | 20113851 | G | T | None |  |  | 0 | None | intergenic_variant | LOW |  | None |  | None | 0.063157895 |
| chr15 | 20117559 | 20117560 | G | A | None |  |  | 1 | None | intergenic_variant | LOW |  | None |  | None | 0.166666667 |
| chr15 | 20119729 | 20119730 | T | C | None |  |  | 0 | None | intergenic_variant | LOW |  | None |  | None | 0.056737589 |
| chr15 | 20119779 | 20119780 | T | A | None |  |  | 0 | None | intergenic_variant | LOW |  | None |  | None | 0.089041096 |
| chr15 | 20126715 | 20126716 | T | A | None |  |  | 0 | None | intergenic_variant | LOW |  | None |  | None | 0.078947368 |
| chr15 | 20127305 | 20127306 | A | C | None |  |  | 0 | None | intergenic_variant | LOW |  | None |  | None | 0.069767442 |
| chr15 | 20132400 | 20132401 | T | C | None |  |  | 0 | None | intergenic_variant | LOW |  | None |  | None | 0.078947368 |
| chr15 | 20138522 | 20138523 | G | A | None |  |  | 0 | None | intergenic_variant | LOW |  | None |  | None | 0.073684211 |
| chr15 | 20161481 | 20161482 | A | G | None |  |  | 1 | None | intergenic_variant | LOW |  | None |  | None | 0.18 |
| chr15 | 20307665 | 20307666 | C | G | None |  |  | 0 | None | intergenic_variant | LOW |  | None |  | None | 0.058139535 |
| chr15 | 20536087 | 20536088 | T | G | None |  |  | 1 | None | intergenic_variant | LOW |  | None |  | None | 0.28 |
| chr15 | 20536087 | 20536088 | T | A | None |  |  | 0 | None | intergenic_variant | LOW |  | None |  | None | 0.36 |
| chr15 | 20640858 | 20640859 | A | C | HERC2P3 |  | ENST00000428453 | 1 | None | intron_variant | LOW |  | None |  | None | 0.144736842 |
| chr15 | 21923836 | 21923837 | A | T | None |  |  | 1 | None | intergenic_variant | LOW |  | None |  | None | 0.25 |
| chr15 | 21923836 | 21923837 | A | G | None |  |  | 1 | None | intergenic_variant | LOW |  | None |  | None | 0.47 |
| chr15 | 22164017 | 22164018 | T | C | None |  |  | 0 | None | intergenic_variant | LOW |  | None |  | None | 0.142857143 |
| chr15 | 22398406 | 22398407 | G | T | RP11-69H14.6 |  | ENST00000559392 | 0 | None | intron_variant | LOW |  | None |  | None | 0.130952381 |
| chr15 | 22436020 | 22436021 | T | C | RP11-2F9.4 |  | ENST00000565129 | 0 | None | intron_variant | LOW |  | None |  | None | 0.158536585 |
| chr15 | 22824343 | 22824344 | G | A | None |  |  | 1 | None | intergenic_variant | LOW |  | None |  | None | 0.139534884 |
| chr15 | 23109002 | 23109003 | C | T | RP11-566K19.6 |  | ENST00000561118 | 1 | None | intron_variant | LOW |  | None |  | None | 0.245283019 |
| chr15 | 23128936 | 23128937 | A | G | RP11-26F2.1 |  | ENST00000560053 | 0 | None | intron_variant | LOW |  | None |  | None | 0.112903226 |
| chr15 | 23775816 | 23775817 | T | C | None |  |  | 0 | None | intergenic_variant | LOW |  | None |  | None | 0.203703704 |
| chr15 | 24353697 | 24353698 | G | A | None |  |  | 0 | None | intergenic_variant | LOW |  | None |  | None | 0.161290323 |
| chr15 | 24393773 | 24393774 | T | C | None |  |  | 0 | None | intergenic_variant | LOW |  | None |  | None | 0.115942029 |
| chr15 | 24393780 | 24393781 | G | A | None |  |  | 0 | None | intergenic_variant | LOW |  | None |  | None | 0.107692308 |
| chr15 | 24412848 | 24412849 | A | G | PWRN2 |  | ENST00000567854 | 1 | None | intron_variant | LOW |  | None |  | None | 0.176470588 |
| chr15 | 24486028 | 24486029 | T | C | RP11-580I1.2 |  | ENST00000561964 | 1 | None | intron_variant | LOW |  | None |  | None | 0.088235294 |
| chr15 | 24600439 | 24600440 | C | T | None |  |  | 0 | None | intergenic_variant | LOW |  | None |  | None | 0.11627907 |
| chr15 | 24705910 | 24705911 | C | A | None |  |  | 0 | None | intergenic_variant | LOW |  | None |  | None | 0.118644068 |
| chr15 | 24720473 | 24720474 | G | C | None |  |  | 0 | None | intergenic_variant | LOW |  | None |  | None | 0.28125 |
| chr15 | 24768586 | 24768587 | C | G | PWRN1 |  | ENST00000565512 | 0 | None | intron_variant | LOW |  | None |  | None | 0.291666667 |
| chr15 | 24984122 | 24984123 | G | T | None |  |  | 0 | None | intergenic_variant | LOW |  | None |  | None | 0.163265306 |
| chr15 | 25028227 | 25028228 | T | A | None |  |  | 0 | None | intergenic_variant | LOW |  | None |  | None | 0.244897959 |
| chr15 | 25158498 | 25158499 | G | T | SNRPN |  | ENST00000400100 | 0 | None | intron_variant | LOW |  | None |  | None | 0.265306122 |
| chr15 | 25295333 | 25295334 | A | G | RP11-701H24.10 |  | ENST00000552781 | 0 | None | intron_variant | LOW |  | None |  | None | 0.203389831 |
| chr15 | 25370717 | 25370718 | G | T | SNHG14 |  | ENST00000553149 | 0 | None | intron_variant | LOW |  | None |  | None | 0.234042553 |
| chr15 | 26360322 | 26360323 | G | A | LINC00929 |  | ENST00000556159 | 0 | None | upstream_gene_variant | LOW |  | None |  | None | 0.192982456 |
| chr15 | 27176410 | 27176411 | A | G | GABRA5 |  | ENST00000555182 | 0 | None | intron_variant | LOW |  | None |  | None | 0.340909091 |
| chr15 | 28700793 | 28700794 | G | A | RP11-483E23.4 |  | ENST00000564639 | 0 | None | intron_variant | LOW |  | None |  | None | 0.138297872 |
| chr15 | 29104322 | 29104323 | T | A | RP11-578F21.12 |  | ENST00000566178 | 0 | None | downstream_gene_variant | LOW |  | None |  | None | 0.208333333 |
| chr15 | 30389498 | 30389499 | T | C | GOLGA8J |  | ENST00000567927 | 1 | None | downstream_gene_variant | LOW |  | None |  | None | 0.183673469 |
| chr15 | 34478930 | 34478931 | T | A | KATNBL1 |  | ENST00000557877 | 0 | None | intron_variant | LOW |  | None |  | None | 0.142857143 |
| chr15 | 35147375 | 35147376 | C | T | AQR |  | ENST00000156471 | 0 | None | downstream_gene_variant | LOW |  | None |  | None | 0.245283019 |
| chr15 | 35670701 | 35670702 | C | A | DPH6 |  | ENST00000558266 | 0 | None | intron_variant | LOW |  | None |  | None | 0.352941176 |
| chr15 | 35866558 | 35866559 | T | A | DPH6-AS1 |  | ENST00000501169 | 0 | None | intron_variant | LOW |  | None |  | None | 0.211538462 |
| chr15 | 36680143 | 36680144 | G | A | None |  |  | 0 | None | intergenic_variant | LOW |  | None |  | None | 0.277777778 |
| chr15 | 43900939 | 43900940 | A | G | STRC |  | ENST00000450892 | 0 | None | intron_variant | LOW |  | None |  | None | 0.255813953 |
| chr15 | 44157285 | 44157286 | A | C | WDR76 |  | ENST00000381246 | 0 | None | intron_variant | LOW |  | None |  | None | 0.235294118 |
| chr15 | 44656650 | 44656651 | A | G | CASC4 |  | ENST00000299957 | 0 | None | intron_variant | LOW |  | None |  | None | 0.220779221 |
| chr15 | 44656688 | 44656689 | G | C | CASC4 |  | ENST00000558847 | 0 | None | intron_variant | LOW |  | None |  | None | 0.109375 |
| chr15 | 45521133 | 45521134 | G | T | None |  |  | 0 | None | intergenic_variant | LOW |  | None |  | None | 0.15625 |
| chr15 | 46376782 | 46376783 | G | T | None |  |  | 0 | None | intergenic_variant | LOW |  | None |  | None | 0.276595745 |
| chr15 | 47361556 | 47361557 | C | T | None |  |  | 0 | None | intergenic_variant | LOW |  | None |  | None | 0.152173913 |
| chr15 | 47367889 | 47367890 | G | T | None |  |  | 1 | None | intergenic_variant | LOW |  | None |  | None | 0.32 |
| chr15 | 47367889 | 47367890 | G | C | None |  |  | 1 | None | intergenic_variant | LOW |  | None |  | None | 0.33 |
| chr15 | 47694083 | 47694084 | C | T | SEMA6D |  | ENST00000558014 | 1 | None | intron_variant | LOW |  | None |  | None | 0.139534884 |
| chr15 | 48034182 | 48034183 | C | T | SEMA6D |  | ENST00000389433 | 0 | None | intron_variant | LOW |  | None |  | None | 0.256410256 |
| chr15 | 48406352 | 48406353 | C | T | None |  |  | 1 | None | intergenic_variant | LOW |  | None |  | None | 0.28 |
| chr15 | 48807447 | 48807448 | G | A | FBN1 |  | ENST00000316623 | 0 | None | intron_variant | LOW |  | None |  | None | 0.3 |
| chr15 | 53489555 | 53489556 | C | T | None |  |  | 0 | None | intergenic_variant | LOW |  | None |  | None | 0.166666667 |
| chr15 | 54218563 | 54218564 | C | T | None |  |  | 0 | None | intergenic_variant | LOW |  | None |  | None | 0.318181818 |
| chr15 | 57356696 | 57356697 | G | C | TCF12 |  | ENST00000557843 | 0 | None | intron_variant | LOW |  | None |  | None | 0.32 |
| chr15 | 59845153 | 59845154 | T | C | RP11-112N19.2 |  | ENST00000472975 | 0 | None | non_coding_exon_variant | LOW |  | None |  | None | 0.111111111 |
| chr15 | 59845173 | 59845174 | A | G | RP11-112N19.2 |  | ENST00000472975 | 0 | None | non_coding_exon_variant | LOW |  | None |  | None | 0.111111111 |
| chr15 | 59845176 | 59845177 | A | G | RP11-112N19.2 |  | ENST00000472975 | 0 | None | non_coding_exon_variant | LOW |  | None |  | None | 0.109090909 |
| chr15 | 60534145 | 60534146 | A | G | None |  |  | 0 | None | intergenic_variant | LOW |  | None |  | None | 0.22 |
| chr15 | 60534149 | 60534150 | T | C | None |  |  | 0 | None | intergenic_variant | LOW |  | None |  | None | 0.224489796 |
| chr15 | 60534176 | 60534177 | T | A | None |  |  | 0 | None | intergenic_variant | LOW |  | None |  | None | 0.156862745 |
| chr15 | 60691157 | 60691158 | C | T | ANXA2 |  | ENST00000557904 | 0 | None | intron_variant | LOW |  | None |  | None | 0.136363636 |
| chr15 | 60998671 | 60998672 | G | A | RORA |  | ENST00000335670 | 0 | None | intron_variant | LOW |  | None |  | None | 0.245614035 |
| chr15 | 61892585 | 61892586 | T | C | RP11-259A24.1 |  | ENST00000561182 | 0 | None | intron_variant | LOW |  | None |  | None | 0.220779221 |
| chr15 | 61937335 | 61937336 | C | G | RP11-507B12.1 |  | ENST00000560686 | 0 | None | intron_variant | LOW |  | None |  | None | 0.210526316 |
| chr15 | 62068642 | 62068643 | C | T | None |  |  | 0 | None | intergenic_variant | LOW |  | None |  | None | 0.121621622 |
| chr15 | 62876839 | 62876840 | G | T | TLN2 |  | ENST00000561311 | 0 | None | intron_variant | LOW |  | None |  | None | 0.152542373 |
| chr15 | 64226140 | 64226141 | C | G | DAPK2 |  | ENST00000261891 | 0 | None | intron_variant | LOW |  | None |  | None | 0.13559322 |
| chr15 | 64536324 | 64536325 | G | A | CSNK1G1 |  | ENST00000303032 | 0 | None | intron_variant | LOW |  | None |  | None | 0.126984127 |
| chr15 | 64800882 | 64800883 | A | T | ZNF609 |  | ENST00000326648 | 0 | None | intron_variant | LOW |  | None |  | None | 0.292682927 |
| chr15 | 65056873 | 65056874 | A | G | RBPMS2 |  | ENST00000300069 | 0 | None | intron_variant | LOW |  | None |  | None | 0.106666667 |
| chr15 | 65368046 | 65368047 | T | C | RASL12 |  | ENST00000434605 | 0 | None | intron_variant | LOW |  | None |  | None | 0.243902439 |
| chr15 | 65678650 | 65678651 | G | C | IGDCC4 |  | ENST00000352385 | 0 | None | intron_variant | LOW |  | None |  | None | 0.196428571 |
| chr15 | 65892587 | 65892588 | G | A | VWA9 |  | ENST00000567744 | 0 | None | intron_variant | LOW |  | None |  | None | 0.128571429 |
| chr15 | 65955858 | 65955859 | T | G | DENND4A |  | ENST00000431932 | 0 | None | intron_variant | LOW |  | None |  | None | 0.216666667 |
| chr15 | 66991773 | 66991774 | A | G | SMAD6 |  | ENST00000457357 | 0 | None | upstream_gene_variant | LOW |  | None |  | None | 0.156862745 |
| chr15 | 67546278 | 67546279 | C | G | AAGAB |  | ENST00000561452 | 0 | None | intron_variant | LOW |  | None |  | None | 0.116666667 |
| chr15 | 67895526 | 67895527 | C | A | MAP2K5 |  | ENST00000395476 | 0 | None | intron_variant | LOW |  | None |  | None | 0.144927536 |
| chr15 | 67936459 | 67936460 | G | A | MAP2K5 |  | ENST00000178640 | 0 | None | intron_variant | LOW |  | None |  | None | 0.185185185 |
| chr15 | 67954162 | 67954163 | T | A | MAP2K5 |  | ENST00000354498 | 0 | None | intron_variant | LOW |  | None |  | None | 0.224137931 |
| chr15 | 68881990 | 68881991 | G | T | CORO2B |  | ENST00000566799 | 0 | None | intron_variant | LOW |  | None |  | None | 0.176470588 |
| chr15 | 70362712 | 70362713 | C | T | TLE3 |  | ENST00000557984 | 0 | None | intron_variant | LOW |  | None |  | None | 0.202898551 |
| chr15 | 72354083 | 72354084 | T | C | MYO9A |  | ENST00000444904 | 0 | None | intron_variant | LOW |  | None |  | None | 0.164556962 |
| chr15 | 72564134 | 72564135 | T | C | PARP6 |  | ENST00000568360 | 0 | None | intron_variant | LOW |  | None |  | None | 0.320754717 |
| chr15 | 73619465 | 73619466 | G | T | HCN4 |  | ENST00000261917 | 0 | None | intron_variant | LOW |  | None |  | None | 0.174603175 |
| chr15 | 74500380 | 74500381 | G | T | STRA6 |  | ENST00000574278 | 0 | None | 5_prime_UTR_variant | LOW |  | None |  | None | 0.142857143 |
| chr15 | 74519456 | 74519457 | G | A | CCDC33 |  | ENST00000321288 | 0 | None | intron_variant | LOW |  | None |  | None | 0.189655172 |
| chr15 | 74598701 | 74598702 | C | T | CCDC33 |  | ENST00000558659 | 0 | None | intron_variant | LOW |  | None |  | None | 0.179104478 |
| chr15 | 74634718 | 74634719 | G | A | CYP11A1 |  | ENST00000419019 | 0 | None | intron_variant | LOW |  | None |  | None | 0.133333333 |
| chr15 | 75450080 | 75450081 | C | G | None |  |  | 0 | None | intergenic_variant | LOW |  | None |  | None | 0.140625 |
| chr15 | 75545891 | 75545892 | T | G | MKI67IPP4 |  | ENST00000567275 | 0 | None | upstream_gene_variant | LOW |  | None |  | None | 0.206896552 |
| chr15 | 75993738 | 75993739 | T | C | CSPG4 |  | ENST00000308508 | 0 | None | intron_variant | LOW |  | None |  | None | 0.108108108 |
| chr15 | 76627202 | 76627203 | C | T | ISL2 |  | ENST00000290759 | 0 | None | upstream_gene_variant | LOW |  | None |  | None | 0.2 |
| chr15 | 77888793 | 77888794 | A | G | RP11-307C19.2 |  | ENST00000561123 | 0 | None | intron_variant | LOW |  | None |  | None | 0.196078431 |
| chr15 | 78121886 | 78121887 | C | T | None |  |  | 1 | None | intergenic_variant | LOW |  | None |  | None | 0.169491525 |
| chr15 | 78135703 | 78135704 | C | T | None |  |  | 0 | None | intergenic_variant | LOW |  | None |  | None | 0.103448276 |
| chr15 | 78979366 | 78979367 | T | C | RP11-160C18.2 |  | ENST00000569846 | 0 | None | intron_variant | LOW |  | None |  | None | 0.209677419 |
| chr15 | 79058338 | 79058339 | A | C | ADAMTS7 | V/G | ENST00000388820 | 1 | None | missense_variant | MED | benign | 0 | tolerated | 0.39 | 0.128571429 |
| chr15 | 79480128 | 79480129 | G | T | RP11-17L5.4 |  | ENST00000560872 | 0 | None | downstream_gene_variant | LOW |  | None |  | None | 0.275862069 |
| chr15 | 79482832 | 79482833 | G | A | RP11-17L5.4 |  | ENST00000559225 | 0 | None | downstream_gene_variant | LOW |  | None |  | None | 0.245901639 |
| chr15 | 81579144 | 81579145 | C | T | IL16 |  | ENST00000559383 | 0 | None | intron_variant | LOW |  | None |  | None | 0.296296296 |
| chr15 | 81988439 | 81988440 | A | T | RP11-499F3.2 |  | ENST00000560054 | 0 | None | intron_variant | LOW |  | None |  | None | 0.203389831 |
| chr15 | 82089646 | 82089647 | A | C | RP11-499F3.2 |  | ENST00000560097 | 0 | None | intron_variant | LOW |  | None |  | None | 0.175438596 |
| chr15 | 82284597 | 82284598 | G | C | RP11-276M12.1 |  | ENST00000559299 | 0 | None | intron_variant | LOW |  | None |  | None | 0.217391304 |
| chr15 | 82630808 | 82630809 | C | G | GOLGA6L10 |  | ENST00000439287 | 0 | None | downstream_gene_variant | LOW |  | None |  | None | 0.25 |
| chr15 | 83377900 | 83377901 | G | C | AP3B2 |  | ENST00000535359 | 0 | None | intron_variant | LOW |  | None |  | None | 0.166666667 |
| chr15 | 83536264 | 83536265 | A | C | HOMER2 |  | ENST00000304231 | 0 | None | intron_variant | LOW |  | None |  | None | 0.240740741 |
| chr15 | 83905677 | 83905678 | T | G | RP11-382A20.4 |  | ENST00000565495 | 1 | None | intron_variant | LOW |  | None |  | None | 0.24 |
| chr15 | 85952581 | 85952582 | T | C | AKAP13 |  | ENST00000560302 | 0 | None | intron_variant | LOW |  | None |  | None | 0.226415094 |
| chr15 | 87162145 | 87162146 | T | A | AGBL1 |  | ENST00000389298 | 0 | None | intron_variant | LOW |  | None |  | None | 0.193548387 |
| chr15 | 88259799 | 88259800 | G | C | None |  |  | 0 | None | intergenic_variant | LOW |  | None |  | None | 0.209302326 |
| chr15 | 88769008 | 88769009 | G | A | NTRK3 |  | ENST00000394480 | 1 | None | intron_variant | LOW |  | None |  | None | 0.178571429 |
| chr15 | 89139701 | 89139702 | A | G | RP11-97O12.2 |  | ENST00000506090 | 0 | None | intron_variant | LOW |  | None |  | None | 0.294117647 |
| chr15 | 89978583 | 89978584 | G | A | None |  |  | 0 | None | intergenic_variant | LOW |  | None |  | None | 0.122807018 |
| chr15 | 91903563 | 91903564 | G | A | None |  |  | 0 | None | intergenic_variant | LOW |  | None |  | None | 0.245283019 |
| chr15 | 92012489 | 92012490 | G | T | RP11-661P17.1 |  | ENST00000555947 | 0 | None | intron_variant | LOW |  | None |  | None | 0.230769231 |
| chr15 | 92436721 | 92436722 | A | C | SLCO3A1 |  | ENST00000318445 | 0 | None | intron_variant | LOW |  | None |  | None | 0.272727273 |
| chr15 | 93901071 | 93901072 | T | C | RP11-266O8.1 |  | ENST00000543286 | 0 | None | intron_variant | LOW |  | None |  | None | 0.224137931 |
| chr15 | 94249931 | 94249932 | G | A | RP11-739G5.1 |  | ENST00000554318 | 0 | None | intron_variant | LOW |  | None |  | None | 0.16 |
| chr15 | 94251330 | 94251331 | G | A | RP11-739G5.1 |  | ENST00000554318 | 0 | None | intron_variant | LOW |  | None |  | None | 0.236363636 |
| chr15 | 94369388 | 94369389 | G | A | CTD-3049M7.1 |  | ENST00000553818 | 0 | None | intron_variant | LOW |  | None |  | None | 0.123076923 |
| chr15 | 95143011 | 95143012 | T | C | RP11-57P19.1 |  | ENST00000560391 | 0 | None | upstream_gene_variant | LOW |  | None |  | None | 0.166666667 |
| chr15 | 95150436 | 95150437 | C | T | None |  |  | 0 | None | intergenic_variant | LOW |  | None |  | None | 0.214285714 |
| chr15 | 95183648 | 95183649 | C | G | None |  |  | 0 | None | intergenic_variant | LOW |  | None |  | None | 0.184615385 |
| chr15 | 95247952 | 95247953 | T | C | None |  |  | 0 | None | intergenic_variant | LOW |  | None |  | None | 0.211538462 |
| chr15 | 95634208 | 95634209 | G | A | RP11-255M2.2 |  | ENST00000554787 | 0 | None | intron_variant | LOW |  | None |  | None | 0.116666667 |
| chr15 | 96508895 | 96508896 | T | A | None |  |  | 0 | None | intergenic_variant | LOW |  | None |  | None | 0.280701754 |
| chr15 | 96900982 | 96900983 | A | G | AC087477.1 |  | ENST00000600790 | 0 | None | upstream_gene_variant | LOW |  | None |  | None | 0.264150943 |
| chr15 | 97124296 | 97124297 | A | T | None |  |  | 0 | None | intergenic_variant | LOW |  | None |  | None | 0.157894737 |
| chr15 | 97141067 | 97141068 | A | T | None |  |  | 0 | None | intergenic_variant | LOW |  | None |  | None | 0.111111111 |
| chr15 | 97595583 | 97595584 | A | G | None |  |  | 1 | None | intergenic_variant | LOW |  | None |  | None | 0.15 |
| chr15 | 97924235 | 97924236 | G | A | CTD-2147F2.2 |  | ENST00000558621 | 0 | None | intron_variant | LOW |  | None |  | None | 0.169491525 |
| chr15 | 98081536 | 98081537 | G | T | None |  |  | 1 | None | intergenic_variant | LOW |  | None |  | None | 0.28 |
| chr15 | 98951495 | 98951496 | C | T | None |  |  | 0 | None | intergenic_variant | LOW |  | None |  | None | 0.166666667 |
| chr15 | 100214698 | 100214699 | A | G | MEF2A | P | ENST00000354410 | 0 | None | synonymous_variant | LOW |  | None |  | None | 0.185185185 |
| chr15 | 100231085 | 100231086 | T | G | MEF2A |  | ENST00000557942 | 0 | None | intron_variant | LOW |  | None |  | None | 0.180327869 |
| chr15 | 101501462 | 101501463 | G | A | LRRK1 |  | ENST00000284395 | 0 | None | intron_variant | LOW |  | None |  | None | 0.235294118 |
| chr15 | 101742317 | 101742318 | G | C | CHSY1 |  | ENST00000254190 | 1 | None | intron_variant | LOW |  | None |  | None | 0.111111111 |
| chr15 | 102286565 | 102286566 | C | T | RP11-89K11.1 |  | ENST00000560292 | 0 | None | upstream_gene_variant | LOW |  | None |  | None | 0.170731707 |
| chr15 | 102327681 | 102327682 | A | G | DNM1P47 |  | ENST00000560757 | 1 | None | intron_variant | LOW |  | None |  | None | 0.113924051 |
| chr15 | 102333153 | 102333154 | T | C | DNM1P47 |  | ENST00000560757 | 1 | None | intron_variant | LOW |  | None |  | None | 0.166666667 |
| chr16 | 191701 | 191702 | A | C | NPRL3 |  | ENST00000399951 | 0 | None | upstream_gene_variant | LOW |  | None |  | None | 0.254237288 |
| chr16 | 992667 | 992668 | T | A | LMF1 |  | ENST00000570014 | 1 | None | intron_variant | LOW |  | None |  | None | 0.14 |
| chr16 | 1037844 | 1037845 | G | A | SOX8 |  | ENST00000293894 | 0 | None | downstream_gene_variant | LOW |  | None |  | None | 0.183098592 |
| chr16 | 1139935 | 1139936 | C | G | C1QTNF8 |  | ENST00000328449 | 0 | None | downstream_gene_variant | LOW |  | None |  | None | 0.144927536 |
| chr16 | 1970184 | 1970185 | G | C | HS3ST6 |  | ENST00000293937 | 1 | None | upstream_gene_variant | LOW |  | None |  | None | 0.115384615 |
| chr16 | 2030890 | 2030891 | G | A | TBL3 |  | ENST00000568546 | 0 | None | 3_prime_UTR_variant | LOW |  | None |  | None | 0.146666667 |
| chr16 | 2265693 | 2265694 | C | G | BRICD5 |  | ENST00000562360 | 0 | None | upstream_gene_variant | LOW |  | None |  | None | 0.12 |
| chr16 | 2442726 | 2442727 | G | C | ABCA17P |  | ENST00000469908 | 0 | None | intron_variant | LOW |  | None |  | None | 0.1 |
| chr16 | 3061592 | 3061593 | A | G | CLDN9 |  | ENST00000445369 | 0 | None | upstream_gene_variant | LOW |  | None |  | None | 0.283018868 |
| chr16 | 3356903 | 3356904 | T | C | ZNF75A |  | ENST00000574298 | 0 | None | intron_variant | LOW |  | None |  | None | 0.322580645 |
| chr16 | 4019157 | 4019158 | C | T | ADCY9 |  | ENST00000576936 | 0 | None | intron_variant | LOW |  | None |  | None | 0.283950617 |
| chr16 | 4205219 | 4205220 | C | A | None |  |  | 0 | None | intergenic_variant | LOW |  | None |  | None | 0.206349206 |
| chr16 | 4284079 | 4284080 | C | T | SRL |  | ENST00000399609 | 0 | None | intron_variant | LOW |  | None |  | None | 0.208333333 |
| chr16 | 4617954 | 4617955 | T | C | C16orf96 |  | ENST00000444310 | 0 | None | intron_variant | LOW |  | None |  | None | 0.125 |
| chr16 | 5080665 | 5080666 | G | C | NAGPA |  | ENST00000563578 | 0 | None | intron_variant | LOW |  | None |  | None | 0.163636364 |
| chr16 | 5270291 | 5270292 | A | T | RP11-382N13.2 |  | ENST00000566684 | 0 | None | intron_variant | LOW |  | None |  | None | 0.280701754 |
| chr16 | 5289829 | 5289830 | G | C | RP11-420N3.2 |  | ENST00000569895 | 0 | None | non_coding_exon_variant | LOW |  | None |  | None | 0.219512195 |
| chr16 | 5289887 | 5289888 | A | G | RP11-420N3.2 |  | ENST00000569895 | 1 | None | non_coding_exon_variant | LOW |  | None |  | None | 0.204301075 |
| chr16 | 5289910 | 5289911 | C | T | RP11-420N3.2 |  | ENST00000569895 | 1 | None | non_coding_exon_variant | LOW |  | None |  | None | 0.217391304 |
| chr16 | 5289944 | 5289945 | C | G | RP11-420N3.2 |  | ENST00000569895 | 0 | None | non_coding_exon_variant | LOW |  | None |  | None | 0.120879121 |
| chr16 | 5867310 | 5867311 | C | T | RP11-420N3.2 |  | ENST00000569895 | 0 | None | intron_variant | LOW |  | None |  | None | 0.173913043 |
| chr16 | 6241292 | 6241293 | C | T | RBFOX1 |  | ENST00000553186 | 0 | None | intron_variant | LOW |  | None |  | None | 0.16 |
| chr16 | 6921776 | 6921777 | C | A | RBFOX1 |  | ENST00000550418 | 0 | None | intron_variant | LOW |  | None |  | None | 0.229166667 |
| chr16 | 7266000 | 7266001 | G | C | RBFOX1 |  | ENST00000547605 | 0 | None | intron_variant | LOW |  | None |  | None | 0.195121951 |
| chr16 | 7271753 | 7271754 | C | G | RBFOX1 |  | ENST00000547338 | 0 | None | intron_variant | LOW |  | None |  | None | 0.16 |
| chr16 | 7424807 | 7424808 | G | A | RBFOX1 |  | ENST00000436368 | 0 | None | intron_variant | LOW |  | None |  | None | 0.229166667 |
| chr16 | 7461585 | 7461586 | A | T | RBFOX1 |  | ENST00000535565 | 0 | None | intron_variant | LOW |  | None |  | None | 0.24137931 |
| chr16 | 7527593 | 7527594 | C | T | RBFOX1 |  | ENST00000547605 | 0 | None | intron_variant | LOW |  | None |  | None | 0.140625 |
| chr16 | 7849554 | 7849555 | C | T | None |  |  | 0 | None | intergenic_variant | LOW |  | None |  | None | 0.209677419 |
| chr16 | 7944558 | 7944559 | A | G | CTD-2535I10.1 |  | ENST00000567103 | 1 | None | upstream_gene_variant | LOW |  | None |  | None | 0.192307692 |
| chr16 | 7970983 | 7970984 | A | C | None |  |  | 1 | None | intergenic_variant | LOW |  | None |  | None | 0.098360656 |
| chr16 | 8015262 | 8015263 | A | T | None |  |  | 0 | None | intergenic_variant | LOW |  | None |  | None | 0.19047619 |
| chr16 | 8104047 | 8104048 | C | A | None |  |  | 0 | None | intergenic_variant | LOW |  | None |  | None | 0.19047619 |
| chr16 | 8301438 | 8301439 | G | T | None |  |  | 0 | None | intergenic_variant | LOW |  | None |  | None | 0.151515152 |
| chr16 | 8325844 | 8325845 | G | A | RP11-279O17.2 |  | ENST00000562361 | 0 | None | downstream_gene_variant | LOW |  | None |  | None | 0.274509804 |
| chr16 | 8461973 | 8461974 | C | T | None |  |  | 0 | None | intergenic_variant | LOW |  | None |  | None | 0.166666667 |
| chr16 | 9048067 | 9048068 | C | A | USP7 |  | ENST00000344836 | 0 | None | intron_variant | LOW |  | None |  | None | 0.145454545 |
| chr16 | 9314003 | 9314004 | A | C | None |  |  | 0 | None | intergenic_variant | LOW |  | None |  | None | 0.1875 |
| chr16 | 9438291 | 9438292 | G | A | None |  |  | 0 | None | intergenic_variant | LOW |  | None |  | None | 0.140350877 |
| chr16 | 9521844 | 9521845 | G | A | None |  |  | 0 | None | intergenic_variant | LOW |  | None |  | None | 0.244444444 |
| chr16 | 9525337 | 9525338 | A | G | None |  |  | 0 | None | intergenic_variant | LOW |  | None |  | None | 0.153846154 |
| chr16 | 9542333 | 9542334 | C | T | RP11-243A14.3 |  | ENST00000570118 | 0 | None | intron_variant | LOW |  | None |  | None | 0.127659574 |
| chr16 | 10222484 | 10222485 | G | T | GRIN2A |  | ENST00000404927 | 0 | None | intron_variant | LOW |  | None |  | None | 0.25 |
| chr16 | 11541606 | 11541607 | C | T | CTD-3088G3.8 |  | ENST00000598234 | 0 | None | intron_variant | LOW |  | None |  | None | 0.132075472 |
| chr16 | 13211478 | 13211479 | C | T | SHISA9 |  | ENST00000558583 | 0 | None | intron_variant | LOW |  | None |  | None | 0.203703704 |
| chr16 | 13379740 | 13379741 | G | T | U91319.1 |  | ENST00000571619 | 0 | None | intron_variant | LOW |  | None |  | None | 0.339285714 |
| chr16 | 13784866 | 13784867 | G | A | None |  |  | 0 | None | intergenic_variant | LOW |  | None |  | None | 0.276595745 |
| chr16 | 14348108 | 14348109 | G | T | MKL2 |  | ENST00000574045 | 0 | None | intron_variant | LOW |  | None |  | None | 0.322033898 |
| chr16 | 14490768 | 14490769 | A | G | None |  |  | 0 | None | intergenic_variant | LOW |  | None |  | None | 0.152777778 |
| chr16 | 14490770 | 14490771 | A | G | None |  |  | 0 | None | intergenic_variant | LOW |  | None |  | None | 0.14084507 |
| chr16 | 15592932 | 15592933 | G | T | C16orf45 |  | ENST00000300006 | 0 | None | intron_variant | LOW |  | None |  | None | 0.183098592 |
| chr16 | 15613681 | 15613682 | C | T | C16orf45 |  | ENST00000566490 | 0 | None | intron_variant | LOW |  | None |  | None | 0.140625 |
| chr16 | 15628395 | 15628396 | G | A | C16orf45 |  | ENST00000564389 | 0 | None | intron_variant | LOW |  | None |  | None | 0.23943662 |
| chr16 | 15770046 | 15770047 | C | G | NDE1 |  | ENST00000396355 | 0 | None | intron_variant | LOW |  | None |  | None | 0.152173913 |
| chr16 | 15778620 | 15778621 | A | G | NDE1 |  | ENST00000342673 | 0 | None | intron_variant | LOW |  | None |  | None | 0.185185185 |
| chr16 | 15946722 | 15946723 | C | T | MYH11 |  | ENST00000576790 | 0 | None | intron_variant | LOW |  | None |  | None | 0.203125 |
| chr16 | 16557814 | 16557815 | T | C | None |  |  | 0 | None | intergenic_variant | LOW |  | None |  | None | 0.18 |
| chr16 | 17033010 | 17033011 | A | G | None |  |  | 0 | None | intergenic_variant | LOW |  | None |  | None | 0.25 |
| chr16 | 17129081 | 17129082 | C | G | None |  |  | 0 | None | intergenic_variant | LOW |  | None |  | None | 0.208955224 |
| chr16 | 17195902 | 17195903 | G | C | XYLT1 |  | ENST00000261381 | 0 | None | 3_prime_UTR_variant | LOW |  | None |  | None | 0.220338983 |
| chr16 | 17786665 | 17786666 | G | A | None |  |  | 0 | None | intergenic_variant | LOW |  | None |  | None | 0.238095238 |
| chr16 | 17893066 | 17893067 | C | A | None |  |  | 0 | None | intergenic_variant | LOW |  | None |  | None | 0.183673469 |
| chr16 | 18161909 | 18161910 | C | A | CTA-481E9.4 |  | ENST00000567304 | 0 | None | intron_variant | LOW |  | None |  | None | 0.173076923 |
| chr16 | 19584773 | 19584774 | G | T | C16orf62 |  | ENST00000417362 | 0 | None | intron_variant | LOW |  | None |  | None | 0.145454545 |
| chr16 | 19865653 | 19865654 | C | G | IQCK |  | ENST00000320394 | 0 | None | intron_variant | LOW |  | None |  | None | 0.205882353 |
| chr16 | 19954755 | 19954756 | G | C | None |  |  | 0 | None | intergenic_variant | LOW |  | None |  | None | 0.227272727 |
| chr16 | 20438323 | 20438324 | T | G | ACSM5 |  | ENST00000331849 | 1 | None | intron_variant | LOW |  | None |  | None | 0.215686275 |
| chr16 | 20578225 | 20578226 | A | G | ACSM2B |  | ENST00000568882 | 0 | None | intron_variant | LOW |  | None |  | None | 0.24137931 |
| chr16 | 20977327 | 20977328 | C | G | DNAH3 |  | ENST00000415178 | 0 | None | intron_variant | LOW |  | None |  | None | 0.23255814 |
| chr16 | 21586811 | 21586812 | G | A | None |  |  | 0 | None | intergenic_variant | LOW |  | None |  | None | 0.136690647 |
| chr16 | 21929685 | 21929686 | C | G | RP11-645C24.2 |  | ENST00000517529 | 1 | None | intron_variant | LOW |  | None |  | None | 0.150943396 |
| chr16 | 21946956 | 21946957 | A | G | RP11-645C24.4 |  | ENST00000550410 | 1 | None | upstream_gene_variant | LOW |  | None |  | None | 0.108108108 |
| chr16 | 22963743 | 22963744 | T | C | None |  |  | 0 | None | intergenic_variant | LOW |  | None |  | None | 0.272727273 |
| chr16 | 23855351 | 23855352 | C | T | PRKCB |  | ENST00000303531 | 0 | None | intron_variant | LOW |  | None |  | None | 0.279069767 |
| chr16 | 24064596 | 24064597 | G | A | PRKCB |  | ENST00000321728 | 0 | None | intron_variant | LOW |  | None |  | None | 0.225352113 |
| chr16 | 24745153 | 24745154 | G | A | TNRC6A |  | ENST00000395799 | 0 | None | intron_variant | LOW |  | None |  | None | 0.153846154 |
| chr16 | 25468803 | 25468804 | A | C | CYCSP39 |  | ENST00000565821 | 0 | None | upstream_gene_variant | LOW |  | None |  | None | 0.2 |
| chr16 | 25816446 | 25816447 | G | T | HS3ST4 |  | ENST00000331351 | 0 | None | intron_variant | LOW |  | None |  | None | 0.153846154 |
| chr16 | 26008387 | 26008388 | T | G | HS3ST4 |  | ENST00000331351 | 0 | None | intron_variant | LOW |  | None |  | None | 0.160714286 |
| chr16 | 26113485 | 26113486 | A | C | HS3ST4 |  | ENST00000331351 | 0 | None | intron_variant | LOW |  | None |  | None | 0.140350877 |
| chr16 | 28334791 | 28334792 | C | G | SBK1 |  | ENST00000341901 | 0 | None | 3_prime_UTR_variant | LOW |  | None |  | None | 0.214285714 |
| chr16 | 28636757 | 28636758 | A | T | SULT1A1 |  | ENST00000350842 | 1 | None | upstream_gene_variant | LOW |  | None |  | None | 0.216666667 |
| chr16 | 28898985 | 28898986 | G | A | ATP2A1 | G/R | ENST00000395503 | 0 | COSM673841 | missense_variant | MED | probably_damaging | 1 | deleterious | 0.02 | 0.261538462 |
| chr16 | 29163216 | 29163217 | C | T | RP11-426C22.5 |  | ENST00000562902 | 1 | None | intron_variant | LOW |  | None |  | None | 0.205128205 |
| chr16 | 29305498 | 29305499 | G | A | SNX29P2 |  | ENST00000604430 | 0 | None | intron_variant | LOW |  | None |  | None | 0.183098592 |
| chr16 | 30067613 | 30067614 | G | A | ALDOA |  | ENST00000338110 | 0 | None | intron_variant | LOW |  | None |  | None | 0.169811321 |
| chr16 | 30067627 | 30067628 | C | T | ALDOA |  | ENST00000566897 | 0 | None | intron_variant | LOW |  | None |  | None | 0.163636364 |
| chr16 | 30772478 | 30772479 | A | G | PHKG2 |  | ENST00000563588 | 0 | None | 3_prime_UTR_variant | LOW |  | None |  | None | 0.173913043 |
| chr16 | 31028669 | 31028670 | G | T | None |  |  | 0 | None | intergenic_variant | LOW |  | None |  | None | 0.133333333 |
| chr16 | 31430292 | 31430293 | C | T | ITGAD |  | ENST00000389202 | 0 | None | intron_variant | LOW |  | None |  | None | 0.146341463 |
| chr16 | 31615502 | 31615503 | T | G | ZNF720P1 |  | ENST00000562403 | 0 | None | upstream_gene_variant | LOW |  | None |  | None | 0.157894737 |
| chr16 | 31976758 | 31976759 | T | A | RP11-170L3.7 |  | ENST00000356559 | 0 | None | intron_variant | LOW |  | None |  | None | 0.230769231 |
| chr16 | 32033891 | 32033892 | T | C | RP11-1166P10.6 |  | ENST00000566806 | 0 | None | intron_variant | LOW |  | None |  | None | 0.18 |
| chr16 | 32080795 | 32080796 | G | T | RP11-1166P10.6 |  | ENST00000566806 | 0 | None | intron_variant | LOW |  | None |  | None | 0.206896552 |
| chr16 | 32115575 | 32115576 | G | A | HERC2P4 |  | ENST00000564145 | 0 | None | intron_variant | LOW |  | None |  | None | 0.131067961 |
| chr16 | 32129643 | 32129644 | G | T | HERC2P4 |  | ENST00000564145 | 1 | None | non_coding_exon_variant | LOW |  | None |  | None | 0.085365854 |
| chr16 | 32135898 | 32135899 | C | T | HERC2P4 |  | ENST00000564145 | 1 | None | intron_variant | LOW |  | None |  | None | 0.108108108 |
| chr16 | 32137655 | 32137656 | C | T | HERC2P4 |  | ENST00000564145 | 0 | None | intron_variant | LOW |  | None |  | None | 0.076923077 |
| chr16 | 32138293 | 32138294 | G | A | HERC2P4 |  | ENST00000564145 | 1 | None | intron_variant | LOW |  | None |  | None | 0.158536585 |
| chr16 | 32379558 | 32379559 | G | A | RP11-17M15.4 |  | ENST00000562853 | 0 | None | upstream_gene_variant | LOW |  | None |  | None | 0.079646018 |
| chr16 | 32413355 | 32413356 | G | T | RP11-626K17.5 |  | ENST00000562908 | 0 | None | intron_variant | LOW |  | None |  | None | 0.266666667 |
| chr16 | 32424591 | 32424592 | G | C | RP11-626K17.5 |  | ENST00000562908 | 0 | None | intron_variant | LOW |  | None |  | None | 0.171875 |
| chr16 | 32431880 | 32431881 | C | T | RP11-626K17.5 |  | ENST00000562908 | 0 | None | intron_variant | LOW |  | None |  | None | 0.118421053 |
| chr16 | 32438103 | 32438104 | C | T | RP11-626K17.5 |  | ENST00000562908 | 0 | None | non_coding_exon_variant | LOW |  | None |  | None | 0.183908046 |
| chr16 | 32495814 | 32495815 | G | T | None |  |  | 0 | None | intergenic_variant | LOW |  | None |  | None | 0.136363636 |
| chr16 | 32531545 | 32531546 | A | T | None |  |  | 0 | None | intergenic_variant | LOW |  | None |  | None | 0.112149533 |
| chr16 | 32550229 | 32550230 | T | C | None |  |  | 0 | None | intergenic_variant | LOW |  | None |  | None | 0.083333333 |
| chr16 | 32563456 | 32563457 | C | T | None |  |  | 0 | None | intergenic_variant | LOW |  | None |  | None | 0.06 |
| chr16 | 32822251 | 32822252 | G | A | RP11-67H24.2 |  | ENST00000569859 | 0 | None | intron_variant | LOW |  | None |  | None | 0.071428571 |
| chr16 | 33038958 | 33038959 | A | G | RP11-19N8.2 |  | ENST00000567619 | 0 | None | non_coding_exon_variant | LOW |  | None |  | None | 0.06 |
| chr16 | 33038958 | 33038959 | A | T | RP11-19N8.2 |  | ENST00000567619 | 0 | None | non_coding_exon_variant | LOW |  | None |  | None | 0.18 |
| chr16 | 33342808 | 33342809 | C | G | RP11-989E6.10 |  | ENST00000568752 | 0 | None | downstream_gene_variant | LOW |  | None |  | None | 0.169014085 |
| chr16 | 33413794 | 33413795 | G | T | RP11-293B20.2 |  | ENST00000566346 | 0 | None | intron_variant | LOW |  | None |  | None | 0.181818182 |
| chr16 | 33427198 | 33427199 | C | A | RP11-293B20.2 |  | ENST00000566346 | 0 | None | downstream_gene_variant | LOW |  | None |  | None | 0.082191781 |
| chr16 | 33432934 | 33432935 | G | T | None |  |  | 0 | None | intergenic_variant | LOW |  | None |  | None | 0.214285714 |
| chr16 | 33518097 | 33518098 | G | T | None |  |  | 1 | None | intergenic_variant | LOW |  | None |  | None | 0.0703125 |
| chr16 | 33518707 | 33518708 | G | T | None |  |  | 1 | None | intergenic_variant | LOW |  | None |  | None | 0.09 |
| chr16 | 33518707 | 33518708 | G | A | None |  |  | 1 | None | intergenic_variant | LOW |  | None |  | None | 0.20 |
| chr16 | 33521515 | 33521516 | G | T | None |  |  | 0 | None | intergenic_variant | LOW |  | None |  | None | 0.092105263 |
| chr16 | 33579230 | 33579231 | A | C | None |  |  | 1 | None | intergenic_variant | LOW |  | None |  | None | 0.142857143 |
| chr16 | 33579320 | 33579321 | G | A | None |  |  | 0 | None | intergenic_variant | LOW |  | None |  | None | 0.155172414 |
| chr16 | 33585637 | 33585638 | G | A | None |  |  | 1 | None | intergenic_variant | LOW |  | None |  | None | 0.22972973 |
| chr16 | 33693995 | 33693996 | A | T | None |  |  | 1 | None | intergenic_variant | LOW |  | None |  | None | 0.172413793 |
| chr16 | 33870860 | 33870861 | C | T | None |  |  | 0 | None | intergenic_variant | LOW |  | None |  | None | 0.092307692 |
| chr16 | 33894406 | 33894407 | T | A | None |  |  | 1 | None | intergenic_variant | LOW |  | None |  | None | 0.235294118 |
| chr16 | 34180299 | 34180300 | G | C | None |  |  | 0 | None | intergenic_variant | LOW |  | None |  | None | 0.054054054 |
| chr16 | 34182039 | 34182040 | A | G | None |  |  | 1 | None | intergenic_variant | LOW |  | None |  | None | 0.074074074 |
| chr16 | 34188498 | 34188499 | G | T | None |  |  | 0 | None | intergenic_variant | LOW |  | None |  | None | 0.18 |
| chr16 | 34188498 | 34188499 | G | A | None |  |  | 1 | None | intergenic_variant | LOW |  | None |  | None | 0.23 |
| chr16 | 34189014 | 34189015 | T | A | None |  |  | 0 | None | intergenic_variant | LOW |  | None |  | None | 0.088235294 |
| chr16 | 34215080 | 34215081 | T | C | CTD-2144E22.8 |  | ENST00000568121 | 0 | None | upstream_gene_variant | LOW |  | None |  | None | 0.275 |
| chr16 | 34233138 | 34233139 | G | T | AC135776.1 |  | ENST00000541787 | 0 | None | downstream_gene_variant | LOW |  | None |  | None | 0.240740741 |
| chr16 | 34865146 | 34865147 | A | T | None |  |  | 0 | None | intergenic_variant | LOW |  | None |  | None | 0.164179104 |
| chr16 | 35166703 | 35166704 | G | T | None |  |  | 0 | None | intergenic_variant | LOW |  | None |  | None | 0.133333333 |
| chr16 | 35170921 | 35170922 | G | T | None |  |  | 1 | None | intergenic_variant | LOW |  | None |  | None | 0.270833333 |
| chr16 | 35258913 | 35258914 | A | T | None |  |  | 0 | None | intergenic_variant | LOW |  | None |  | None | 0.229166667 |
| chr16 | 35280183 | 35280184 | G | A | None |  |  | 0 | None | intergenic_variant | LOW |  | None |  | None | 0.107142857 |
| chr16 | 46457379 | 46457380 | A | G | None |  |  | 1 | None | intergenic_variant | LOW |  | None |  | None | 0.170212766 |
| chr16 | 46470728 | 46470729 | C | G | None |  |  | 0 | None | intergenic_variant | LOW |  | None |  | None | 0.086206897 |
| chr16 | 46474425 | 46474426 | G | A | None |  |  | 0 | None | intergenic_variant | LOW |  | None |  | None | 0.114285714 |
| chr16 | 46474441 | 46474442 | T | C | None |  |  | 0 | None | intergenic_variant | LOW |  | None |  | None | 0.107142857 |
| chr16 | 46475441 | 46475442 | C | T | None |  |  | 0 | None | intergenic_variant | LOW |  | None |  | None | 0.108433735 |
| chr16 | 46481578 | 46481579 | T | G | None |  |  | 0 | None | intergenic_variant | LOW |  | None |  | None | 0.102803738 |
| chr16 | 46481599 | 46481600 | C | G | None |  |  | 1 | None | intergenic_variant | LOW |  | None |  | None | 0.104166667 |
| chr16 | 46481614 | 46481615 | A | G | None |  |  | 1 | None | intergenic_variant | LOW |  | None |  | None | 0.105263158 |
| chr16 | 46487658 | 46487659 | C | G | None |  |  | 0 | None | intergenic_variant | LOW |  | None |  | None | 0.25 |
| chr16 | 46487672 | 46487673 | A | T | None |  |  | 0 | None | intergenic_variant | LOW |  | None |  | None | 0.244897959 |
| chr16 | 46493117 | 46493118 | T | C | None |  |  | 0 | None | intergenic_variant | LOW |  | None |  | None | 0.067114094 |
| chr16 | 46493178 | 46493179 | A | T | None |  |  | 0 | None | intergenic_variant | LOW |  | None |  | None | 0.092198582 |
| chr16 | 46493183 | 46493184 | G | C | None |  |  | 1 | None | intergenic_variant | LOW |  | None |  | None | 0.15 |
| chr16 | 46493183 | 46493184 | G | T | None |  |  | 1 | None | intergenic_variant | LOW |  | None |  | None | 0.54 |
| chr16 | 46495217 | 46495218 | T | G | None |  |  | 1 | None | intergenic_variant | LOW |  | None |  | None | 0.123076923 |
| chr16 | 46499612 | 46499613 | G | A | ANKRD26P1 |  | ENST00000571006 | 1 | None | downstream_gene_variant | LOW |  | None |  | None | 0.16 |
| chr16 | 46499612 | 46499613 | G | T | ANKRD26P1 |  | ENST00000571006 | 0 | None | downstream_gene_variant | LOW |  | None |  | None | 0.22 |
| chr16 | 46615624 | 46615625 | A | G | SHCBP1 |  | ENST00000303383 | 0 | None | 3_prime_UTR_variant | LOW |  | None |  | None | 0.263157895 |
| chr16 | 47044147 | 47044148 | C | A | RP11-169E6.4 |  | ENST00000564193 | 0 | None | intron_variant | LOW |  | None |  | None | 0.230769231 |
| chr16 | 47046786 | 47046787 | A | T | RP11-169E6.4 |  | ENST00000564193 | 0 | None | intron_variant | LOW |  | None |  | None | 0.259259259 |
| chr16 | 47186219 | 47186220 | C | A | ITFG1 |  | ENST00000544001 | 0 | None | downstream_gene_variant | LOW |  | None |  | None | 0.205882353 |
| chr16 | 49087596 | 49087597 | A | T | None |  |  | 0 | None | intergenic_variant | LOW |  | None |  | None | 0.117647059 |
| chr16 | 49349913 | 49349914 | C | A | None |  |  | 0 | None | intergenic_variant | LOW |  | None |  | None | 0.1 |
| chr16 | 49528275 | 49528276 | C | G | ZNF423 |  | ENST00000562520 | 0 | None | intron_variant | LOW |  | None |  | None | 0.267605634 |
| chr16 | 51428753 | 51428754 | G | T | None |  |  | 0 | None | intergenic_variant | LOW |  | None |  | None | 0.208333333 |
| chr16 | 52231457 | 52231458 | T | C | RP11-142G1.3 |  | ENST00000569998 | 0 | None | downstream_gene_variant | LOW |  | None |  | None | 0.288888889 |
| chr16 | 52687785 | 52687786 | C | A | CASC16 |  | ENST00000563844 | 0 | None | upstream_gene_variant | LOW |  | None |  | None | 0.320754717 |
| chr16 | 56029726 | 56029727 | G | A | None |  |  | 0 | None | intergenic_variant | LOW |  | None |  | None | 0.357142857 |
| chr16 | 58977072 | 58977073 | C | T | RP11-410D17.2 |  | ENST00000500117 | 1 | None | intron_variant | LOW |  | None |  | None | 0.211538462 |
| chr16 | 59039383 | 59039384 | G | A | RP11-410D17.2 |  | ENST00000500117 | 0 | None | intron_variant | LOW |  | None |  | None | 0.257142857 |
| chr16 | 59478523 | 59478524 | T | C | None |  |  | 0 | None | intergenic_variant | LOW |  | None |  | None | 0.139534884 |
| chr16 | 59769279 | 59769280 | C | T | RP11-105C20.2 |  | ENST00000564533 | 1 | None | downstream_gene_variant | LOW |  | None |  | None | 0.157894737 |
| chr16 | 59944620 | 59944621 | G | A | RP11-430C1.1 |  | ENST00000568279 | 0 | None | intron_variant | LOW |  | None |  | None | 0.260869565 |
| chr16 | 60117646 | 60117647 | G | A | None |  |  | 0 | None | intergenic_variant | LOW |  | None |  | None | 0.159090909 |
| chr16 | 60143544 | 60143545 | C | G | None |  |  | 0 | None | intergenic_variant | LOW |  | None |  | None | 0.421052632 |
| chr16 | 61044489 | 61044490 | T | A | None |  |  | 1 | None | intergenic_variant | LOW |  | None |  | None | 0.15 |
| chr16 | 61583027 | 61583028 | A | G | None |  |  | 0 | None | intergenic_variant | LOW |  | None |  | None | 0.3 |
| chr16 | 61789177 | 61789178 | T | A | CDH8 |  | ENST00000577730 | 0 | None | intron_variant | LOW |  | None |  | None | 0.230769231 |
| chr16 | 62091675 | 62091676 | C | T | None |  |  | 0 | None | intergenic_variant | LOW |  | None |  | None | 0.363636364 |
| chr16 | 62321393 | 62321394 | T | G | None |  |  | 0 | None | intergenic_variant | LOW |  | None |  | None | 0.333333333 |
| chr16 | 64351074 | 64351075 | C | A | AC012322.1 |  | ENST00000561657 | 0 | None | intron_variant | LOW |  | None |  | None | 0.342857143 |
| chr16 | 66144917 | 66144918 | G | A | None |  |  | 0 | None | intergenic_variant | LOW |  | None |  | None | 0.326923077 |
| chr16 | 66664682 | 66664683 | C | G | CMTM4 |  | ENST00000394106 | 0 | None | intron_variant | LOW |  | None |  | None | 0.119402985 |
| chr16 | 66801800 | 66801801 | T | A | CCDC79 |  | ENST00000433574 | 0 | None | intron_variant | LOW |  | None |  | None | 0.166666667 |
| chr16 | 66801802 | 66801803 | G | A | CCDC79 |  | ENST00000415744 | 0 | None | intron_variant | LOW |  | None |  | None | 0.166666667 |
| chr16 | 66801831 | 66801832 | T | G | CCDC79 |  | ENST00000415744 | 0 | None | intron_variant | LOW |  | None |  | None | 0.12 |
| chr16 | 66801835 | 66801836 | G | A | CCDC79 |  | ENST00000432602 | 0 | None | intron_variant | LOW |  | None |  | None | 0.115384615 |
| chr16 | 66954458 | 66954459 | T | C | CDH16 |  | ENST00000565235 | 0 | None | upstream_gene_variant | LOW |  | None |  | None | 0.132075472 |
| chr16 | 67410988 | 67410989 | A | G | LRRC36 |  | ENST00000290940 | 0 | None | intron_variant | LOW |  | None |  | None | 0.173076923 |
| chr16 | 67438902 | 67438903 | C | A | ZDHHC1 |  | ENST00000348579 | 0 | None | intron_variant | LOW |  | None |  | None | 0.285714286 |
| chr16 | 76371225 | 76371226 | G | A | CNTNAP4 |  | ENST00000478060 | 0 | None | intron_variant | LOW |  | None |  | None | 0.333333333 |
| chr16 | 76422287 | 76422288 | T | C | CNTNAP4 |  | ENST00000478060 | 0 | None | intron_variant | LOW |  | None |  | None | 0.276595745 |
| chr16 | 76528256 | 76528257 | A | T | CNTNAP4 |  | ENST00000307431 | 0 | None | intron_variant | LOW |  | None |  | None | 0.342857143 |
| chr16 | 76551787 | 76551788 | T | A | CNTNAP4 |  | ENST00000307431 | 1 | None | intron_variant | LOW |  | None |  | None | 0.285714286 |
| chr16 | 77117798 | 77117799 | T | A | None |  |  | 0 | None | intergenic_variant | LOW |  | None |  | None | 0.1875 |
| chr16 | 77877001 | 77877002 | C | A | VAT1L |  | ENST00000302536 | 0 | None | intron_variant | LOW |  | None |  | None | 0.272727273 |
| chr16 | 80763355 | 80763356 | G | T | CDYL2 |  | ENST00000570137 | 1 | None | intron_variant | LOW |  | None |  | None | 0.222222222 |
| chr16 | 81681420 | 81681421 | C | T | CMIP |  | ENST00000398040 | 0 | None | intron_variant | LOW |  | None |  | None | 0.267857143 |
| chr16 | 82590285 | 82590286 | G | T | RP11-2L4.1 |  | ENST00000565374 | 0 | None | intron_variant | LOW |  | None |  | None | 0.261904762 |
| chr16 | 82637103 | 82637104 | C | T | None |  |  | 0 | None | intergenic_variant | LOW |  | None |  | None | 0.25 |
| chr16 | 82731244 | 82731245 | G | A | CDH13 |  | ENST00000565636 | 0 | None | intron_variant | LOW |  | None |  | None | 0.263157895 |
| chr16 | 82856129 | 82856130 | C | A | CDH13 |  | ENST00000565636 | 0 | None | intron_variant | LOW |  | None |  | None | 0.210526316 |
| chr16 | 83015672 | 83015673 | G | T | CDH13 |  | ENST00000446376 | 0 | None | intron_variant | LOW |  | None |  | None | 0.235294118 |
| chr16 | 83437788 | 83437789 | T | A | CDH13 |  | ENST00000268613 | 0 | None | intron_variant | LOW |  | None |  | None | 0.245614035 |
| chr16 | 84966064 | 84966065 | C | T | RP11-254F19.3 |  | ENST00000569104 | 0 | None | intron_variant | LOW |  | None |  | None | 0.24 |
| chr16 | 85189625 | 85189626 | C | T | None |  |  | 0 | None | intergenic_variant | LOW |  | None |  | None | 0.306122449 |
| chr16 | 86314119 | 86314120 | A | G | Y_RNA |  | ENST00000363079 | 0 | None | upstream_gene_variant | LOW |  | None |  | None | 0.244897959 |
| chr16 | 86314123 | 86314124 | C | T | LINC01081 |  | ENST00000597373 | 0 | None | upstream_gene_variant | LOW |  | None |  | None | 0.244897959 |
| chr16 | 86314145 | 86314146 | C | G | LINC01081 |  | ENST00000597373 | 0 | None | upstream_gene_variant | LOW |  | None |  | None | 0.236363636 |
| chr16 | 87209817 | 87209818 | C | T | C16orf95 |  | ENST00000562840 | 0 | None | intron_variant | LOW |  | None |  | None | 0.285714286 |
| chr16 | 89521062 | 89521063 | C | T | ANKRD11 |  | ENST00000378330 | 0 | None | intron_variant | LOW |  | None |  | None | 0.314285714 |
| chr16 | 89752803 | 89752804 | A | C | RP11-368I7.4 |  | ENST00000567544 | 0 | None | intron_variant | LOW |  | None |  | None | 0.236842105 |
| chr17 | 84984 | 84985 | G | C | RPH3AL |  | ENST00000323434 | 1 | None | intron_variant | LOW |  | None |  | None | 0.166666667 |
| chr17 | 99544 | 99545 | G | C | RPH3AL |  | ENST00000331302 | 0 | None | intron_variant | LOW |  | None |  | None | 0.138888889 |
| chr17 | 217737 | 217738 | C | T | RPH3AL |  | ENST00000575130 | 0 | None | intron_variant | LOW |  | None |  | None | 0.18 |
| chr17 | 672602 | 672603 | A | G | GLOD4 |  | ENST00000573137 | 0 | None | intron_variant | LOW |  | None |  | None | 0.2 |
| chr17 | 687768 | 687769 | G | T | RNMTL1 |  | ENST00000304478 | 0 | None | intron_variant | LOW |  | None |  | None | 0.225 |
| chr17 | 764118 | 764119 | C | A | NXN |  | ENST00000537628 | 0 | None | intron_variant | LOW |  | None |  | None | 0.307692308 |
| chr17 | 1231868 | 1231869 | T | A | None |  |  | 1 | None | intergenic_variant | LOW |  | None |  | None | 0.22 |
| chr17 | 1231868 | 1231869 | T | C | None |  |  | 1 | None | intergenic_variant | LOW |  | None |  | None | 0.22 |
| chr17 | 1231885 | 1231886 | T | G | None |  |  | 0 | None | intergenic_variant | LOW |  | None |  | None | 0.16 |
| chr17 | 2234461 | 2234462 | T | C | TSR1 |  | ENST00000301364 | 0 | None | intron_variant | LOW |  | None |  | None | 0.388888889 |
| chr17 | 2424429 | 2424430 | C | T | None |  |  | 0 | None | intergenic_variant | LOW |  | None |  | None | 0.163265306 |
| chr17 | 2623124 | 2623125 | C | G | RP11-74E22.4 |  | ENST00000575506 | 1 | None | downstream_gene_variant | LOW |  | None |  | None | 0.196078431 |
| chr17 | 2623158 | 2623159 | C | T | RP11-74E22.4 |  | ENST00000575506 | 1 | None | downstream_gene_variant | LOW |  | None |  | None | 0.169491525 |
| chr17 | 3455739 | 3455740 | G | A | TRPV3 |  | ENST00000301365 | 0 | None | intron_variant | LOW |  | None |  | None | 0.113207547 |
| chr17 | 6931807 | 6931808 | G | C | BCL6B |  | ENST00000293805 | 1 | None | 3_prime_UTR_variant | LOW |  | None |  | None | NA |
| chr17 | 7083884 | 7083885 | A | T | ASGR1 |  | ENST00000572879 | 0 | None | upstream_gene_variant | LOW |  | None |  | None | 0.166666667 |
| chr17 | 8722114 | 8722115 | G | A | PIK3R6 |  | ENST00000311434 | 0 | None | intron_variant | LOW |  | None |  | None | 0.282608696 |
| chr17 | 8771497 | 8771498 | T | C | PIK3R6 |  | ENST00000311434 | 0 | None | upstream_gene_variant | LOW |  | None |  | None | 0.244444444 |
| chr17 | 9994486 | 9994487 | A | C | GAS7 |  | ENST00000540214 | 0 | None | intron_variant | LOW |  | None |  | None | 0.325 |
| chr17 | 10084449 | 10084450 | G | T | GAS7 |  | ENST00000432992 | 0 | None | intron_variant | LOW |  | None |  | None | 0.192307692 |
| chr17 | 12669458 | 12669459 | T | G | MYOCD |  | ENST00000425538 | 0 | None | 3_prime_UTR_variant | LOW |  | None |  | None | 0.219512195 |
| chr17 | 13326957 | 13326958 | A | T | None |  |  | 0 | None | intergenic_variant | LOW |  | None |  | None | 0.346938776 |
| chr17 | 13713861 | 13713862 | A | G | COX10-AS1 |  | ENST00000602743 | 0 | None | intron_variant | LOW |  | None |  | None | 0.317073171 |
| chr17 | 14051301 | 14051302 | C | T | COX10 |  | ENST00000537334 | 0 | None | intron_variant | LOW |  | None |  | None | 0.208333333 |
| chr17 | 15491512 | 15491513 | G | A | RP11-385D13.1 |  | ENST00000455584 | 1 | None | intron_variant | LOW |  | None |  | None | 0.163265306 |
| chr17 | 15902203 | 15902204 | A | G | ZSWIM7 |  | ENST00000399277 | 0 | None | intron_variant | LOW |  | None |  | None | 0.12244898 |
| chr17 | 16369003 | 16369004 | A | T | FAM211A |  | ENST00000470794 | 0 | None | intron_variant | LOW |  | None |  | None | 0.263157895 |
| chr17 | 16650809 | 16650810 | T | C | CCDC144A |  | ENST00000443444 | 1 | None | intron_variant | LOW |  | None |  | None | 0.166666667 |
| chr17 | 17251943 | 17251944 | G | A | NT5M |  | ENST00000389022 | 1 | None | downstream_gene_variant | LOW |  | None |  | None | 0.092105263 |
| chr17 | 19904943 | 19904944 | C | G | None |  |  | 0 | None | intergenic_variant | LOW |  | None |  | None | 0.153846154 |
| chr17 | 20773706 | 20773707 | T | G | CCDC144NL |  | ENST00000327925 | 0 | None | intron_variant | LOW |  | None |  | None | 0.358208955 |
| chr17 | 21198351 | 21198352 | G | T | MAP2K3 |  | ENST00000316920 | 0 | None | intron_variant | LOW |  | None |  | None | 0.20 |
| chr17 | 21198351 | 21198352 | G | C | MAP2K3 |  | ENST00000316920 | 1 | None | intron_variant | LOW |  | None |  | None | 0.23 |
| chr17 | 21318628 | 21318629 | C | A | KCNJ12 |  | ENST00000583088 | 1 | None | 5_prime_UTR_variant | LOW |  | None |  | None | 0.27 |
| chr17 | 21318628 | 21318629 | C | T | KCNJ12 |  | ENST00000583088 | 1 | None | 5_prime_UTR_variant | LOW |  | None |  | None | 0.33 |
| chr17 | 21784166 | 21784167 | T | C | RP11-1109M24.16 |  | ENST00000580507 | 0 | None | intron_variant | LOW |  | None |  | None | 0.255813953 |
| chr17 | 21909748 | 21909749 | T | A | RP11-744K17.9 |  | ENST00000581223 | 0 | None | non_coding_exon_variant | LOW |  | None |  | None | 0.218181818 |
| chr17 | 21981417 | 21981418 | C | A | None |  |  | 0 | None | intergenic_variant | LOW |  | None |  | None | 0.162790698 |
| chr17 | 22016432 | 22016433 | C | T | RP11-846F4.1 |  | ENST00000578634 | 0 | None | upstream_gene_variant | LOW |  | None |  | None | 0.26 |
| chr17 | 22108113 | 22108114 | C | A | RP11-846F4.10 |  | ENST00000584626 | 0 | None | downstream_gene_variant | LOW |  | None |  | None | 0.103448276 |
| chr17 | 22208817 | 22208818 | G | A | RP11-846F4.11 |  | ENST00000578745 | 0 | None | downstream_gene_variant | LOW |  | None |  | None | 0.093333333 |
| chr17 | 25289901 | 25289902 | A | G | None |  |  | 1 | None | intergenic_variant | LOW |  | None |  | None | 0.24 |
| chr17 | 25289901 | 25289902 | A | T | None |  |  | 1 | None | intergenic_variant | LOW |  | None |  | None | 0.47 |
| chr17 | 25312352 | 25312353 | C | T | RP11-260A9.6 |  | ENST00000580686 | 1 | None | downstream_gene_variant | LOW |  | None |  | None | 0.196078431 |
| chr17 | 25312371 | 25312372 | T | C | RP11-260A9.6 |  | ENST00000580686 | 1 | None | downstream_gene_variant | LOW |  | None |  | None | 0.169491525 |
| chr17 | 26557560 | 26557561 | G | A | PYY2 |  | ENST00000441253 | 0 | None | downstream_gene_variant | LOW |  | None |  | None | 0.151515152 |
| chr17 | 27079081 | 27079082 | T | A | TRAF4 |  | ENST00000473421 | 0 | None | downstream_gene_variant | LOW |  | None |  | None | 0.116666667 |
| chr17 | 27155112 | 27155113 | A | T | FAM222B |  | ENST00000583307 | 0 | None | intron_variant | LOW |  | None |  | None | 0.111111111 |
| chr17 | 27338116 | 27338117 | G | A | SEZ6 |  | ENST00000317338 | 0 | None | upstream_gene_variant | LOW |  | None |  | None | 0.306122449 |
| chr17 | 27414167 | 27414168 | C | G | MYO18A |  | ENST00000533112 | 0 | None | intron_variant | LOW |  | None |  | None | 0.155844156 |
| chr17 | 28013746 | 28013747 | C | T | SSH2 |  | ENST00000540801 | 0 | None | intron_variant | LOW |  | None |  | None | 0.137254902 |
| chr17 | 28942067 | 28942068 | A | C | AC005562.1 |  | ENST00000578265 | 0 | None | intron_variant | LOW |  | None |  | None | 0.220338983 |
| chr17 | 29069813 | 29069814 | T | A | SUZ12P |  | ENST00000497969 | 0 | None | intron_variant | LOW |  | None |  | None | 0.179104478 |
| chr17 | 30824380 | 30824381 | A | C | MYO1D |  | ENST00000394649 | 0 | None | intron_variant | LOW |  | None |  | None | 0.327272727 |
| chr17 | 31523077 | 31523078 | G | A | ASIC2 |  | ENST00000359872 | 0 | None | intron_variant | LOW |  | None |  | None | 0.114754098 |
| chr17 | 32296340 | 32296341 | G | A | ASIC2 |  | ENST00000359872 | 0 | None | intron_variant | LOW |  | None |  | None | 0.203703704 |
| chr17 | 32423324 | 32423325 | A | G | ASIC2 |  | ENST00000359872 | 0 | None | intron_variant | LOW |  | None |  | None | 0.220338983 |
| chr17 | 32575814 | 32575815 | C | T | None |  |  | 0 | None | intergenic_variant | LOW |  | None |  | None | 0.254237288 |
| chr17 | 33419759 | 33419760 | G | A | RAD51L3-RFFL |  | ENST00000593039 | 0 | None | intron_variant | LOW |  | None |  | None | 0.210526316 |
| chr17 | 35252398 | 35252399 | C | T | RP11-445F12.1 |  | ENST00000528383 | 0 | None | intron_variant | LOW |  | None |  | None | 0.255813953 |
| chr17 | 35949978 | 35949979 | C | T | SYNRG |  | ENST00000345615 | 0 | None | intron_variant | LOW |  | None |  | None | 0.118644068 |
| chr17 | 36544857 | 36544858 | C | T | SOCS7 |  | ENST00000577233 | 0 | None | intron_variant | LOW |  | None |  | None | 0.2 |
| chr17 | 37860891 | 37860892 | G | T | ERBB2 |  | ENST00000540147 | 0 | None | intron_variant | LOW |  | None |  | None | 0.268656716 |
| chr17 | 38128076 | 38128077 | T | C | GSDMA |  | ENST00000301659 | 0 | None | intron_variant | LOW |  | None |  | None | 0.202898551 |
| chr17 | 38417286 | 38417287 | T | A | WIPF2 |  | ENST00000536600 | 1 | None | intron_variant | LOW |  | None |  | None | 0.13559322 |
| chr17 | 38546948 | 38546949 | G | A | TOP2A |  | ENST00000423485 | 0 | None | intron_variant | LOW |  | None |  | None | 0.25 |
| chr17 | 38835137 | 38835138 | C | T | None |  |  | 0 | None | intergenic_variant | LOW |  | None |  | None | 0.224489796 |
| chr17 | 40035937 | 40035938 | A | C | ACLY |  | ENST00000353196 | 0 | None | intron_variant | LOW |  | None |  | None | 0.266666667 |
| chr17 | 40321366 | 40321367 | G | A | KCNH4 |  | ENST00000607371 | 0 | None | intron_variant | LOW |  | None |  | None | 0.192982456 |
| chr17 | 40322706 | 40322707 | G | C | KCNH4 |  | ENST00000264661 | 0 | None | intron_variant | LOW |  | None |  | None | 0.212121212 |
| chr17 | 41566680 | 41566681 | T | A | DHX8 |  | ENST00000605777 | 0 | None | intron_variant | LOW |  | None |  | None | 0.25 |
| chr17 | 42414563 | 42414564 | C | T | None |  |  | 0 | None | intergenic_variant | LOW |  | None |  | None | 0.271186441 |
| chr17 | 42612258 | 42612259 | C | T | None |  |  | 0 | None | intergenic_variant | LOW |  | None |  | None | 0.267857143 |
| chr17 | 44310624 | 44310625 | G | A | None |  |  | 0 | None | intergenic_variant | LOW |  | None |  | None | 0.189655172 |
| chr17 | 45380287 | 45380288 | A | C | ITGB3 |  | ENST00000435993 | 0 | None | intron_variant | LOW |  | None |  | None | 0.181818182 |
| chr17 | 46082060 | 46082061 | G | T | None |  |  | 0 | None | intergenic_variant | LOW |  | None |  | None | 0.171428571 |
| chr17 | 46297970 | 46297971 | C | T | SKAP1 |  | ENST00000336915 | 0 | None | intron_variant | LOW |  | None |  | None | 0.125 |
| chr17 | 46826152 | 46826153 | A | G | None |  |  | 0 | None | intergenic_variant | LOW |  | None |  | None | 0.203125 |
| chr17 | 47227470 | 47227471 | A | G | B4GALNT2 |  | ENST00000393354 | 1 | None | intron_variant | LOW |  | None |  | None | 0.2 |
| chr17 | 47434912 | 47434913 | A | T | ZNF652 |  | ENST00000362063 | 0 | None | intron_variant | LOW |  | None |  | None | 0.205882353 |
| chr17 | 47683012 | 47683013 | C | T | SPOP |  | ENST00000504102 | 0 | None | intron_variant | LOW |  | None |  | None | 0.159090909 |
| chr17 | 48059505 | 48059506 | C | T | None |  |  | 0 | None | intergenic_variant | LOW |  | None |  | None | 0.233333333 |
| chr17 | 49059102 | 49059103 | A | T | SPAG9 |  | ENST00000505279 | 0 | None | intron_variant | LOW |  | None |  | None | 0.136363636 |
| chr17 | 50394694 | 50394695 | T | A | None |  |  | 0 | None | intergenic_variant | LOW |  | None |  | None | 0.3 |
| chr17 | 50931207 | 50931208 | T | A | None |  |  | 0 | None | intergenic_variant | LOW |  | None |  | None | 0.166666667 |
| chr17 | 51041800 | 51041801 | A | C | None |  |  | 1 | None | intergenic_variant | LOW |  | None |  | None | 0.152173913 |
| chr17 | 51130336 | 51130337 | A | T | None |  |  | 0 | None | intergenic_variant | LOW |  | None |  | None | 0.241935484 |
| chr17 | 51183023 | 51183024 | G | A | RP11-750B16.1 |  | ENST00000574371 | 0 | None | upstream_gene_variant | LOW |  | None |  | None | 0.147540984 |
| chr17 | 51183045 | 51183046 | C | A | RP11-750B16.1 |  | ENST00000574371 | 0 | None | upstream_gene_variant | LOW |  | None |  | None | 0.134328358 |
| chr17 | 51183046 | 51183047 | A | C | RP11-750B16.1 |  | ENST00000574371 | 0 | None | upstream_gene_variant | LOW |  | None |  | None | 0.138461538 |
| chr17 | 51194433 | 51194434 | T | C | None |  |  | 0 | None | intergenic_variant | LOW |  | None |  | None | 0.162790698 |
| chr17 | 52082436 | 52082437 | T | C | None |  |  | 0 | None | intergenic_variant | LOW |  | None |  | None | 0.188679245 |
| chr17 | 52367876 | 52367877 | G | T | None |  |  | 0 | None | intergenic_variant | LOW |  | None |  | None | 0.209677419 |
| chr17 | 54700486 | 54700487 | A | T | None |  |  | 0 | None | intergenic_variant | LOW |  | None |  | None | 0.146341463 |
| chr17 | 55037895 | 55037896 | A | T | COIL |  | ENST00000240316 | 0 | None | intron_variant | LOW |  | None |  | None | 0.125 |
| chr17 | 56154178 | 56154179 | C | A | RP11-159D12.10 |  | ENST00000584805 | 0 | None | downstream_gene_variant | LOW |  | None |  | None | 0.206349206 |
| chr17 | 56382602 | 56382603 | C | A | BZRAP1 |  | ENST00000268893 | 0 | None | intron_variant | LOW |  | None |  | None | 0.107142857 |
| chr17 | 56523864 | 56523865 | T | A | HSF5 |  | ENST00000323777 | 0 | None | intron_variant | LOW |  | None |  | None | 0.172413793 |
| chr17 | 56645213 | 56645214 | C | A | TEX14 |  | ENST00000240361 | 1 | None | intron_variant | LOW |  | None |  | None | 0.228070175 |
| chr17 | 56866038 | 56866039 | T | C | PPM1E |  | ENST00000308249 | 0 | None | intron_variant | LOW |  | None |  | None | 0.253521127 |
| chr17 | 57219719 | 57219720 | C | T | SKA2 |  | ENST00000581068 | 0 | None | intron_variant | LOW |  | None |  | None | 0.191489362 |
| chr17 | 60152160 | 60152161 | T | A | None |  |  | 0 | None | intergenic_variant | LOW |  | None |  | None | 0.185185185 |
| chr17 | 62971867 | 62971868 | C | A | AMZ2P1 |  | ENST00000430983 | 0 | None | upstream_gene_variant | LOW |  | None |  | None | 0.220338983 |
| chr17 | 63498370 | 63498371 | A | T | None |  |  | 0 | None | intergenic_variant | LOW |  | None |  | None | 0.129032258 |
| chr17 | 64167972 | 64167973 | A | T | CEP112 |  | ENST00000537949 | 0 | None | intron_variant | LOW |  | None |  | None | 0.2 |
| chr17 | 64190755 | 64190756 | C | T | CEP112 |  | ENST00000537949 | 0 | None | upstream_gene_variant | LOW |  | None |  | None | 0.304347826 |
| chr17 | 64638792 | 64638793 | A | G | PRKCA |  | ENST00000413366 | 0 | None | intron_variant | LOW |  | None |  | None | 0.111111111 |
| chr17 | 64732112 | 64732113 | A | G | PRKCA |  | ENST00000413366 | 0 | None | intron_variant | LOW |  | None |  | None | 0.2 |
| chr17 | 65262448 | 65262449 | A | T | RNA5SP447 |  | ENST00000363749 | 0 | None | upstream_gene_variant | LOW |  | None |  | None | 0.131147541 |
| chr17 | 66056709 | 66056710 | C | A | None |  |  | 0 | None | intergenic_variant | LOW |  | None |  | None | 0.133333333 |
| chr17 | 66147507 | 66147508 | T | C | LRRC37A16P |  | ENST00000585915 | 0 | None | upstream_gene_variant | LOW |  | None |  | None | 0.115384615 |
| chr17 | 66398865 | 66398866 | C | G | ARSG |  | ENST00000448504 | 0 | None | intron_variant | LOW |  | None |  | None | 0.184615385 |
| chr17 | 66708302 | 66708303 | G | T | RP11-118B18.1 |  | ENST00000589610 | 0 | None | intron_variant | LOW |  | None |  | None | 0.208333333 |
| chr17 | 66824628 | 66824629 | C | T | None |  |  | 0 | None | intergenic_variant | LOW |  | None |  | None | 0.166666667 |
| chr17 | 67561420 | 67561421 | G | A | None |  |  | 1 | None | intergenic_variant | LOW |  | None |  | None | 0.193548387 |
| chr17 | 67632320 | 67632321 | G | T | AC003051.1 |  | ENST00000591334 | 0 | None | intron_variant | LOW |  | None |  | None | 0.12962963 |
| chr17 | 69007836 | 69007837 | C | T | RP11-1003J3.1 |  | ENST00000604782 | 0 | None | upstream_gene_variant | LOW |  | None |  | None | 0.245614035 |
| chr17 | 69354421 | 69354422 | G | A | None |  |  | 0 | None | intergenic_variant | LOW |  | None |  | None | 0.090909091 |
| chr17 | 70489258 | 70489259 | T | C | LINC00511 |  | ENST00000581801 | 0 | None | intron_variant | LOW |  | None |  | None | 0.208333333 |
| chr17 | 70528130 | 70528131 | A | G | LINC00511 |  | ENST00000457958 | 0 | None | intron_variant | LOW |  | None |  | None | 0.2 |
| chr17 | 70921706 | 70921707 | A | G | SLC39A11 |  | ENST00000583146 | 0 | None | intron_variant | LOW |  | None |  | None | 0.236363636 |
| chr17 | 71223126 | 71223127 | G | A | FAM104A |  | ENST00000579872 | 0 | None | intron_variant | LOW |  | None |  | None | 0.13559322 |
| chr17 | 71293512 | 71293513 | T | G | CDC42EP4 |  | ENST00000581014 | 0 | None | intron_variant | LOW |  | None |  | None | 0.22 |
| chr17 | 72655111 | 72655112 | G | C | None |  |  | 0 | None | intergenic_variant | LOW |  | None |  | None | 0.220338983 |
| chr17 | 73167210 | 73167211 | T | A | SUMO2 |  | ENST00000420826 | 0 | None | intron_variant | LOW |  | None |  | None | 0.211538462 |
| chr17 | 74523278 | 74523279 | G | C | CYGB |  | ENST00000589342 | 0 | None | downstream_gene_variant | LOW |  | None |  | None | 0.153846154 |
| chr17 | 76477383 | 76477384 | A | G | DNAH17 |  | ENST00000585328 | 0 | None | intron_variant | LOW |  | None |  | None | 0.169491525 |
| chr17 | 77016764 | 77016765 | G | A | C1QTNF1-AS1 |  | ENST00000581579 | 0 | None | intron_variant | LOW |  | None |  | None | 0.265625 |
| chr17 | 77399021 | 77399022 | C | T | RBFOX3 |  | ENST00000584778 | 1 | None | intron_variant | LOW |  | None |  | None | 0.216216216 |
| chr17 | 78552623 | 78552624 | G | C | RPTOR |  | ENST00000544334 | 0 | None | intron_variant | LOW |  | None |  | None | 0.166666667 |
| chr17 | 78615596 | 78615597 | C | T | RPTOR |  | ENST00000306801 | 1 | None | intron_variant | LOW |  | None |  | None | 0.241935484 |
| chr17 | 79148199 | 79148200 | A | G | AATK-AS1 |  | ENST00000414089 | 0 | None | intron_variant | LOW |  | None |  | None | 0.12345679 |
| chr17 | 79967645 | 79967646 | T | C | ASPSCR1 |  | ENST00000306739 | 1 | None | intron_variant | LOW |  | None |  | None | 0.173913043 |
| chr17 | 80096524 | 80096525 | A | G | CCDC57 |  | ENST00000389641 | 1 | None | intron_variant | LOW |  | None |  | None | 0.128205128 |
| chr17 | 80581380 | 80581381 | G | C | WDR45B |  | ENST00000392325 | 0 | None | intron_variant | LOW |  | None |  | None | 0.175438596 |
| chr17 | 80618256 | 80618257 | A | C | RAB40B |  | ENST00000571995 | 0 | None | intron_variant | LOW |  | None |  | None | 0.084210526 |
| chr17 | 80969774 | 80969775 | G | C | B3GNTL1 |  | ENST00000320865 | 0 | None | intron_variant | LOW |  | None |  | None | 0.226415094 |
| chr17 | 80969781 | 80969782 | G | A | B3GNTL1 |  | ENST00000576599 | 0 | None | intron_variant | LOW |  | None |  | None | 0.142857143 |
| chr17 | 80989191 | 80989192 | A | G | B3GNTL1 |  | ENST00000320865 | 1 | None | intron_variant | LOW |  | None |  | None | 0.229166667 |
| chr17 | 81165524 | 81165525 | C | T | AC139099.6 |  | ENST00000572850 | 0 | None | intron_variant | LOW |  | None |  | None | 0.2 |
| chr18 | 12280 | 12281 | C | T | AP005530.1 |  | ENST00000572573 | 0 | None | intron_variant | LOW |  | None |  | None | 0.134502924 |
| chr18 | 1145112 | 1145113 | C | T | RP11-78F17.1 |  | ENST00000581556 | 0 | None | intron_variant | LOW |  | None |  | None | 0.127659574 |
| chr18 | 1155594 | 1155595 | C | T | RP11-78F17.1 |  | ENST00000577719 | 0 | None | intron_variant | LOW |  | None |  | None | 0.195652174 |
| chr18 | 2184301 | 2184302 | G | C | RP11-161I6.2 |  | ENST00000579097 | 0 | None | intron_variant | LOW |  | None |  | None | 0.117647059 |
| chr18 | 2341579 | 2341580 | A | G | None |  |  | 0 | None | intergenic_variant | LOW |  | None |  | None | 0.148148148 |
| chr18 | 2353459 | 2353460 | G | T | None |  |  | 0 | None | intergenic_variant | LOW |  | None |  | None | 0.219512195 |
| chr18 | 4489546 | 4489547 | G | C | None |  |  | 0 | None | intergenic_variant | LOW |  | None |  | None | 0.266666667 |
| chr18 | 4671821 | 4671822 | T | A | None |  |  | 0 | None | intergenic_variant | LOW |  | None |  | None | 0.212765957 |
| chr18 | 4706755 | 4706756 | A | G | None |  |  | 0 | None | intergenic_variant | LOW |  | None |  | None | 0.2 |
| chr18 | 4765426 | 4765427 | T | A | None |  |  | 0 | None | intergenic_variant | LOW |  | None |  | None | 0.140625 |
| chr18 | 5272194 | 5272195 | C | A | None |  |  | 0 | None | intergenic_variant | LOW |  | None |  | None | 0.156862745 |
| chr18 | 5476590 | 5476591 | C | T | EPB41L3 |  | ENST00000584670 | 0 | None | intron_variant | LOW |  | None |  | None | 0.22 |
| chr18 | 6062045 | 6062046 | T | C | L3MBTL4 |  | ENST00000535782 | 0 | None | intron_variant | LOW |  | None |  | None | 0.225 |
| chr18 | 7015975 | 7015976 | C | T | LAMA1 |  | ENST00000389658 | 1 | None | intron_variant | LOW |  | None |  | None | 0.181818182 |
| chr18 | 7394923 | 7394924 | A | T | None |  |  | 0 | None | intergenic_variant | LOW |  | None |  | None | 0.151515152 |
| chr18 | 8455951 | 8455952 | G | C | None |  |  | 0 | None | intergenic_variant | LOW |  | None |  | None | 0.202702703 |
| chr18 | 8874596 | 8874597 | C | A | None |  |  | 0 | None | intergenic_variant | LOW |  | None |  | None | 0.192307692 |
| chr18 | 10530105 | 10530106 | T | A | NAPG |  | ENST00000322897 | 0 | None | intron_variant | LOW |  | None |  | None | 0.14516129 |
| chr18 | 10614738 | 10614739 | G | A | RP11-856M7.6 |  | ENST00000584734 | 1 | None | intron_variant | LOW |  | None |  | None | 0.225 |
| chr18 | 10625391 | 10625392 | C | A | RP11-856M7.6 |  | ENST00000584734 | 0 | None | intron_variant | LOW |  | None |  | None | 0.208333333 |
| chr18 | 11668680 | 11668681 | C | T | RP11-677O4.2 |  | ENST00000561598 | 0 | None | non_coding_exon_variant | LOW |  | None |  | None | 0.145833333 |
| chr18 | 13527684 | 13527685 | A | T | LDLRAD4 |  | ENST00000361205 | 0 | None | intron_variant | LOW |  | None |  | None | 0.184615385 |
| chr18 | 14322432 | 14322433 | C | T | None |  |  | 0 | None | intergenic_variant | LOW |  | None |  | None | 0.096774194 |
| chr18 | 14404217 | 14404218 | C | T | None |  |  | 0 | None | intergenic_variant | LOW |  | None |  | None | 0.303571429 |
| chr18 | 15179339 | 15179340 | A | T | None |  |  | 0 | None | intergenic_variant | LOW |  | None |  | None | 0.072580645 |
| chr18 | 15191985 | 15191986 | C | A | None |  |  | 1 | None | intergenic_variant | LOW |  | None |  | None | 0.15 |
| chr18 | 15191995 | 15191996 | T | G | None |  |  | 0 | None | intergenic_variant | LOW |  | None |  | None | 0.131147541 |
| chr18 | 15192134 | 15192135 | C | G | RP11-454P7.3 |  | ENST00000581666 | 1 | None | downstream_gene_variant | LOW |  | None |  | None | 0.12195122 |
| chr18 | 15196144 | 15196145 | G | A | RP11-454P7.3 |  | ENST00000581666 | 1 | None | downstream_gene_variant | LOW |  | None |  | None | 0.077777778 |
| chr18 | 15245814 | 15245815 | T | A | BNIP3P3 |  | ENST00000577336 | 0 | None | downstream_gene_variant | LOW |  | None |  | None | 0.14893617 |
| chr18 | 15315600 | 15315601 | C | T | AP005901.1 |  | ENST00000504516 | 1 | None | downstream_gene_variant | LOW |  | None |  | None | 0.137254902 |
| chr18 | 15344555 | 15344556 | A | G | None |  |  | 1 | None | intergenic_variant | LOW |  | None |  | None | 0.16 |
| chr18 | 15344655 | 15344656 | A | G | None |  |  | 1 | None | intergenic_variant | LOW |  | None |  | None | 0.186440678 |
| chr18 | 15383275 | 15383276 | A | C | None |  |  | 0 | None | intergenic_variant | LOW |  | None |  | None | 0.125 |
| chr18 | 15402631 | 15402632 | G | A | None |  |  | 0 | None | intergenic_variant | LOW |  | None |  | None | 0.083333333 |
| chr18 | 15408853 | 15408854 | A | T | None |  |  | 0 | None | intergenic_variant | LOW |  | None |  | None | 0.111111111 |
| chr18 | 18901030 | 18901031 | G | A | GREB1L |  | ENST00000269218 | 0 | None | intron_variant | LOW |  | None |  | None | 0.14 |
| chr18 | 19812306 | 19812307 | C | A | RP11-627G18.4 |  | ENST00000578741 | 1 | None | downstream_gene_variant | LOW |  | None |  | None | 0.177570093 |
| chr18 | 21025148 | 21025149 | T | G | None |  |  | 0 | None | intergenic_variant | LOW |  | None |  | None | 0.205882353 |
| chr18 | 21890024 | 21890025 | G | A | OSBPL1A |  | ENST00000357041 | 1 | None | intron_variant | LOW |  | None |  | None | 0.12244898 |
| chr18 | 23112902 | 23112903 | A | C | None |  |  | 0 | None | intergenic_variant | LOW |  | None |  | None | 0.215686275 |
| chr18 | 25486201 | 25486202 | G | A | None |  |  | 0 | None | intergenic_variant | LOW |  | None |  | None | 0.264705882 |
| chr18 | 25498969 | 25498970 | G | A | None |  |  | 0 | None | intergenic_variant | LOW |  | None |  | None | 0.347826087 |
| chr18 | 25564698 | 25564699 | C | G | CDH2 |  | ENST00000399380 | 0 | None | intron_variant | LOW |  | None |  | None | 0.339622642 |
| chr18 | 26197286 | 26197287 | T | A | None |  |  | 0 | None | intergenic_variant | LOW |  | None |  | None | 0.2 |
| chr18 | 26582812 | 26582813 | C | T | None |  |  | 0 | None | intergenic_variant | LOW |  | None |  | None | 0.133333333 |
| chr18 | 26645582 | 26645583 | C | A | None |  |  | 0 | None | intergenic_variant | LOW |  | None |  | None | 0.181818182 |
| chr18 | 26659365 | 26659366 | C | T | None |  |  | 0 | None | intergenic_variant | LOW |  | None |  | None | 0.203389831 |
| chr18 | 26879768 | 26879769 | G | C | CTD-2515C13.2 |  | ENST00000577674 | 0 | None | intron_variant | LOW |  | None |  | None | 0.169491525 |
| chr18 | 27134411 | 27134412 | C | T | None |  |  | 0 | None | intergenic_variant | LOW |  | None |  | None | 0.12244898 |
| chr18 | 27305298 | 27305299 | G | A | None |  |  | 0 | None | intergenic_variant | LOW |  | None |  | None | 0.196428571 |
| chr18 | 27395306 | 27395307 | G | A | None |  |  | 0 | None | intergenic_variant | LOW |  | None |  | None | 0.262295082 |
| chr18 | 27519785 | 27519786 | G | A | None |  |  | 0 | None | intergenic_variant | LOW |  | None |  | None | 0.326086957 |
| chr18 | 27717648 | 27717649 | A | G | None |  |  | 0 | None | intergenic_variant | LOW |  | None |  | None | 0.11627907 |
| chr18 | 27931735 | 27931736 | T | G | None |  |  | 0 | None | intergenic_variant | LOW |  | None |  | None | 0.127659574 |
| chr18 | 28137032 | 28137033 | A | G | None |  |  | 0 | None | intergenic_variant | LOW |  | None |  | None | 0.223880597 |
| chr18 | 28146238 | 28146239 | T | G | None |  |  | 0 | None | intergenic_variant | LOW |  | None |  | None | 0.170212766 |
| chr18 | 28269634 | 28269635 | T | C | None |  |  | 0 | None | intergenic_variant | LOW |  | None |  | None | 0.279069767 |
| chr18 | 28496948 | 28496949 | G | A | None |  |  | 0 | None | intergenic_variant | LOW |  | None |  | None | 0.276595745 |
| chr18 | 28505405 | 28505406 | C | G | None |  |  | 0 | None | intergenic_variant | LOW |  | None |  | None | 0.28 |
| chr18 | 29987944 | 29987945 | T | A | GAREM |  | ENST00000399218 | 1 | None | intron_variant | LOW |  | None |  | None | 0.210526316 |
| chr18 | 31157477 | 31157478 | T | G | ASXL3 |  | ENST00000586596 | 0 | None | upstream_gene_variant | LOW |  | None |  | None | 0.338983051 |
| chr18 | 31440503 | 31440504 | C | T | NOL4 |  | ENST00000269185 | 0 | None | intron_variant | LOW |  | None |  | None | 0.224137931 |
| chr18 | 32192348 | 32192349 | G | A | DTNA |  | ENST00000588684 | 0 | None | intron_variant | LOW |  | None |  | None | 0.163636364 |
| chr18 | 33223567 | 33223568 | C | A | GALNT1 |  | ENST00000591924 | 0 | None | intron_variant | LOW |  | None |  | None | 0.25 |
| chr18 | 34056721 | 34056722 | A | G | FHOD3 |  | ENST00000445677 | 0 | None | intron_variant | LOW |  | None |  | None | 0.240740741 |
| chr18 | 34488993 | 34488994 | A | G | KIAA1328 |  | ENST00000591619 | 0 | None | intron_variant | LOW |  | None |  | None | 0.259259259 |
| chr18 | 34784799 | 34784800 | G | C | KIAA1328 |  | ENST00000280020 | 0 | None | intron_variant | LOW |  | None |  | None | 0.181818182 |
| chr18 | 34853982 | 34853983 | G | T | CELF4 |  | ENST00000420428 | 0 | None | intron_variant | LOW |  | None |  | None | 0.103448276 |
| chr18 | 35267471 | 35267472 | G | T | None |  |  | 0 | None | intergenic_variant | LOW |  | None |  | None | 0.163636364 |
| chr18 | 35360362 | 35360363 | G | A | None |  |  | 1 | None | intergenic_variant | LOW |  | None |  | None | 0.1875 |
| chr18 | 35574851 | 35574852 | A | G | None |  |  | 0 | None | intergenic_variant | LOW |  | None |  | None | 0.156862745 |
| chr18 | 35653373 | 35653374 | G | A | None |  |  | 0 | None | intergenic_variant | LOW |  | None |  | None | 0.214285714 |
| chr18 | 35674704 | 35674705 | G | A | None |  |  | 0 | None | intergenic_variant | LOW |  | None |  | None | 0.12195122 |
| chr18 | 35720187 | 35720188 | G | A | None |  |  | 0 | None | intergenic_variant | LOW |  | None |  | None | 0.166666667 |
| chr18 | 35767778 | 35767779 | C | T | None |  |  | 1 | None | intergenic_variant | LOW |  | None |  | None | 0.163636364 |
| chr18 | 36524585 | 36524586 | C | G | None |  |  | 0 | None | intergenic_variant | LOW |  | None |  | None | 0.226415094 |
| chr18 | 36939970 | 36939971 | G | T | LINC00669 |  | ENST00000591629 | 0 | None | intron_variant | LOW |  | None |  | None | 0.209677419 |
| chr18 | 36940432 | 36940433 | G | A | LINC00669 |  | ENST00000591629 | 0 | None | intron_variant | LOW |  | None |  | None | 0.179104478 |
| chr18 | 37507151 | 37507152 | T | A | RP11-636O21.1 |  | ENST00000585822 | 0 | None | upstream_gene_variant | LOW |  | None |  | None | 0.163636364 |
| chr18 | 38035792 | 38035793 | T | C | None |  |  | 0 | None | intergenic_variant | LOW |  | None |  | None | 0.196428571 |
| chr18 | 38449616 | 38449617 | C | T | None |  |  | 0 | None | intergenic_variant | LOW |  | None |  | None | 0.245614035 |
| chr18 | 39471638 | 39471639 | T | A | None |  |  | 0 | None | intergenic_variant | LOW |  | None |  | None | 0.233333333 |
| chr18 | 39628178 | 39628179 | A | C | PIK3C3 |  | ENST00000597477 | 0 | None | intron_variant | LOW |  | None |  | None | 0.208333333 |
| chr18 | 40021734 | 40021735 | T | A | LINC00907 |  | ENST00000589068 | 1 | None | intron_variant | LOW |  | None |  | None | 0.125 |
| chr18 | 42176434 | 42176435 | C | G | None |  |  | 0 | None | intergenic_variant | LOW |  | None |  | None | 0.311111111 |
| chr18 | 42821359 | 42821360 | C | A | SLC14A2 |  | ENST00000586448 | 1 | None | intron_variant | LOW |  | None |  | None | 0.142857143 |
| chr18 | 43369574 | 43369575 | T | A | RP11-618K16.4 |  | ENST00000587507 | 0 | None | downstream_gene_variant | LOW |  | None |  | None | 0.208333333 |
| chr18 | 44549335 | 44549336 | C | T | TCEB3CL | P | ENST00000451265 | 1 | None | synonymous_variant | LOW |  | None |  | None | 0.058295964 |
| chr18 | 44893865 | 44893866 | C | G | CTD-2130O13.1 |  | ENST00000586905 | 0 | None | intron_variant | LOW |  | None |  | None | 0.163265306 |
| chr18 | 45333484 | 45333485 | T | G | None |  |  | 0 | None | intergenic_variant | LOW |  | None |  | None | 0.233333333 |
| chr18 | 46450854 | 46450855 | C | A | SMAD7 |  | ENST00000589634 | 0 | None | intron_variant | LOW |  | None |  | None | 0.179487179 |
| chr18 | 47349682 | 47349683 | C | T | MYO5B |  | ENST00000285039 | 1 | None | 3_prime_UTR_variant | LOW |  | None |  | None | 0.137931034 |
| chr18 | 48668863 | 48668864 | C | T | None |  |  | 0 | None | intergenic_variant | LOW |  | None |  | None | 0.134328358 |
| chr18 | 48882629 | 48882630 | C | T | RP11-267C16.1 |  | ENST00000582689 | 0 | None | intron_variant | LOW |  | None |  | None | 0.327586207 |
| chr18 | 48958779 | 48958780 | G | A | RP11-267C16.1 |  | ENST00000580841 | 0 | None | intron_variant | LOW |  | None |  | None | 0.227272727 |
| chr18 | 49231497 | 49231498 | G | A | None |  |  | 0 | None | intergenic_variant | LOW |  | None |  | None | 0.361702128 |
| chr18 | 49652383 | 49652384 | C | T | None |  |  | 0 | None | intergenic_variant | LOW |  | None |  | None | 0.255319149 |
| chr18 | 49779014 | 49779015 | G | A | None |  |  | 0 | None | intergenic_variant | LOW |  | None |  | None | 0.104166667 |
| chr18 | 50084055 | 50084056 | A | G | DCC |  | ENST00000442544 | 0 | None | intron_variant | LOW |  | None |  | None | 0.240740741 |
| chr18 | 50085711 | 50085712 | A | G | DCC |  | ENST00000442544 | 0 | None | intron_variant | LOW |  | None |  | None | 0.325581395 |
| chr18 | 50986619 | 50986620 | G | A | DCC |  | ENST00000442544 | 0 | None | intron_variant | LOW |  | None |  | None | 0.189189189 |
| chr18 | 53205258 | 53205259 | A | G | TCF4 |  | ENST00000563824 | 1 | None | intron_variant | LOW |  | None |  | None | 0.159090909 |
| chr18 | 53270262 | 53270263 | C | T | TCF4 |  | ENST00000569357 | 0 | None | intron_variant | LOW |  | None |  | None | 0.134615385 |
| chr18 | 53821541 | 53821542 | C | G | CTD-2008L17.2 |  | ENST00000382897 | 0 | None | intron_variant | LOW |  | None |  | None | 0.175438596 |
| chr18 | 53821555 | 53821556 | A | C | CTD-2008L17.2 |  | ENST00000382897 | 0 | None | intron_variant | LOW |  | None |  | None | 0.163636364 |
| chr18 | 54382212 | 54382213 | T | A | WDR7 |  | ENST00000357574 | 0 | None | intron_variant | LOW |  | None |  | None | 0.12244898 |
| chr18 | 54693980 | 54693981 | C | T | WDR7 |  | ENST00000254442 | 0 | None | intron_variant | LOW |  | None |  | None | 0.316666667 |
| chr18 | 55050239 | 55050240 | A | T | None |  |  | 0 | None | intergenic_variant | LOW |  | None |  | None | 0.227272727 |
| chr18 | 55568222 | 55568223 | G | A | None |  |  | 0 | None | intergenic_variant | LOW |  | None |  | None | 0.236363636 |
| chr18 | 56757543 | 56757544 | A | T | None |  |  | 0 | None | intergenic_variant | LOW |  | None |  | None | 0.227272727 |
| chr18 | 57395065 | 57395066 | T | C | None |  |  | 0 | None | intergenic_variant | LOW |  | None |  | None | 0.345454545 |
| chr18 | 57798584 | 57798585 | G | A | RP11-795H16.3 |  | ENST00000588794 | 0 | None | intron_variant | LOW |  | None |  | None | 0.127272727 |
| chr18 | 58471576 | 58471577 | G | A | RP11-325K19.1 |  | ENST00000591869 | 0 | None | splice_region_variant | LOW |  | None |  | None | 0.292682927 |
| chr18 | 59948689 | 59948690 | C | T | KIAA1468 |  | ENST00000398130 | 0 | None | intron_variant | LOW |  | None |  | None | 0.265625 |
| chr18 | 59974910 | 59974911 | A | G | KIAA1468 |  | ENST00000256858 | 0 | None | downstream_gene_variant | LOW |  | None |  | None | 0.194029851 |
| chr18 | 61112589 | 61112590 | G | T | None |  |  | 0 | None | intergenic_variant | LOW |  | None |  | None | 0.267857143 |
| chr18 | 62038707 | 62038708 | T | A | RP11-146N18.1 |  | ENST00000589376 | 0 | None | intron_variant | LOW |  | None |  | None | 0.185185185 |
| chr18 | 63063726 | 63063727 | G | A | None |  |  | 0 | None | intergenic_variant | LOW |  | None |  | None | 0.149253731 |
| chr18 | 63131826 | 63131827 | G | C | None |  |  | 0 | None | intergenic_variant | LOW |  | None |  | None | 0.215686275 |
| chr18 | 63244192 | 63244193 | G | A | None |  |  | 0 | None | intergenic_variant | LOW |  | None |  | None | 0.177777778 |
| chr18 | 63882705 | 63882706 | T | C | None |  |  | 0 | None | intergenic_variant | LOW |  | None |  | None | 0.2 |
| chr18 | 64459344 | 64459345 | C | A | None |  |  | 0 | None | intergenic_variant | LOW |  | None |  | None | 0.163265306 |
| chr18 | 64711909 | 64711910 | C | T | AC113195.1 |  | ENST00000583185 | 0 | None | downstream_gene_variant | LOW |  | None |  | None | 0.235294118 |
| chr18 | 64867669 | 64867670 | A | C | None |  |  | 0 | None | intergenic_variant | LOW |  | None |  | None | 0.13559322 |
| chr18 | 65412799 | 65412800 | G | T | RP11-638L3.1 |  | ENST00000583687 | 0 | None | intron_variant | LOW |  | None |  | None | 0.176470588 |
| chr18 | 65716554 | 65716555 | T | C | None |  |  | 0 | None | intergenic_variant | LOW |  | None |  | None | 0.208333333 |
| chr18 | 65850975 | 65850976 | C | T | None |  |  | 1 | None | intergenic_variant | LOW |  | None |  | None | 0.140350877 |
| chr18 | 65981858 | 65981859 | G | A | None |  |  | 0 | None | intergenic_variant | LOW |  | None |  | None | 0.225806452 |
| chr18 | 66084010 | 66084011 | C | T | None |  |  | 0 | None | intergenic_variant | LOW |  | None |  | None | 0.222222222 |
| chr18 | 66112343 | 66112344 | G | C | None |  |  | 0 | None | intergenic_variant | LOW |  | None |  | None | 0.152173913 |
| chr18 | 66382610 | 66382611 | G | A | CCDC102B |  | ENST00000584775 | 0 | None | intron_variant | LOW |  | None |  | None | 0.206896552 |
| chr18 | 66596895 | 66596896 | C | T | CCDC102B |  | ENST00000319445 | 0 | None | intron_variant | LOW |  | None |  | None | 0.2 |
| chr18 | 67006493 | 67006494 | T | C | None |  |  | 0 | None | intergenic_variant | LOW |  | None |  | None | 0.210526316 |
| chr18 | 67091291 | 67091292 | A | T | DOK6 |  | ENST00000382713 | 0 | None | intron_variant | LOW |  | None |  | None | 0.267605634 |
| chr18 | 67508981 | 67508982 | C | T | DOK6 |  | ENST00000382713 | 0 | None | 3_prime_UTR_variant | LOW |  | None |  | None | 0.264150943 |
| chr18 | 67587799 | 67587800 | C | T | CD226 |  | ENST00000582621 | 0 | None | intron_variant | LOW |  | None |  | None | 0.147058824 |
| chr18 | 68009416 | 68009417 | C | T | RP11-41O4.1 |  | ENST00000584919 | 1 | None | intron_variant | LOW |  | None |  | None | 0.101694915 |
| chr18 | 68009428 | 68009429 | C | T | RP11-41O4.1 |  | ENST00000584919 | 0 | None | intron_variant | LOW |  | None |  | None | 0.127272727 |
| chr18 | 68260086 | 68260087 | A | G | None |  |  | 0 | None | intergenic_variant | LOW |  | None |  | None | 0.244897959 |
| chr18 | 68397689 | 68397690 | T | C | None |  |  | 0 | None | intergenic_variant | LOW |  | None |  | None | 0.220338983 |
| chr18 | 68430156 | 68430157 | C | T | None |  |  | 1 | None | intergenic_variant | LOW |  | None |  | None | 0.195652174 |
| chr18 | 68444595 | 68444596 | A | G | None |  |  | 1 | None | intergenic_variant | LOW |  | None |  | None | 0.12962963 |
| chr18 | 68878296 | 68878297 | T | C | None |  |  | 0 | None | intergenic_variant | LOW |  | None |  | None | 0.215686275 |
| chr18 | 69009358 | 69009359 | C | T | None |  |  | 0 | None | intergenic_variant | LOW |  | None |  | None | 0.159090909 |
| chr18 | 69045398 | 69045399 | C | T | None |  |  | 0 | None | intergenic_variant | LOW |  | None |  | None | 0.179487179 |
| chr18 | 69098468 | 69098469 | G | T | None |  |  | 0 | None | intergenic_variant | LOW |  | None |  | None | 0.228070175 |
| chr18 | 69293173 | 69293174 | T | C | None |  |  | 0 | None | intergenic_variant | LOW |  | None |  | None | 0.267857143 |
| chr18 | 69878622 | 69878623 | G | A | None |  |  | 0 | None | intergenic_variant | LOW |  | None |  | None | 0.2 |
| chr18 | 69898568 | 69898569 | G | T | None |  |  | 0 | None | intergenic_variant | LOW |  | None |  | None | 0.181818182 |
| chr18 | 70651676 | 70651677 | G | A | None |  |  | 0 | None | intergenic_variant | LOW |  | None |  | None | 0.185185185 |
| chr18 | 71795614 | 71795615 | C | A | FBXO15 |  | ENST00000269500 | 0 | None | intron_variant | LOW |  | None |  | None | 0.206349206 |
| chr18 | 72756296 | 72756297 | T | A | ZNF407 |  | ENST00000299687 | 0 | None | intron_variant | LOW |  | None |  | None | 0.265306122 |
| chr18 | 72938414 | 72938415 | G | T | TSHZ1 |  | ENST00000580243 | 0 | None | intron_variant | LOW |  | None |  | None | 0.118421053 |
| chr18 | 73228015 | 73228016 | C | A | None |  |  | 0 | None | intergenic_variant | LOW |  | None |  | None | 0.15625 |
| chr18 | 73457405 | 73457406 | T | A | None |  |  | 0 | None | intergenic_variant | LOW |  | None |  | None | 0.196721311 |
| chr18 | 73520386 | 73520387 | C | T | None |  |  | 0 | None | intergenic_variant | LOW |  | None |  | None | 0.245283019 |
| chr18 | 73598973 | 73598974 | A | T | None |  |  | 0 | None | intergenic_variant | LOW |  | None |  | None | 0.274509804 |
| chr18 | 73611771 | 73611772 | G | T | None |  |  | 0 | None | intergenic_variant | LOW |  | None |  | None | 0.236842105 |
| chr18 | 73635061 | 73635062 | G | T | None |  |  | 0 | None | intergenic_variant | LOW |  | None |  | None | 0.166666667 |
| chr18 | 74163790 | 74163791 | G | A | ZNF516 |  | ENST00000532857 | 0 | None | intron_variant | LOW |  | None |  | None | 0.196721311 |
| chr18 | 74647330 | 74647331 | T | A | ZNF236 |  | ENST00000320610 | 0 | None | intron_variant | LOW |  | None |  | None | 0.232142857 |
| chr18 | 74930886 | 74930887 | A | C | RP11-751H17.1 |  | ENST00000584843 | 0 | None | intron_variant | LOW |  | None |  | None | 0.245901639 |
| chr18 | 75006898 | 75006899 | G | A | None |  |  | 0 | None | intergenic_variant | LOW |  | None |  | None | 0.282608696 |
| chr18 | 75452479 | 75452480 | C | A | None |  |  | 0 | None | intergenic_variant | LOW |  | None |  | None | 0.161290323 |
| chr18 | 75498698 | 75498699 | G | C | None |  |  | 0 | None | intergenic_variant | LOW |  | None |  | None | 0.220588235 |
| chr18 | 75569763 | 75569764 | G | T | None |  |  | 0 | None | intergenic_variant | LOW |  | None |  | None | 0.175438596 |
| chr18 | 75583229 | 75583230 | A | T | None |  |  | 0 | None | intergenic_variant | LOW |  | None |  | None | 0.259259259 |
| chr18 | 75786301 | 75786302 | C | T | None |  |  | 0 | None | intergenic_variant | LOW |  | None |  | None | 0.189655172 |
| chr18 | 75884605 | 75884606 | C | T | None |  |  | 0 | None | intergenic_variant | LOW |  | None |  | None | 0.157142857 |
| chr18 | 75936138 | 75936139 | C | T | None |  |  | 0 | None | intergenic_variant | LOW |  | None |  | None | 0.224137931 |
| chr18 | 75938506 | 75938507 | C | A | None |  |  | 0 | None | intergenic_variant | LOW |  | None |  | None | 0.183333333 |
| chr18 | 76235651 | 76235652 | C | T | RP11-451L19.1 |  | ENST00000579769 | 0 | None | upstream_gene_variant | LOW |  | None |  | None | 0.094117647 |
| chr18 | 76268668 | 76268669 | C | T | RP11-123I22.1 |  | ENST00000581634 | 1 | None | downstream_gene_variant | LOW |  | None |  | None | 0.171428571 |
| chr18 | 76774747 | 76774748 | G | C | None |  |  | 1 | None | intergenic_variant | LOW |  | None |  | None | 0.084745763 |
| chr18 | 76774832 | 76774833 | A | G | None |  |  | 1 | None | intergenic_variant | LOW |  | None |  | None | 0.083333333 |
| chr18 | 77087658 | 77087659 | T | G | ATP9B |  | ENST00000589732 | 0 | None | intron_variant | LOW |  | None |  | None | 0.214285714 |
| chr18 | 77243484 | 77243485 | G | A | NFATC1 |  | ENST00000542384 | 0 | None | intron_variant | LOW |  | None |  | None | 0.135135135 |
| chr18 | 77306674 | 77306675 | G | A | None |  |  | 0 | None | intergenic_variant | LOW |  | None |  | None | 0.125 |
| chr18 | 77607852 | 77607853 | A | C | None |  |  | 0 | None | intergenic_variant | LOW |  | None |  | None | 0.258064516 |
| chr18 | 77654281 | 77654282 | C | G | KCNG2 |  | ENST00000316249 | 1 | None | intron_variant | LOW |  | None |  | None | 0.098360656 |
| chr19 | 81832 | 81833 | C | T | FAM138F |  | ENST00000448235 | 0 | None | upstream_gene_variant | LOW |  | None |  | None | 0.288461538 |
| chr19 | 247015 | 247016 | G | C | None |  |  | 0 | None | intergenic_variant | LOW |  | None |  | None | 0.151515152 |
| chr19 | 429269 | 429270 | C | A | SHC2 |  | ENST00000264554 | 0 | None | intron_variant | LOW |  | None |  | None | 0.229166667 |
| chr19 | 650088 | 650089 | T | C | RNF126 |  | ENST00000592418 | 0 | None | intron_variant | LOW |  | None |  | None | 0.282051282 |
| chr19 | 949463 | 949464 | G | A | ARID3A |  | ENST00000587532 | 0 | None | intron_variant | LOW |  | None |  | None | 0.1875 |
| chr19 | 1330878 | 1330879 | T | C | MUM1 |  | ENST00000344663 | 0 | None | intron_variant | LOW |  | None |  | None | 0.178082192 |
| chr19 | 1330904 | 1330905 | G | C | MUM1 |  | ENST00000344663 | 0 | None | intron_variant | LOW |  | None |  | None | 0.13559322 |
| chr19 | 1956267 | 1956268 | T | G | CSNK1G2 |  | ENST00000589350 | 0 | None | intron_variant | LOW |  | None |  | None | 0.208955224 |
| chr19 | 2773992 | 2773993 | A | G | SGTA |  | ENST00000589251 | 1 | None | intron_variant | LOW |  | None |  | None | 0.196078431 |
| chr19 | 2918024 | 2918025 | T | C | ZNF57 | M/T | ENST00000306908 | 0 | None | missense_variant | MED | benign | 0.171 | tolerated | 1 | 0.150684932 |
| chr19 | 3137880 | 3137881 | C | A | GNA15 |  | ENST00000262958 | 0 | None | intron_variant | LOW |  | None |  | None | 0.136363636 |
| chr19 | 3762972 | 3762973 | C | T | MRPL54 |  | ENST00000330133 | 0 | None | intron_variant | LOW |  | None |  | None | 0.102941176 |
| chr19 | 3790643 | 3790644 | C | T | MATK |  | ENST00000590849 | 0 | None | intron_variant | LOW |  | None |  | None | 0.216666667 |
| chr19 | 4056208 | 4056209 | G | A | ZBTB7A |  | ENST00000601588 | 0 | None | intron_variant | LOW |  | None |  | None | 0.229508197 |
| chr19 | 4131171 | 4131172 | T | C | None |  |  | 0 | None | intergenic_variant | LOW |  | None |  | None | 0.261538462 |
| chr19 | 4478200 | 4478201 | T | G | HDGFRP2 |  | ENST00000586684 | 1 | None | intron_variant | LOW |  | None |  | None | 0.259259259 |
| chr19 | 5315822 | 5315823 | G | T | PTPRS |  | ENST00000357368 | 0 | None | intron_variant | LOW |  | None |  | None | 0.132075472 |
| chr19 | 5820092 | 5820093 | T | C | NRTN |  | ENST00000303212 | 1 | None | upstream_gene_variant | LOW |  | None |  | None | 0.181818182 |
| chr19 | 5820126 | 5820127 | T | A | NRTN |  | ENST00000303212 | 0 | None | upstream_gene_variant | LOW |  | None |  | None | 0.153846154 |
| chr19 | 6639066 | 6639067 | T | C | None |  |  | 0 | None | intergenic_variant | LOW |  | None |  | None | 0.125 |
| chr19 | 8506770 | 8506771 | A | C | HNRNPM |  | ENST00000600092 | 0 | None | upstream_gene_variant | LOW |  | None |  | None | 0.206896552 |
| chr19 | 8952429 | 8952430 | G | A | MBD3L1 |  | ENST00000595891 | 0 | None | intron_variant | LOW |  | None |  | None | 0.271186441 |
| chr19 | 9209527 | 9209528 | G | A | OR7G2 |  | ENST00000305456 | 0 | None | downstream_gene_variant | LOW |  | None |  | None | 0.277777778 |
| chr19 | 9539180 | 9539181 | A | G | ZNF266 |  | ENST00000590306 | 0 | None | intron_variant | LOW |  | None |  | None | 0.280701754 |
| chr19 | 10641657 | 10641658 | G | C | None |  |  | 0 | None | intergenic_variant | LOW |  | None |  | None | 0.129032258 |
| chr19 | 10737480 | 10737481 | A | G | SLC44A2 |  | ENST00000335757 | 0 | None | intron_variant | LOW |  | None |  | None | 0.351351351 |
| chr19 | 11210233 | 11210234 | T | G | LDLR |  | ENST00000558013 | 0 | None | intron_variant | LOW |  | None |  | None | 0.117647059 |
| chr19 | 11210243 | 11210244 | A | C | LDLR |  | ENST00000557933 | 0 | None | intron_variant | LOW |  | None |  | None | 0.114285714 |
| chr19 | 11408037 | 11408038 | C | A | TSPAN16 |  | ENST00000590327 | 0 | None | intron_variant | LOW |  | None |  | None | 0.16 |
| chr19 | 12026255 | 12026256 | T | C | ZNF69 |  | ENST00000340180 | 1 | None | downstream_gene_variant | LOW |  | None |  | None | 0.150684932 |
| chr19 | 13291705 | 13291706 | C | T | None |  |  | 0 | None | intergenic_variant | LOW |  | None |  | None | 0.222222222 |
| chr19 | 13291706 | 13291707 | A | C | None |  |  | 0 | None | intergenic_variant | LOW |  | None |  | None | 0.238095238 |
| chr19 | 13651839 | 13651840 | A | T | CACNA1A |  | ENST00000592864 | 1 | None | intron_variant | LOW |  | None |  | None | 0.175438596 |
| chr19 | 13918567 | 13918568 | C | G | ZSWIM4 |  | ENST00000440752 | 0 | None | intron_variant | LOW |  | None |  | None | 0.24 |
| chr19 | 14908631 | 14908632 | T | C | OR7C1 |  | ENST00000248073 | 0 | None | downstream_gene_variant | LOW |  | None |  | None | 0.260869565 |
| chr19 | 15220836 | 15220837 | C | T | SYDE1 | Y | ENST00000600440 | 0 | None | synonymous_variant | LOW |  | None |  | None | 0.242424242 |
| chr19 | 15320760 | 15320761 | A | C | None |  |  | 0 | None | intergenic_variant | LOW |  | None |  | None | 0.288135593 |
| chr19 | 15475590 | 15475591 | G | A | AKAP8 |  | ENST00000269701 | 0 | None | intron_variant | LOW |  | None |  | None | 0.194444444 |
| chr19 | 15496234 | 15496235 | A | G | AKAP8L |  | ENST00000397410 | 0 | None | intron_variant | LOW |  | None |  | None | 0.285714286 |
| chr19 | 15496263 | 15496264 | T | C | AKAP8L |  | ENST00000595465 | 0 | None | intron_variant | LOW |  | None |  | None | 0.261904762 |
| chr19 | 15989695 | 15989696 | G | C | CYP4F2 | A/G | ENST00000221700 | 1 | COSM225400 | missense_variant | MED | benign | 0.127 | deleterious | 0.02 | 0.111111111 |
| chr19 | 16456540 | 16456541 | G | A | None |  |  | 0 | None | intergenic_variant | LOW |  | None |  | None | 0.292307692 |
| chr19 | 16785239 | 16785240 | C | A | TMEM38A |  | ENST00000187762 | 0 | None | intron_variant | LOW |  | None |  | None | 0.169491525 |
| chr19 | 17415817 | 17415818 | C | T | MRPL34 |  | ENST00000595444 | 0 | None | intron_variant | LOW |  | None |  | None | 0.254237288 |
| chr19 | 17572275 | 17572276 | A | T | CTD-2521M24.10 |  | ENST00000594663 | 0 | None | upstream_gene_variant | LOW |  | None |  | None | 0.119402985 |
| chr19 | 18040398 | 18040399 | A | C | CCDC124 |  | ENST00000445755 | 0 | None | upstream_gene_variant | LOW |  | None |  | None | 0.222222222 |
| chr19 | 18077291 | 18077292 | C | A | KCNN1 |  | ENST00000222249 | 0 | None | intron_variant | LOW |  | None |  | None | 0.253521127 |
| chr19 | 19130656 | 19130657 | G | A | SUGP2 |  | ENST00000600377 | 1 | None | intron_variant | LOW |  | None |  | None | 0.133333333 |
| chr19 | 19833313 | 19833314 | G | A | ZNF14 |  | ENST00000344099 | 0 | None | intron_variant | LOW |  | None |  | None | 0.117647059 |
| chr19 | 19896280 | 19896281 | A | G | ZNF506 |  | ENST00000587461 | 0 | None | downstream_gene_variant | LOW |  | None |  | None | 0.140350877 |
| chr19 | 19896308 | 19896309 | T | C | ZNF506 |  | ENST00000587461 | 0 | None | downstream_gene_variant | LOW |  | None |  | None | 0.150943396 |
| chr19 | 19896309 | 19896310 | G | A | ZNF506 |  | ENST00000587461 | 0 | None | downstream_gene_variant | LOW |  | None |  | None | 0.132075472 |
| chr19 | 19896323 | 19896324 | C | T | ZNF506 |  | ENST00000587461 | 0 | None | downstream_gene_variant | LOW |  | None |  | None | 0.117647059 |
| chr19 | 20288955 | 20288956 | A | C | ZNF486 |  | ENST00000597083 | 0 | None | intron_variant | LOW |  | None |  | None | 0.142857143 |
| chr19 | 20630671 | 20630672 | G | T | CTC-513N18.6 |  | ENST00000598131 | 0 | None | intron_variant | LOW |  | None |  | None | 0.26984127 |
| chr19 | 21734413 | 21734414 | C | G | ZNF429 |  | ENST00000594385 | 0 | None | intron_variant | LOW |  | None |  | None | 0.163934426 |
| chr19 | 22914146 | 22914147 | A | T | None |  |  | 0 | None | intergenic_variant | LOW |  | None |  | None | 0.193548387 |
| chr19 | 23001457 | 23001458 | C | T | None |  |  | 0 | None | intergenic_variant | LOW |  | None |  | None | 0.188679245 |
| chr19 | 23179705 | 23179706 | C | T | ZNF728 |  | ENST00000594710 | 0 | None | intron_variant | LOW |  | None |  | None | 0.235294118 |
| chr19 | 23355200 | 23355201 | G | A | None |  |  | 0 | None | intergenic_variant | LOW |  | None |  | None | 0.340425532 |
| chr19 | 23462458 | 23462459 | G | T | None |  |  | 0 | None | intergenic_variant | LOW |  | None |  | None | 0.145454545 |
| chr19 | 23476066 | 23476067 | T | C | VN1R90P |  | ENST00000595717 | 0 | None | downstream_gene_variant | LOW |  | None |  | None | 0.169811321 |
| chr19 | 23740631 | 23740632 | C | A | ZNF675 |  | ENST00000600313 | 0 | None | intron_variant | LOW |  | None |  | None | 0.296296296 |
| chr19 | 24284842 | 24284843 | G | A | ZNF254 |  | ENST00000342944 | 1 | None | intron_variant | LOW |  | None |  | None | 0.222222222 |
| chr19 | 24505510 | 24505511 | C | T | None |  |  | 0 | None | intergenic_variant | LOW |  | None |  | None | 0.153846154 |
| chr19 | 24550198 | 24550199 | G | A | None |  |  | 0 | None | intergenic_variant | LOW |  | None |  | None | 0.105882353 |
| chr19 | 24560813 | 24560814 | C | G | None |  |  | 0 | None | intergenic_variant | LOW |  | None |  | None | 0.1 |
| chr19 | 24579929 | 24579930 | C | A | None |  |  | 0 | None | intergenic_variant | LOW |  | None |  | None | 0.112359551 |
| chr19 | 27739467 | 27739468 | A | C | None |  |  | 1 | None | intergenic_variant | LOW |  | None |  | None | 0.208 |
| chr19 | 27778882 | 27778883 | G | C | None |  |  | 1 | None | intergenic_variant | LOW |  | None |  | None | 0.066666667 |
| chr19 | 27778909 | 27778910 | C | G | None |  |  | 0 | None | intergenic_variant | LOW |  | None |  | None | 0.152173913 |
| chr19 | 27848698 | 27848699 | G | A | None |  |  | 0 | None | intergenic_variant | LOW |  | None |  | None | 0.14 |
| chr19 | 27848698 | 27848699 | G | T | None |  |  | 1 | None | intergenic_variant | LOW |  | None |  | None | 0.32 |
| chr19 | 27912108 | 27912109 | C | G | None |  |  | 1 | None | intergenic_variant | LOW |  | None |  | None | 0.01 |
| chr19 | 27912108 | 27912109 | C | T | None |  |  | 0 | None | intergenic_variant | LOW |  | None |  | None | 0.08 |
| chr19 | 28392977 | 28392978 | G | A | LLNLF-65H9.1 |  | ENST00000592806 | 0 | None | intron_variant | LOW |  | None |  | None | 0.240740741 |
| chr19 | 29115465 | 29115466 | C | T | AC005394.1 |  | ENST00000585394 | 0 | None | intron_variant | LOW |  | None |  | None | 0.365384615 |
| chr19 | 29227963 | 29227964 | A | C | None |  |  | 0 | None | intergenic_variant | LOW |  | None |  | None | 0.133333333 |
| chr19 | 29571193 | 29571194 | G | A | None |  |  | 0 | None | intergenic_variant | LOW |  | None |  | None | 0.14 |
| chr19 | 29920645 | 29920646 | C | T | CTC-525D6.1 |  | ENST00000577849 | 0 | None | intron_variant | LOW |  | None |  | None | 0.333333333 |
| chr19 | 29933177 | 29933178 | G | A | CTC-525D6.1 |  | ENST00000582581 | 0 | None | intron_variant | LOW |  | None |  | None | 0.183673469 |
| chr19 | 30266639 | 30266640 | A | T | None |  |  | 0 | None | intergenic_variant | LOW |  | None |  | None | 0.1 |
| chr19 | 30660165 | 30660166 | G | A | None |  |  | 0 | None | intergenic_variant | LOW |  | None |  | None | 0.301369863 |
| chr19 | 31168215 | 31168216 | T | C | ZNF536 |  | ENST00000592773 | 0 | None | intron_variant | LOW |  | None |  | None | 0.174603175 |
| chr19 | 31794316 | 31794317 | G | A | TSHZ3 |  | ENST00000240587 | 0 | None | intron_variant | LOW |  | None |  | None | 0.225352113 |
| chr19 | 32769680 | 32769681 | C | T | None |  |  | 0 | None | intergenic_variant | LOW |  | None |  | None | 0.210526316 |
| chr19 | 33039283 | 33039284 | G | C | None |  |  | 0 | None | intergenic_variant | LOW |  | None |  | None | 0.28 |
| chr19 | 34107051 | 34107052 | A | G | None |  |  | 0 | None | intergenic_variant | LOW |  | None |  | None | 0.185185185 |
| chr19 | 34608850 | 34608851 | G | A | None |  |  | 0 | None | intergenic_variant | LOW |  | None |  | None | 0.176470588 |
| chr19 | 34694808 | 34694809 | T | A | LSM14A |  | ENST00000433627 | 0 | None | intron_variant | LOW |  | None |  | None | 0.127272727 |
| chr19 | 34918931 | 34918932 | A | C | UBA2 |  | ENST00000590048 | 0 | None | upstream_gene_variant | LOW |  | None |  | None | 0.256410256 |
| chr19 | 34921925 | 34921926 | T | C | UBA2 |  | ENST00000590048 | 0 | None | intron_variant | LOW |  | None |  | None | 0.35 |
| chr19 | 35191712 | 35191713 | C | G | None |  |  | 1 | None | intergenic_variant | LOW |  | None |  | None | 0.114285714 |
| chr19 | 35239207 | 35239208 | C | T | None |  |  | 0 | None | intergenic_variant | LOW |  | None |  | None | 0.269230769 |
| chr19 | 35498219 | 35498220 | G | T | GRAMD1A |  | ENST00000411896 | 0 | None | intron_variant | LOW |  | None |  | None | 0.123076923 |
| chr19 | 35708935 | 35708936 | C | T | None |  |  | 1 | None | intergenic_variant | LOW |  | None |  | None | 0.189189189 |
| chr19 | 36700362 | 36700363 | C | T | ZNF565 |  | ENST00000304116 | 0 | None | intron_variant | LOW |  | None |  | None | 0.185185185 |
| chr19 | 37618642 | 37618643 | T | C | ZNF420 | P | ENST00000304239 | 0 | None | synonymous_variant | LOW |  | None |  | None | 0.160714286 |
| chr19 | 38265018 | 38265019 | G | A | ZNF573 |  | ENST00000392138 | 0 | None | intron_variant | LOW |  | None |  | None | 0.192982456 |
| chr19 | 38461775 | 38461776 | G | T | SIPA1L3 |  | ENST00000222345 | 0 | None | intron_variant | LOW |  | None |  | None | 0.327868852 |
| chr19 | 39825558 | 39825559 | G | A | GMFG |  | ENST00000595636 | 0 | None | intron_variant | LOW |  | None |  | None | 0.246376812 |
| chr19 | 40855676 | 40855677 | T | C | PLD3 |  | ENST00000599685 | 0 | None | intron_variant | LOW |  | None |  | None | 0.189655172 |
| chr19 | 41573903 | 41573904 | A | C | None |  |  | 0 | None | intergenic_variant | LOW |  | None |  | None | 0.166666667 |
| chr19 | 41608819 | 41608820 | G | A | None |  |  | 1 | None | intergenic_variant | LOW |  | None |  | None | 0.086206897 |
| chr19 | 43319447 | 43319448 | A | G | PSG8 |  | ENST00000401467 | 0 | None | intron_variant | LOW |  | None |  | None | 0.22 |
| chr19 | 43436837 | 43436838 | G | T | PSG7 |  | ENST00000471557 | 0 | None | downstream_gene_variant | LOW |  | None |  | None | 0.20754717 |
| chr19 | 43556819 | 43556820 | A | G | None |  |  | 0 | None | intergenic_variant | LOW |  | None |  | None | 0.27027027 |
| chr19 | 43629998 | 43629999 | A | T | None |  |  | 0 | None | intergenic_variant | LOW |  | None |  | None | 0.195652174 |
| chr19 | 43694796 | 43694797 | T | C | PSG5 |  | ENST00000342951 | 0 | None | upstream_gene_variant | LOW |  | None |  | None | 0.218181818 |
| chr19 | 44002884 | 44002885 | C | T | PHLDB3 |  | ENST00000292140 | 0 | None | intron_variant | LOW |  | None |  | None | 0.237288136 |
| chr19 | 44100743 | 44100744 | A | G | ZNF576 |  | ENST00000533118 | 0 | None | splice_region_variant | LOW |  | None |  | None | 0.205882353 |
| chr19 | 44718057 | 44718058 | G | C | ZNF227 |  | ENST00000589707 | 0 | None | intron_variant | LOW |  | None |  | None | 0.263157895 |
| chr19 | 44914981 | 44914982 | T | C | CTC-512J12.4 |  | ENST00000588655 | 0 | None | intron_variant | LOW |  | None |  | None | 0.095588235 |
| chr19 | 44961440 | 44961441 | A | C | None |  |  | 0 | None | intergenic_variant | LOW |  | None |  | None | 0.064327485 |
| chr19 | 45049443 | 45049444 | G | A | CEACAM22P |  | ENST00000446628 | 1 | None | intron_variant | LOW |  | None |  | None | 0.125 |
| chr19 | 46312510 | 46312511 | G | A | RSPH6A |  | ENST00000600188 | 0 | None | intron_variant | LOW |  | None |  | None | 0.116666667 |
| chr19 | 48088093 | 48088094 | T | C | RN7SL322P |  | ENST00000583519 | 0 | None | downstream_gene_variant | LOW |  | None |  | None | 0.114754098 |
| chr19 | 48088099 | 48088100 | T | C | RN7SL322P |  | ENST00000583519 | 0 | None | downstream_gene_variant | LOW |  | None |  | None | 0.15 |
| chr19 | 48311975 | 48311976 | A | G | TPRX1 |  | ENST00000535759 | 1 | None | intron_variant | LOW |  | None |  | None | 0.090909091 |
| chr19 | 48865059 | 48865060 | A | T | TMEM143 |  | ENST00000601522 | 0 | None | intron_variant | LOW |  | None |  | None | 0.1 |
| chr19 | 49671118 | 49671119 | G | A | TRPM4 |  | ENST00000355712 | 0 | None | intron_variant | LOW |  | None |  | None | 0.103448276 |
| chr19 | 49742005 | 49742006 | G | A | None |  |  | 0 | None | intergenic_variant | LOW |  | None |  | None | 0.203125 |
| chr19 | 51280519 | 51280520 | A | T | CTD-2568A17.1 |  | ENST00000563228 | 1 | None | intron_variant | LOW |  | None |  | None | 0.285714286 |
| chr19 | 51849009 | 51849010 | A | G | ETFB |  | ENST00000309244 | 1 | None | intron_variant | LOW |  | None |  | None | 0.222222222 |
| chr19 | 52289249 | 52289250 | T | A | FPR1 |  | ENST00000594900 | 0 | None | intron_variant | LOW |  | None |  | None | 0.126760563 |
| chr19 | 52493369 | 52493370 | T | A | ZNF350 |  | ENST00000594929 | 0 | None | upstream_gene_variant | LOW |  | None |  | None | 0.203389831 |
| chr19 | 53049306 | 53049307 | G | C | ZNF808 |  | ENST00000486474 | 0 | None | intron_variant | LOW |  | None |  | None | 0.084210526 |
| chr19 | 54058646 | 54058647 | A | G | ZNF331 |  | ENST00000504493 | 1 | None | 5_prime_UTR_variant | LOW |  | None |  | None | 0.20754717 |
| chr19 | 54219510 | 54219511 | G | T | MIR517B |  | ENST00000385102 | 0 | None | upstream_gene_variant | LOW |  | None |  | None | 0.090909091 |
| chr19 | 54258945 | 54258946 | G | A | MIR1283-2 |  | ENST00000408621 | 0 | None | upstream_gene_variant | LOW |  | None |  | None | 0.125 |
| chr19 | 54298106 | 54298107 | G | A | NLRP12 |  | ENST00000345770 | 0 | None | intron_variant | LOW |  | None |  | None | 0.166666667 |
| chr19 | 54316552 | 54316553 | C | T | NLRP12 |  | ENST00000354278 | 0 | None | intron_variant | LOW |  | None |  | None | 0.20754717 |
| chr19 | 55078304 | 55078305 | G | T | None |  |  | 0 | None | intergenic_variant | LOW |  | None |  | None | 0.166666667 |
| chr19 | 55642244 | 55642245 | C | T | TNNT1 |  | ENST00000588981 | 1 | None | downstream_gene_variant | LOW |  | None |  | None | 0.14516129 |
| chr19 | 55736192 | 55736193 | A | G | AC010327.2 |  | ENST00000598855 | 0 | None | upstream_gene_variant | LOW |  | None |  | None | 0.138461538 |
| chr19 | 55941937 | 55941938 | A | G | SHISA7 |  | ENST00000376325 | 0 | None | 3_prime_UTR_variant | LOW |  | None |  | None | 0.188679245 |
| chr19 | 56301794 | 56301795 | G | A | NLRP11 |  | ENST00000360133 | 0 | None | intron_variant | LOW |  | None |  | None | 0.246153846 |
| chr19 | 56356948 | 56356949 | A | T | NLRP4 |  | ENST00000301295 | 0 | None | intron_variant | LOW |  | None |  | None | 0.183098592 |
| chr19 | 56617465 | 56617466 | T | C | ZNF787 |  | ENST00000270459 | 0 | None | intron_variant | LOW |  | None |  | None | 0.163934426 |
| chr19 | 56677195 | 56677196 | A | C | ZNF444 |  | ENST00000337080 | 1 | None | downstream_gene_variant | LOW |  | None |  | None | 0.26 |
| chr19 | 56914379 | 56914380 | C | T | ZNF583 |  | ENST00000333201 | 0 | None | upstream_gene_variant | LOW |  | None |  | None | 0.178571429 |
| chr19 | 56961935 | 56961936 | T | C | ZNF667 |  | ENST00000587555 | 0 | None | intron_variant | LOW |  | None |  | None | 0.193548387 |
| chr19 | 57182527 | 57182528 | G | T | ZNF835 |  | ENST00000601659 | 0 | None | intron_variant | LOW |  | None |  | None | 0.138461538 |
| chr19 | 57182552 | 57182553 | G | A | ZNF835 |  | ENST00000537055 | 0 | None | intron_variant | LOW |  | None |  | None | 0.120689655 |
| chr19 | 57347810 | 57347811 | G | A | PEG3 |  | ENST00000599577 | 0 | None | intron_variant | LOW |  | None |  | None | 0.241935484 |
| chr19 | 57504730 | 57504731 | C | T | None |  |  | 0 | None | intergenic_variant | LOW |  | None |  | None | 0.245901639 |
| chr19 | 57532140 | 57532141 | A | G | None |  |  | 0 | None | intergenic_variant | LOW |  | None |  | None | 0.2 |
| chr19 | 57681798 | 57681799 | A | T | DUXA |  | ENST00000554048 | 1 | None | upstream_gene_variant | LOW |  | None |  | None | 0.153846154 |
| chr19 | 58011169 | 58011170 | C | T | ZNF773 |  | ENST00000598770 | 0 | None | upstream_gene_variant | LOW |  | None |  | None | 0.294117647 |
| chr19 | 59116753 | 59116754 | C | T | None |  |  | 0 | None | intergenic_variant | LOW |  | None |  | None | 0.086956522 |
| chr2 | 18736 | 18737 | C | T | None |  |  | 0 | None | intergenic_variant | LOW |  | None |  | None | 0.155844156 |
| chr2 | 288102 | 288103 | G | A | FAM150B |  | ENST00000403610 | 0 | None | 5_prime_UTR_variant | LOW |  | None |  | None | 0.204081633 |
| chr2 | 388882 | 388883 | C | T | AC105393.1 |  | ENST00000431911 | 0 | None | intron_variant | LOW |  | None |  | None | 0.12244898 |
| chr2 | 423066 | 423067 | G | C | AC105393.2 |  | ENST00000449119 | 0 | None | downstream_gene_variant | LOW |  | None |  | None | 0.247191011 |
| chr2 | 560290 | 560291 | A | G | AC093326.3 |  | ENST00000444079 | 0 | None | splice_region_variant | LOW |  | None |  | None | 0.169014085 |
| chr2 | 1159870 | 1159871 | G | C | SNTG2 |  | ENST00000308624 | 0 | None | intron_variant | LOW |  | None |  | None | 0.12345679 |
| chr2 | 1444795 | 1444796 | A | G | TPO |  | ENST00000346956 | 0 | None | intron_variant | LOW |  | None |  | None | 0.222222222 |
| chr2 | 1971062 | 1971063 | T | C | MYT1L |  | ENST00000399161 | 0 | None | intron_variant | LOW |  | None |  | None | 0.266666667 |
| chr2 | 2308276 | 2308277 | C | A | MYT1L |  | ENST00000399161 | 0 | None | intron_variant | LOW |  | None |  | None | 0.16 |
| chr2 | 2312078 | 2312079 | G | A | MYT1L |  | ENST00000399161 | 0 | None | intron_variant | LOW |  | None |  | None | 0.184210526 |
| chr2 | 3074844 | 3074845 | C | A | AC019118.2 |  | ENST00000457478 | 0 | None | intron_variant | LOW |  | None |  | None | 0.263157895 |
| chr2 | 3719295 | 3719296 | C | T | ALLC |  | ENST00000252505 | 0 | None | intron_variant | LOW |  | None |  | None | 0.191176471 |
| chr2 | 5968859 | 5968860 | G | T | None |  |  | 0 | None | intergenic_variant | LOW |  | None |  | None | 0.212765957 |
| chr2 | 6642708 | 6642709 | T | C | AC021021.2 |  | ENST00000436082 | 0 | None | intron_variant | LOW |  | None |  | None | 0.303571429 |
| chr2 | 7562931 | 7562932 | A | C | AC013460.1 |  | ENST00000419713 | 0 | None | intron_variant | LOW |  | None |  | None | 0.183673469 |
| chr2 | 8017589 | 8017590 | G | C | None |  |  | 0 | None | intergenic_variant | LOW |  | None |  | None | 0.177419355 |
| chr2 | 9077777 | 9077778 | G | A | MBOAT2 |  | ENST00000462696 | 0 | None | intron_variant | LOW |  | None |  | None | 0.171428571 |
| chr2 | 9275124 | 9275125 | G | T | None |  |  | 0 | None | intergenic_variant | LOW |  | None |  | None | 0.129032258 |
| chr2 | 10246840 | 10246841 | A | C | None |  |  | 0 | None | intergenic_variant | LOW |  | None |  | None | 0.160714286 |
| chr2 | 10472107 | 10472108 | A | G | HPCAL1 |  | ENST00000423674 | 0 | None | intron_variant | LOW |  | None |  | None | 0.145833333 |
| chr2 | 10595815 | 10595816 | G | T | RP11-320M2.1 |  | ENST00000553181 | 0 | None | downstream_gene_variant | LOW |  | None |  | None | 0.160714286 |
| chr2 | 12599286 | 12599287 | C | A | AC096559.1 |  | ENST00000412294 | 0 | None | intron_variant | LOW |  | None |  | None | 0.320754717 |
| chr2 | 13921687 | 13921688 | G | A | LINC00276 |  | ENST00000417751 | 1 | None | intron_variant | LOW |  | None |  | None | 0.288888889 |
| chr2 | 14240441 | 14240442 | T | C | LINC00276 |  | ENST00000417751 | 0 | None | intron_variant | LOW |  | None |  | None | 0.189655172 |
| chr2 | 16649563 | 16649564 | A | C | None |  |  | 0 | None | intergenic_variant | LOW |  | None |  | None | 0.232876712 |
| chr2 | 17648256 | 17648257 | A | T | None |  |  | 0 | None | intergenic_variant | LOW |  | None |  | None | 0.215384615 |
| chr2 | 17877051 | 17877052 | C | T | SMC6 |  | ENST00000351948 | 0 | None | intron_variant | LOW |  | None |  | None | 0.266666667 |
| chr2 | 18246588 | 18246589 | T | A | KCNS3 |  | ENST00000465292 | 0 | None | intron_variant | LOW |  | None |  | None | 0.25862069 |
| chr2 | 19156049 | 19156050 | T | A | None |  |  | 0 | None | intergenic_variant | LOW |  | None |  | None | 0.132075472 |
| chr2 | 21584132 | 21584133 | C | T | AC067959.1 |  | ENST00000435237 | 0 | None | intron_variant | LOW |  | None |  | None | 0.268292683 |
| chr2 | 22717187 | 22717188 | A | T | AC068490.2 |  | ENST00000450551 | 0 | None | intron_variant | LOW |  | None |  | None | 0.314814815 |
| chr2 | 23076729 | 23076730 | A | T | None |  |  | 0 | None | intergenic_variant | LOW |  | None |  | None | 0.174603175 |
| chr2 | 23322756 | 23322757 | A | G | AC016768.1 |  | ENST00000440785 | 0 | None | intron_variant | LOW |  | None |  | None | 0.28 |
| chr2 | 24532169 | 24532170 | T | C | ITSN2 |  | ENST00000355123 | 0 | None | intron_variant | LOW |  | None |  | None | 0.169230769 |
| chr2 | 24577650 | 24577651 | G | A | ITSN2 |  | ENST00000361999 | 0 | None | intron_variant | LOW |  | None |  | None | 0.228070175 |
| chr2 | 25426591 | 25426592 | G | A | AC012457.2 |  | ENST00000431650 | 0 | None | upstream_gene_variant | LOW |  | None |  | None | 0.238095238 |
| chr2 | 25768744 | 25768745 | C | T | DTNB |  | ENST00000406818 | 0 | None | intron_variant | LOW |  | None |  | None | 0.23255814 |
| chr2 | 25929001 | 25929002 | A | G | None |  |  | 1 | None | intergenic_variant | LOW |  | None |  | None | 0.120879121 |
| chr2 | 26320723 | 26320724 | T | G | RAB10 |  | ENST00000264710 | 0 | None | intron_variant | LOW |  | None |  | None | 0.255319149 |
| chr2 | 26445685 | 26445686 | T | C | HADHA |  | ENST00000457468 | 0 | None | intron_variant | LOW |  | None |  | None | 0.189655172 |
| chr2 | 26631079 | 26631080 | C | A | DRC1 |  | ENST00000288710 | 0 | None | intron_variant | LOW |  | None |  | None | 0.197368421 |
| chr2 | 26788432 | 26788433 | G | A | C2orf70 |  | ENST00000409392 | 0 | None | intron_variant | LOW |  | None |  | None | 0.220338983 |
| chr2 | 26973304 | 26973305 | C | T | None |  |  | 0 | None | intergenic_variant | LOW |  | None |  | None | 0.06741573 |
| chr2 | 29075049 | 29075050 | G | A | TRMT61B |  | ENST00000306108 | 0 | None | intron_variant | LOW |  | None |  | None | 0.228070175 |
| chr2 | 29075064 | 29075065 | G | C | TRMT61B |  | ENST00000306108 | 0 | None | intron_variant | LOW |  | None |  | None | 0.215686275 |
| chr2 | 31705119 | 31705120 | T | A | None |  |  | 1 | None | intergenic_variant | LOW |  | None |  | None | 0.3125 |
| chr2 | 32652566 | 32652567 | T | A | BIRC6 |  | ENST00000421745 | 0 | None | intron_variant | LOW |  | None |  | None | 0.172413793 |
| chr2 | 32867629 | 32867630 | T | G | TTC27 |  | ENST00000317907 | 0 | None | intron_variant | LOW |  | None |  | None | 0.173076923 |
| chr2 | 32886044 | 32886045 | T | A | TTC27 |  | ENST00000317907 | 0 | None | intron_variant | LOW |  | None |  | None | 0.088235294 |
| chr2 | 34051255 | 34051256 | T | G | AC009499.1 |  | ENST00000366209 | 1 | None | intron_variant | LOW |  | None |  | None | 0.152173913 |
| chr2 | 34641483 | 34641484 | A | G | AC073218.1 |  | ENST00000422558 | 0 | None | intron_variant | LOW |  | None |  | None | 0.211538462 |
| chr2 | 35162964 | 35162965 | C | A | AC012593.1 |  | ENST00000588650 | 0 | None | intron_variant | LOW |  | None |  | None | 0.271428571 |
| chr2 | 35378605 | 35378606 | A | T | AC012593.1 |  | ENST00000586769 | 0 | None | intron_variant | LOW |  | None |  | None | 0.175 |
| chr2 | 35467565 | 35467566 | T | C | None |  |  | 0 | None | intergenic_variant | LOW |  | None |  | None | 0.14893617 |
| chr2 | 36316956 | 36316957 | A | G | None |  |  | 0 | None | intergenic_variant | LOW |  | None |  | None | 0.096385542 |
| chr2 | 37575477 | 37575478 | G | A | QPCT |  | ENST00000537448 | 0 | None | intron_variant | LOW |  | None |  | None | 0.142857143 |
| chr2 | 38119909 | 38119910 | A | G | None |  |  | 1 | None | intergenic_variant | LOW |  | None |  | None | 0.105263158 |
| chr2 | 38133951 | 38133952 | G | A | None |  |  | 0 | None | intergenic_variant | LOW |  | None |  | None | 0.142857143 |
| chr2 | 38611898 | 38611899 | T | C | None |  |  | 0 | None | intergenic_variant | LOW |  | None |  | None | 0.112676056 |
| chr2 | 38768956 | 38768957 | T | C | None |  |  | 0 | None | intergenic_variant | LOW |  | None |  | None | 0.186440678 |
| chr2 | 39405011 | 39405012 | A | T | CDKL4 |  | ENST00000451199 | 0 | None | intron_variant | LOW |  | None |  | None | 0.12962963 |
| chr2 | 40757553 | 40757554 | C | T | SLC8A1 |  | ENST00000405269 | 0 | None | intron_variant | LOW |  | None |  | None | 0.103448276 |
| chr2 | 40765065 | 40765066 | C | A | SLC8A1 |  | ENST00000405269 | 0 | None | intron_variant | LOW |  | None |  | None | 0.158730159 |
| chr2 | 41631308 | 41631309 | C | A | None |  |  | 0 | None | intergenic_variant | LOW |  | None |  | None | 0.146341463 |
| chr2 | 42187756 | 42187757 | C | T | None |  |  | 0 | None | intergenic_variant | LOW |  | None |  | None | 0.260869565 |
| chr2 | 43620679 | 43620680 | C | T | THADA |  | ENST00000330266 | 0 | None | intron_variant | LOW |  | None |  | None | 0.18 |
| chr2 | 43998041 | 43998042 | C | A | DYNC2LI1 |  | ENST00000443170 | 0 | None | upstream_gene_variant | LOW |  | None |  | None | 0.238095238 |
| chr2 | 44087047 | 44087048 | C | G | ABCG8 |  | ENST00000272286 | 0 | None | intron_variant | LOW |  | None |  | None | 0.174603175 |
| chr2 | 44484758 | 44484759 | C | T | None |  |  | 0 | None | intergenic_variant | LOW |  | None |  | None | 0.280701754 |
| chr2 | 45985467 | 45985468 | T | C | PRKCE |  | ENST00000421201 | 0 | None | intron_variant | LOW |  | None |  | None | 0.225806452 |
| chr2 | 46021857 | 46021858 | C | T | PRKCE |  | ENST00000306156 | 0 | None | intron_variant | LOW |  | None |  | None | 0.16 |
| chr2 | 46036745 | 46036746 | G | C | PRKCE |  | ENST00000306156 | 0 | None | intron_variant | LOW |  | None |  | None | 0.138461538 |
| chr2 | 48723047 | 48723048 | A | G | PPP1R21 |  | ENST00000281394 | 0 | None | intron_variant | LOW |  | None |  | None | 0.169014085 |
| chr2 | 49942193 | 49942194 | C | G | None |  |  | 0 | None | intergenic_variant | LOW |  | None |  | None | 0.178571429 |
| chr2 | 50386065 | 50386066 | G | A | NRXN1 |  | ENST00000406859 | 0 | None | intron_variant | LOW |  | None |  | None | 0.186440678 |
| chr2 | 50459134 | 50459135 | T | C | NRXN1 |  | ENST00000401669 | 0 | None | intron_variant | LOW |  | None |  | None | 0.208333333 |
| chr2 | 50940079 | 50940080 | T | A | NRXN1 |  | ENST00000406316 | 0 | None | intron_variant | LOW |  | None |  | None | 0.25 |
| chr2 | 51061881 | 51061882 | C | T | NRXN1 |  | ENST00000401669 | 0 | None | intron_variant | LOW |  | None |  | None | 0.301886792 |
| chr2 | 51537744 | 51537745 | C | T | AC007682.1 |  | ENST00000440698 | 0 | None | intron_variant | LOW |  | None |  | None | 0.173913043 |
| chr2 | 51821528 | 51821529 | G | T | AC007682.1 |  | ENST00000440698 | 0 | None | intron_variant | LOW |  | None |  | None | 0.170212766 |
| chr2 | 51839935 | 51839936 | C | T | AC007682.1 |  | ENST00000440698 | 0 | None | intron_variant | LOW |  | None |  | None | 0.236363636 |
| chr2 | 52131093 | 52131094 | C | A | AC007682.1 |  | ENST00000440698 | 0 | None | intron_variant | LOW |  | None |  | None | 0.285714286 |
| chr2 | 52141074 | 52141075 | G | A | AC007682.1 |  | ENST00000440698 | 0 | None | intron_variant | LOW |  | None |  | None | 0.184210526 |
| chr2 | 52275976 | 52275977 | G | C | AC007682.1 |  | ENST00000440698 | 0 | None | intron_variant | LOW |  | None |  | None | 0.137254902 |
| chr2 | 52290918 | 52290919 | C | T | AC007682.1 |  | ENST00000440698 | 0 | None | intron_variant | LOW |  | None |  | None | 0.195652174 |
| chr2 | 52307457 | 52307458 | G | A | AC007682.1 |  | ENST00000440698 | 0 | None | intron_variant | LOW |  | None |  | None | 0.1875 |
| chr2 | 52636212 | 52636213 | T | A | AC007682.1 |  | ENST00000440698 | 0 | None | downstream_gene_variant | LOW |  | None |  | None | 0.282051282 |
| chr2 | 52677249 | 52677250 | T | A | None |  |  | 0 | None | intergenic_variant | LOW |  | None |  | None | 0.245283019 |
| chr2 | 52809101 | 52809102 | G | T | None |  |  | 0 | None | intergenic_variant | LOW |  | None |  | None | 0.274509804 |
| chr2 | 53260577 | 53260578 | G | T | None |  |  | 0 | None | intergenic_variant | LOW |  | None |  | None | 0.12 |
| chr2 | 53346676 | 53346677 | G | A | None |  |  | 0 | None | intergenic_variant | LOW |  | None |  | None | 0.1875 |
| chr2 | 53419144 | 53419145 | C | T | None |  |  | 0 | None | intergenic_variant | LOW |  | None |  | None | 0.170212766 |
| chr2 | 55854733 | 55854734 | C | T | None |  |  | 0 | None | intergenic_variant | LOW |  | None |  | None | 0.163265306 |
| chr2 | 56044097 | 56044098 | A | T | None |  |  | 0 | None | intergenic_variant | LOW |  | None |  | None | 0.195652174 |
| chr2 | 56288478 | 56288479 | C | T | RP11-481J13.1 |  | ENST00000606639 | 1 | None | intron_variant | LOW |  | None |  | None | 0.103448276 |
| chr2 | 56538780 | 56538781 | T | C | CCDC85A |  | ENST00000407595 | 0 | None | intron_variant | LOW |  | None |  | None | 0.245283019 |
| chr2 | 56852549 | 56852550 | C | A | None |  |  | 0 | None | intergenic_variant | LOW |  | None |  | None | 0.245901639 |
| chr2 | 56875316 | 56875317 | A | T | None |  |  | 0 | None | intergenic_variant | LOW |  | None |  | None | 0.142857143 |
| chr2 | 57130765 | 57130766 | A | G | None |  |  | 0 | None | intergenic_variant | LOW |  | None |  | None | 0.214285714 |
| chr2 | 57168813 | 57168814 | G | C | None |  |  | 0 | None | intergenic_variant | LOW |  | None |  | None | 0.272727273 |
| chr2 | 57338046 | 57338047 | T | G | None |  |  | 0 | None | intergenic_variant | LOW |  | None |  | None | 0.276595745 |
| chr2 | 57361071 | 57361072 | T | A | None |  |  | 0 | None | intergenic_variant | LOW |  | None |  | None | 0.185185185 |
| chr2 | 57389058 | 57389059 | A | G | None |  |  | 0 | None | intergenic_variant | LOW |  | None |  | None | 0.2 |
| chr2 | 57431635 | 57431636 | C | A | None |  |  | 0 | None | intergenic_variant | LOW |  | None |  | None | 0.212765957 |
| chr2 | 57809110 | 57809111 | A | T | None |  |  | 0 | None | intergenic_variant | LOW |  | None |  | None | 0.214285714 |
| chr2 | 57933909 | 57933910 | A | G | None |  |  | 0 | None | intergenic_variant | LOW |  | None |  | None | 0.204545455 |
| chr2 | 58025674 | 58025675 | A | G | None |  |  | 0 | None | intergenic_variant | LOW |  | None |  | None | 0.15 |
| chr2 | 58065795 | 58065796 | T | C | None |  |  | 0 | None | intergenic_variant | LOW |  | None |  | None | 0.153846154 |
| chr2 | 58661852 | 58661853 | G | A | AC007092.1 |  | ENST00000429664 | 0 | None | intron_variant | LOW |  | None |  | None | 0.298245614 |
| chr2 | 58754865 | 58754866 | T | G | AC007092.1 |  | ENST00000449448 | 1 | None | intron_variant | LOW |  | None |  | None | 0.135135135 |
| chr2 | 58834211 | 58834212 | C | T | AC007092.1 |  | ENST00000429664 | 0 | None | intron_variant | LOW |  | None |  | None | 0.290909091 |
| chr2 | 59062922 | 59062923 | A | T | AC007092.1 |  | ENST00000429095 | 0 | None | intron_variant | LOW |  | None |  | None | 0.140350877 |
| chr2 | 60028484 | 60028485 | C | T | RP11-444A22.1 |  | ENST00000606382 | 0 | None | intron_variant | LOW |  | None |  | None | 0.189655172 |
| chr2 | 60346391 | 60346392 | C | T | None |  |  | 0 | None | intergenic_variant | LOW |  | None |  | None | 0.166666667 |
| chr2 | 60355571 | 60355572 | G | A | None |  |  | 0 | None | intergenic_variant | LOW |  | None |  | None | 0.166666667 |
| chr2 | 60359299 | 60359300 | C | T | None |  |  | 0 | None | intergenic_variant | LOW |  | None |  | None | 0.2 |
| chr2 | 60379864 | 60379865 | G | A | None |  |  | 0 | None | intergenic_variant | LOW |  | None |  | None | 0.275862069 |
| chr2 | 60416480 | 60416481 | G | A | None |  |  | 0 | None | intergenic_variant | LOW |  | None |  | None | 0.14516129 |
| chr2 | 60868619 | 60868620 | G | C | RN7SL361P |  | ENST00000470785 | 0 | None | downstream_gene_variant | LOW |  | None |  | None | 0.131578947 |
| chr2 | 61090709 | 61090710 | G | T | AC010733.4 |  | ENST00000452343 | 0 | None | intron_variant | LOW |  | None |  | None | 0.203389831 |
| chr2 | 61601472 | 61601473 | T | C | USP34 |  | ENST00000398571 | 0 | None | intron_variant | LOW |  | None |  | None | 0.277777778 |
| chr2 | 62318429 | 62318430 | T | A | COMMD1 |  | ENST00000311832 | 1 | None | intron_variant | LOW |  | None |  | None | 0.101449275 |
| chr2 | 62815861 | 62815862 | T | C | AC092155.4 |  | ENST00000444672 | 0 | None | downstream_gene_variant | LOW |  | None |  | None | 0.389830508 |
| chr2 | 63297284 | 63297285 | T | A | None |  |  | 0 | None | intergenic_variant | LOW |  | None |  | None | 0.258064516 |
| chr2 | 65798968 | 65798969 | G | A | AC074391.1 |  | ENST00000377977 | 0 | None | intron_variant | LOW |  | None |  | None | 0.271186441 |
| chr2 | 66382659 | 66382660 | A | G | None |  |  | 0 | None | intergenic_variant | LOW |  | None |  | None | 0.296875 |
| chr2 | 66405769 | 66405770 | C | A | None |  |  | 0 | None | intergenic_variant | LOW |  | None |  | None | 0.152173913 |
| chr2 | 66859647 | 66859648 | C | G | AC007392.3 |  | ENST00000433396 | 0 | None | intron_variant | LOW |  | None |  | None | 0.163636364 |
| chr2 | 67134058 | 67134059 | A | T | AC007403.2 |  | ENST00000426260 | 0 | None | intron_variant | LOW |  | None |  | None | 0.266666667 |
| chr2 | 67520836 | 67520837 | T | C | AC023115.2 |  | ENST00000452716 | 0 | None | intron_variant | LOW |  | None |  | None | 0.24137931 |
| chr2 | 67533768 | 67533769 | C | T | None |  |  | 0 | None | intergenic_variant | LOW |  | None |  | None | 0.142857143 |
| chr2 | 68559075 | 68559076 | G | T | None |  |  | 0 | None | intergenic_variant | LOW |  | None |  | None | 0.16 |
| chr2 | 68716788 | 68716789 | T | C | APLF |  | ENST00000303795 | 0 | None | intron_variant | LOW |  | None |  | None | 0.28 |
| chr2 | 69257475 | 69257476 | A | G | ANTXR1 |  | ENST00000409829 | 0 | None | intron_variant | LOW |  | None |  | None | 0.145454545 |
| chr2 | 70333007 | 70333008 | A | G | AC016700.3 |  | ENST00000442326 | 0 | None | upstream_gene_variant | LOW |  | None |  | None | 0.178082192 |
| chr2 | 70333009 | 70333010 | G | A | AC016700.3 |  | ENST00000442326 | 0 | None | upstream_gene_variant | LOW |  | None |  | None | 0.097222222 |
| chr2 | 70959800 | 70959801 | G | A | ADD2 |  | ENST00000413157 | 0 | None | intron_variant | LOW |  | None |  | None | 0.278481013 |
| chr2 | 71473891 | 71473892 | C | A | None |  |  | 0 | None | intergenic_variant | LOW |  | None |  | None | 0.151515152 |
| chr2 | 72304512 | 72304513 | T | A | None |  |  | 0 | None | intergenic_variant | LOW |  | None |  | None | 0.127272727 |
| chr2 | 73493785 | 73493786 | G | A | FBXO41 | F | ENST00000295133 | 0 | None | synonymous_variant | LOW |  | None |  | None | 0.256097561 |
| chr2 | 76321228 | 76321229 | C | T | None |  |  | 0 | None | intergenic_variant | LOW |  | None |  | None | 0.211538462 |
| chr2 | 76742705 | 76742706 | C | T | None |  |  | 1 | None | intergenic_variant | LOW |  | None |  | None | 0.21875 |
| chr2 | 78135604 | 78135605 | A | T | None |  |  | 0 | None | intergenic_variant | LOW |  | None |  | None | 0.157894737 |
| chr2 | 78174662 | 78174663 | A | T | None |  |  | 0 | None | intergenic_variant | LOW |  | None |  | None | 0.188679245 |
| chr2 | 78324756 | 78324757 | A | G | AC012494.1 |  | ENST00000439259 | 0 | None | intron_variant | LOW |  | None |  | None | 0.142857143 |
| chr2 | 78367653 | 78367654 | A | G | None |  |  | 0 | None | intergenic_variant | LOW |  | None |  | None | 0.224489796 |
| chr2 | 78439921 | 78439922 | A | G | None |  |  | 0 | None | intergenic_variant | LOW |  | None |  | None | 0.260869565 |
| chr2 | 78656500 | 78656501 | A | T | None |  |  | 0 | None | intergenic_variant | LOW |  | None |  | None | 0.254545455 |
| chr2 | 79005128 | 79005129 | T | A | None |  |  | 0 | None | intergenic_variant | LOW |  | None |  | None | 0.115942029 |
| chr2 | 79096103 | 79096104 | A | G | None |  |  | 0 | None | intergenic_variant | LOW |  | None |  | None | 0.226415094 |
| chr2 | 79419752 | 79419753 | G | A | CTNNA2 |  | ENST00000466387 | 0 | None | intron_variant | LOW |  | None |  | None | 0.196078431 |
| chr2 | 79545404 | 79545405 | C | T | CTNNA2 |  | ENST00000466387 | 0 | None | intron_variant | LOW |  | None |  | None | 0.155555556 |
| chr2 | 79644016 | 79644017 | C | T | CTNNA2 |  | ENST00000466387 | 0 | None | intron_variant | LOW |  | None |  | None | 0.216216216 |
| chr2 | 80500567 | 80500568 | C | A | CTNNA2 |  | ENST00000541047 | 0 | None | intron_variant | LOW |  | None |  | None | 0.285714286 |
| chr2 | 80855264 | 80855265 | C | A | CTNNA2 |  | ENST00000466387 | 0 | None | intron_variant | LOW |  | None |  | None | 0.235294118 |
| chr2 | 81217405 | 81217406 | G | A | None |  |  | 0 | None | intergenic_variant | LOW |  | None |  | None | 0.259259259 |
| chr2 | 82298292 | 82298293 | C | T | None |  |  | 1 | None | intergenic_variant | LOW |  | None |  | None | 0.150943396 |
| chr2 | 82939514 | 82939515 | T | C | None |  |  | 0 | None | intergenic_variant | LOW |  | None |  | None | 0.13559322 |
| chr2 | 82991444 | 82991445 | G | T | None |  |  | 0 | None | intergenic_variant | LOW |  | None |  | None | 0.166666667 |
| chr2 | 83086896 | 83086897 | C | T | AC098817.5 |  | ENST00000455595 | 0 | None | downstream_gene_variant | LOW |  | None |  | None | 0.222222222 |
| chr2 | 83120924 | 83120925 | C | A | None |  |  | 0 | None | intergenic_variant | LOW |  | None |  | None | 0.163636364 |
| chr2 | 83941638 | 83941639 | C | T | None |  |  | 0 | None | intergenic_variant | LOW |  | None |  | None | 0.115942029 |
| chr2 | 85172854 | 85172855 | A | G | None |  |  | 1 | None | intergenic_variant | LOW |  | None |  | None | 0.179104478 |
| chr2 | 85853230 | 85853231 | C | G | USP39 |  | ENST00000409766 | 0 | None | intron_variant | LOW |  | None |  | None | 0.191780822 |
| chr2 | 86304810 | 86304811 | C | T | POLR1A |  | ENST00000263857 | 0 | None | intron_variant | LOW |  | None |  | None | 0.290322581 |
| chr2 | 86607219 | 86607220 | G | T | None |  |  | 0 | None | intergenic_variant | LOW |  | None |  | None | 0.271186441 |
| chr2 | 87061646 | 87061647 | T | C | CD8B |  | ENST00000393759 | 1 | None | intron_variant | LOW |  | None |  | None | 0.163636364 |
| chr2 | 87591681 | 87591682 | T | A | AC068279.1 |  | ENST00000393756 | 0 | None | upstream_gene_variant | LOW |  | None |  | None | 0.11627907 |
| chr2 | 88243283 | 88243284 | A | C | RGPD2 |  | ENST00000420840 | 0 | None | intron_variant | LOW |  | None |  | None | 0.354166667 |
| chr2 | 88243451 | 88243452 | G | T | RGPD2 |  | ENST00000420840 | 0 | None | intron_variant | LOW |  | None |  | None | 0.296296296 |
| chr2 | 88666400 | 88666401 | G | A | RNU6-568P |  | ENST00000517220 | 0 | None | downstream_gene_variant | LOW |  | None |  | None | 0.19047619 |
| chr2 | 88794467 | 88794468 | T | C | None |  |  | 0 | None | intergenic_variant | LOW |  | None |  | None | 0.115384615 |
| chr2 | 88946503 | 88946504 | G | A | None |  |  | 0 | None | intergenic_variant | LOW |  | None |  | None | 0.236842105 |
| chr2 | 89072221 | 89072222 | C | T | ANKRD36BP2 |  | ENST00000427055 | 1 | None | intron_variant | LOW |  | None |  | None | 0.109090909 |
| chr2 | 89455443 | 89455444 | T | A | IGKV6-21 |  | ENST00000390256 | 0 | None | downstream_gene_variant | LOW |  | None |  | None | 0.227272727 |
| chr2 | 89473653 | 89473654 | A | G | IGKV2-24 |  | ENST00000484817 | 0 | None | downstream_gene_variant | LOW |  | None |  | None | 0.181818182 |
| chr2 | 89599220 | 89599221 | T | C | IGKV1-37 |  | ENST00000465170 | 0 | None | upstream_gene_variant | LOW |  | None |  | None | 0.217391304 |
| chr2 | 89861265 | 89861266 | C | A | None |  |  | 0 | None | intergenic_variant | LOW |  | None |  | None | 0.1 |
| chr2 | 90243688 | 90243689 | A | G | None |  |  | 0 | None | intergenic_variant | LOW |  | None |  | None | 0.16 |
| chr2 | 90243912 | 90243913 | T | C | IGKV1D-43 |  | ENST00000468879 | 0 | None | upstream_gene_variant | LOW |  | None |  | None | 0.138888889 |
| chr2 | 90431860 | 90431861 | T | A | None |  |  | 1 | None | intergenic_variant | LOW |  | None |  | None | 0.080357143 |
| chr2 | 90431882 | 90431883 | G | A | None |  |  | 0 | None | intergenic_variant | LOW |  | None |  | None | 0.121212121 |
| chr2 | 90466784 | 90466785 | T | G | None |  |  | 0 | None | intergenic_variant | LOW |  | None |  | None | 0.154929577 |
| chr2 | 90466800 | 90466801 | A | T | None |  |  | 0 | None | intergenic_variant | LOW |  | None |  | None | 0.173913043 |
| chr2 | 90472761 | 90472762 | T | C | None |  |  | 1 | None | intergenic_variant | LOW |  | None |  | None | 0.078947368 |
| chr2 | 90481511 | 90481512 | C | G | None |  |  | 0 | None | intergenic_variant | LOW |  | None |  | None | 0.119402985 |
| chr2 | 91661036 | 91661037 | C | T | None |  |  | 0 | None | intergenic_variant | LOW |  | None |  | None | 0.063694268 |
| chr2 | 91661046 | 91661047 | C | T | None |  |  | 1 | None | intergenic_variant | LOW |  | None |  | None | 0.075862069 |
| chr2 | 91693016 | 91693017 | T | A | AC018696.2 |  | ENST00000366169 | 1 | None | downstream_gene_variant | LOW |  | None |  | None | 0.070175439 |
| chr2 | 91728544 | 91728545 | G | A | AC018696.5 |  | ENST00000419163 | 1 | None | upstream_gene_variant | LOW |  | None |  | None | 0.24 |
| chr2 | 91728544 | 91728545 | G | C | AC018696.5 |  | ENST00000419163 | 1 | None | upstream_gene_variant | LOW |  | None |  | None | 0.26 |
| chr2 | 91758303 | 91758304 | G | A | None |  |  | 0 | None | intergenic_variant | LOW |  | None |  | None | 0.11 |
| chr2 | 91880094 | 91880095 | G | C | AC027612.3 |  | ENST00000436174 | 1 | None | intron_variant | LOW |  | None |  | None | 0.138461538 |
| chr2 | 91977652 | 91977653 | T | A | None |  |  | 0 | None | intergenic_variant | LOW |  | None |  | None | 0.056179775 |
| chr2 | 92062371 | 92062372 | A | G | PABPC1P6 |  | ENST00000445594 | 1 | None | upstream_gene_variant | LOW |  | None |  | None | 0.25 |
| chr2 | 92072833 | 92072834 | T | C | SLC9B1P2 |  | ENST00000398120 | 0 | None | intron_variant | LOW |  | None |  | None | 0.101265823 |
| chr2 | 92098236 | 92098237 | C | G | SLC9B1P2 |  | ENST00000606405 | 1 | None | intron_variant | LOW |  | None |  | None | 0.12 |
| chr2 | 92098236 | 92098237 | C | A | SLC9B1P2 |  | ENST00000606405 | 1 | None | intron_variant | LOW |  | None |  | None | 0.20 |
| chr2 | 92169134 | 92169135 | C | G | None |  |  | 0 | None | intergenic_variant | LOW |  | None |  | None | 0.153846154 |
| chr2 | 92194211 | 92194212 | T | C | AC128677.3 |  | ENST00000436016 | 1 | None | downstream_gene_variant | LOW |  | None |  | None | 0.07 |
| chr2 | 92194211 | 92194212 | T | A | AC128677.3 |  | ENST00000436016 | 1 | None | downstream_gene_variant | LOW |  | None |  | None | 0.49 |
| chr2 | 92243805 | 92243806 | T | C | None |  |  | 0 | None | intergenic_variant | LOW |  | None |  | None | 0.103703704 |
| chr2 | 92259475 | 92259476 | G | C | None |  |  | 0 | None | intergenic_variant | LOW |  | None |  | None | 0.06 |
| chr2 | 92279295 | 92279296 | C | T | None |  |  | 0 | None | intergenic_variant | LOW |  | None |  | None | 0.061728395 |
| chr2 | 95471103 | 95471104 | T | G | AC073464.11 |  | ENST00000429823 | 0 | None | intron_variant | LOW |  | None |  | None | 0.14 |
| chr2 | 95475009 | 95475010 | A | T | ANKRD20A8P |  | ENST00000432432 | 0 | None | downstream_gene_variant | LOW |  | None |  | None | 0.061538462 |
| chr2 | 95475039 | 95475040 | A | T | ANKRD20A8P |  | ENST00000432432 | 1 | None | downstream_gene_variant | LOW |  | None |  | None | 0.051282051 |
| chr2 | 95475041 | 95475042 | A | G | ANKRD20A8P |  | ENST00000432432 | 1 | None | downstream_gene_variant | LOW |  | None |  | None | 0.05 |
| chr2 | 95515109 | 95515110 | C | A | ANKRD20A8P |  | ENST00000432432 | 1 | None | intron_variant | LOW |  | None |  | None | 0.15 |
| chr2 | 95582589 | 95582590 | G | T | AC097374.2 |  | ENST00000582835 | 0 | None | intron_variant | LOW |  | None |  | None | 0.25 |
| chr2 | 96052957 | 96052958 | G | T | KCNIP3 |  | ENST00000295225 | 0 | None | downstream_gene_variant | LOW |  | None |  | None | 0.142857143 |
| chr2 | 96433161 | 96433162 | T | C | None |  |  | 1 | None | intergenic_variant | LOW |  | None |  | None | 0.153846154 |
| chr2 | 96433197 | 96433198 | A | G | None |  |  | 1 | None | intergenic_variant | LOW |  | None |  | None | 0.12 |
| chr2 | 96499103 | 96499104 | C | T | None |  |  | 0 | None | intergenic_variant | LOW |  | None |  | None | 0.103448276 |
| chr2 | 96522578 | 96522579 | T | A | ANKRD36C |  | ENST00000420871 | 1 | None | intron_variant | LOW |  | None |  | None | 0.29 |
| chr2 | 96522578 | 96522579 | T | C | ANKRD36C |  | ENST00000420871 | 1 | None | intron_variant | LOW |  | None |  | None | 0.34 |
| chr2 | 96603132 | 96603133 | T | C | ANKRD36C |  | ENST00000456556 | 0 | None | intron_variant | LOW |  | None |  | None | 0.068181818 |
| chr2 | 96619772 | 96619773 | A | C | ANKRD36C |  | ENST00000456556 | 1 | None | intron_variant | LOW |  | None |  | None | 0.061946903 |
| chr2 | 97829059 | 97829060 | G | C | ANKRD36 |  | ENST00000420699 | 1 | None | intron_variant | LOW |  | None |  | None | 0.064748201 |
| chr2 | 97915946 | 97915947 | T | C | ANKRD36 |  | ENST00000357042 | 0 | None | intron_variant | LOW |  | None |  | None | 0.255813953 |
| chr2 | 98171064 | 98171065 | C | T | ANKRD36B |  | ENST00000443455 | 0 | None | intron_variant | LOW |  | None |  | None | 0.204545455 |
| chr2 | 99950769 | 99950770 | A | C | TXNDC9 |  | ENST00000409705 | 0 | None | intron_variant | LOW |  | None |  | None | 0.156862745 |
| chr2 | 100564861 | 100564862 | C | A | AFF3 |  | ENST00000409579 | 0 | None | intron_variant | LOW |  | None |  | None | 0.245901639 |
| chr2 | 100564862 | 100564863 | C | T | AFF3 |  | ENST00000317233 | 0 | None | intron_variant | LOW |  | None |  | None | 0.233333333 |
| chr2 | 100694383 | 100694384 | G | C | AFF3 |  | ENST00000415384 | 0 | None | intron_variant | LOW |  | None |  | None | 0.233333333 |
| chr2 | 100966170 | 100966171 | C | T | None |  |  | 0 | None | intergenic_variant | LOW |  | None |  | None | 0.25 |
| chr2 | 101025811 | 101025812 | T | G | CHST10 |  | ENST00000420858 | 0 | None | intron_variant | LOW |  | None |  | None | 0.2 |
| chr2 | 103109326 | 103109327 | G | T | SLC9A4 |  | ENST00000295269 | 0 | None | intron_variant | LOW |  | None |  | None | 0.189655172 |
| chr2 | 103486098 | 103486099 | A | G | AC007251.2 |  | ENST00000447987 | 0 | None | downstream_gene_variant | LOW |  | None |  | None | 0.342105263 |
| chr2 | 103935128 | 103935129 | G | T | None |  |  | 0 | None | intergenic_variant | LOW |  | None |  | None | 0.157894737 |
| chr2 | 104277964 | 104277965 | C | T | None |  |  | 0 | None | intergenic_variant | LOW |  | None |  | None | 0.292682927 |
| chr2 | 104309442 | 104309443 | T | C | None |  |  | 0 | None | intergenic_variant | LOW |  | None |  | None | 0.176470588 |
| chr2 | 104590553 | 104590554 | A | G | RP11-76I14.1 |  | ENST00000537492 | 0 | None | intron_variant | LOW |  | None |  | None | 0.211538462 |
| chr2 | 104905102 | 104905103 | G | A | None |  |  | 0 | None | intergenic_variant | LOW |  | None |  | None | 0.145454545 |
| chr2 | 104926409 | 104926410 | C | A | None |  |  | 0 | None | intergenic_variant | LOW |  | None |  | None | 0.173913043 |
| chr2 | 104956021 | 104956022 | C | A | None |  |  | 0 | None | intergenic_variant | LOW |  | None |  | None | 0.27027027 |
| chr2 | 104967046 | 104967047 | C | G | None |  |  | 0 | None | intergenic_variant | LOW |  | None |  | None | 0.213114754 |
| chr2 | 106672265 | 106672266 | G | A | None |  |  | 0 | None | intergenic_variant | LOW |  | None |  | None | 0.114754098 |
| chr2 | 106904385 | 106904386 | A | C | AC114755.2 |  | ENST00000595915 | 1 | None | downstream_gene_variant | LOW |  | None |  | None | 0.345454545 |
| chr2 | 107883640 | 107883641 | A | G | AC006227.1 |  | ENST00000455614 | 0 | None | intron_variant | LOW |  | None |  | None | 0.15 |
| chr2 | 107899569 | 107899570 | C | A | AC006227.1 |  | ENST00000455614 | 0 | None | intron_variant | LOW |  | None |  | None | 0.137254902 |
| chr2 | 108059146 | 108059147 | C | T | AC096669.2 |  | ENST00000414300 | 1 | None | intron_variant | LOW |  | None |  | None | 0.227272727 |
| chr2 | 108120204 | 108120205 | C | T | AC096669.2 |  | ENST00000414300 | 0 | None | intron_variant | LOW |  | None |  | None | 0.229166667 |
| chr2 | 108329505 | 108329506 | G | A | None |  |  | 0 | None | intergenic_variant | LOW |  | None |  | None | 0.224489796 |
| chr2 | 108438450 | 108438451 | G | T | RGPD4 |  | ENST00000408999 | 1 | None | upstream_gene_variant | LOW |  | None |  | None | 0.146341463 |
| chr2 | 108597216 | 108597217 | T | C | None |  |  | 0 | None | intergenic_variant | LOW |  | None |  | None | 0.256410256 |
| chr2 | 109450686 | 109450687 | G | T | CCDC138 |  | ENST00000295124 | 1 | None | intron_variant | LOW |  | None |  | None | 0.130434783 |
| chr2 | 109764343 | 109764344 | A | G | SH3RF3 |  | ENST00000309415 | 0 | None | intron_variant | LOW |  | None |  | None | 0.145454545 |
| chr2 | 110882869 | 110882870 | T | C | NPHP1 | K/E | ENST00000417665 | 0 | None | missense_variant | MED | unknown | 0 | deleterious | 0 | 0.231578947 |
| chr2 | 112084832 | 112084833 | A | T | MIR4435-1HG |  | ENST00000431385 | 1 | None | intron_variant | LOW |  | None |  | None | 0.115942029 |
| chr2 | 112440150 | 112440151 | A | G | None |  |  | 0 | None | intergenic_variant | LOW |  | None |  | None | 0.274509804 |
| chr2 | 112493447 | 112493448 | T | G | None |  |  | 0 | None | intergenic_variant | LOW |  | None |  | None | 0.204301075 |
| chr2 | 112605012 | 112605013 | T | C | ANAPC1 |  | ENST00000427997 | 0 | None | intron_variant | LOW |  | None |  | None | 0.14084507 |
| chr2 | 112611678 | 112611679 | G | A | ANAPC1 |  | ENST00000427997 | 0 | None | intron_variant | LOW |  | None |  | None | 0.16 |
| chr2 | 112667602 | 112667603 | A | T | MERTK |  | ENST00000409780 | 0 | None | intron_variant | LOW |  | None |  | None | 0.193548387 |
| chr2 | 113016394 | 113016395 | C | T | ZC3H8 |  | ENST00000272570 | 0 | None | upstream_gene_variant | LOW |  | None |  | None | 0.138461538 |
| chr2 | 113189204 | 113189205 | C | A | RGPD8 |  | ENST00000302558 | 0 | None | intron_variant | LOW |  | None |  | None | 0.254237288 |
| chr2 | 113935693 | 113935694 | C | G | PSD4 |  | ENST00000441564 | 0 | None | intron_variant | LOW |  | None |  | None | 0.2 |
| chr2 | 114343837 | 114343838 | C | T | MIR1302-3 |  | ENST00000408128 | 1 | None | upstream_gene_variant | LOW |  | None |  | None | 0.2 |
| chr2 | 115093014 | 115093015 | C | A | None |  |  | 0 | None | intergenic_variant | LOW |  | None |  | None | 0.265306122 |
| chr2 | 115927978 | 115927979 | G | A | DPP10 |  | ENST00000436732 | 0 | None | intron_variant | LOW |  | None |  | None | 0.277777778 |
| chr2 | 116781760 | 116781761 | C | A | None |  |  | 0 | None | intergenic_variant | LOW |  | None |  | None | 0.25862069 |
| chr2 | 116828130 | 116828131 | G | T | None |  |  | 0 | None | intergenic_variant | LOW |  | None |  | None | 0.214285714 |
| chr2 | 116852662 | 116852663 | G | T | None |  |  | 0 | None | intergenic_variant | LOW |  | None |  | None | 0.232142857 |
| chr2 | 116890660 | 116890661 | G | A | None |  |  | 0 | None | intergenic_variant | LOW |  | None |  | None | 0.178571429 |
| chr2 | 116998583 | 116998584 | C | T | None |  |  | 0 | None | intergenic_variant | LOW |  | None |  | None | 0.213114754 |
| chr2 | 117021816 | 117021817 | C | A | None |  |  | 0 | None | intergenic_variant | LOW |  | None |  | None | 0.176470588 |
| chr2 | 117206459 | 117206460 | T | C | None |  |  | 0 | None | intergenic_variant | LOW |  | None |  | None | 0.301886792 |
| chr2 | 117227856 | 117227857 | C | A | None |  |  | 0 | None | intergenic_variant | LOW |  | None |  | None | 0.196078431 |
| chr2 | 117468500 | 117468501 | C | A | None |  |  | 0 | None | intergenic_variant | LOW |  | None |  | None | 0.183333333 |
| chr2 | 117598125 | 117598126 | C | A | None |  |  | 0 | None | intergenic_variant | LOW |  | None |  | None | 0.274509804 |
| chr2 | 117763025 | 117763026 | A | G | None |  |  | 0 | None | intergenic_variant | LOW |  | None |  | None | 0.274509804 |
| chr2 | 118306134 | 118306135 | C | G | None |  |  | 0 | None | intergenic_variant | LOW |  | None |  | None | 0.105263158 |
| chr2 | 118350927 | 118350928 | A | G | None |  |  | 0 | None | intergenic_variant | LOW |  | None |  | None | 0.265306122 |
| chr2 | 119370501 | 119370502 | G | T | None |  |  | 0 | None | intergenic_variant | LOW |  | None |  | None | 0.194444444 |
| chr2 | 119528933 | 119528934 | T | A | None |  |  | 0 | None | intergenic_variant | LOW |  | None |  | None | 0.203703704 |
| chr2 | 120408734 | 120408735 | C | T | PCDP1 |  | ENST00000602047 | 0 | None | intron_variant | LOW |  | None |  | None | 0.272727273 |
| chr2 | 121283841 | 121283842 | C | T | None |  |  | 0 | None | intergenic_variant | LOW |  | None |  | None | 0.164179104 |
| chr2 | 121772005 | 121772006 | G | C | None |  |  | 0 | None | intergenic_variant | LOW |  | None |  | None | 0.23880597 |
| chr2 | 122750592 | 122750593 | C | A | None |  |  | 0 | None | intergenic_variant | LOW |  | None |  | None | 0.18 |
| chr2 | 122879622 | 122879623 | G | T | None |  |  | 0 | None | intergenic_variant | LOW |  | None |  | None | 0.153846154 |
| chr2 | 122904578 | 122904579 | A | G | None |  |  | 0 | None | intergenic_variant | LOW |  | None |  | None | 0.290909091 |
| chr2 | 123189688 | 123189689 | C | T | None |  |  | 0 | None | intergenic_variant | LOW |  | None |  | None | 0.225 |
| chr2 | 123337858 | 123337859 | A | G | None |  |  | 0 | None | intergenic_variant | LOW |  | None |  | None | 0.119047619 |
| chr2 | 123496660 | 123496661 | G | A | None |  |  | 0 | None | intergenic_variant | LOW |  | None |  | None | 0.134615385 |
| chr2 | 123896164 | 123896165 | G | A | None |  |  | 0 | None | intergenic_variant | LOW |  | None |  | None | 0.238095238 |
| chr2 | 124353271 | 124353272 | T | C | None |  |  | 0 | None | intergenic_variant | LOW |  | None |  | None | 0.218181818 |
| chr2 | 124885345 | 124885346 | A | G | CNTNAP5 |  | ENST00000431078 | 0 | None | intron_variant | LOW |  | None |  | None | 0.227272727 |
| chr2 | 125389078 | 125389079 | G | A | CNTNAP5 |  | ENST00000431078 | 0 | None | intron_variant | LOW |  | None |  | None | 0.142857143 |
| chr2 | 125509965 | 125509966 | C | T | CNTNAP5 |  | ENST00000431078 | 0 | None | intron_variant | LOW |  | None |  | None | 0.145833333 |
| chr2 | 125578702 | 125578703 | G | A | CNTNAP5 |  | ENST00000431078 | 0 | None | intron_variant | LOW |  | None |  | None | 0.2 |
| chr2 | 125789226 | 125789227 | T | A | None |  |  | 0 | None | intergenic_variant | LOW |  | None |  | None | 0.243902439 |
| chr2 | 126650164 | 126650165 | G | A | None |  |  | 0 | None | intergenic_variant | LOW |  | None |  | None | 0.33 |
| chr2 | 126650164 | 126650165 | G | T | None |  |  | 0 | None | intergenic_variant | LOW |  | None |  | None | 0.48 |
| chr2 | 126650214 | 126650215 | A | C | None |  |  | 0 | None | intergenic_variant | LOW |  | None |  | None | 0.82 |
| chr2 | 126986284 | 126986285 | G | A | None |  |  | 0 | None | intergenic_variant | LOW |  | None |  | None | 0.117647059 |
| chr2 | 127092465 | 127092466 | T | C | AC023347.1 |  | ENST00000435352 | 0 | None | intron_variant | LOW |  | None |  | None | 0.246153846 |
| chr2 | 127450803 | 127450804 | G | A | GYPC |  | ENST00000259254 | 1 | None | intron_variant | LOW |  | None |  | None | 0.213114754 |
| chr2 | 128383434 | 128383435 | C | A | MYO7B |  | ENST00000389524 | 0 | None | intron_variant | LOW |  | None |  | None | 0.265625 |
| chr2 | 129585671 | 129585672 | C | G | None |  |  | 0 | None | intergenic_variant | LOW |  | None |  | None | 0.107142857 |
| chr2 | 129585745 | 129585746 | A | G | None |  |  | 0 | None | intergenic_variant | LOW |  | None |  | None | 0.101694915 |
| chr2 | 129757178 | 129757179 | A | C | None |  |  | 0 | None | intergenic_variant | LOW |  | None |  | None | 0.22 |
| chr2 | 130145088 | 130145089 | A | G | None |  |  | 0 | None | intergenic_variant | LOW |  | None |  | None | 0.14 |
| chr2 | 131006095 | 131006096 | G | C | AC134915.1 |  | ENST00000426461 | 1 | None | intron_variant | LOW |  | None |  | None | 0.171428571 |
| chr2 | 132176983 | 132176984 | G | T | GNAQP1 |  | ENST00000444815 | 0 | None | downstream_gene_variant | LOW |  | None |  | None | 0.233333333 |
| chr2 | 132513837 | 132513838 | G | T | C2orf27A |  | ENST00000355171 | 0 | None | intron_variant | LOW |  | None |  | None | 0.076923077 |
| chr2 | 132768949 | 132768950 | T | C | None |  |  | 0 | None | intergenic_variant | LOW |  | None |  | None | 0.071428571 |
| chr2 | 132780708 | 132780709 | T | A | None |  |  | 1 | None | intergenic_variant | LOW |  | None |  | None | 0.301369863 |
| chr2 | 132782920 | 132782921 | T | C | None |  |  | 0 | None | intergenic_variant | LOW |  | None |  | None | 0.065420561 |
| chr2 | 132798167 | 132798168 | G | C | AC093787.1 |  | ENST00000450396 | 0 | None | upstream_gene_variant | LOW |  | None |  | None | 0.09375 |
| chr2 | 132817050 | 132817051 | G | A | None |  |  | 1 | None | intergenic_variant | LOW |  | None |  | None | 0.170212766 |
| chr2 | 132827593 | 132827594 | G | T | None |  |  | 0 | None | intergenic_variant | LOW |  | None |  | None | 0.1 |
| chr2 | 132851261 | 132851262 | A | T | AC098826.4 |  | ENST00000433943 | 0 | None | intron_variant | LOW |  | None |  | None | 0.132352941 |
| chr2 | 132961399 | 132961400 | A | G | ANKRD30BL |  | ENST00000470729 | 0 | None | intron_variant | LOW |  | None |  | None | 0.191176471 |
| chr2 | 133094537 | 133094538 | G | A | None |  |  | 0 | None | intergenic_variant | LOW |  | None |  | None | 0.092592593 |
| chr2 | 134278398 | 134278399 | G | T | NCKAP5 |  | ENST00000358991 | 0 | None | intron_variant | LOW |  | None |  | None | 0.285714286 |
| chr2 | 134909276 | 134909277 | C | T | MGAT5 |  | ENST00000409645 | 1 | None | intron_variant | LOW |  | None |  | None | 0.153846154 |
| chr2 | 135040666 | 135040667 | G | A | MGAT5 |  | ENST00000409645 | 0 | None | intron_variant | LOW |  | None |  | None | 0.223880597 |
| chr2 | 135503563 | 135503564 | C | T | AC016725.4 |  | ENST00000392929 | 0 | None | intron_variant | LOW |  | None |  | None | 0.228571429 |
| chr2 | 135601618 | 135601619 | C | G | ACMSD |  | ENST00000283054 | 0 | None | intron_variant | LOW |  | None |  | None | 0.272727273 |
| chr2 | 135875343 | 135875344 | T | G | RAB3GAP1 |  | ENST00000442034 | 0 | None | intron_variant | LOW |  | None |  | None | 0.08988764 |
| chr2 | 137357686 | 137357687 | C | T | None |  |  | 0 | None | intergenic_variant | LOW |  | None |  | None | 0.25 |
| chr2 | 137504594 | 137504595 | C | T | None |  |  | 0 | None | intergenic_variant | LOW |  | None |  | None | 0.269230769 |
| chr2 | 137523498 | 137523499 | G | T | THSD7B |  | ENST00000409968 | 0 | None | intron_variant | LOW |  | None |  | None | 0.236111111 |
| chr2 | 137907149 | 137907150 | G | A | THSD7B |  | ENST00000413152 | 0 | None | intron_variant | LOW |  | None |  | None | 0.14893617 |
| chr2 | 137939521 | 137939522 | G | T | THSD7B |  | ENST00000272643 | 0 | None | intron_variant | LOW |  | None |  | None | 0.328358209 |
| chr2 | 137953230 | 137953231 | A | C | THSD7B |  | ENST00000413152 | 0 | None | intron_variant | LOW |  | None |  | None | 0.157142857 |
| chr2 | 137987062 | 137987063 | G | T | THSD7B |  | ENST00000543459 | 0 | None | intron_variant | LOW |  | None |  | None | 0.177777778 |
| chr2 | 138076258 | 138076259 | C | T | THSD7B |  | ENST00000272643 | 0 | None | intron_variant | LOW |  | None |  | None | 0.224137931 |
| chr2 | 138592460 | 138592461 | C | A | None |  |  | 0 | None | intergenic_variant | LOW |  | None |  | None | 0.12244898 |
| chr2 | 139747279 | 139747280 | C | G | None |  |  | 0 | None | intergenic_variant | LOW |  | None |  | None | 0.346938776 |
| chr2 | 140801410 | 140801411 | C | G | None |  |  | 0 | None | intergenic_variant | LOW |  | None |  | None | 0.244444444 |
| chr2 | 140858110 | 140858111 | A | G | AC073928.2 |  | ENST00000417715 | 0 | None | downstream_gene_variant | LOW |  | None |  | None | 0.145454545 |
| chr2 | 140927917 | 140927918 | C | A | None |  |  | 0 | None | intergenic_variant | LOW |  | None |  | None | 0.195652174 |
| chr2 | 140927918 | 140927919 | C | A | None |  |  | 0 | None | intergenic_variant | LOW |  | None |  | None | 0.195652174 |
| chr2 | 140972727 | 140972728 | T | A | MTND2P19 |  | ENST00000449129 | 0 | None | downstream_gene_variant | LOW |  | None |  | None | 0.170731707 |
| chr2 | 140978972 | 140978973 | A | G | AC092156.3 |  | ENST00000441935 | 0 | None | upstream_gene_variant | LOW |  | None |  | None | 0.18 |
| chr2 | 140978973 | 140978974 | G | A | MTND2P19 |  | ENST00000449129 | 0 | None | upstream_gene_variant | LOW |  | None |  | None | 0.163265306 |
| chr2 | 141145403 | 141145404 | G | A | LRP1B |  | ENST00000389484 | 0 | None | intron_variant | LOW |  | None |  | None | 0.230769231 |
| chr2 | 141233159 | 141233160 | T | C | LRP1B |  | ENST00000389484 | 0 | None | intron_variant | LOW |  | None |  | None | 0.327272727 |
| chr2 | 141715455 | 141715456 | A | G | LRP1B |  | ENST00000389484 | 0 | None | intron_variant | LOW |  | None |  | None | 0.268292683 |
| chr2 | 142011517 | 142011518 | T | C | LRP1B |  | ENST00000434794 | 0 | None | intron_variant | LOW |  | None |  | None | 0.127659574 |
| chr2 | 142064448 | 142064449 | G | T | LRP1B |  | ENST00000434794 | 0 | None | intron_variant | LOW |  | None |  | None | 0.18 |
| chr2 | 142279809 | 142279810 | A | C | LRP1B |  | ENST00000389484 | 0 | None | intron_variant | LOW |  | None |  | None | 0.256756757 |
| chr2 | 142394580 | 142394581 | T | C | LRP1B |  | ENST00000434794 | 0 | None | intron_variant | LOW |  | None |  | None | 0.209677419 |
| chr2 | 142736963 | 142736964 | T | A | LRP1B |  | ENST00000389484 | 0 | None | intron_variant | LOW |  | None |  | None | 0.163636364 |
| chr2 | 143115958 | 143115959 | G | C | None |  |  | 0 | None | intergenic_variant | LOW |  | None |  | None | 0.176470588 |
| chr2 | 143124033 | 143124034 | T | G | None |  |  | 0 | None | intergenic_variant | LOW |  | None |  | None | 0.245901639 |
| chr2 | 143125019 | 143125020 | G | T | None |  |  | 0 | None | intergenic_variant | LOW |  | None |  | None | 0.344262295 |
| chr2 | 143125545 | 143125546 | T | A | None |  |  | 0 | None | intergenic_variant | LOW |  | None |  | None | 0.228070175 |
| chr2 | 143287907 | 143287908 | T | A | None |  |  | 0 | None | intergenic_variant | LOW |  | None |  | None | 0.148148148 |
| chr2 | 143462377 | 143462378 | T | C | None |  |  | 0 | None | intergenic_variant | LOW |  | None |  | None | 0.173913043 |
| chr2 | 144556836 | 144556837 | C | T | AC079584.2 |  | ENST00000426716 | 0 | None | upstream_gene_variant | LOW |  | None |  | None | 0.175438596 |
| chr2 | 144749238 | 144749239 | G | A | GTDC1 |  | ENST00000463875 | 0 | None | intron_variant | LOW |  | None |  | None | 0.222222222 |
| chr2 | 147185678 | 147185679 | G | C | None |  |  | 0 | None | intergenic_variant | LOW |  | None |  | None | 0.181818182 |
| chr2 | 147765627 | 147765628 | C | T | None |  |  | 0 | None | intergenic_variant | LOW |  | None |  | None | 0.166666667 |
| chr2 | 147889900 | 147889901 | C | T | None |  |  | 0 | None | intergenic_variant | LOW |  | None |  | None | 0.15 |
| chr2 | 148370566 | 148370567 | G | T | None |  |  | 0 | None | intergenic_variant | LOW |  | None |  | None | 0.139534884 |
| chr2 | 149138570 | 149138571 | A | C | MBD5 |  | ENST00000407073 | 0 | None | intron_variant | LOW |  | None |  | None | 0.18 |
| chr2 | 149258617 | 149258618 | T | C | MBD5 |  | ENST00000404807 | 0 | None | intron_variant | LOW |  | None |  | None | 0.097222222 |
| chr2 | 150126652 | 150126653 | G | C | None |  |  | 0 | None | intergenic_variant | LOW |  | None |  | None | 0.327586207 |
| chr2 | 151274487 | 151274488 | G | A | None |  |  | 0 | None | intergenic_variant | LOW |  | None |  | None | 0.204081633 |
| chr2 | 151898932 | 151898933 | T | A | AC023469.1 |  | ENST00000409243 | 0 | None | intron_variant | LOW |  | None |  | None | 0.159090909 |
| chr2 | 152034428 | 152034429 | C | T | AC018731.3 |  | ENST00000447078 | 0 | None | intron_variant | LOW |  | None |  | None | 0.238095238 |
| chr2 | 153947750 | 153947751 | G | A | None |  |  | 0 | None | intergenic_variant | LOW |  | None |  | None | 0.220338983 |
| chr2 | 154112147 | 154112148 | C | T | None |  |  | 0 | None | intergenic_variant | LOW |  | None |  | None | 0.166666667 |
| chr2 | 154552657 | 154552658 | G | A | None |  |  | 0 | None | intergenic_variant | LOW |  | None |  | None | 0.127272727 |
| chr2 | 154769879 | 154769880 | G | C | GALNT13 |  | ENST00000392825 | 0 | None | intron_variant | LOW |  | None |  | None | 0.185185185 |
| chr2 | 154817138 | 154817139 | C | A | GALNT13 |  | ENST00000409237 | 0 | None | intron_variant | LOW |  | None |  | None | 0.15 |
| chr2 | 154911483 | 154911484 | T | G | GALNT13 |  | ENST00000392825 | 0 | None | intron_variant | LOW |  | None |  | None | 0.209302326 |
| chr2 | 154913910 | 154913911 | A | G | GALNT13 |  | ENST00000392825 | 0 | None | intron_variant | LOW |  | None |  | None | 0.166666667 |
| chr2 | 155079442 | 155079443 | C | T | GALNT13 |  | ENST00000409237 | 0 | None | intron_variant | LOW |  | None |  | None | 0.169811321 |
| chr2 | 155094759 | 155094760 | T | C | GALNT13 |  | ENST00000392825 | 0 | None | intron_variant | LOW |  | None |  | None | 0.340425532 |
| chr2 | 155212634 | 155212635 | G | T | GALNT13 |  | ENST00000409237 | 0 | None | intron_variant | LOW |  | None |  | None | 0.186046512 |
| chr2 | 155661476 | 155661477 | T | C | KCNJ3 |  | ENST00000295101 | 0 | None | intron_variant | LOW |  | None |  | None | 0.229508197 |
| chr2 | 155915902 | 155915903 | G | T | None |  |  | 0 | None | intergenic_variant | LOW |  | None |  | None | 0.196969697 |
| chr2 | 155954766 | 155954767 | T | A | None |  |  | 0 | None | intergenic_variant | LOW |  | None |  | None | 0.189189189 |
| chr2 | 156527101 | 156527102 | C | T | None |  |  | 0 | None | intergenic_variant | LOW |  | None |  | None | 0.288461538 |
| chr2 | 156527103 | 156527104 | C | A | None |  |  | 0 | None | intergenic_variant | LOW |  | None |  | None | 0.288461538 |
| chr2 | 157626011 | 157626012 | G | T | None |  |  | 0 | None | intergenic_variant | LOW |  | None |  | None | 0.222222222 |
| chr2 | 158225994 | 158225995 | G | A | None |  |  | 0 | None | intergenic_variant | LOW |  | None |  | None | 0.12962963 |
| chr2 | 158518044 | 158518045 | G | T | None |  |  | 0 | None | intergenic_variant | LOW |  | None |  | None | 0.2 |
| chr2 | 159127680 | 159127681 | T | G | CCDC148 |  | ENST00000283233 | 0 | None | intron_variant | LOW |  | None |  | None | 0.293103448 |
| chr2 | 159725017 | 159725018 | C | G | None |  |  | 0 | None | intergenic_variant | LOW |  | None |  | None | 0.103448276 |
| chr2 | 159725021 | 159725022 | G | A | None |  |  | 0 | None | intergenic_variant | LOW |  | None |  | None | 0.12962963 |
| chr2 | 159825644 | 159825645 | C | T | TANC1 |  | ENST00000263635 | 0 | None | intron_variant | LOW |  | None |  | None | 0.225806452 |
| chr2 | 160106966 | 160106967 | C | G | WDSUB1 |  | ENST00000358147 | 0 | None | intron_variant | LOW |  | None |  | None | 0.163636364 |
| chr2 | 160369747 | 160369748 | A | G | BAZ2B |  | ENST00000541068 | 0 | None | intron_variant | LOW |  | None |  | None | 0.188405797 |
| chr2 | 160399549 | 160399550 | T | C | BAZ2B |  | ENST00000355831 | 0 | None | intron_variant | LOW |  | None |  | None | 0.161290323 |
| chr2 | 161573413 | 161573414 | G | C | None |  |  | 0 | None | intergenic_variant | LOW |  | None |  | None | 0.245901639 |
| chr2 | 161654263 | 161654264 | C | T | None |  |  | 0 | None | intergenic_variant | LOW |  | None |  | None | 0.101449275 |
| chr2 | 161654280 | 161654281 | T | C | None |  |  | 0 | None | intergenic_variant | LOW |  | None |  | None | 0.092307692 |
| chr2 | 161665273 | 161665274 | T | C | None |  |  | 0 | None | intergenic_variant | LOW |  | None |  | None | 0.142857143 |
| chr2 | 161975988 | 161975989 | G | T | AC009313.1 |  | ENST00000425470 | 0 | None | intron_variant | LOW |  | None |  | None | 0.285714286 |
| chr2 | 162202885 | 162202886 | A | G | PSMD14 |  | ENST00000437630 | 0 | None | intron_variant | LOW |  | None |  | None | 0.229166667 |
| chr2 | 163581898 | 163581899 | C | T | KCNH7 |  | ENST00000332142 | 0 | None | intron_variant | LOW |  | None |  | None | 0.293103448 |
| chr2 | 163707953 | 163707954 | T | C | None |  |  | 0 | None | intergenic_variant | LOW |  | None |  | None | 0.212765957 |
| chr2 | 166067586 | 166067587 | A | T | None |  |  | 0 | None | intergenic_variant | LOW |  | None |  | None | 0.206349206 |
| chr2 | 166810205 | 166810206 | G | A | TTC21B | Q/* | ENST00000243344 | 0 | None | stop_gained | HIGH |  | None |  | None | 0.25862069 |
| chr2 | 167155018 | 167155019 | G | C | SCN9A |  | ENST00000454569 | 0 | None | intron_variant | LOW |  | None |  | None | 0.21875 |
| chr2 | 167337606 | 167337607 | A | G | SCN7A |  | ENST00000409855 | 0 | None | intron_variant | LOW |  | None |  | None | 0.272727273 |
| chr2 | 168033580 | 168033581 | G | A | XIRP2 |  | ENST00000420519 | 0 | None | intron_variant | LOW |  | None |  | None | 0.233333333 |
| chr2 | 170018429 | 170018430 | T | G | LRP2 |  | ENST00000263816 | 0 | None | intron_variant | LOW |  | None |  | None | 0.122807018 |
| chr2 | 171684912 | 171684913 | C | T | GAD1 |  | ENST00000456864 | 0 | None | intron_variant | LOW |  | None |  | None | 0.157894737 |
| chr2 | 171776049 | 171776050 | C | A | AC010092.1 |  | ENST00000583255 | 0 | None | downstream_gene_variant | LOW |  | None |  | None | 0.204081633 |
| chr2 | 172499999 | 172500000 | G | A | None |  |  | 0 | None | intergenic_variant | LOW |  | None |  | None | 0.237288136 |
| chr2 | 173950150 | 173950151 | A | C | MLTK |  | ENST00000539448 | 0 | None | intron_variant | LOW |  | None |  | None | 0.203125 |
| chr2 | 174562995 | 174562996 | C | T | None |  |  | 0 | None | intergenic_variant | LOW |  | None |  | None | 0.153846154 |
| chr2 | 175382450 | 175382451 | G | A | None |  |  | 0 | None | intergenic_variant | LOW |  | None |  | None | 0.272727273 |
| chr2 | 175874395 | 175874396 | G | T | CHN1 |  | ENST00000409900 | 0 | None | upstream_gene_variant | LOW |  | None |  | None | 0.296296296 |
| chr2 | 179376539 | 179376540 | T | C | PLEKHA3 |  | ENST00000234453 | 0 | None | 3_prime_UTR_variant | LOW |  | None |  | None | 0.127272727 |
| chr2 | 180076515 | 180076516 | T | C | SESTD1 |  | ENST00000428443 | 0 | None | intron_variant | LOW |  | None |  | None | 0.2 |
| chr2 | 180477704 | 180477705 | T | G | ZNF385B |  | ENST00000410066 | 0 | None | intron_variant | LOW |  | None |  | None | 0.145454545 |
| chr2 | 181703765 | 181703766 | G | A | None |  |  | 1 | None | intergenic_variant | LOW |  | None |  | None | 0.183333333 |
| chr2 | 184141071 | 184141072 | G | T | None |  |  | 0 | None | intergenic_variant | LOW |  | None |  | None | 0.240740741 |
| chr2 | 184503704 | 184503705 | C | T | None |  |  | 0 | None | intergenic_variant | LOW |  | None |  | None | 0.169230769 |
| chr2 | 185413575 | 185413576 | C | A | None |  |  | 0 | None | intergenic_variant | LOW |  | None |  | None | 0.227272727 |
| chr2 | 185522320 | 185522321 | A | G | ZNF804A |  | ENST00000302277 | 0 | None | intron_variant | LOW |  | None |  | None | 0.295454545 |
| chr2 | 185570359 | 185570360 | A | G | ZNF804A |  | ENST00000302277 | 0 | None | intron_variant | LOW |  | None |  | None | 0.152542373 |
| chr2 | 185729301 | 185729302 | T | A | ZNF804A |  | ENST00000302277 | 0 | None | intron_variant | LOW |  | None |  | None | 0.208955224 |
| chr2 | 186445490 | 186445491 | G | A | None |  |  | 0 | None | intergenic_variant | LOW |  | None |  | None | 0.263157895 |
| chr2 | 187030403 | 187030404 | A | T | AC104058.1 |  | ENST00000448028 | 0 | None | upstream_gene_variant | LOW |  | None |  | None | 0.227272727 |
| chr2 | 187739801 | 187739802 | A | T | None |  |  | 0 | None | intergenic_variant | LOW |  | None |  | None | 0.206349206 |
| chr2 | 187911858 | 187911859 | C | T | AC007319.1 |  | ENST00000412276 | 1 | None | intron_variant | LOW |  | None |  | None | 0.188679245 |
| chr2 | 188140458 | 188140459 | G | A | AC007319.1 |  | ENST00000412276 | 1 | None | intron_variant | LOW |  | None |  | None | 0.145833333 |
| chr2 | 188844876 | 188844877 | C | T | AC068718.1 |  | ENST00000434418 | 0 | None | intron_variant | LOW |  | None |  | None | 0.25 |
| chr2 | 189754135 | 189754136 | C | A | None |  |  | 0 | None | intergenic_variant | LOW |  | None |  | None | 0.174603175 |
| chr2 | 189915607 | 189915608 | C | A | COL5A2 |  | ENST00000374866 | 0 | None | intron_variant | LOW |  | None |  | None | 0.1125 |
| chr2 | 190010118 | 190010119 | T | C | COL5A2 |  | ENST00000374866 | 0 | None | intron_variant | LOW |  | None |  | None | 0.113207547 |
| chr2 | 190677701 | 190677702 | T | C | PMS1 |  | ENST00000409823 | 0 | None | intron_variant | LOW |  | None |  | None | 0.209677419 |
| chr2 | 193155491 | 193155492 | G | A | None |  |  | 0 | None | intergenic_variant | LOW |  | None |  | None | 0.169230769 |
| chr2 | 193623215 | 193623216 | C | G | PCGEM1 |  | ENST00000606314 | 0 | None | intron_variant | LOW |  | None |  | None | 0.29787234 |
| chr2 | 193728969 | 193728970 | A | G | None |  |  | 0 | None | intergenic_variant | LOW |  | None |  | None | 0.236363636 |
| chr2 | 193781168 | 193781169 | C | T | None |  |  | 0 | None | intergenic_variant | LOW |  | None |  | None | 0.235294118 |
| chr2 | 194843250 | 194843251 | T | A | None |  |  | 0 | None | intergenic_variant | LOW |  | None |  | None | 0.270833333 |
| chr2 | 195577939 | 195577940 | A | G | None |  |  | 0 | None | intergenic_variant | LOW |  | None |  | None | 0.25 |
| chr2 | 195955037 | 195955038 | G | A | None |  |  | 1 | None | intergenic_variant | LOW |  | None |  | None | 0.210526316 |
| chr2 | 196495814 | 196495815 | A | G | SLC39A10 |  | ENST00000458054 | 0 | None | intron_variant | LOW |  | None |  | None | 0.318181818 |
| chr2 | 197526319 | 197526320 | T | C | CCDC150 |  | ENST00000272831 | 0 | None | intron_variant | LOW |  | None |  | None | 0.176470588 |
| chr2 | 199673503 | 199673504 | C | T | None |  |  | 0 | None | intergenic_variant | LOW |  | None |  | None | 0.234042553 |
| chr2 | 199882849 | 199882850 | G | T | AC018717.1 |  | ENST00000456031 | 0 | None | intron_variant | LOW |  | None |  | None | 0.155555556 |
| chr2 | 200409786 | 200409787 | T | C | None |  |  | 0 | None | intergenic_variant | LOW |  | None |  | None | 0.243902439 |
| chr2 | 201712680 | 201712681 | T | G | AC005037.2 |  | ENST00000408603 | 0 | None | upstream_gene_variant | LOW |  | None |  | None | 0.259259259 |
| chr2 | 202007413 | 202007414 | C | T | CFLAR | P/L | ENST00000439154 | 0 | None | missense_variant | MED | benign | 0 | tolerated | 0.92 | 0.142857143 |
| chr2 | 202062070 | 202062071 | C | T | CASP10 |  | ENST00000374650 | 0 | None | intron_variant | LOW |  | None |  | None | 0.253968254 |
| chr2 | 202768407 | 202768408 | G | A | None |  |  | 0 | None | intergenic_variant | LOW |  | None |  | None | 0.131578947 |
| chr2 | 202853365 | 202853366 | T | C | None |  |  | 0 | None | intergenic_variant | LOW |  | None |  | None | 0.136363636 |
| chr2 | 202853549 | 202853550 | G | A | None |  |  | 1 | None | intergenic_variant | LOW |  | None |  | None | 0.101694915 |
| chr2 | 202860070 | 202860071 | A | C | None |  |  | 0 | None | intergenic_variant | LOW |  | None |  | None | 0.189189189 |
| chr2 | 203068931 | 203068932 | T | A | SUMO1 |  | ENST00000409712 | 0 | None | downstream_gene_variant | LOW |  | None |  | None | 0.240740741 |
| chr2 | 203635455 | 203635456 | T | C | FAM117B |  | ENST00000392238 | 0 | None | downstream_gene_variant | LOW |  | None |  | None | 0.15625 |
| chr2 | 204059663 | 204059664 | T | C | NBEAL1 |  | ENST00000449802 | 0 | None | intron_variant | LOW |  | None |  | None | 0.181818182 |
| chr2 | 205920816 | 205920817 | A | T | PARD3B |  | ENST00000406610 | 0 | None | intron_variant | LOW |  | None |  | None | 0.214285714 |
| chr2 | 206880625 | 206880626 | T | C | INO80D |  | ENST00000424117 | 0 | None | intron_variant | LOW |  | None |  | None | 0.292307692 |
| chr2 | 207823369 | 207823370 | A | G | CPO |  | ENST00000272852 | 0 | None | intron_variant | LOW |  | None |  | None | 0.246153846 |
| chr2 | 208639484 | 208639485 | C | T | None |  |  | 0 | None | intergenic_variant | LOW |  | None |  | None | 0.18 |
| chr2 | 210028454 | 210028455 | G | A | None |  |  | 1 | None | intergenic_variant | LOW |  | None |  | None | 0.35 |
| chr2 | 210980410 | 210980411 | C | T | KANSL1L |  | ENST00000418791 | 0 | None | intron_variant | LOW |  | None |  | None | 0.19047619 |
| chr2 | 211670589 | 211670590 | C | A | None |  |  | 0 | None | intergenic_variant | LOW |  | None |  | None | 0.1875 |
| chr2 | 211857239 | 211857240 | G | T | None |  |  | 0 | None | intergenic_variant | LOW |  | None |  | None | 0.156862745 |
| chr2 | 212173374 | 212173375 | G | A | None |  |  | 0 | None | intergenic_variant | LOW |  | None |  | None | 0.114754098 |
| chr2 | 212731013 | 212731014 | C | A | ERBB4 |  | ENST00000260943 | 0 | None | intron_variant | LOW |  | None |  | None | 0.18 |
| chr2 | 212850086 | 212850087 | G | A | ERBB4 |  | ENST00000402597 | 0 | None | intron_variant | LOW |  | None |  | None | 0.16 |
| chr2 | 212861449 | 212861450 | C | T | ERBB4 |  | ENST00000402597 | 0 | None | intron_variant | LOW |  | None |  | None | 0.157894737 |
| chr2 | 213056335 | 213056336 | T | C | ERBB4 |  | ENST00000402597 | 1 | None | intron_variant | LOW |  | None |  | None | 0.159090909 |
| chr2 | 214396741 | 214396742 | T | A | SPAG16 |  | ENST00000331683 | 0 | None | intron_variant | LOW |  | None |  | None | 0.317073171 |
| chr2 | 214788328 | 214788329 | G | T | SPAG16 |  | ENST00000451561 | 0 | None | intron_variant | LOW |  | None |  | None | 0.233333333 |
| chr2 | 215042639 | 215042640 | C | T | SPAG16 |  | ENST00000451561 | 1 | None | intron_variant | LOW |  | None |  | None | 0.192307692 |
| chr2 | 215087099 | 215087100 | C | T | SPAG16 |  | ENST00000451561 | 0 | None | intron_variant | LOW |  | None |  | None | 0.117647059 |
| chr2 | 215111093 | 215111094 | A | G | SPAG16 |  | ENST00000331683 | 0 | None | intron_variant | LOW |  | None |  | None | 0.16 |
| chr2 | 215457134 | 215457135 | A | G | AC107218.3 |  | ENST00000412896 | 0 | None | intron_variant | LOW |  | None |  | None | 0.147540984 |
| chr2 | 216208602 | 216208603 | G | C | ATIC |  | ENST00000446622 | 0 | None | intron_variant | LOW |  | None |  | None | 0.153846154 |
| chr2 | 216478230 | 216478231 | G | A | AC012668.2 |  | ENST00000422353 | 0 | None | intron_variant | LOW |  | None |  | None | 0.220588235 |
| chr2 | 217226987 | 217226988 | G | T | MARCH4 |  | ENST00000273067 | 0 | None | intron_variant | LOW |  | None |  | None | 0.236363636 |
| chr2 | 217234734 | 217234735 | G | A | MARCH4 | G | ENST00000273067 | 0 | COSM442293 | synonymous_variant | LOW |  | None |  | None | 0.253968254 |
| chr2 | 219737648 | 219737649 | G | T | WNT6 |  | ENST00000233948 | 0 | None | intron_variant | LOW |  | None |  | None | 0.157142857 |
| chr2 | 219817997 | 219817998 | G | C | AC097468.7 |  | ENST00000429343 | 0 | None | intron_variant | LOW |  | None |  | None | 0.26984127 |
| chr2 | 219891846 | 219891847 | G | A | CCDC108 |  | ENST00000410037 | 0 | None | 3_prime_UTR_variant | LOW |  | None |  | None | 0.25 |
| chr2 | 220935691 | 220935692 | A | G | None |  |  | 0 | None | intergenic_variant | LOW |  | None |  | None | 0.114285714 |
| chr2 | 220935692 | 220935693 | G | T | None |  |  | 0 | None | intergenic_variant | LOW |  | None |  | None | 0.1 |
| chr2 | 221172823 | 221172824 | G | A | AC114765.1 |  | ENST00000432993 | 1 | None | intron_variant | LOW |  | None |  | None | 0.152777778 |
| chr2 | 221569858 | 221569859 | T | C | AC067956.1 |  | ENST00000414512 | 1 | None | downstream_gene_variant | LOW |  | None |  | None | 0.088235294 |
[truncated: 411,387 more chars]
