# Supplementary material for: Next generation mapping reveals novel large genomic rearrangements in prostate cancer
Source: Oncotarget. 2017 Mar 1;8(14):23588–602. doi: 10.18632/oncotarget.15802 (PMC5410329; doi:10.18632/oncotarget.15802)
Supplement: Supplementary file 5 [file oncotarget-08-23588-s005.docx]

**Table S8. Annotation of carcinogenic potential for 23 functionally damaging somatic SNVs identified in UP2153.**

| **Gene** | **Chr#** | **Position** | **Allele** | | **Transcript** | **Str** | **mRNA** | **db**  **snp** | **Cosmic ids** | **Variant type** | **AAS** | **CanDrA^1^** | | **Transfic^2^** | | | |
| --- | --- | --- | --- | --- | --- | --- | --- | --- | --- | --- | --- | --- | --- | --- | --- | --- | --- |
|  |  |  | **Ref** | **Mut** |  |  |  |  |  |  |  | **Impact** | **Significance** | **Sift value** | **sift**  **impact** | **pph2 value** | **pph2**  **impact** |
| **NOTCH2** | **1** | **120484227** | **A** | **C** | **ENST00000256646** | **+** | **NM_024408** | **-** | **None** | **ns** | **V968G** | Passenger | 0.82425 | **2.075** | **HIGH** | **1.839** | **HIGH** |
| BNIPL | 1 | 151018289 | C | T | ENST00000368931 | + | NM_138278 | - | None | stop | R290* | NA | NA | NA | NA | NA | NA |
| OR10Z1 | 1 | 158576825 | C | G | ENST00000361284 | + | NM_001004478 | - | None | ns | I199M | Passenger | 0.81349 | 0.489 | MED | -0.843 | LOW |
| TTC21B | 2 | 166810206 | G | A | ENST00000243344 | + | NM_024753 | - | None | stop | Q4* | NA | NA | N/A | NA | NA | NA |
| NPHP1 | 2 | 110882870 | T | C | ENST00000417665 | - | - | - | None | NA | NA | NA | NA | N/A | NA | NA | NA |
| CNTN6 | 3 | 1269619 | C | A | ENST00000539053 | + | - | - | None | stop | C100* | NA | NA | N/A | NA | NA | NA |
| **CNTN6** | **3** | **1269627** | **C** | **T** | **ENST00000446702** | **+** | **-** | **-** | **None** | **ns** | **T103I** | **DRIVER** | **0.60987** | -0.261 | LOW | -0.746 | LOW |
| CNTN4 | 3 | 3078994 | C | T | ENST00000397459 | + | NM_175607 | yes | None | ns | R692W | Passenger | 0.81349 | 1.137 | MED | 0.709 | MED |
| OR11A1 | 6 | 29395210 | A | G | ENST00000377148 | + | - | - | None | ns | F70S | Passenger | 0.37303 | -0.185 | LOW | 0.67 | MED |
| **HCRTR2** | **6** | **55039534** | **C** | **G** | **ENST00000370862** | **+** | **NM_001526** | **yes** | **None** | **ns** | **P50R** | **DRIVER** | **0.62149** | 0.867 | MED | 1.209 | MED |
| **ZNF462** | **9** | **109701254** | **T** | **C** | **ENST00000457913** | **+** | **NM_021224** | **-** | **None** | **ns** | **L2098P** | Passenger | 0.81205 | **2.084** | **HIGH** | **1.818** | **HIGH** |
| CPN1 | 10 | 101835775 | C | T | ENST00000370418 | + | NM_001308 | - | None | ns | E105K | Passenger | 0.52798 | -0.535 | LOW | 0.907 | MED |
| FMNL3 | 12 | 50062319 | G | A | ENST00000293590 | + | NM_175736 | - | None | ns | P49S | Passenger | 0.61765 | 1.1 | MED | 1.459 | MED |
| KIF5A | 12 | 57957931 | G | A | ENST00000455537 | + | NM_004984 | - | COSM169125  COSM694954 | ns | R111Q | Passenger | 0.22525 | NA | NA | NA | NA |
| DAO | 12 | 109274168 | G | A | ENST00000547166 | - | - | - | None | NA | NA | NA | NA | NA | NA | NA | NA |
| ITGBL1 | 13 | 102106263 | C | A | ENST00000376180 | + | NM_004791 | - | None | ns | S43Y | Passenger | 0.64849 | 1.949 | MED | 0.745 | MED |
| UNC79 | 14 | 94152940 | G | T | ENST00000393151 | + | NM_020818 | - | COSM1516231 COSM1516230 | ns | C2143F | No-call | NA | 1.637 | MED | -1.145 | LOW |
| AHNAK2 | 14 | 105413966 | C | T | ENST00000333244 | + | NM_138420 | yes | None | ns | E2608K | Passenger | 0.43687 | -0.38 | LOW | -1.483 | LOW |
| **ATP2A1** | **16** | **28898986** | **G** | **A** | **ENST00000395503** | **+** | **NM_173201** | **-** | **COSM673841** | **ns** | G291R | Passenger | 0.50215 | 0.606 | MED | **1.858** | **HIGH** |
| CYP4F2 | 19 | 15989696 | G | C | ENST00000221700 | + | NM_001082 | yes | COSM225400 | ns | A483G | Passenger | 0.4132 | 0.73 | MED | -1.434 | LOW |
| **OCSTAMP** | **20** | **45174217** | **G** | **A** | **ENST00000279028** | **+** | **NM_080721** | **yes** | **None** | **ns** | **R266W** | **DRIVER** | **0.48883** | 1.637 | MED | 1.44 | MED |
| SLC9A8 | 20 | 48467309 | T | G | ENST00000417961 | - | - | yes | None | NA | NA | NA | NA | NA | NA | NA | NA |
| **ATRX** | **X** | **76778869** | **A** | **G** | **ENST00000373344** | **+** | **NM_000489** | **-** | **None** | **ns** | **L2237P** | **DRIVER** | **0.54675** | NA | NA | **2.342** | **HIGH** |

**Abbreviations**: chr, chromosome; Ref, reference Hg19, Mut, mutation in UP2153; Str, strand orientation; ns, non-synonymous; stop, stop gained; NA, non applicable; AAS, amino acid substitution; *, termination codon; pph2, PolyPhen2. ^1^ CanDrA classifies somatic SNVs into driver, no-call and passenger mutations, while ^2^ transFIC divides somatic mutations into low, medium and high impact based on transformed functional impact scores of SIFT, PolyPhen2 and MutationAssessor. Mutations with a greater score are more likely to be cancer drivers. Variants in **Bold** show oncogenic potential using either CanDrA (Driver) or Transfic (High impact) prediction tools.
