# Supplementary material for: Next generation mapping reveals novel large genomic rearrangements in prostate cancer
Source: Oncotarget. 2017 Mar 1;8(14):23588–602. doi: 10.18632/oncotarget.15802 (PMC5410329; doi:10.18632/oncotarget.15802)
Supplement: Supplementary file 6 [file oncotarget-08-23588-s006.docx]

**Table S11. NGM-derived somatic structural variations (SVs) in UP2153**

| **Smap Entry ID** | **Hg19 Chr** | **Hg19 Start Position** | **Hg19 End Position** | **Tumour Genome Map ID** | **Tumour Start Position** | **Tumour End Position** | **SV Type** | **Adjacent Genes** | **Potential Fusion** | **Overlap N-Fill Gap** | **DGV overlap** | **Overlap Seg Dup** | **Flanked by Seg Dup** | **Overlap Self Chain** | **Flanked By Self Chain** | **Overlap 50% Control SV** | **Overlap Control SV - any base** | **NGS Verification Notes** | **Malacard** | **GeneCard** |
| --- | --- | --- | --- | --- | --- | --- | --- | --- | --- | --- | --- | --- | --- | --- | --- | --- | --- | --- | --- | --- |
| 643 | 7 | 86,941,098 | 86,954,084 | 777 | 275,548 | 277,115 | deletion |  |  | FALSE | esv3542041, esv2660450, esv3614034 | FALSE | FALSE | FALSE | FALSE | FALSE | FALSE | MetaSV |  |  |
| 137 | 14 | 106,278,048 | 106,378,992 | 31 | 903,439 | 1,005,436 | insertion |  |  | FALSE | nsv1055016, dgv422e199, nsv1042131, nsv469723, nsv95, esv3568753, nsv566466, nsv498836, dgv2004n100, dgv2003n100, gv27n68, nsv1041518 | TRUE | FALSE | TRUE | FALSE | TRUE | TRUE | MetaSV |  |  |
| 669 | 3 | 94,496,719 | 94,520,133 | 2233 | 194,516 | 221,533 | insertion |  |  | FALSE | - | FALSE | FALSE | FALSE | FALSE | TRUE | TRUE | MetaSV |  |  |
| 349 | 23 | 99,508,641 | 99,521,404 | 2079 | 211,196 | 222,284 | deletion |  |  | FALSE | - | FALSE | FALSE | FALSE | FALSE | TRUE | TRUE | MetaSV |  |  |
| 13 | 1 | 3,088,341 | 3,100,056 | 14 | 523,652 | 536,750 | insertion | PRDM16 |  | FALSE | - | FALSE | FALSE | FALSE | FALSE | FALSE | FALSE | MetaSV | adult t-cell leukemia/ lymphoma | Protein Coding-myelodysplastic syndrome (MDS) and acute myeloid leukemia (AML) |
| 297 | 15 | 71,704,965 | 71,721,228 | 7023 | 224,135 | 234,945 | deletion | THSD4 |  | FALSE | nsv510664 | FALSE | FALSE | FALSE | FALSE | TRUE | TRUE | MetaSV | N/A | Protein Coding |
| 439 | 10 | 31,238,233 | 31,249,473 | 2829 | 79,010 | 84,825 | deletion | ZNF438 |  | FALSE | - | FALSE | FALSE | FALSE | FALSE | TRUE | TRUE | MetaSV | N/A | Protein Coding; acts as a transcriptional repressor |
| 570 | 2 | 173,164,298 | 173,194,021 | 1788 | 397,856 | 421,229 | deletion |  |  | FALSE | nsv3034 | FALSE | FALSE | FALSE | FALSE | TRUE | TRUE | MetaSV |  |  |
| 734 | 4 | 107,056,058 | 107,065,436 | 3822 | 209,874 | 212,289 | deletion | TBCK |  | FALSE | dgv9194n54,dgv9193n54,esv3564388,nsv820470,dgv890n67,esv6293,nsv819407,esv3779,esv28666,esv2728105,nsv956310,esv3601674,dgv966e199,nsv499056,dgv97n17 | FALSE | FALSE | FALSE | FALSE | TRUE | TRUE | MetaSV | N/A | Protein Coding; cell growth and cell proliferation by regulating the mammalian target of the rapamycin (mTOR) signaling pathway |
| 453 | 20 | 48,230,260 | 48,235,260 | 1285 | 327,298 | 343,915 | insertion |  |  | FALSE | - | FALSE | FALSE | FALSE | FALSE | TRUE | TRUE | No NGS evidence |  |  |
| 768 | 3 | 302,901 | 305,427 | 3540 | 242,023 | 247,106 | insertion | CHL1 |  | FALSE | esv3331462 | FALSE | FALSE | FALSE | FALSE | TRUE | TRUE | No NGS evidence | leukemia (University of Copenhagen DISEASES) | role in cancer |
| 893 | 2 | 191,483,226 | 191,492,688 | 2147 | 420,823 | 436,233 | insertion |  |  | FALSE | - | FALSE | FALSE | FALSE | FALSE | TRUE | TRUE | No NGS evidence |  |  |
| 202 | 9 | 115,821,344 | 115,826,128 | 162 | 401,914 | 423,361 | insertion |  |  | FALSE | - | FALSE | FALSE | FALSE | FALSE | TRUE | TRUE | No NGS coverage |  |  |
| 367 | 20 | 47,130,838 | 47,146,879 | 598 | 175,469 | 203,023 | insertion |  |  | FALSE | nsv3406,nsv509774 | FALSE | FALSE | TRUE | FALSE | TRUE | TRUE | Evidence of read support |  |  |
| 109 | 2 | 304,339 | 317,100 | 4119 | 295,102 | 306,763 | deletion |  |  | FALSE | - | FALSE | FALSE | FALSE | FALSE | TRUE | TRUE | Evidence of read support |  |  |
| 642 | 10 | 9,268,425 | 9,271,628 | 2427 | 213,746 | 215,945 | deletion |  |  | FALSE | - | FALSE | FALSE | FALSE | FALSE | FALSE | FALSE | Evidence of read support |  |  |
| 666 | 5 | 57,109,549 | 57,115,659 | 2826 | 376,997 | 382,105 | deletion |  |  | FALSE | - | FALSE | FALSE | FALSE | FALSE | TRUE | TRUE | Evidence of read support |  |  |
| 729 | 1 | 1,007,323 | 1,030,112 | 679 | 183,111 | 207,516 | insertion | RNF223, C1orf159 |  | FALSE | - | FALSE | FALSE | TRUE | FALSE | FALSE | FALSE | Evidence of read support | N/A | Protein Coding |
| 731 | 20 | 19,379,953 | 19,392,382 | 6756 | 120,303 | 130,932 | deletion | SLC24A3 |  | FALSE | nsv3315 | FALSE | FALSE | FALSE | FALSE | FALSE | FALSE | Evidence of read support | N/A | Protein Coding |
| 778 | 10 | 5,406,428 | 5,423,689 | 7138 | 131,570 | 150,154 | insertion | UCN3 |  | FALSE | - | FALSE | FALSE | FALSE | FALSE | FALSE | FALSE | Evidence of read support | N/A | Protein Coding |
| 727 | 8 | 115,628,083 | 115,647,623 | 1814 | 307,485 | 314,966 | deletion |  |  | FALSE | nsv611984, nsv517280, dgv12281n54, dgv7295n100, dgv7294n100, esv3544047, esv2764094, esv3572949, dgv7296n100, dgv33n14, nsv8377, esv2592263, esv2639301, dgv1302e199, nsv499200, esv3618505, nsv611995, dgv7297n100, esv3584891 | FALSE | FALSE | FALSE | FALSE | FALSE | FALSE | Evidence of read support |  |  |
| 9 | 10 | 1,271,455 | 1,286,745 | 9 | 1,554,567 | 1,572,346 | insertion | ADARB2 |  | FALSE | - | FALSE | FALSE | FALSE | FALSE | TRUE | TRUE | Evidence of read support | N/A | Protein Coding |
| 113 | 10 | 11,322,940 | 11,330,499 | 238 | 117,777 | 126,622 | insertion | CELF2 |  | FALSE | - | FALSE | FALSE | FALSE | FALSE | FALSE | FALSE | Evidence of read support | neuroblastoma | Protein Coding; Modulates the cellular apoptosis program |
| 184 | 17 | 26,781,050 | 26,828,391 | 221 | 629,361 | 678,155 | insertion | SLC13A2 |  | FALSE | - | FALSE | FALSE | TRUE | FALSE | FALSE | TRUE | Evidence of read support | cervix small cell carcinoma | epileptic encephalopathy |
| 190 | 9 | 136,418,435 | 136,442,507 | 185 | 697,977 | 725,255 | insertion | ADAMTSL2 |  | FALSE | esv2656927 | TRUE | FALSE | TRUE | FALSE | FALSE | FALSE | Evidence of read support | geleophysic dysplasia | Protein Coding; geleophysic dysplasia |
| 232 | 9 | 137,975,358 | 137,989,981 | 12 | 647,295 | 663,566 | insertion | OLFM1 |  | FALSE | - | FALSE | FALSE | FALSE | FALSE | FALSE | FALSE | Evidence of read support | neuroblastoma | Protein Coding-neuroblastoma |
| 265 | 2 | 74,006,259 | 74,020,290 | 870 | 515,167 | 519,411 | deletion | DUSP11, C2orf78 | DUSP11-C2orf78 | FALSE | nsv582211 | TRUE | FALSE | TRUE | FALSE | TRUE | TRUE | Evidence of read support | DUSP11-amyotrophic lateral sclerosis | DUSP11-cellular proliferation and differentiation |
| 345 | 18 | 38,592,432 | 38,595,489 | 114 | 696,145 | 697,991 | deletion |  |  | FALSE | - | FALSE | FALSE | FALSE | FALSE | FALSE | FALSE | Evidence of read support |  |  |
| 347 | 4 | 55,021,893 | 55,034,357 | 447 | 570,165 | 584,403 | insertion | PDGFRA |  | FALSE | - | FALSE | FALSE | FALSE | FALSE | FALSE | FALSE | Evidence of read support | gastrointestinal stromal tumor | Protein Coding-familial gastrointestinal stromal tumors, and a variety of other cancers |
| 373 | 2 | 2,289,651 | 2,311,287 | 545 | 156,156 | 179,133 | insertion | MYT1L |  | FALSE | - | FALSE | FALSE | FALSE | FALSE | FALSE | FALSE | Evidence of read support | mental retardation | Protein Coding-associated with neuronal differentiation |
| 516 | 23 | 53,084,390 | 53,112,166 | 2444 | 288,788 | 318,096 | insertion | GPR173, TSPYL2 |  | FALSE | - | FALSE | FALSE | TRUE | FALSE | FALSE | FALSE | Evidence of read support | TSPYL2-anemia, congenital dyserythropoietic | TSPYL2-may play a role in the suppression of tumor growth |
| 571 | 15 | 42,823,523 | 42,851,810 | 1673 | 380,965 | 410,536 | insertion | SNAP23, LRRC57, HAUS2 |  | FALSE | - | FALSE | FALSE | TRUE | FALSE | FALSE | FALSE | Evidence of read support | SNAP23-chief cell adenoma; | SNAP23-Protein Coding; LRRC57-Protein Coding; HAUS2-Protein Coding |
| 646 | 19 | 30,002,818 | 30,010,835 | 1030 | 285,696 | 295,687 | insertion | LOC284395 |  | FALSE | dgv6389n54 | FALSE | FALSE | FALSE | FALSE | TRUE | TRUE | Evidence of read support | N/A | ncRNA |
| 750 | 23 | 146,346,595 | 146,380,360 | 5838 | 245,510 | 287,063 | insertion | MIR510, MIR514A1, MIR514A2 |  | FALSE | nsv7138,nsv216 | TRUE | FALSE | TRUE | FALSE | TRUE | TRUE | Evidence of read support | N/A | RNA Gene |
| 838 | 23 | 56,797,358 | 56,817,061 | 2834 | 184,994 | 199,719 | deletion | LOC550643 |  | FALSE | nsv6918, esv32659, dgv4593e59, nsv206, esv22149, nsv9944, esv1000400, esv3573990 | FALSE | FALSE | TRUE | FALSE | FALSE | TRUE | Evidence of read support | N/A | N/A |
| 848 | 14 | 80,953,271 | 80,970,419 | 1344 | 96,168 | 114,760 | insertion | CEP128 |  | FALSE | - | FALSE | FALSE | FALSE | FALSE | FALSE | TRUE | Evidence of read support | N/A | Protein Coding |
| 878 | 1 | 220,091,434 | 220,096,367 | 1190 | 207,739 | 213,738 | insertion | RNU5F-1, SLC30A10 |  | FALSE | - | FALSE | FALSE | FALSE | FALSE | FALSE | TRUE | Evidence of read support | SLC30A10-hypermanganesemia with dystonia polycythemia and cirrhosis | RNU5F-RNA Gene; SLC30A10-Protein Coding |
| 35 | 2 | 3,417,150 | 3,449,491 | 75 | 444,382 | 479,994 | insertion | TRAPPC12 |  | FALSE | nsv520390 | FALSE | FALSE | FALSE | FALSE | FALSE | TRUE | Evidence of read support | N/A | Protein Coding |
| 40 | 8 | 40,429,172 | 40,449,806 | 315 | 990,442 | 1,017,254 | insertion | ZMAT4 |  | FALSE | - | FALSE | FALSE | FALSE | FALSE | FALSE | FALSE | Evidence of read support | hematological malignancy | Protein Coding |
| 130 | 9 | 95,967,220 | 95,984,068 | 218 | 318,515 | 336,584 | insertion | WNK2 |  | FALSE | nsv950897 | FALSE | FALSE | FALSE | FALSE | TRUE | TRUE | Evidence of read support | N/A | Protein Coding-the regulation of electrolyte homeostasis, cell signaling survival, and proliferation |
| 209 | 10 | 133,965,015 | 133,988,832 | 1206 | 205,521 | 232,714 | insertion | JAKMIP3 |  | FALSE | nsv552503 | FALSE | FALSE | FALSE | FALSE | TRUE | TRUE | Evidence of read support | N/A | Protein Coding |
| 214 | 5 | 144,327,089 | 144,336,740 | 745 | 1,064,785 | 1,075,930 | insertion |  |  | FALSE | - | FALSE | FALSE | FALSE | FALSE | FALSE | FALSE | Evidence of read support |  |  |
| 220 | 6 | 43,697,075 | 43,714,589 | 746 | 494,126 | 512,815 | insertion |  |  | FALSE | - | FALSE | FALSE | FALSE | FALSE | TRUE | TRUE | Evidence of read support |  |  |
| 224 | 8 | 124,673,497 | 124,690,391 | 635 | 703,547 | 721,497 | insertion |  |  | FALSE | - | FALSE | FALSE | FALSE | FALSE | FALSE | FALSE | Evidence of read support |  |  |
| 256 | 2 | 42,047,849 | 42,052,107 | 1371 | 258,595 | 268,970 | insertion |  |  | FALSE | - | FALSE | FALSE | FALSE | FALSE | TRUE | TRUE | Evidence of read support |  |  |
| 272 | 2 | 57,451,438 | 57,461,196 | 110 | 754,946 | 768,178 | insertion |  |  | FALSE | esv3590918, esv3590917, nsv508839 | FALSE | FALSE | FALSE | FALSE | TRUE | TRUE | Evidence of read support |  |  |
| 321 | 9 | 79,559,148 | 79,614,645 | 230 | 904,549 | 962,002 | insertion |  |  | FALSE | - | FALSE | FALSE | FALSE | FALSE | FALSE | FALSE | Evidence of read support |  |  |
| 343 | 6 | 3,219,861 | 3,224,849 | 612 | 301,893 | 308,247 | insertion | TUBB2B |  | FALSE | - | FALSE | TRUE | FALSE | TRUE | FALSE | FALSE | Evidence of read support | N/A | Protein Coding-associated with TUBB2B include polymicrogyria due to tubb2b mutation |
| 378 | 13 | 103,335,686 | 103,351,437 | 320 | 88,656 | 106,227 | insertion | METTL21C |  | FALSE | esv3580867 | FALSE | FALSE | FALSE | FALSE | TRUE | TRUE | Evidence of read support | N/A | Protein Coding |
| 469 | 2 | 13,175,289 | 13,191,347 | 1544 | 707,024 | 724,749 | insertion |  |  | FALSE | - | FALSE | FALSE | FALSE | FALSE | TRUE | TRUE | Evidence of read support |  |  |
| 611 | 8 | 73,018,692 | 73,031,877 | 1021 | 83,973 | 98,528 | insertion |  |  | FALSE | - | FALSE | FALSE | TRUE | FALSE | TRUE | TRUE | Evidence of read support |  |  |
| 651 | 3 | 175,936,086 | 175,977,362 | 4770 | 235,028 | 282,063 | insertion |  |  | FALSE | dgv4993n100, nsv527268,nsv508966, nsv4128, nsv592615 | FALSE | FALSE | FALSE | FALSE | TRUE | TRUE | Evidence of read support |  |  |
| 664 | 16 | 86,877,765 | 86,883,481 | 2089 | 197,423 | 204,276 | insertion |  |  | FALSE | - | FALSE | FALSE | FALSE | FALSE | FALSE | FALSE | Evidence of read support |  |  |
| 679 | 4 | 56,591,752 | 56,636,612 | 1790 | 191,109 | 239,139 | insertion |  |  | FALSE | nsv4346, nsv509004 | FALSE | FALSE | FALSE | FALSE | TRUE | TRUE | Evidence of read support |  |  |
| 716 | 21 | 22,455,183 | 22,480,979 | 2890 | 257,437 | 286,317 | insertion | NCAM2 |  | FALSE | - | FALSE | FALSE | FALSE | FALSE | TRUE | TRUE | Evidence of read support | N/A | Protein Coding |
| 738 | 7 | 148,023,873 | 148,035,461 | 3485 | 670,545 | 684,514 | insertion | CNTNAP2 |  | FALSE | - | FALSE | FALSE | FALSE | FALSE | TRUE | TRUE | Evidence of read support | autism susceptibility | Protein Coding; implicated in multiple neurodevelopmental disorders |
| 762 | 12 | 121,489,534 | 121,491,607 | 2714 | 163,232 | 166,349 | insertion |  |  | FALSE | - | FALSE | FALSE | FALSE | FALSE | FALSE | FALSE | Evidence of read support |  |  |
| 805 | 23 | 77,509,625 | 77,526,652 | 5996 | 116,609 | 135,829 | insertion |  |  | FALSE | esv3558998 | FALSE | FALSE | TRUE | FALSE | FALSE | FALSE | Evidence of read support |  |  |
| 879 | 23 | 66,980,447 | 66,992,317 | 5977 | 131,820 | 145,957 | insertion |  |  | FALSE | - | FALSE | FALSE | TRUE | FALSE | TRUE | TRUE | Evidence of read support |  |  |
| 331 | 5 | 43,277,337 | 43,352,883 | 104 | 1,010,068 | 1,084,437 | deletion | NIM1, HMGCS1 |  | FALSE | - | TRUE | FALSE | TRUE | FALSE | FALSE | FALSE | Evidence of read support | N/A | Protein Coding |
| 620 | 5 | 120,856,655 | 120,873,898 | 1960 | 139,854 | 155,601 | deletion |  |  | FALSE | - | FALSE | FALSE | FALSE | FALSE | FALSE | FALSE | Evidence of read support |  |  |
| 742 | 23 | 13,900,126 | 13,906,052 | 3186 | 281,050 | 285,895 | deletion | GPM6B |  | FALSE | esv991320 | FALSE | FALSE | TRUE | FALSE | FALSE | FALSE | Evidence of read support | rett syndrome | Protein Coding |
| 352 | 7 | 152,549,857 | 152,574,294 | 401 | 688,240 | 706,700 | deletion | ACTR3B |  | FALSE | esv3615532, nsv508491 | FALSE | FALSE | TRUE | FALSE | FALSE | FALSE | Evidence of read support | lung adenocarcinoma | Protein Coding; May decrease the metastatic potential of tumors |
| 522 | 8 | 1,507,732 | 1,531,483 | 762 | 89,489 | 111,904 | deletion | DLGAP2 |  | FALSE | nsv950273, nsv6045 | FALSE | FALSE | TRUE | FALSE | FALSE | FALSE | Evidence of read support | N/A | Protein Coding |
| 562 | 23 | 151,076,623 | 151,096,892 | 4061 | 248,390 | 266,603 | deletion | MAGEA4 |  | FALSE | nsv953706, esv1005324, nsv528228 | TRUE | FALSE | TRUE | FALSE | FALSE | FALSE | Evidence of read support | malignant melanoma | Protein Coding; may play a role in embryonal development and tumor transformation or aspects of tumor progression |
| 658 | 1 | 165,834,901 | 165,858,764 | 3962 | 182,367 | 207,485 | insertion | UCK2 |  | FALSE | nsv437246 | FALSE | FALSE | FALSE | FALSE | FALSE | FALSE | Evidence of read support | testicular germ cell tumor | Protein Coding-testicular germ cell tumor |
| 828 | 4 | 150,385,159 | 150,390,891 | 3068 | 108,644 | 113,328 | deletion |  |  | FALSE | - | FALSE | FALSE | FALSE | FALSE | FALSE | FALSE | Evidence of read support |  |  |
| 329 | 13 | 78,266,678 | 78,284,667 | 1246 | 197,370 | 216,380 | insertion | SLAIN1, MIR3665 |  | FALSE | nsv951899 | FALSE | FALSE | FALSE | FALSE | FALSE | FALSE | Evidence of read support | N/A | SLAIN1-Protein Coding; MIR3665-RNA Gene |
| 546 | 22 | 28,588,686 | 28,644,354 | 4141 | 181,664 | 236,235 | deletion | TTC28 |  | FALSE | - | FALSE | FALSE | FALSE | FALSE | FALSE | FALSE | Evidence of read support | N/A | Protein Coding |
| 420 | 12 | 13,532,812 | 13,564,523 | 1701 | 246,406 | 271,930 | deletion |  |  | FALSE | nsv508666, nsv78, esv33935 | FALSE | FALSE | FALSE | FALSE | TRUE | TRUE | Evidence of read support |  |  |
| 114 | 6 | 164,552,918 | 164,574,139 | 56 | 777,746 | 797,000 | deletion |  |  | FALSE | - | FALSE | FALSE | FALSE | FALSE | FALSE | FALSE | Evidence of read support |  |  |
| 203 | 11 | 93,969,785 | 93,983,256 | 386 | 341,029 | 388,452 | insertion |  |  | FALSE | - | FALSE | FALSE | FALSE | FALSE | FALSE | TRUE | Evidence of read support |  |  |
| 447 | 6 | 16,608,573 | 16,616,316 | 2525 | 323,233 | 329,831 | deletion | ATXN1 |  | FALSE | - | FALSE | FALSE | FALSE | FALSE | FALSE | FALSE | Evidence of read support | spinocerebellar ataxia | Protein Coding |
| 182 | 15 | 77,981,644 | 77,995,101 | 541 | 220,304 | 236,089 | insertion | LINGO1 |  | FALSE | - | FALSE | FALSE | TRUE | FALSE | TRUE | TRUE | Scaffold support | benign essential tremor | Protein Coding-related pathways are Signaling by GPCR and Interleukin receptor SHC signaling |
| 246 | 12 | 108,962,424 | 108,976,667 | 52 | 558,753 | 578,280 | insertion | ISCU |  | FALSE | nsv509475, nsv882 | FALSE | FALSE | TRUE | FALSE | TRUE | TRUE | Scaffold support | pulmonary siderosis | necessary for several mitochondrial enzymes |
| 302 | 14 | 86,374,836 | 86,394,305 | 459 | 501,050 | 526,365 | insertion |  |  | FALSE | nsv509542 | FALSE | FALSE | FALSE | FALSE | TRUE | TRUE | Scaffold support |  |  |
| 404 | 6 | 51,728,532 | 51,761,982 | 360 | 776,672 | 803,869 | deletion | PKHD1 |  | FALSE | - | FALSE | FALSE | FALSE | FALSE | TRUE | TRUE | Scaffold support | polycystic kidney and hepatic disease | Protein Coding-acts in collecting-duct and biliary differentiation |
| 423 | 17 | 64,632,985 | 64,638,018 | 648 | 93,897 | 105,432 | insertion | PRKCA |  | FALSE | - | FALSE | FALSE | FALSE | FALSE | TRUE | TRUE | Scaffold support | glioma (Novoseek) | a class of tumor promoters. |
| 601 | 12 | 106,062,835 | 106,074,557 | 3393 | 135,819 | 148,691 | insertion |  |  | FALSE | - | FALSE | FALSE | FALSE | FALSE | FALSE | FALSE | Scaffold support |  |  |
| 616 | 3 | 151,147,842 | 151,160,926 | 5364 | 242,621 | 261,407 | insertion | MED12L, IGSF10 |  | FALSE | nsv508960 | FALSE | FALSE | FALSE | FALSE | TRUE | TRUE | Scaffold support | MED12L-usher syndrome | Protein Coding |
| 639 | 23 | 83,798,409 | 83,807,891 | 4028 | 186,308 | 197,528 | insertion |  |  | FALSE | - | FALSE | FALSE | TRUE | FALSE | FALSE | FALSE | Scaffold support |  |  |
| 701 | 13 | 61,452,909 | 61,463,644 | 3678 | 185,509 | 202,220 | insertion |  |  | FALSE | - | FALSE | FALSE | FALSE | FALSE | TRUE | TRUE | Scaffold support |  |  |
| 791 | 5 | 99,198,534 | 99,210,373 | 3377 | 96,923 | 116,024 | insertion |  |  | FALSE | - | FALSE | FALSE | TRUE | FALSE | TRUE | TRUE | Scaffold support |  |  |
| 811 | 3 | 186,353,536 | 186,372,499 | 4433 | 87,446 | 112,243 | insertion | FETUB |  | FALSE | - | FALSE | FALSE | FALSE | FALSE | FALSE | TRUE | Scaffold support | N/A | Protein Coding |
| 837 | 23 | 83,326,566 | 83,340,767 | 6986 | 145,983 | 161,432 | insertion | RPS6KA6 |  | FALSE | - | FALSE | FALSE | TRUE | FALSE | FALSE | FALSE | Scaffold support | N/A | Protein Coding-may participate in p53/TP53-dependent cell growth arrest signaling and play an inhibitory role during embryogenesis |
